# Supplementary material for: Asymmetric cyanation of imines via dipeptide-derived organophosphine dual-reagent catalysis
Source: Nat Commun. 2016 Sep 14;7:12720. doi: 10.1038/ncomms12720 (PMC5027285; doi:10.1038/ncomms12720)
Supplement: Supplementary Information — Supplementary Figures 1-77, Supplementary Tables 1-2, Supplementary Methods and Supplementary References [file ncomms12720-s1.pdf]

Supplementary Figure 1.The racemic cyanation of the ketoimine derived from isatin.

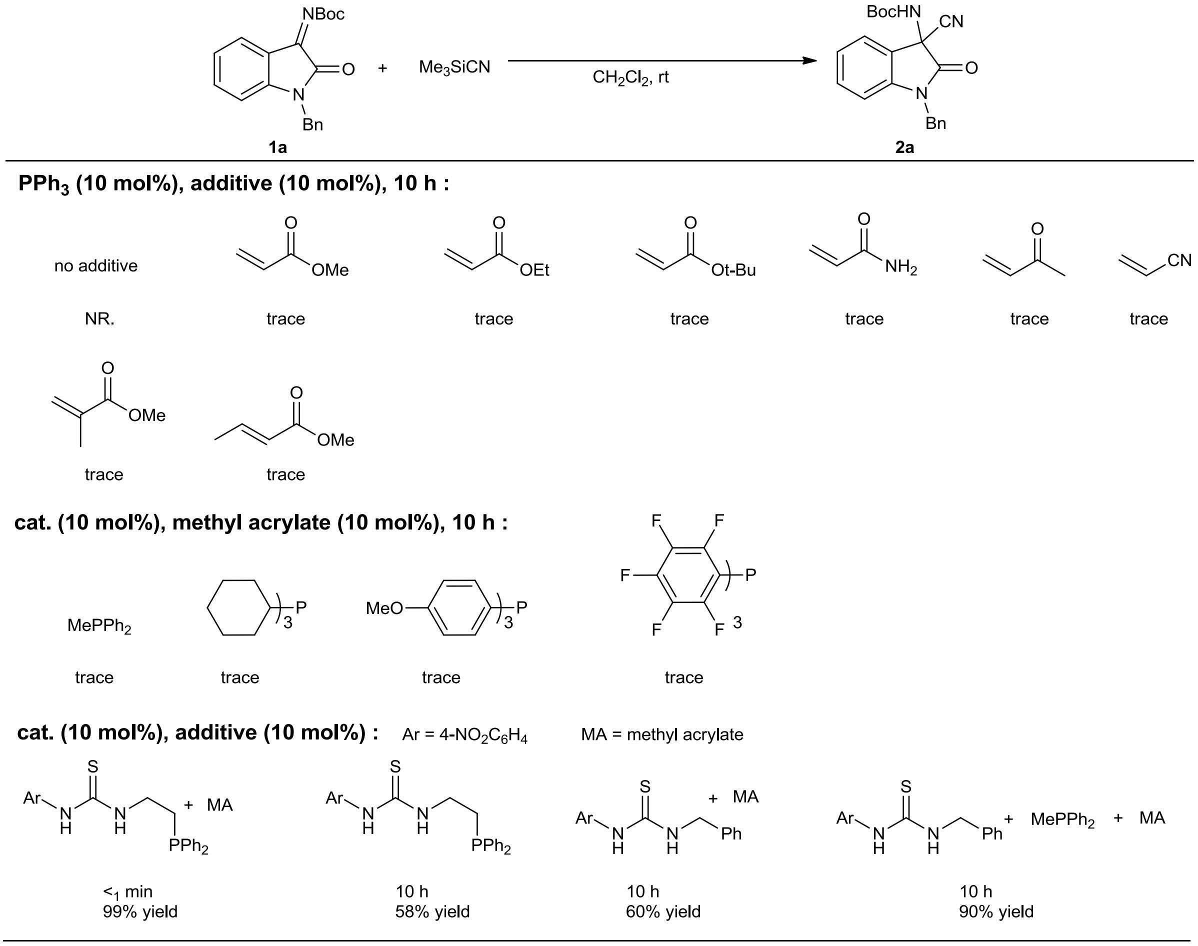

Supplementary Figure 2. The scope of the cyanation of aldimines with different protecting groups.

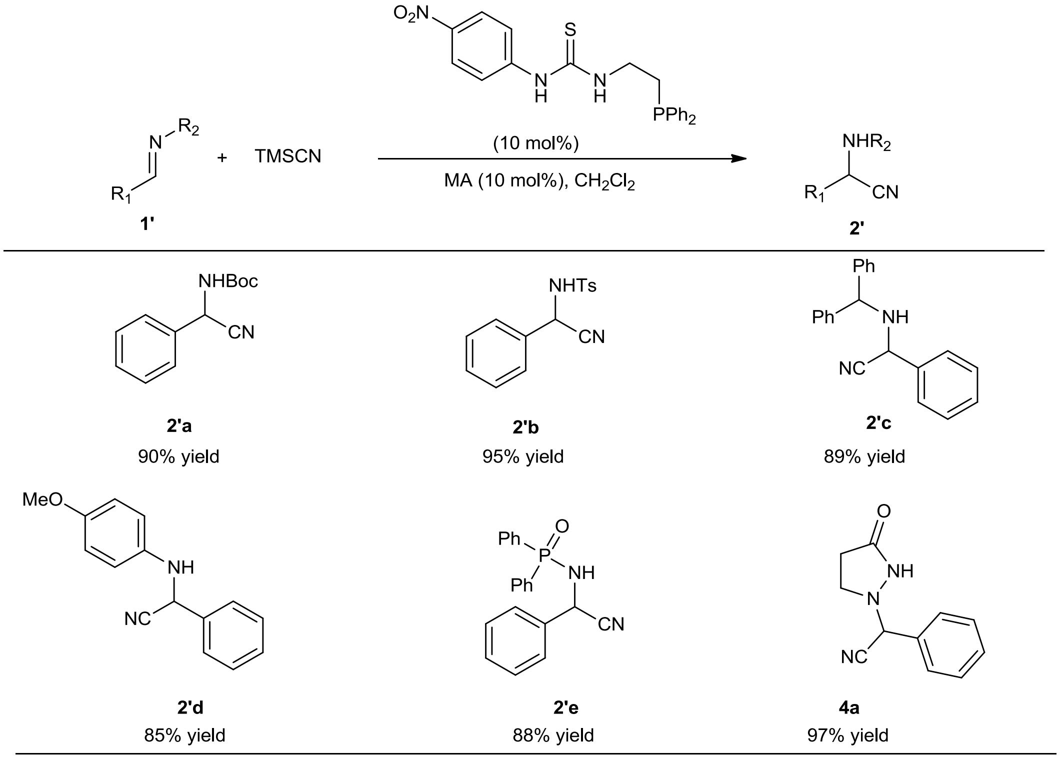

Supplementary Figure 3. The asymmetric cyanation of different imines.

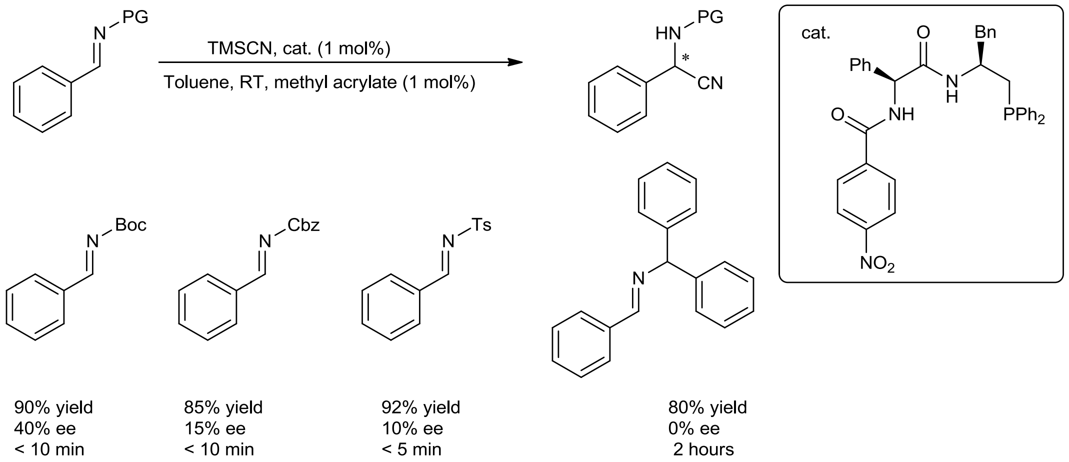

Supplementary Figure 4. The ESI-MS spectra.

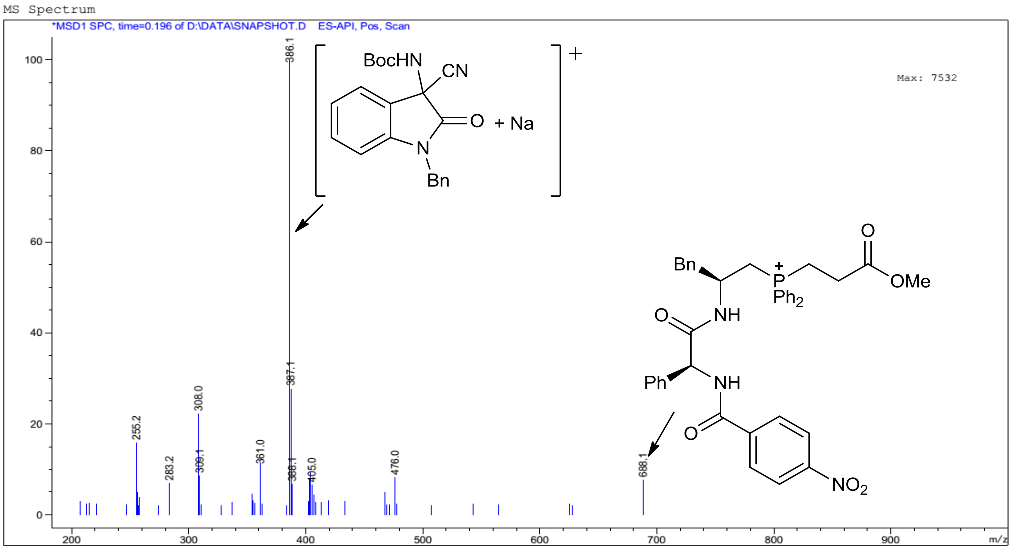

Supplementary Figure 5. The ^{31}P-NMR spectra.

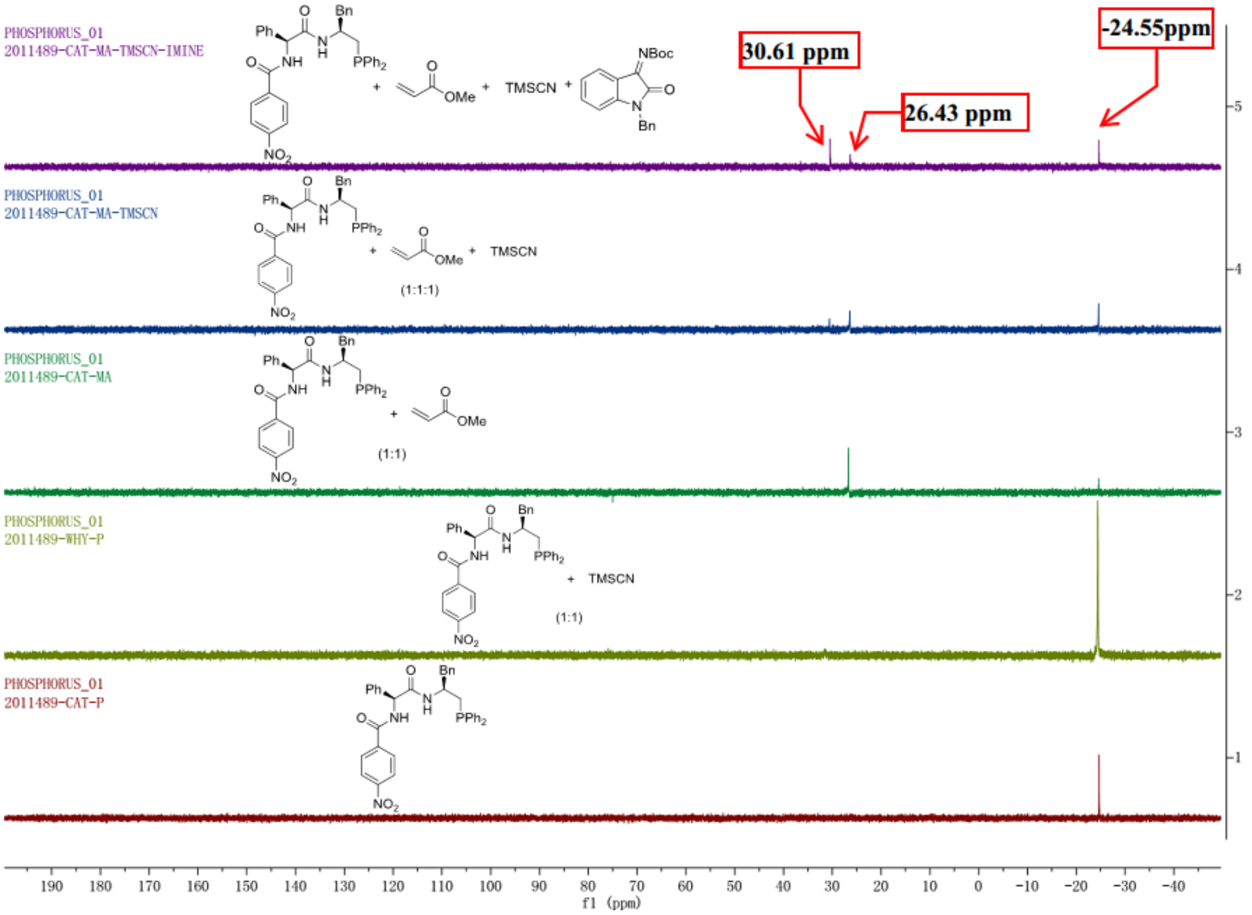

Supplementary Figure 6. The possible transition states.

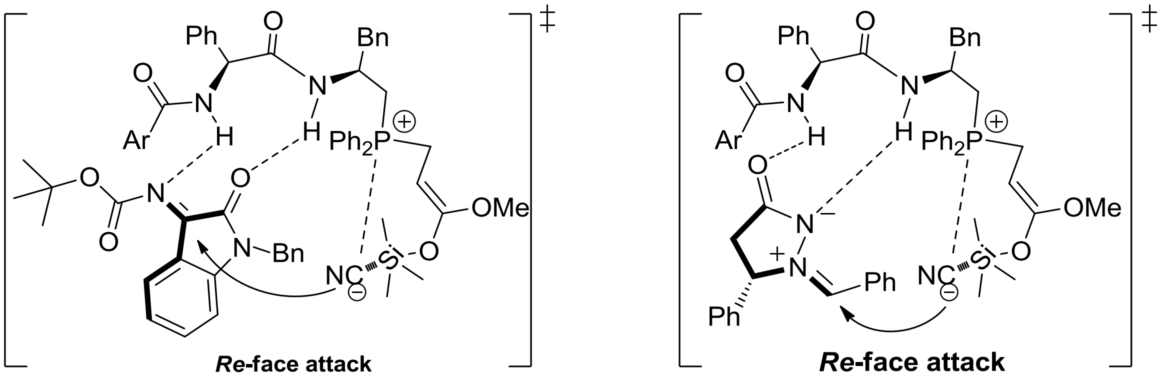

A **B**

Possible transition states. **A**: a new tight ion pair between the zwitterion and Me_3SiCN with hypervalentpentacoordinated silicon was generated, and the hydrogen-bonding interaction between the double amide N-H and ketoimine controls the reaction to happen from the *Re* face; **B**: the reaction with (±)-**6a** was preferable to proceed with the (*S*)-**6a** as the phenyl group at 3-position does not block the *Re*-face of the imine.

Supplementary Figure 7. ^{1}H and ^{13}C-NMR spectra of catalyst **3j**.

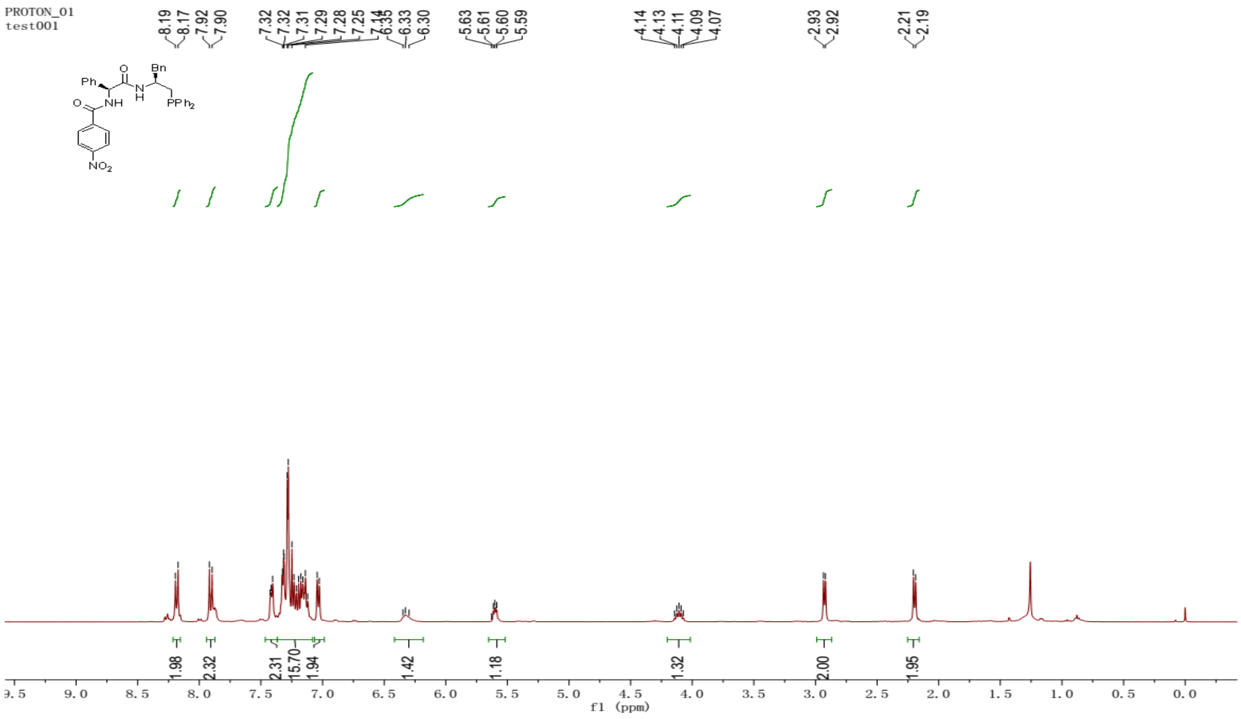

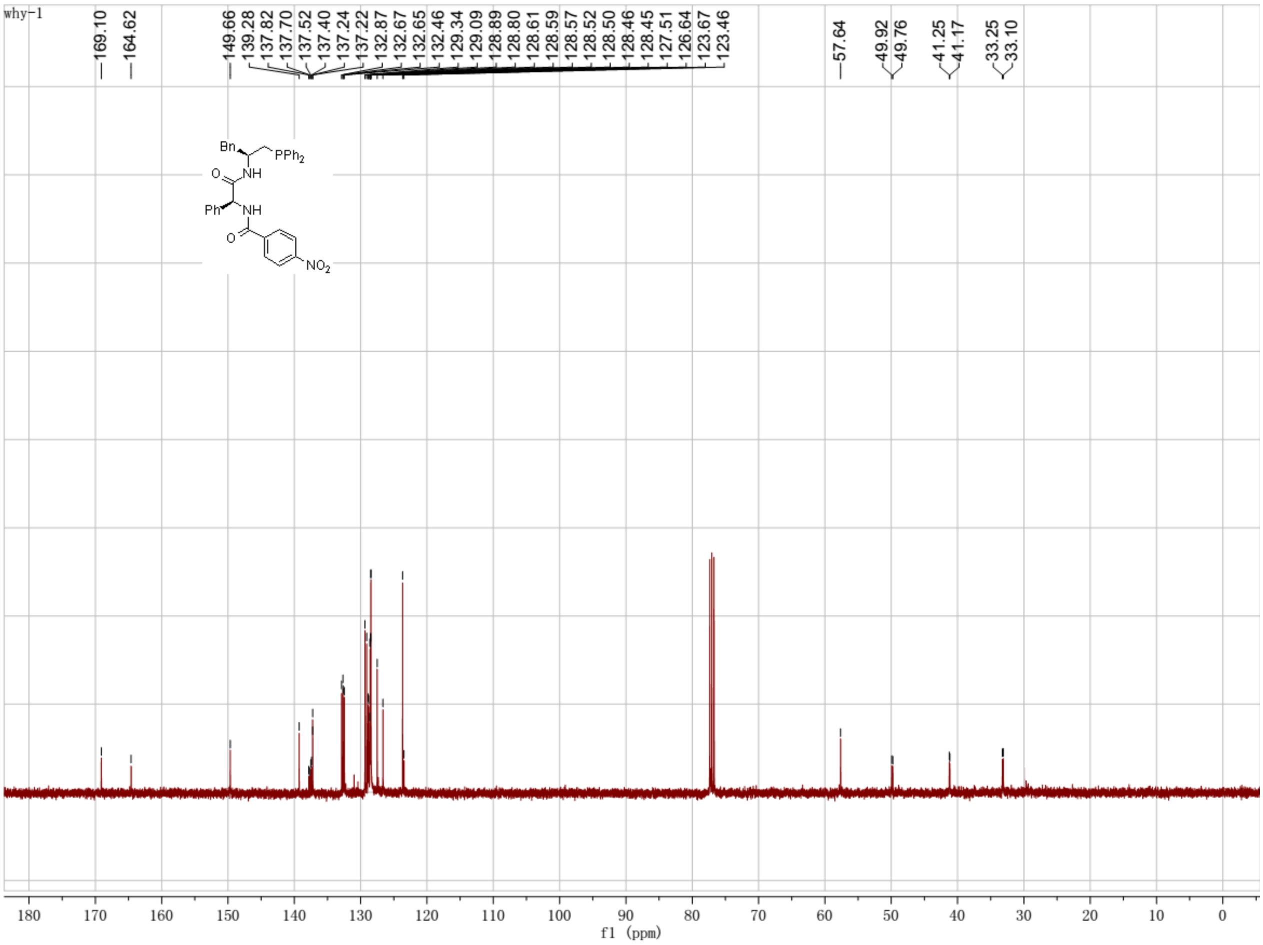

Supplementary Figure 8. ^{1}H and ^{13}C-NMR spectra of catalyst **3k**.

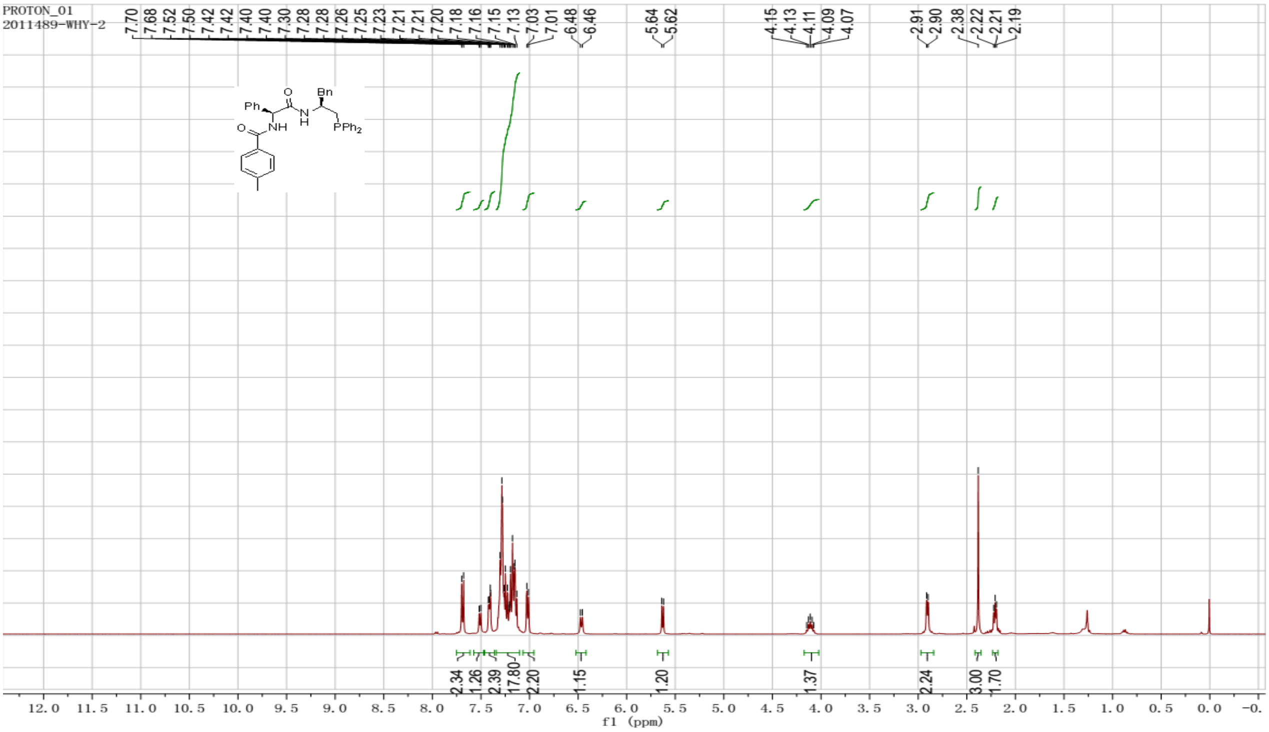

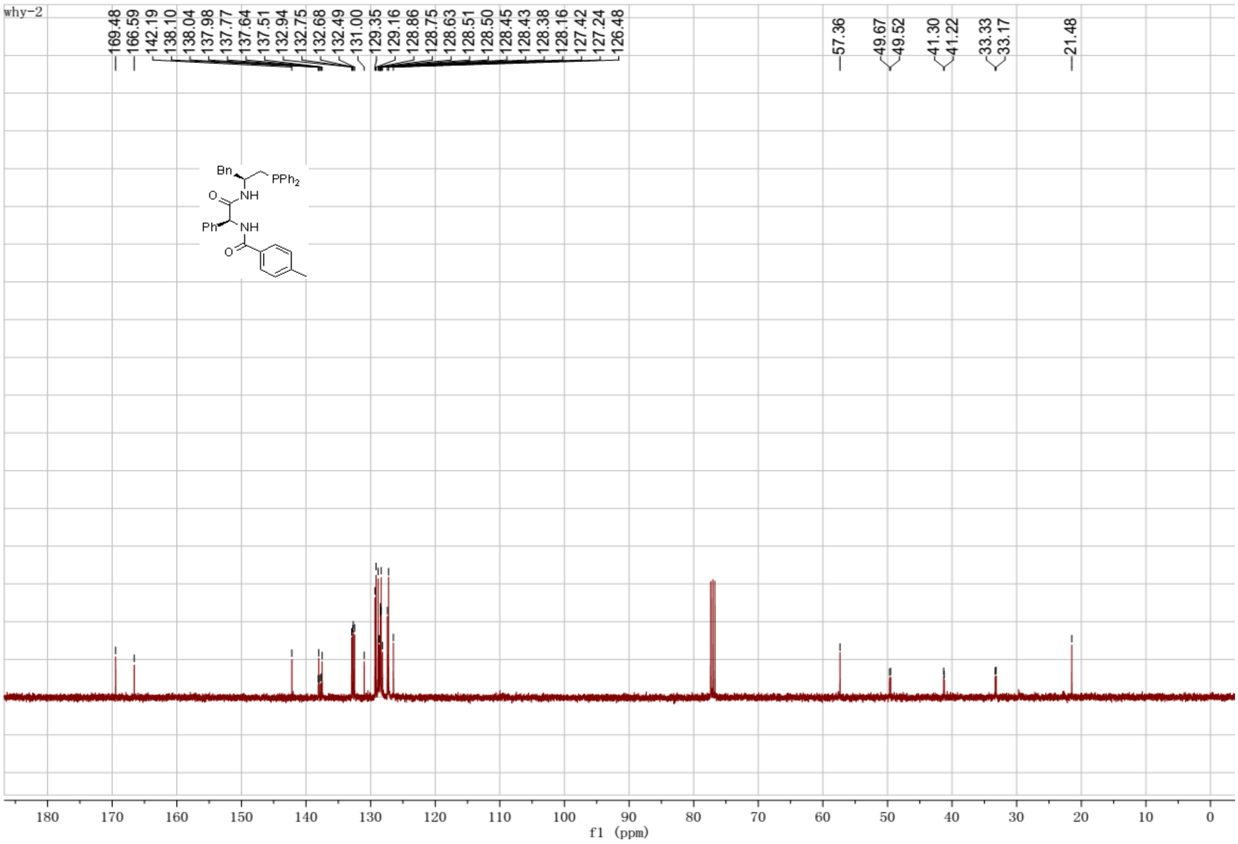

Supplementary Figure 9. ^{1}H and ^{13}C-NMR spectra of catalyst **3l**.

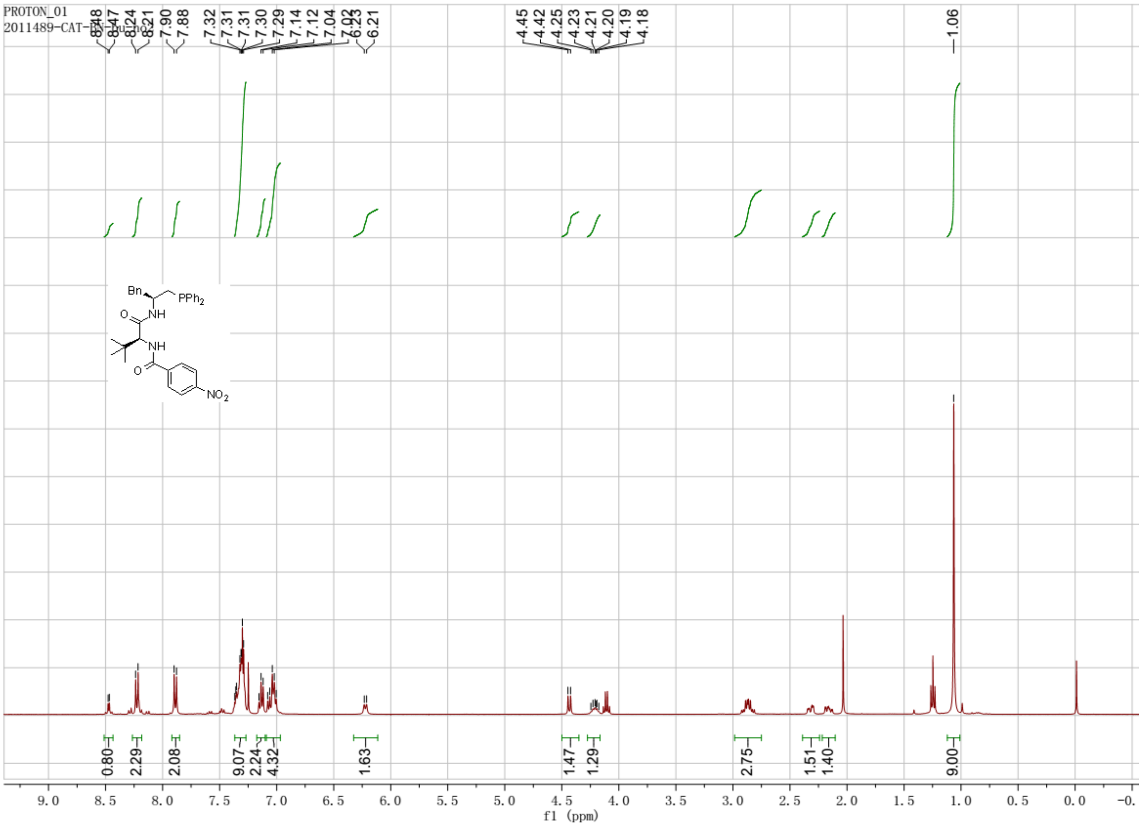

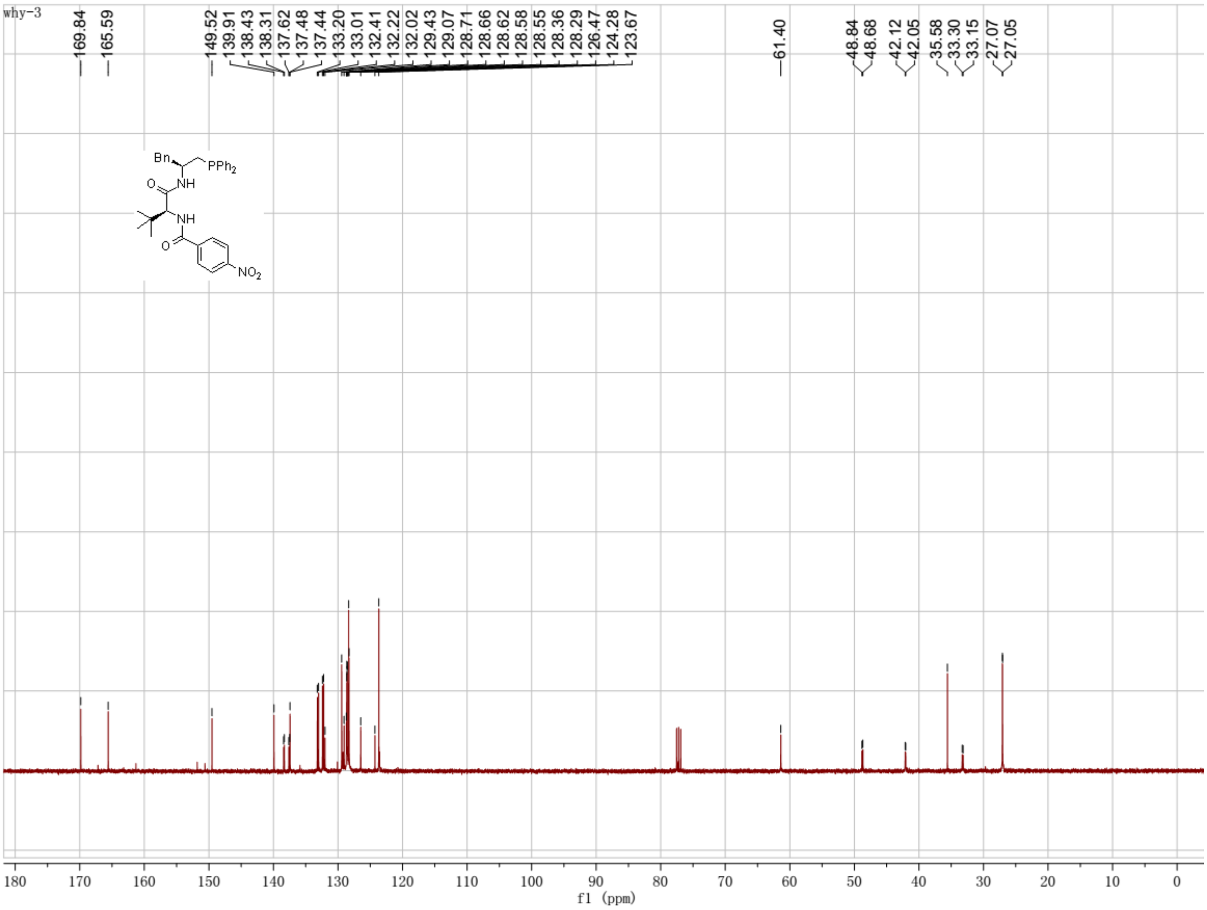

Supplementary Figure 10. ^{1}H and ^{13}C-NMR spectra of catalyst **3m**.

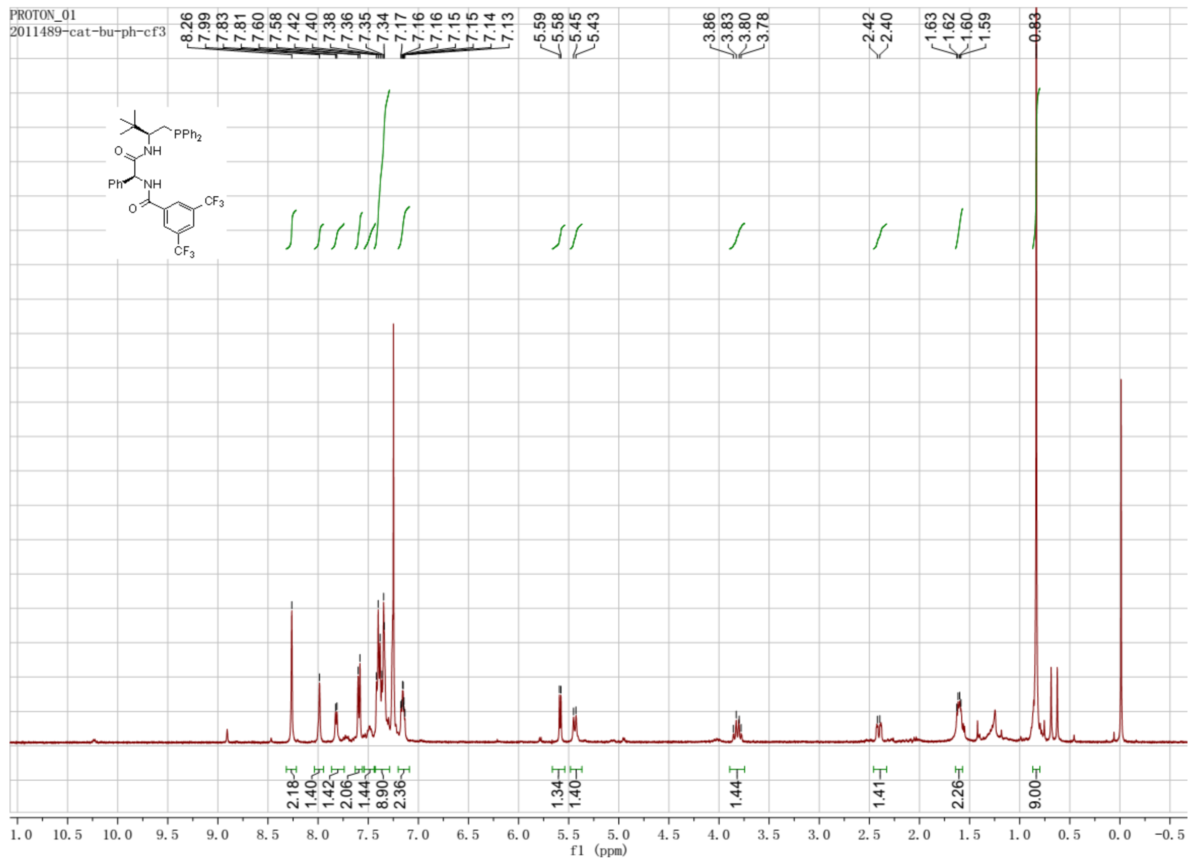

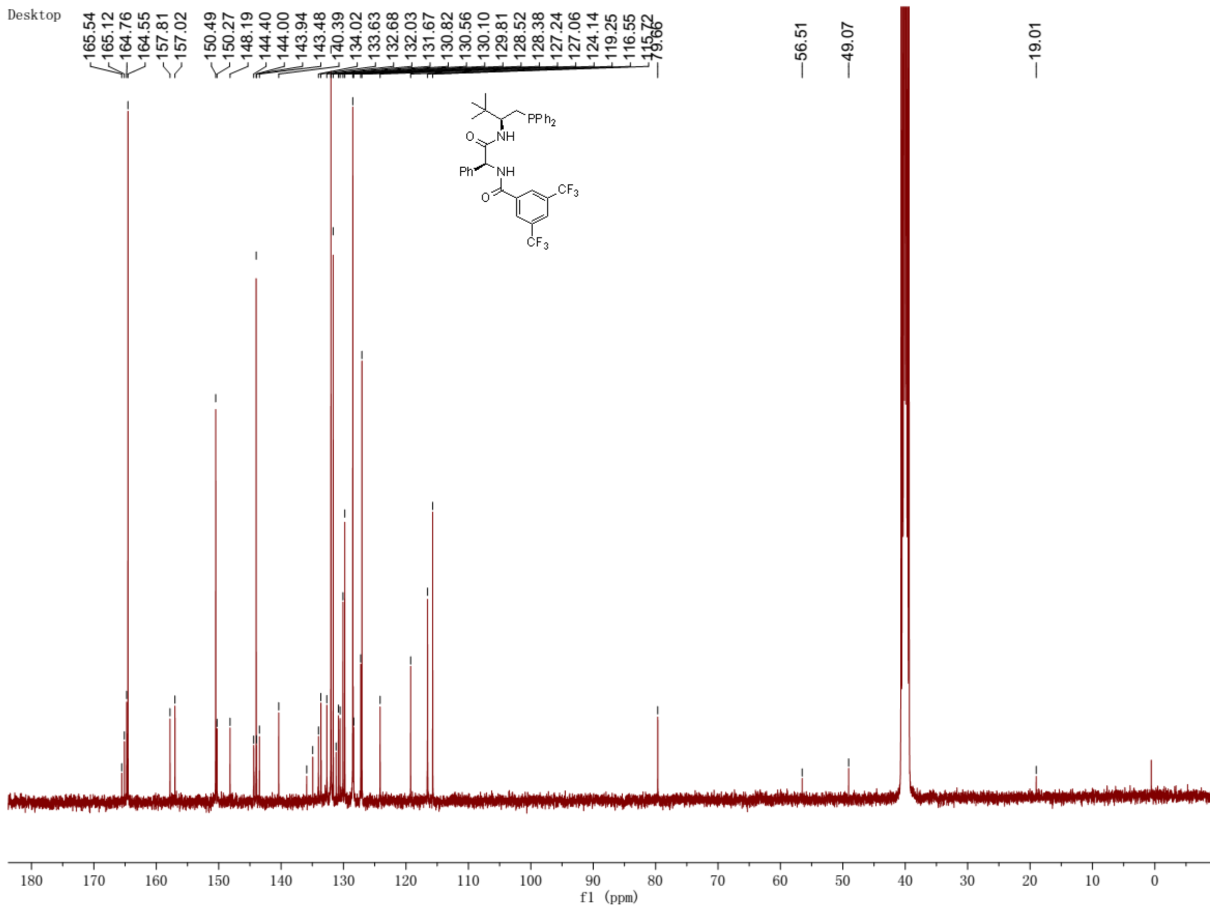

Supplementary Figure 11. ^{1}H and ^{13}C-NMR spectra of catalyst **3n**.

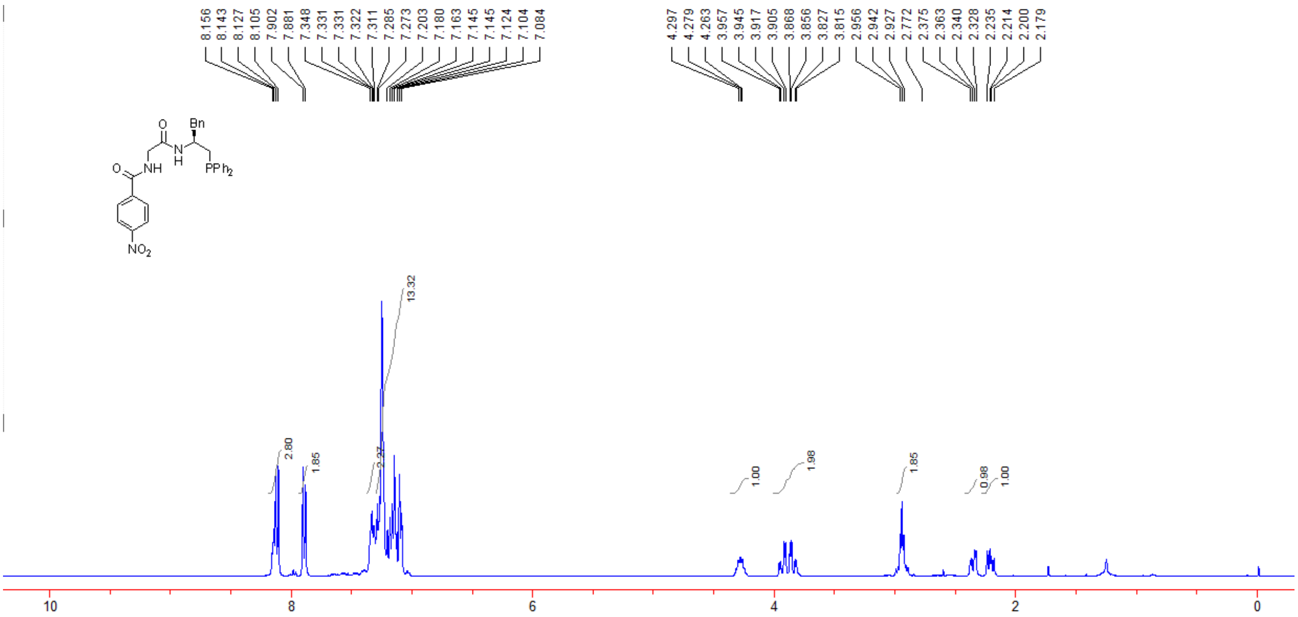

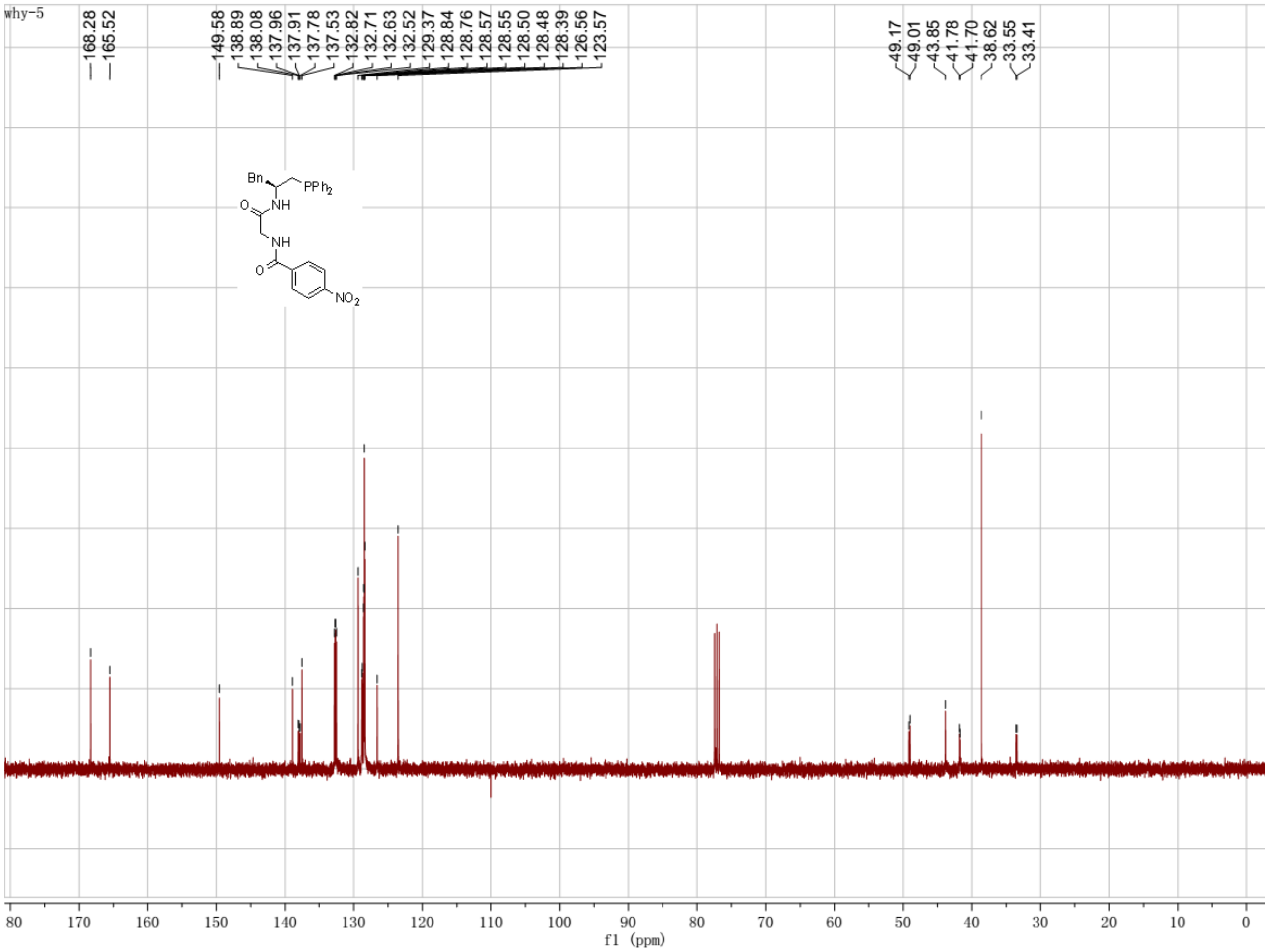

Supplementary Figure 12. ^{1}H and ^{13}C-NMR spectra of catalyst **3o**.

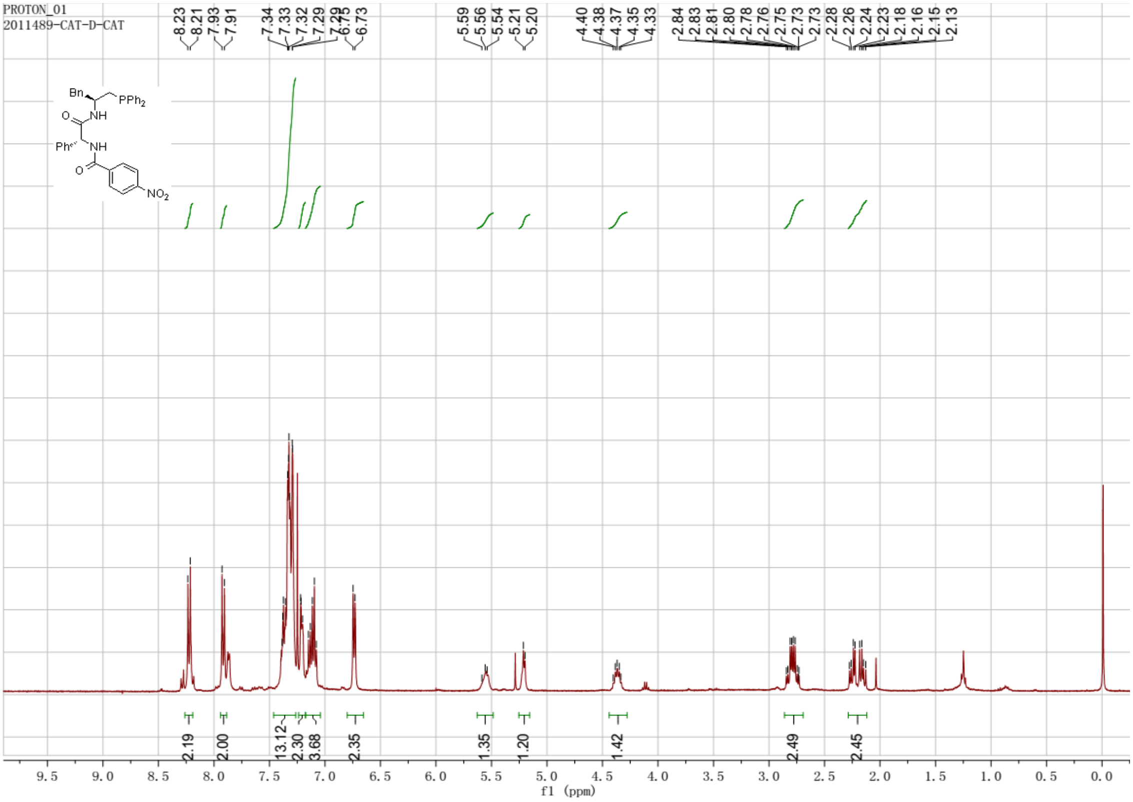

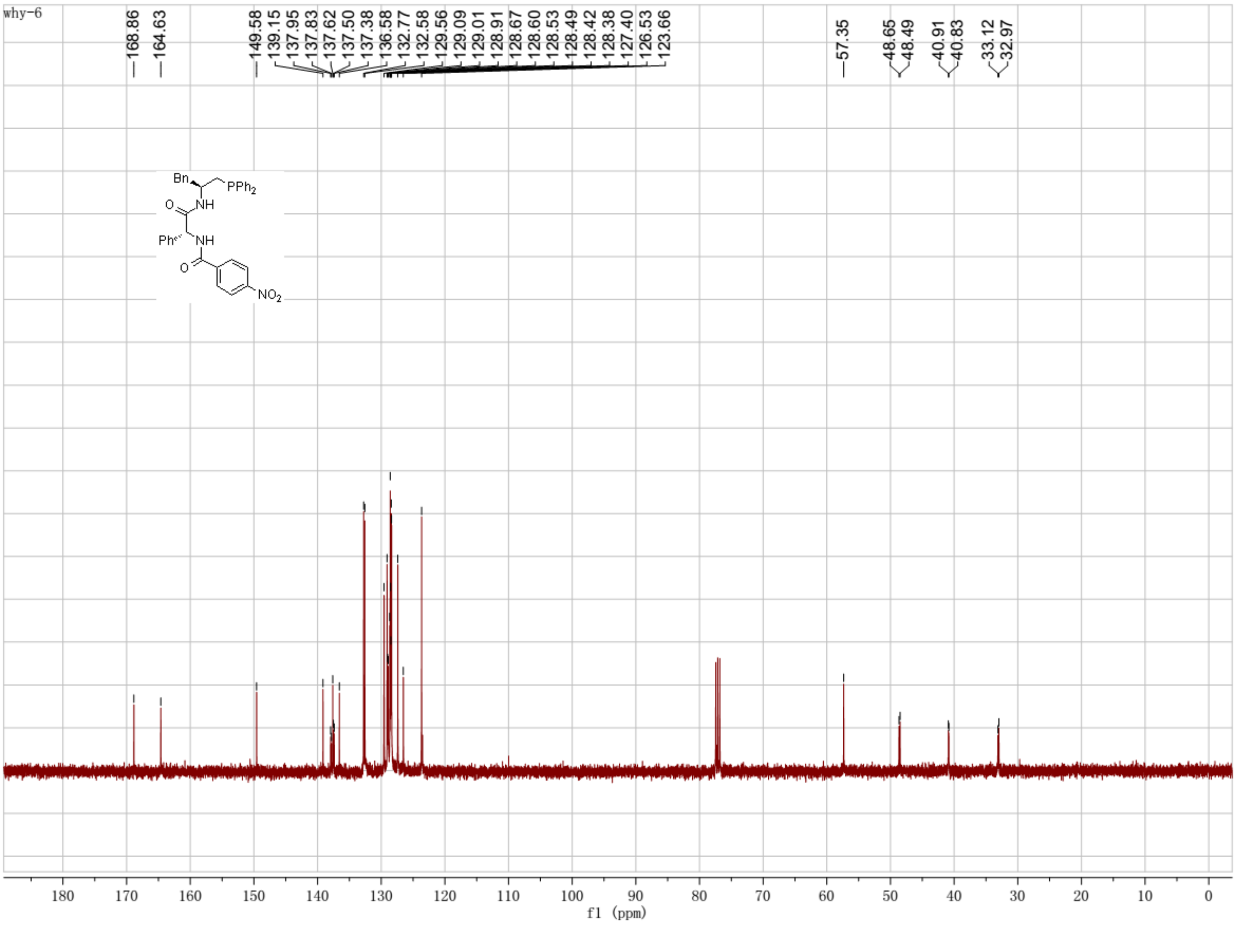

Supplementary Figure 13. ^{1}H and ^{13}C-NMR spectra of product **2d**.

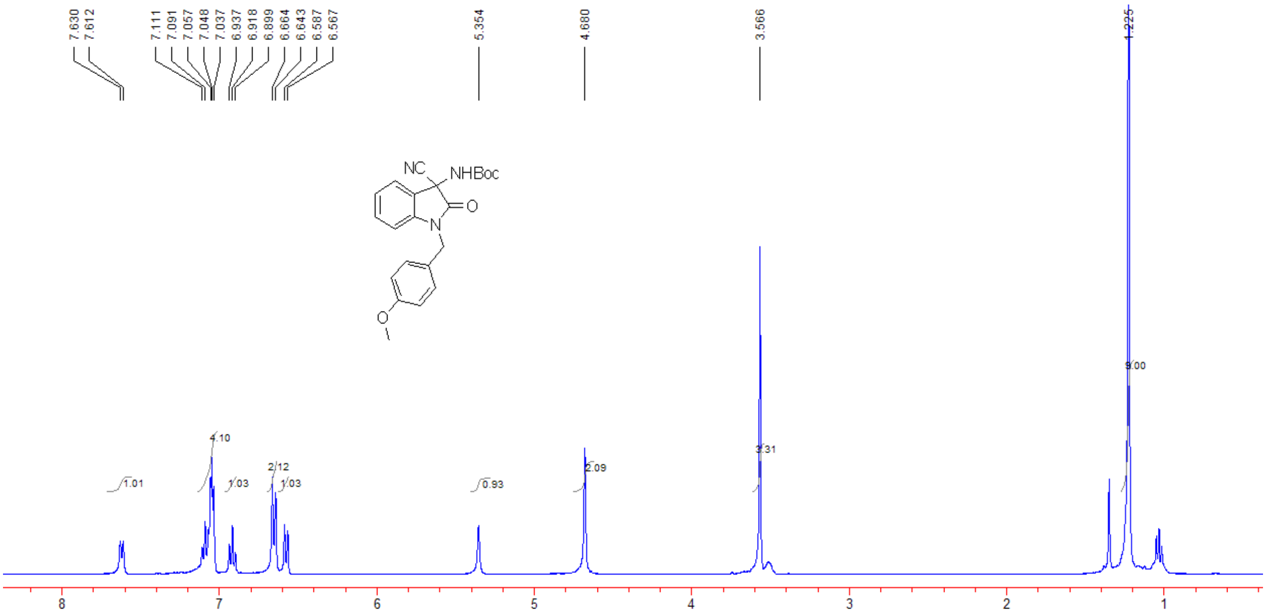

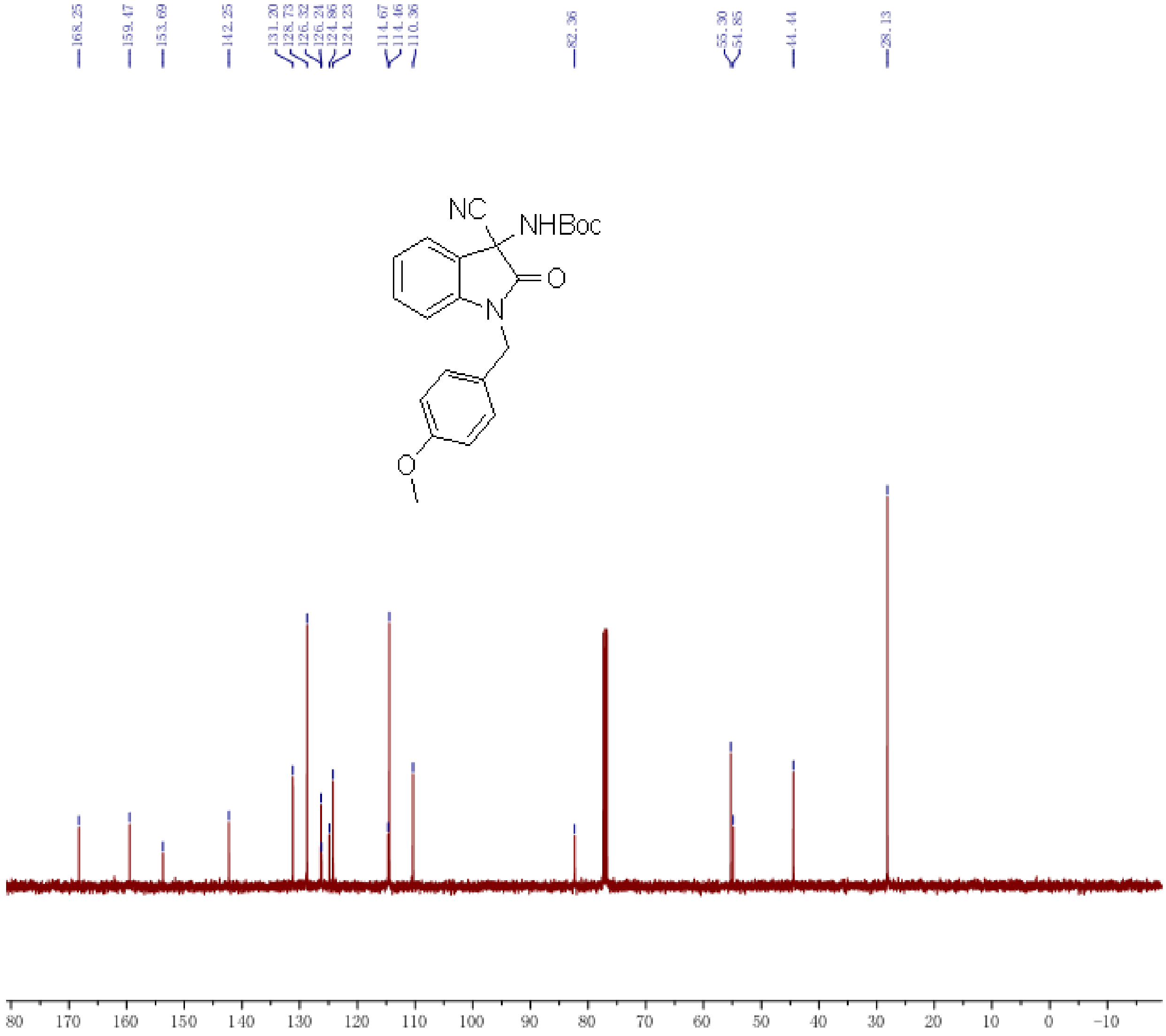

Supplementary Figure 14. ^{1}H and ^{13}C-NMR spectra of product **2e**.

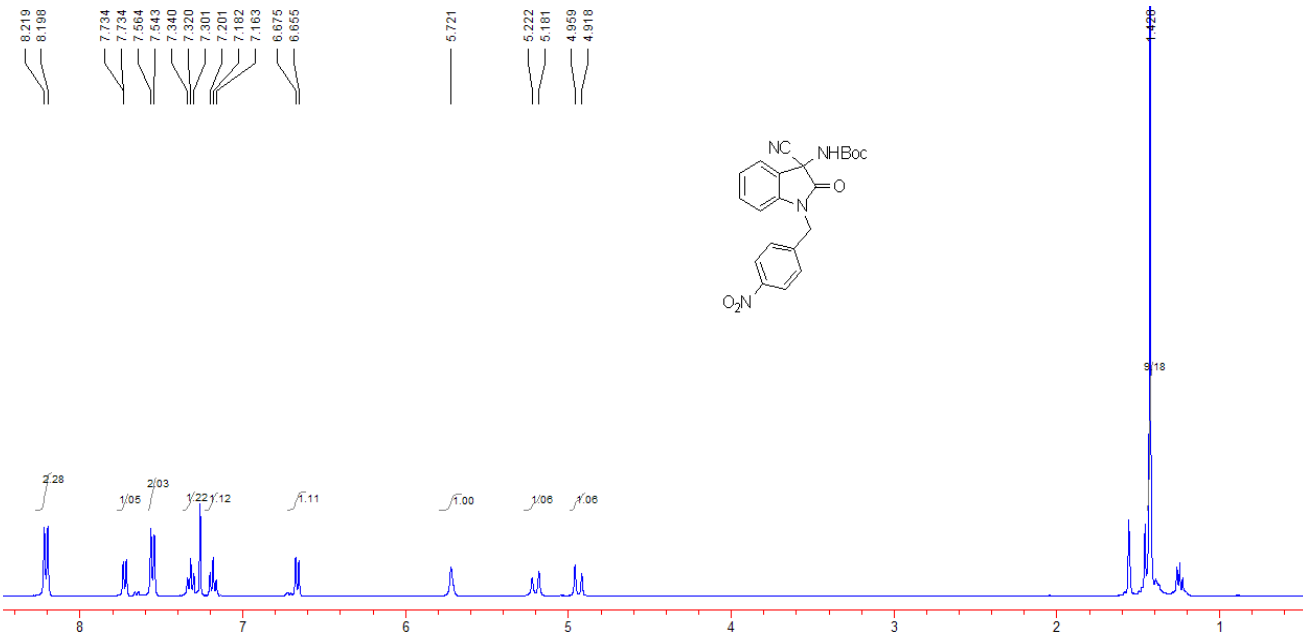

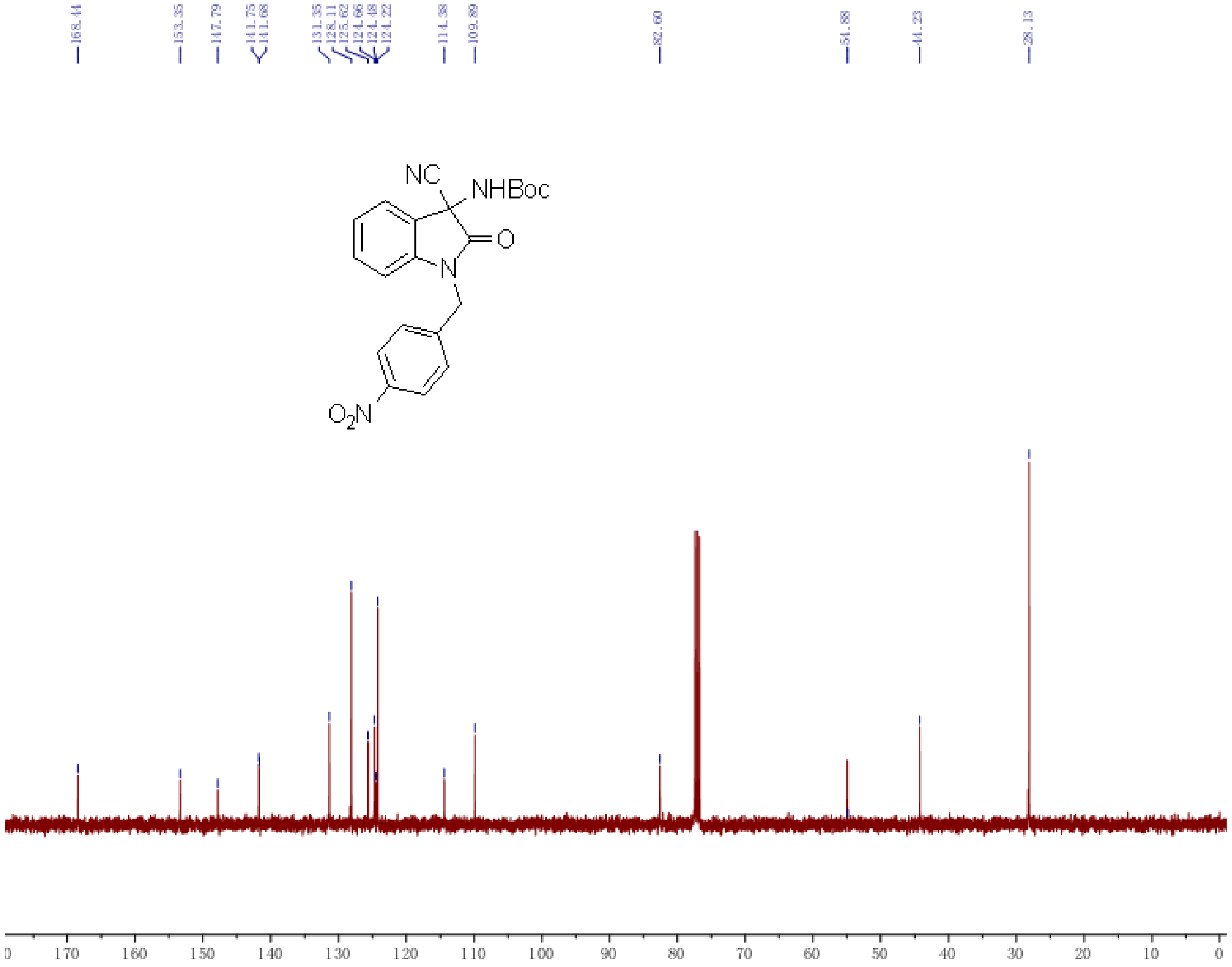

Supplementary Figure 15. ^{1}H and ^{13}C-NMR spectra of product **2f**.

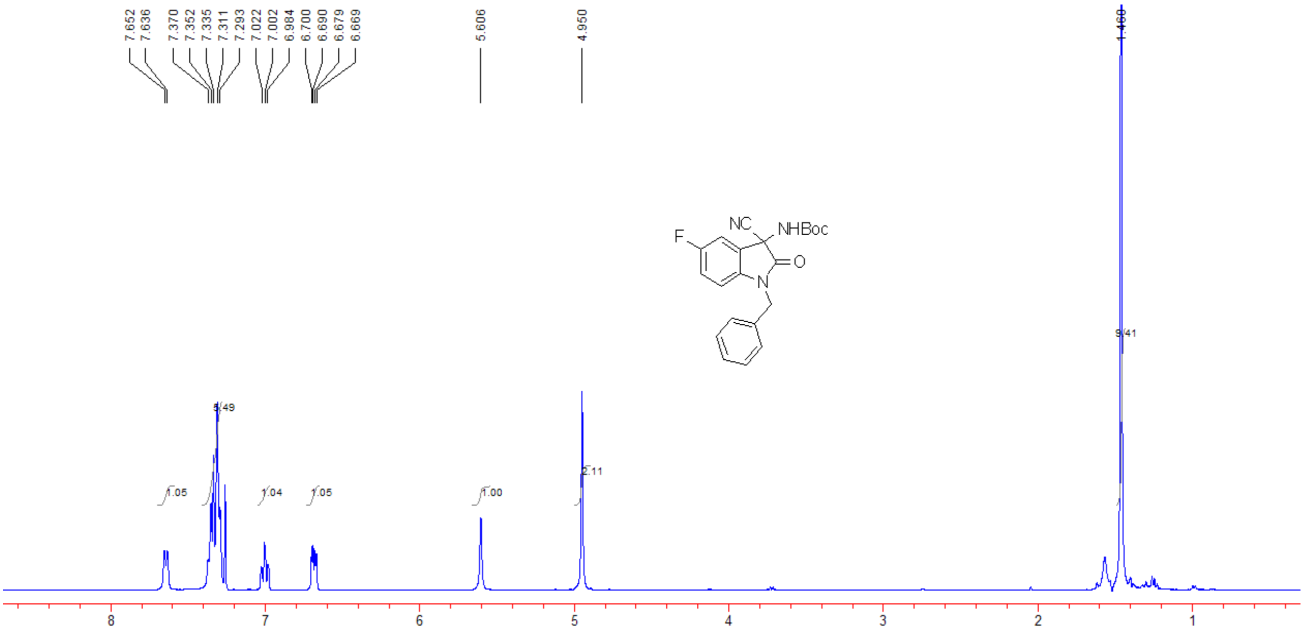

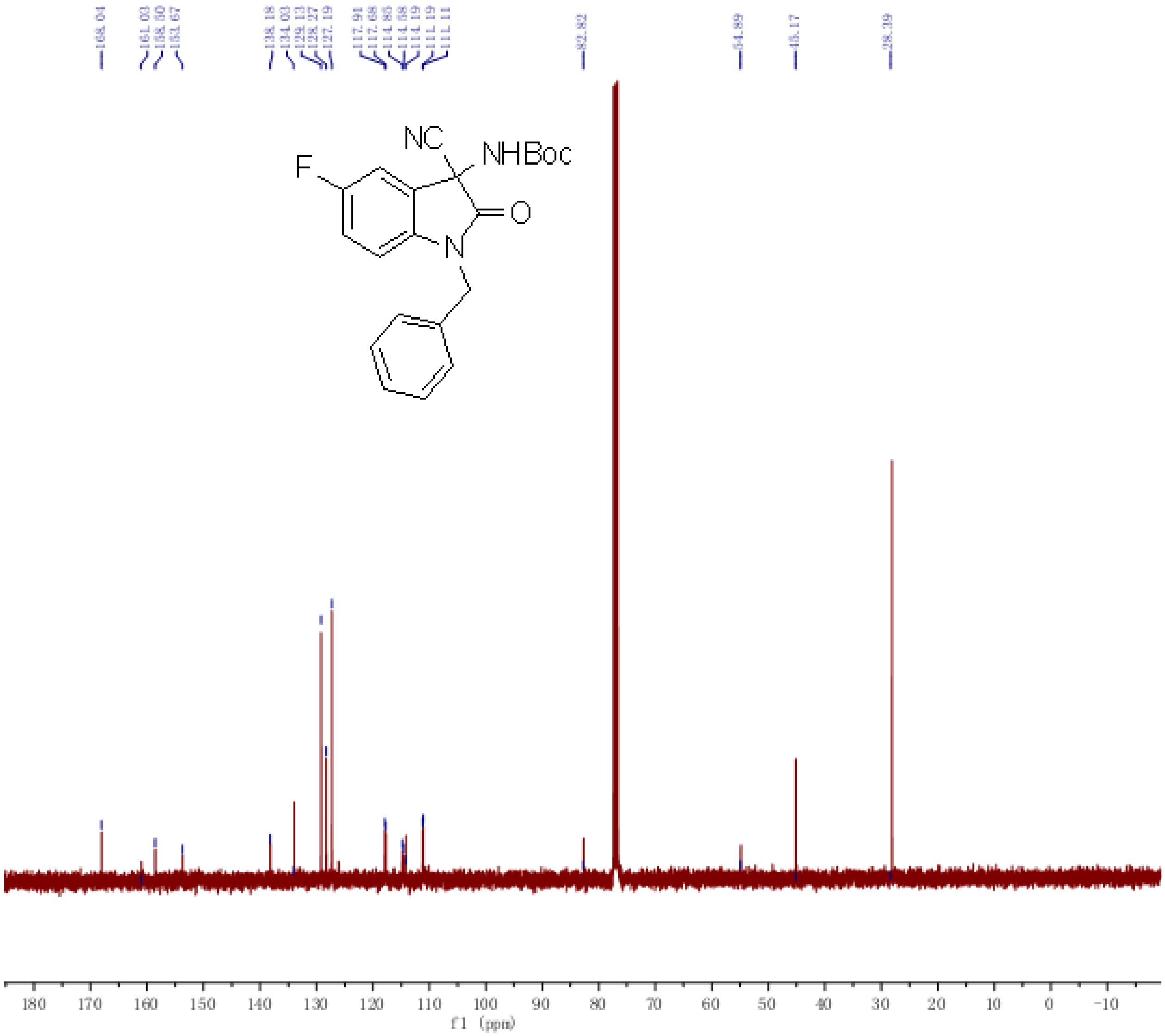

Supplementary Figure 16. ^{1}H and ^{13}C-NMR spectra of product **2g**.

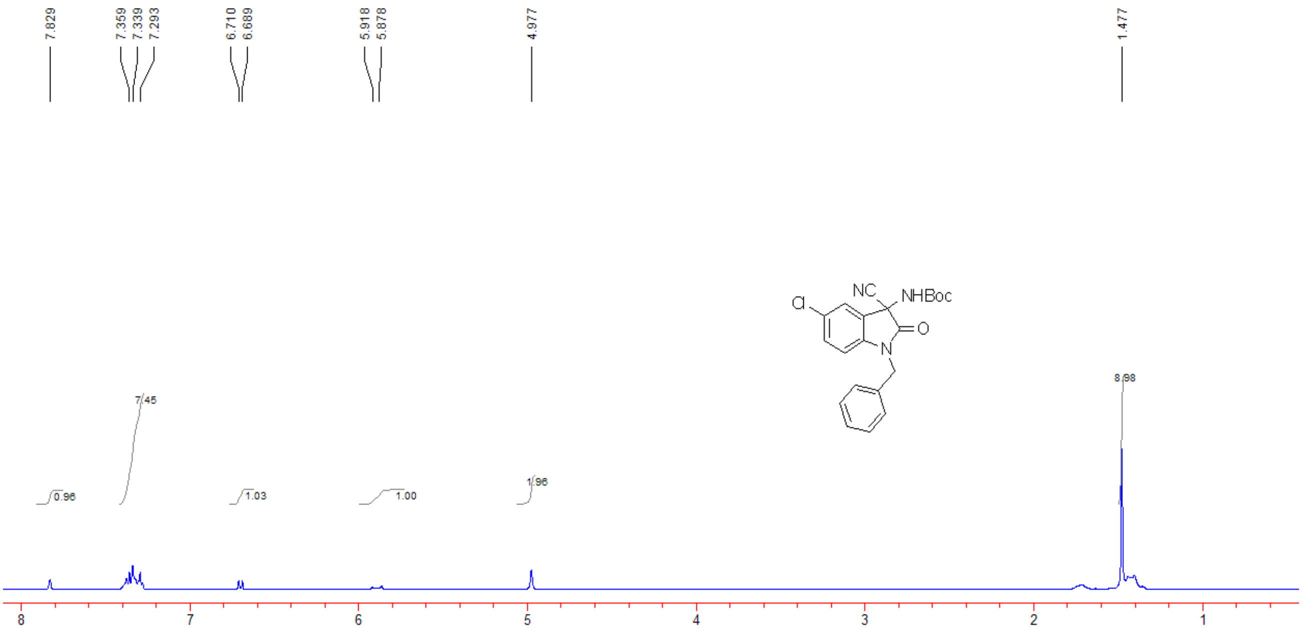

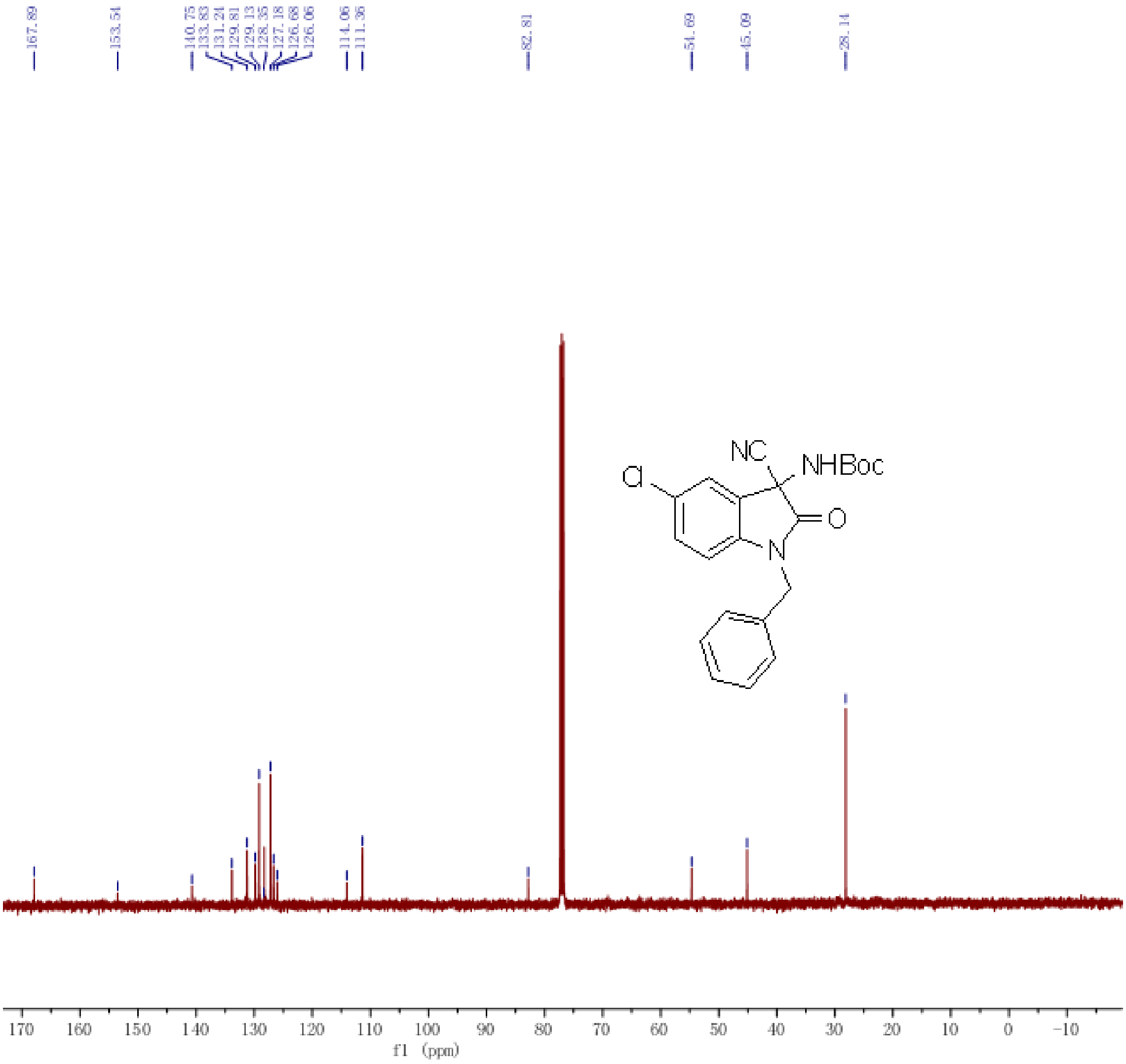

Supplementary Figure 17. ^{1}H and ^{13}C-NMR spectra of product **2h**.

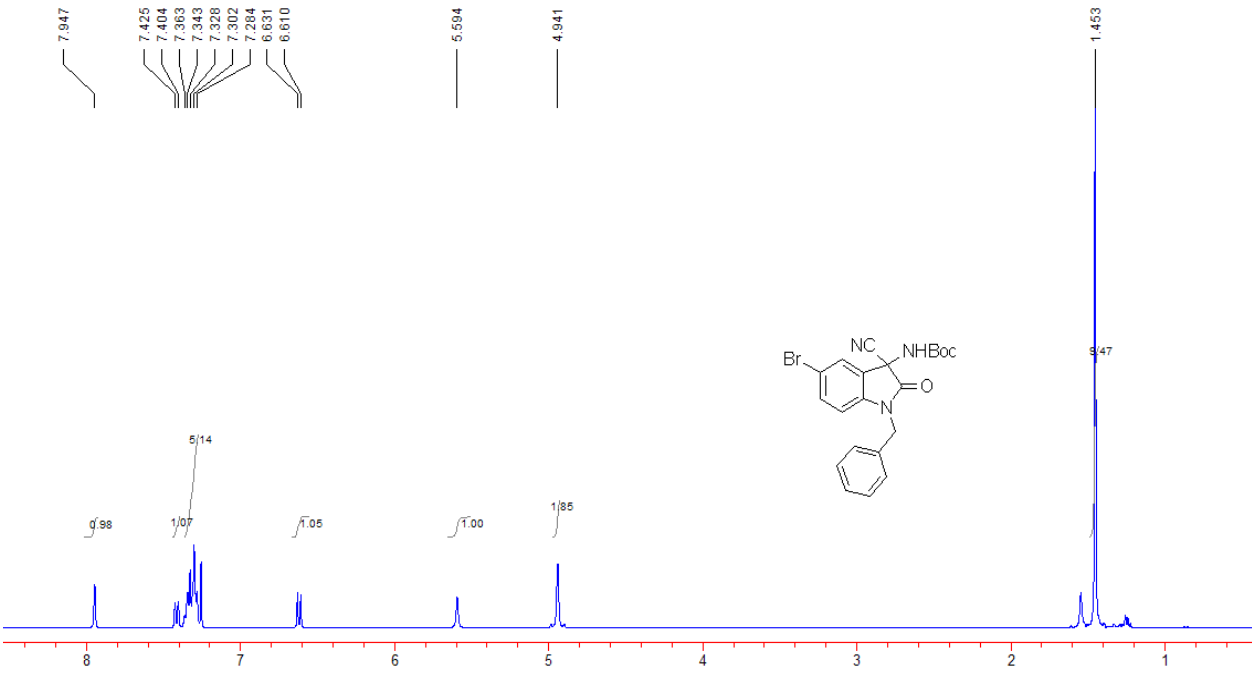

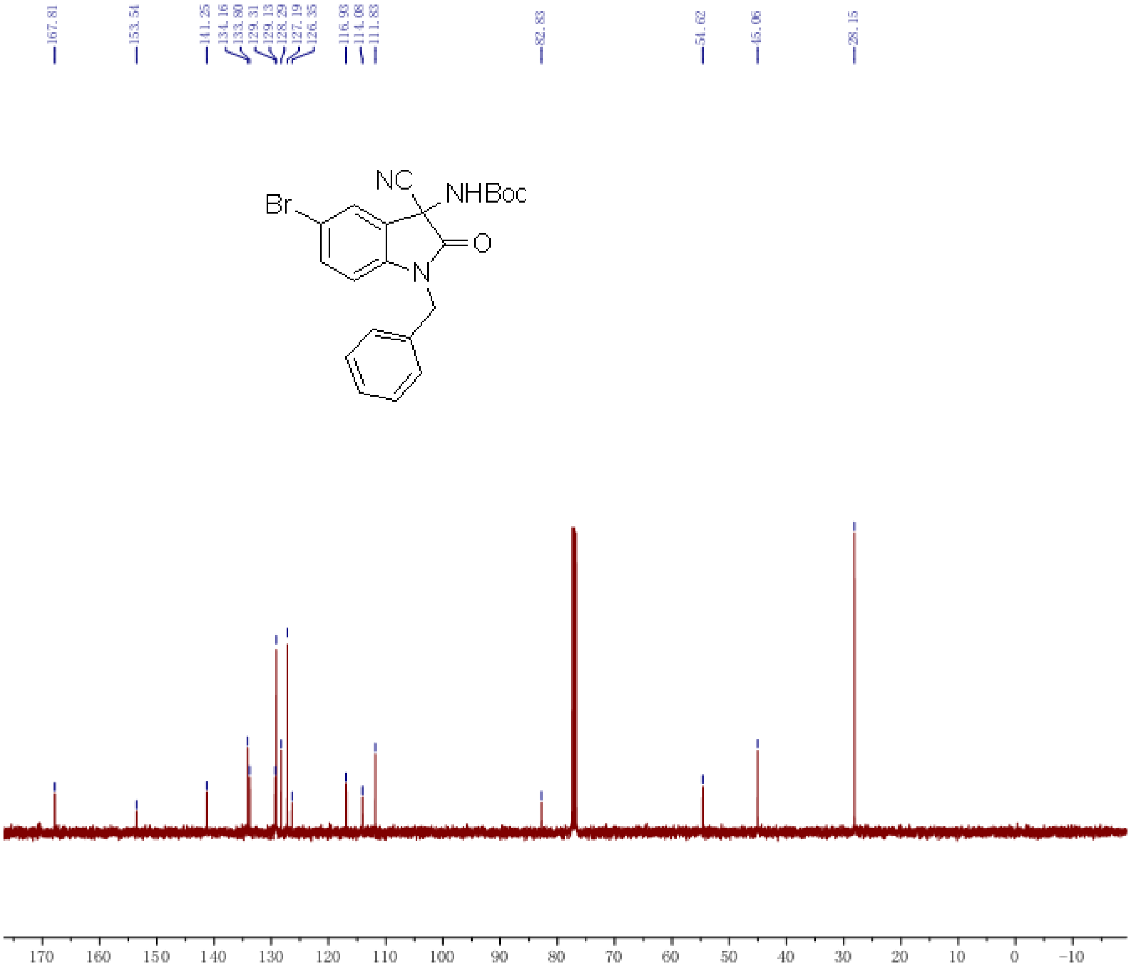

Supplementary Figure 18. ^{1}H and ^{13}C-NMR spectra of product **2i**.

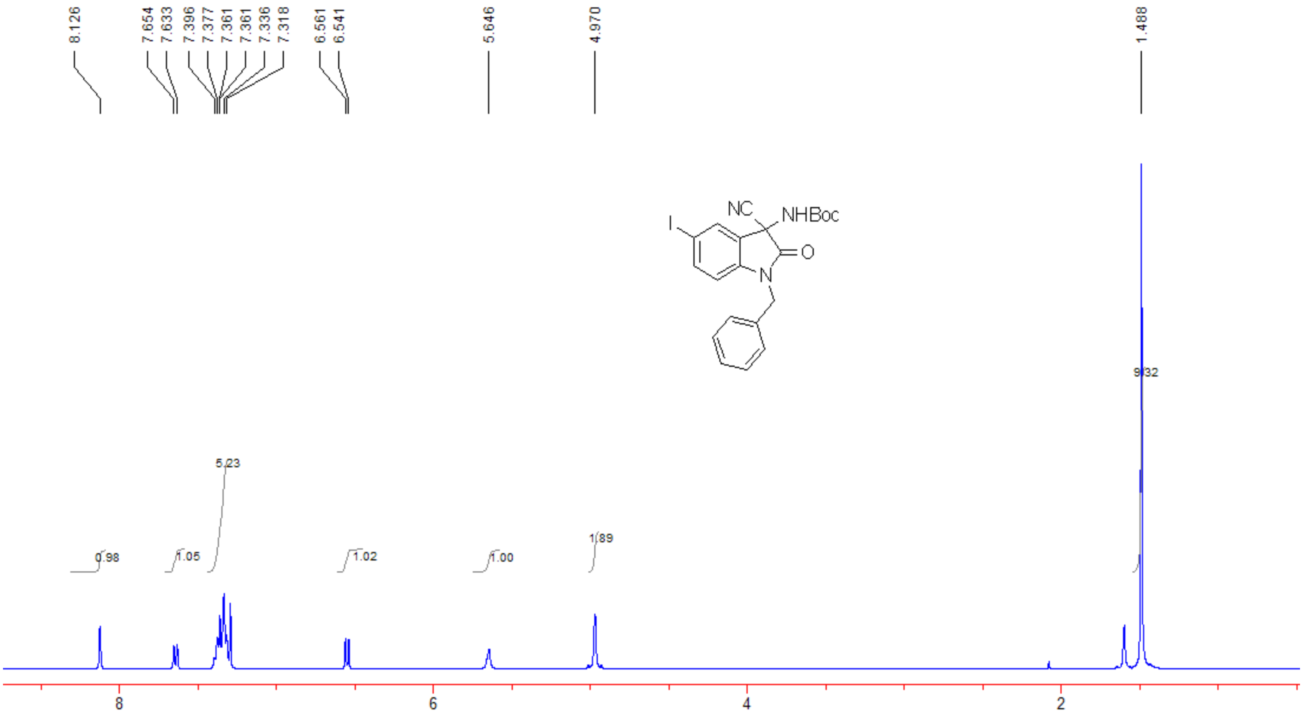

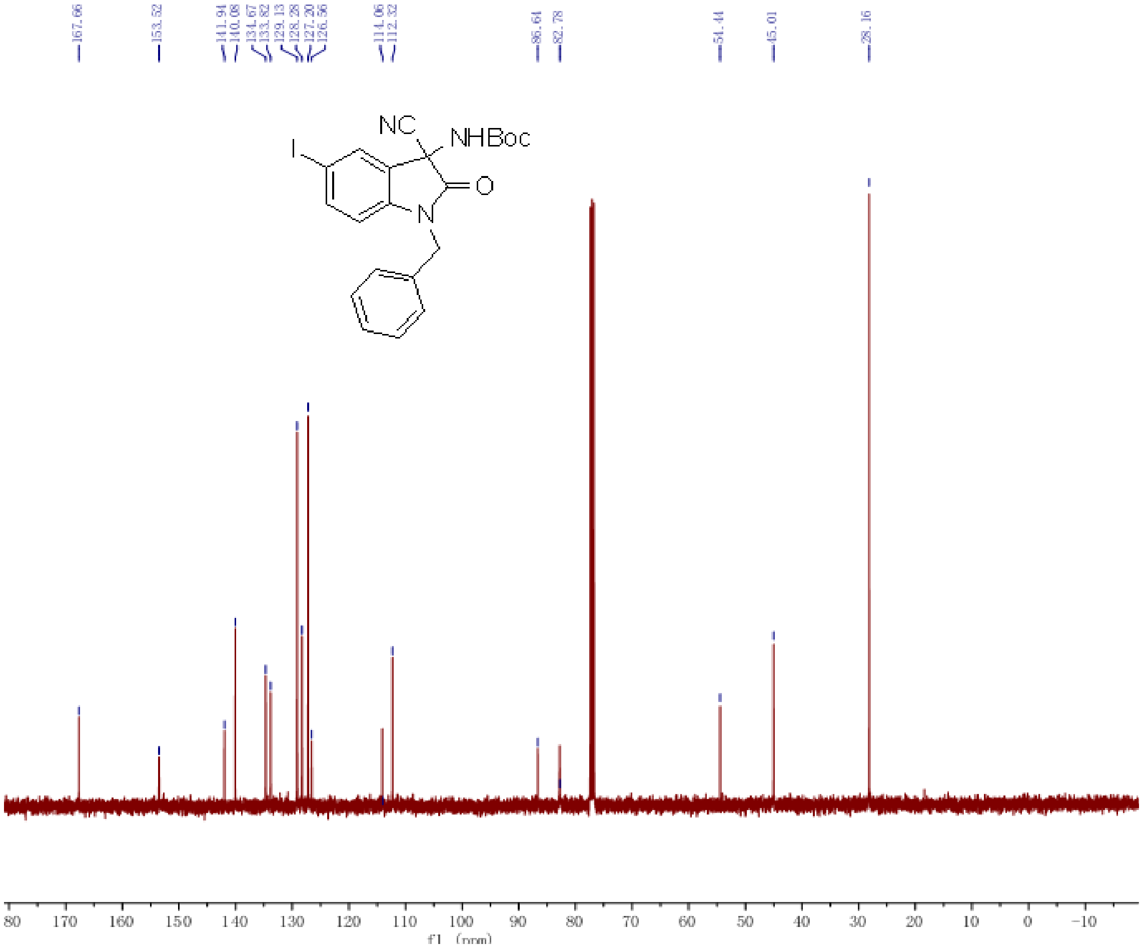

Supplementary Figure 19. ^{1}H and ^{13}C-NMR spectra of product **2j**.

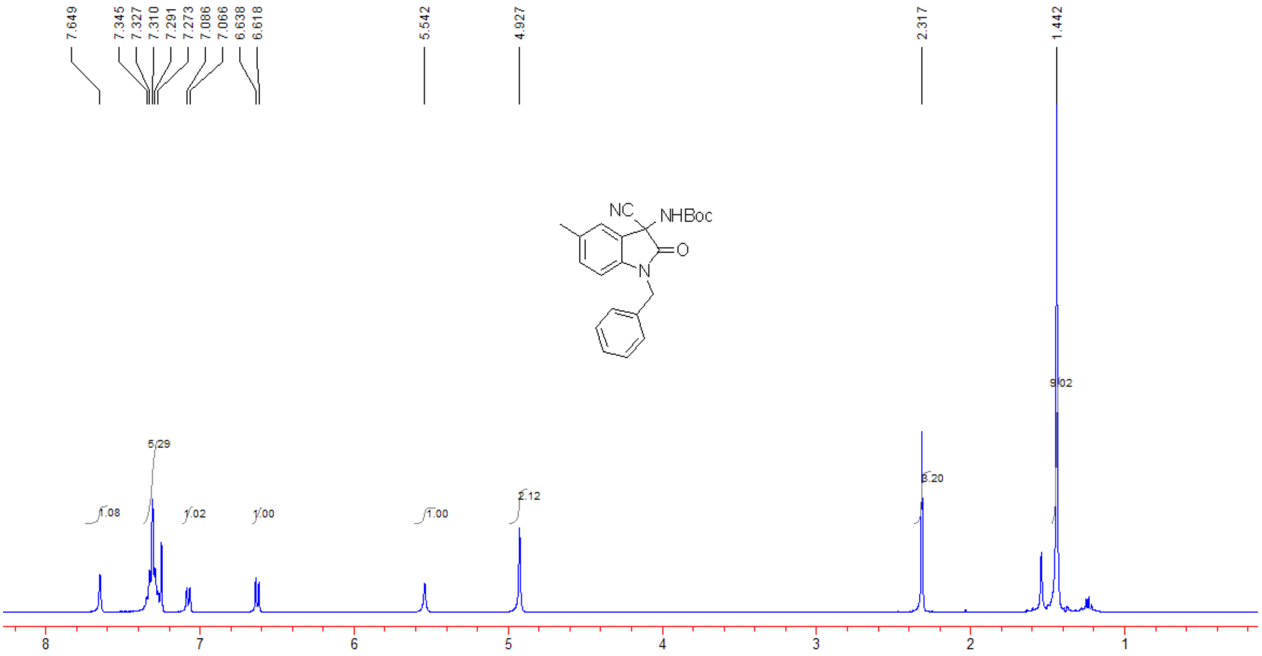

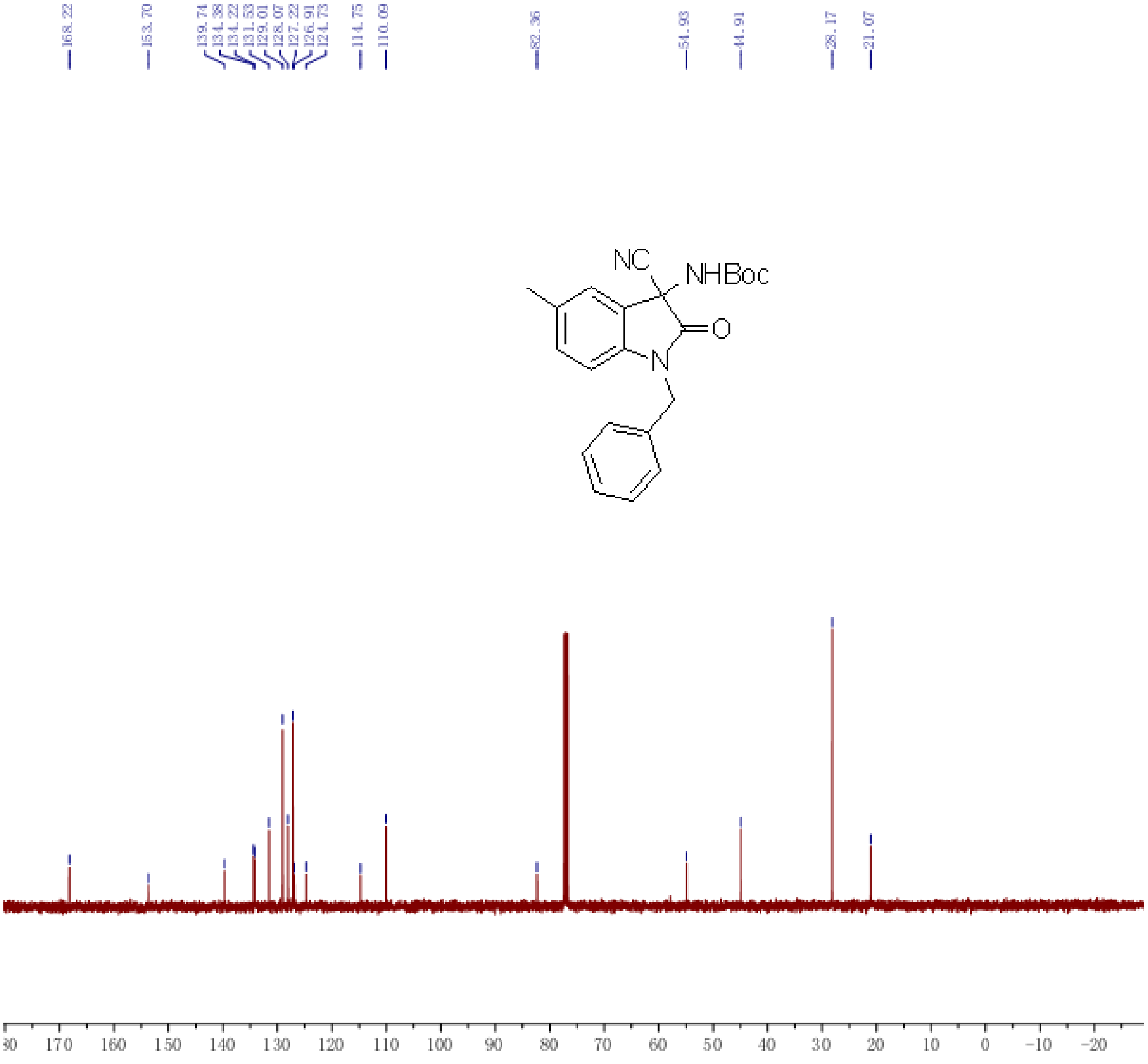

Supplementary Figure 20. ^{1}H and ^{13}C-NMR spectra of product **2k**.

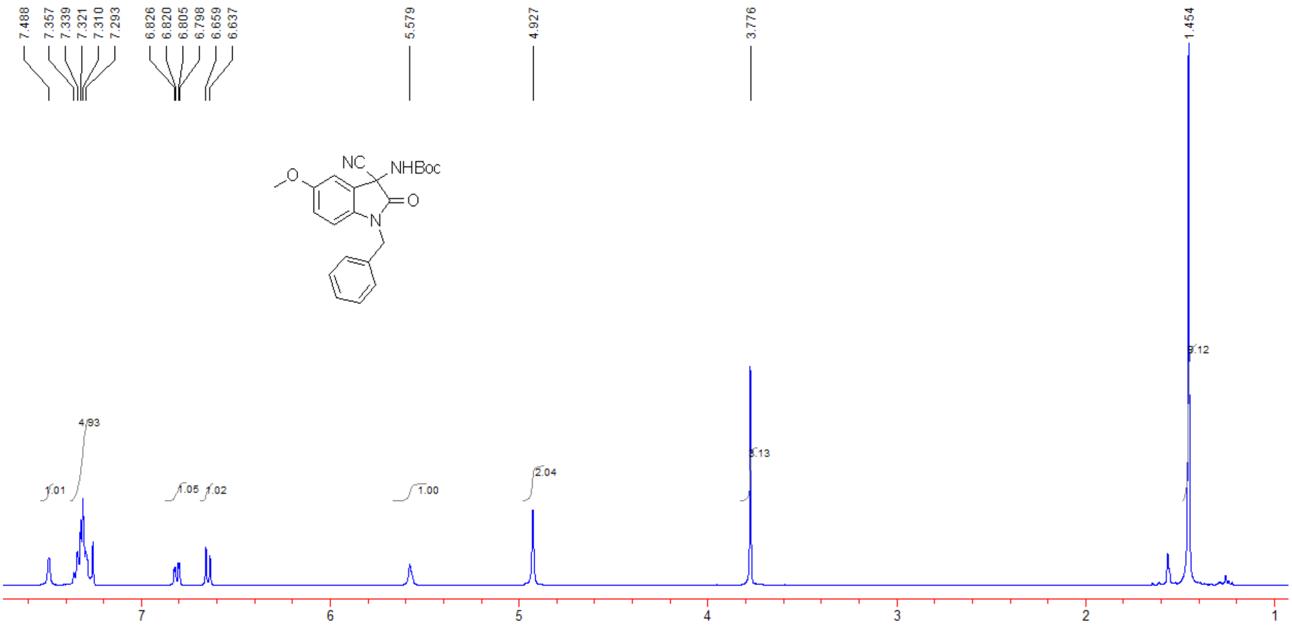

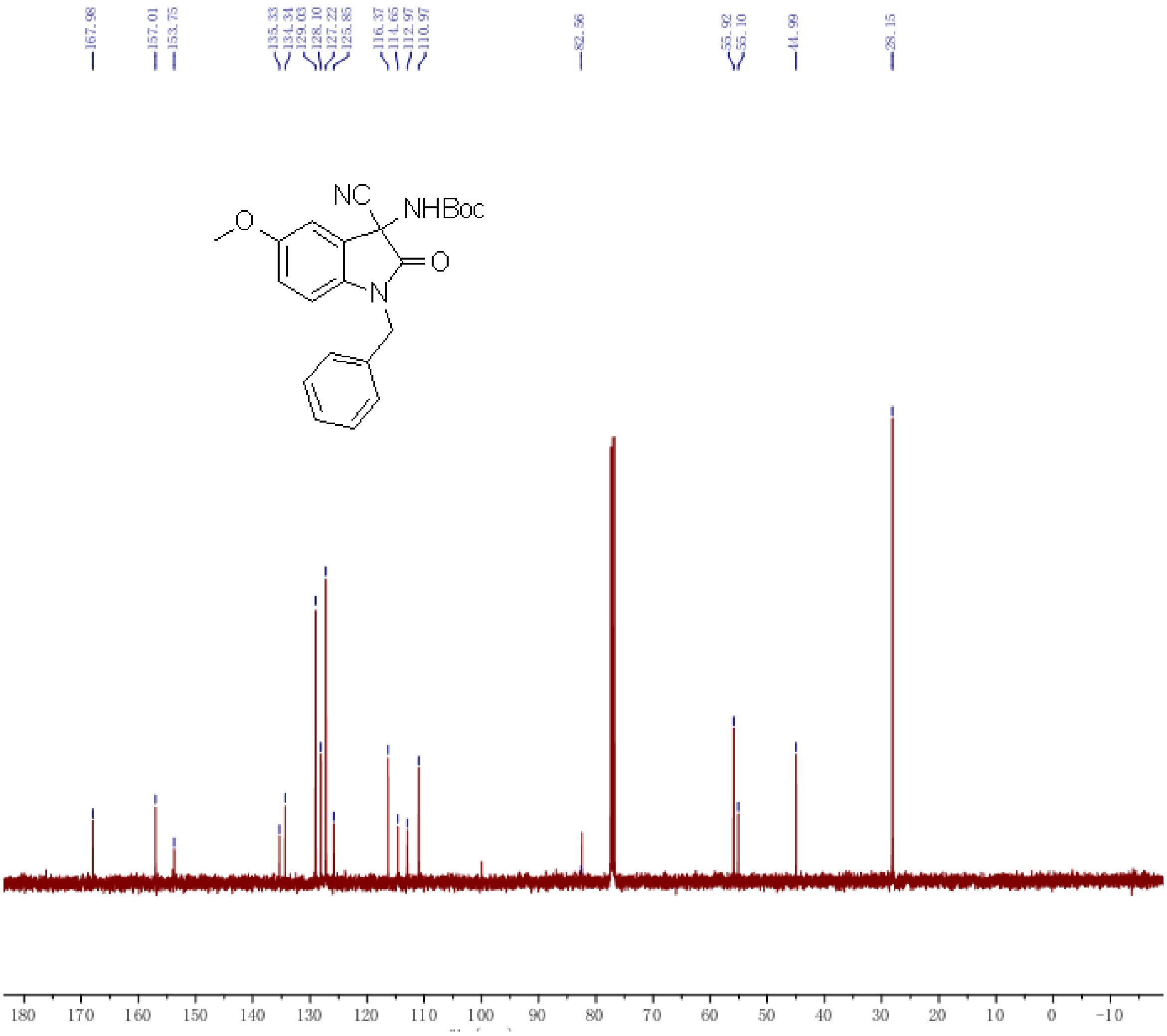

Supplementary Figure 21. ^{1}H and ^{13}C-NMR spectra of product **2l**.

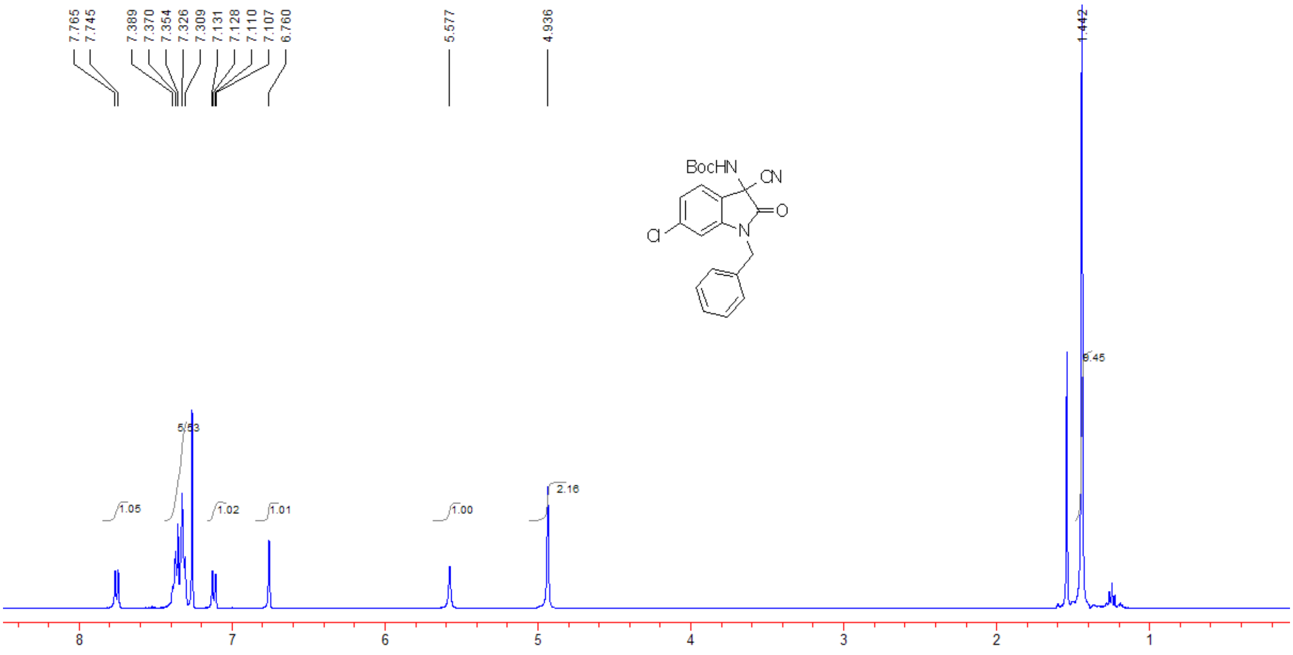

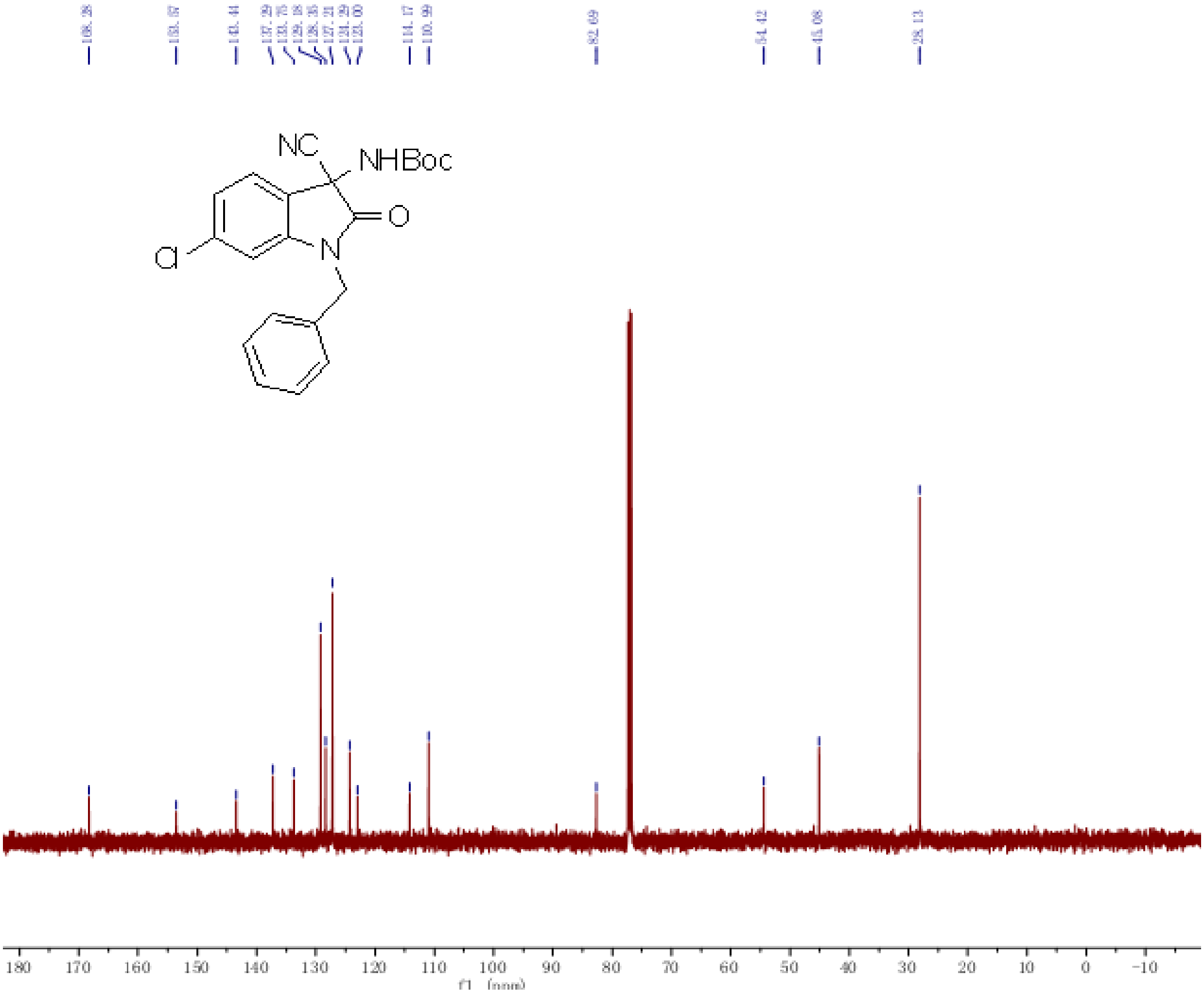

Supplementary Figure 22. ^{1}H and ^{13}C-NMR spectra of product **2m**.

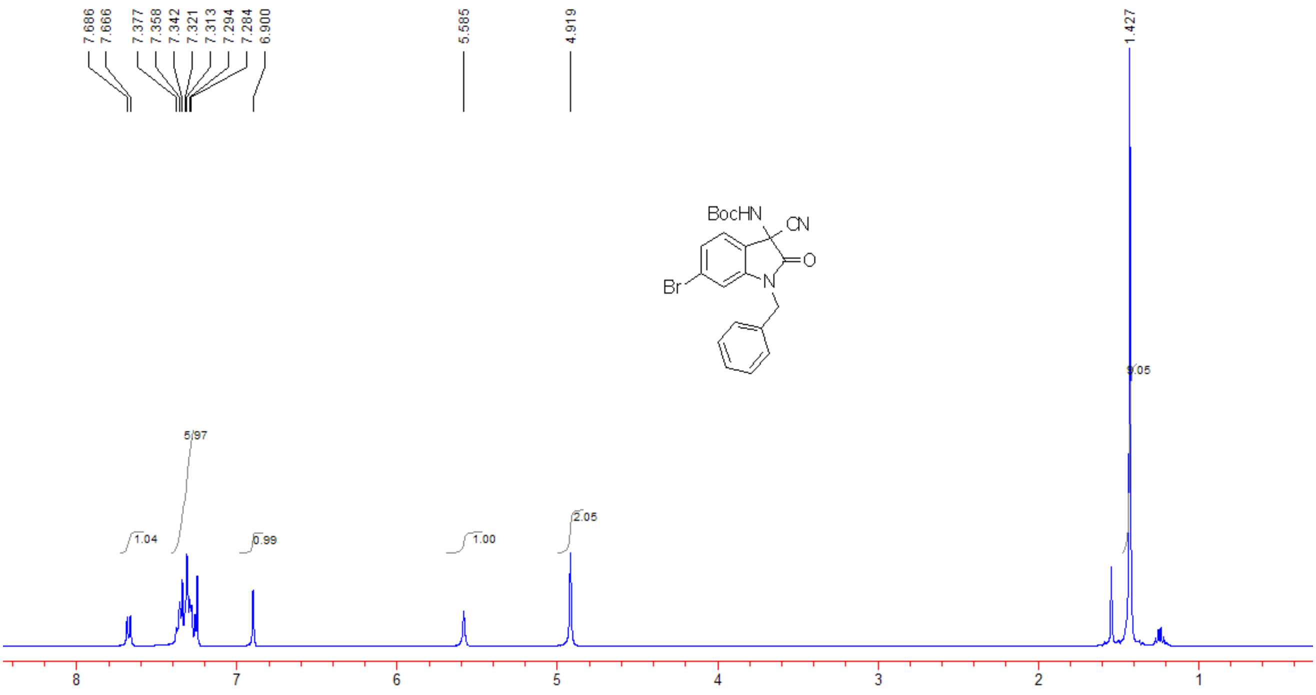

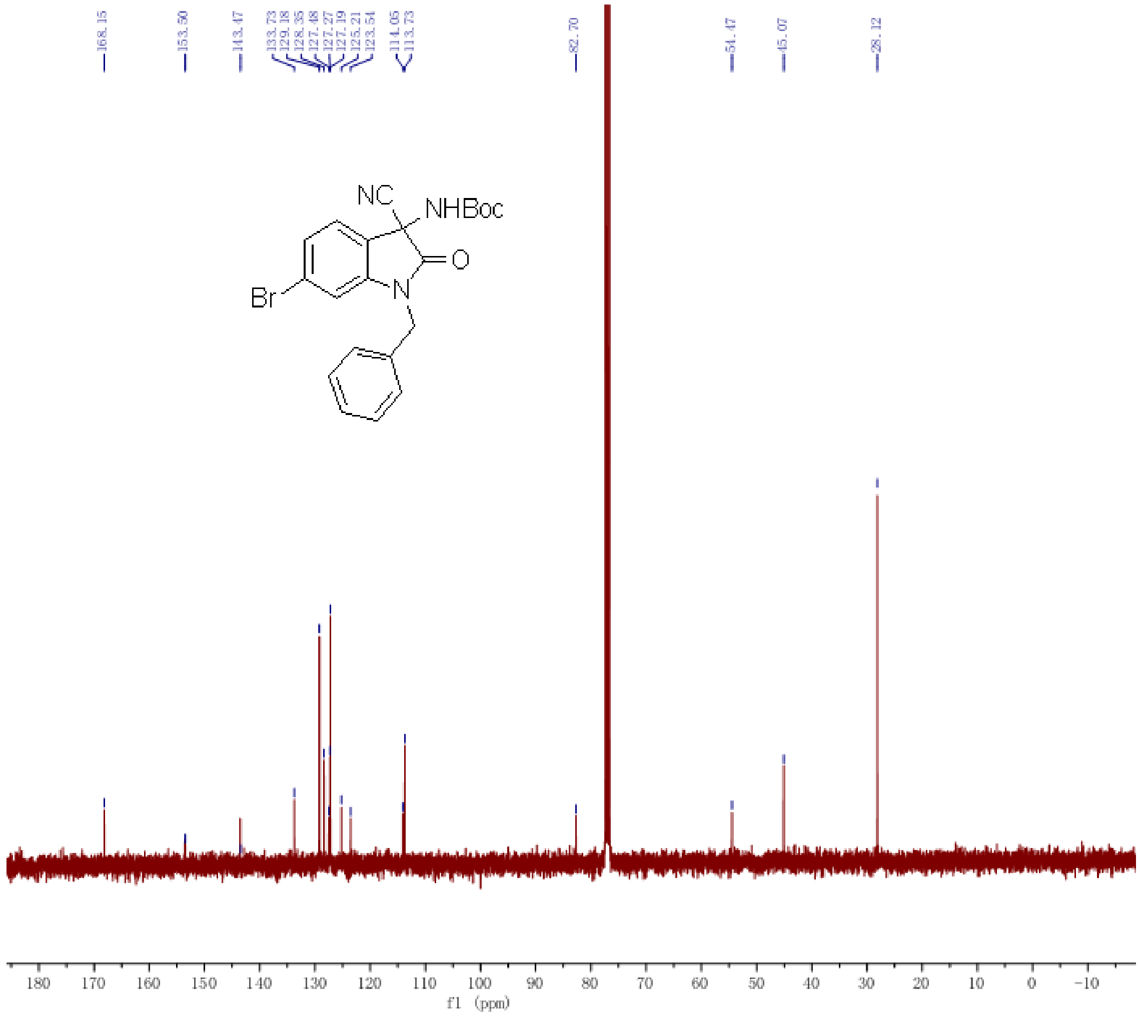

Supplementary Figure 23. ^{1}H and ^{13}C-NMR spectra of product **2n**.

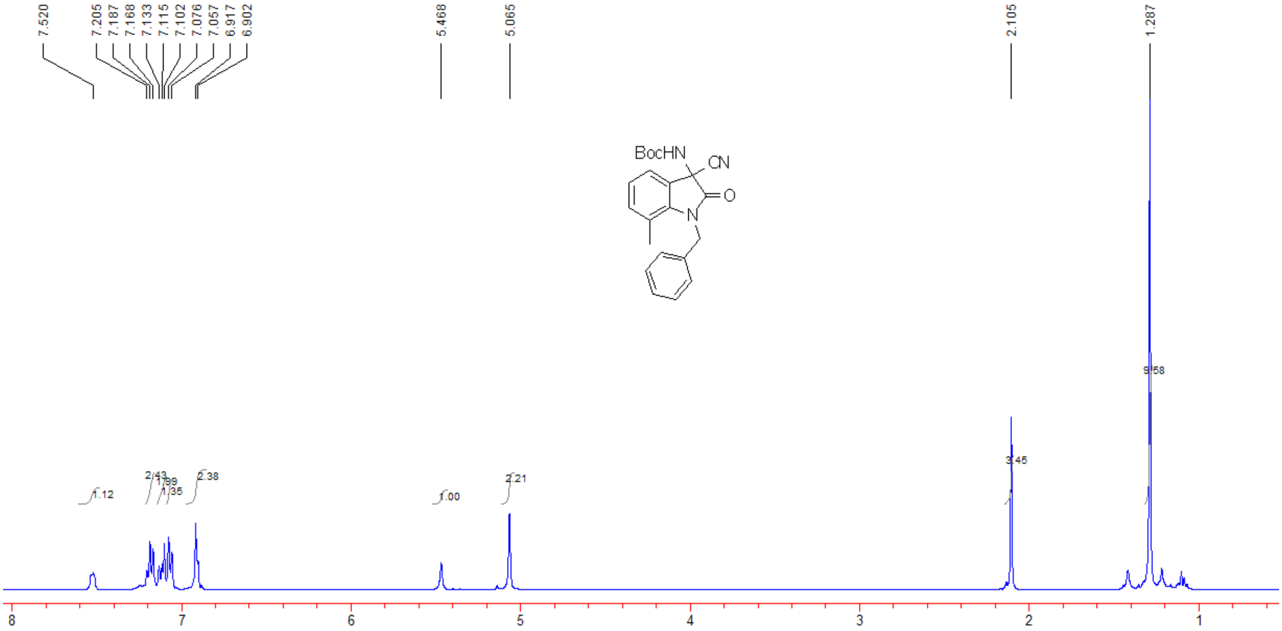

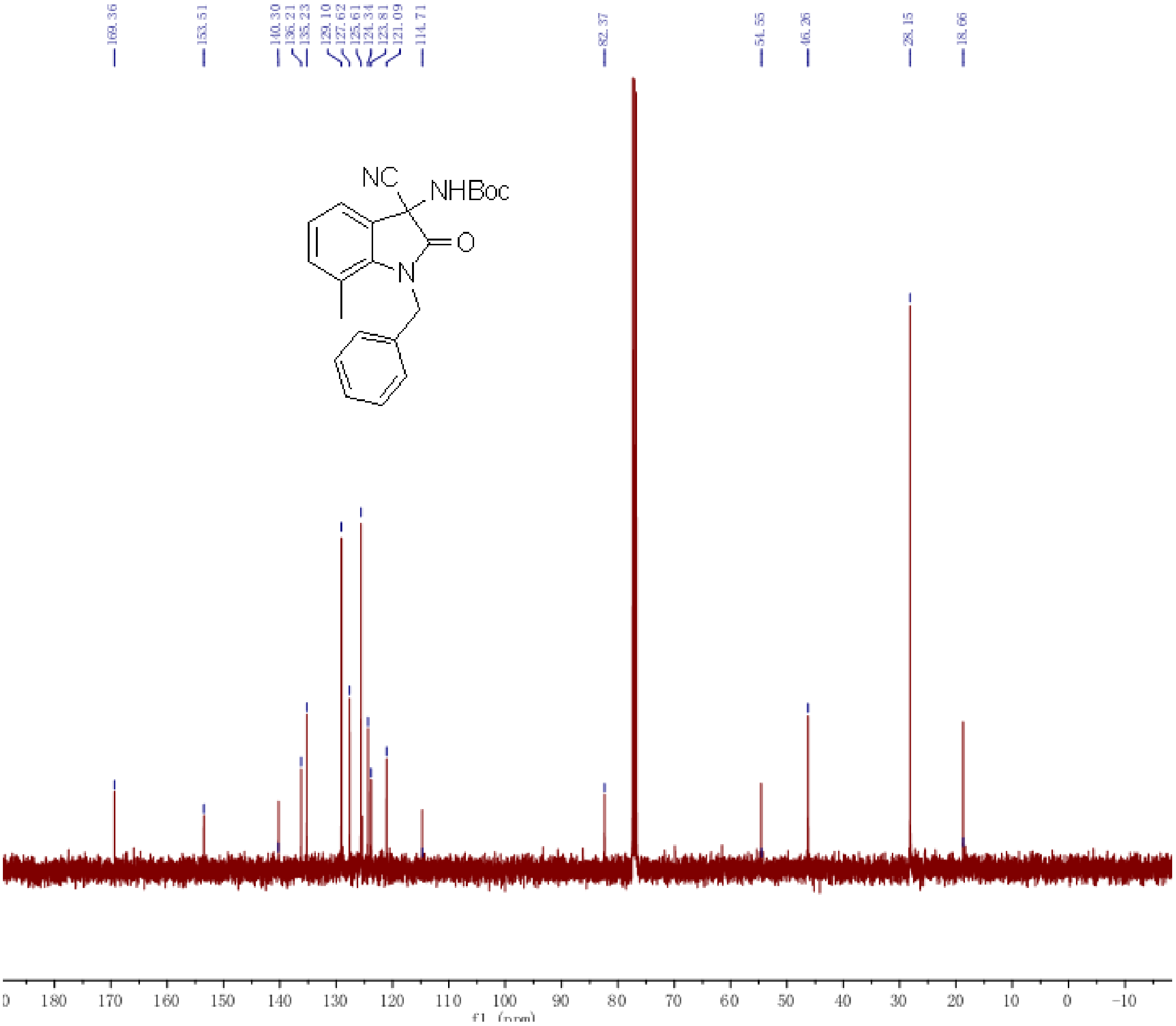

Supplementary Figure 24. ^{1}H and ^{13}C-NMR spectra of product **2p**.

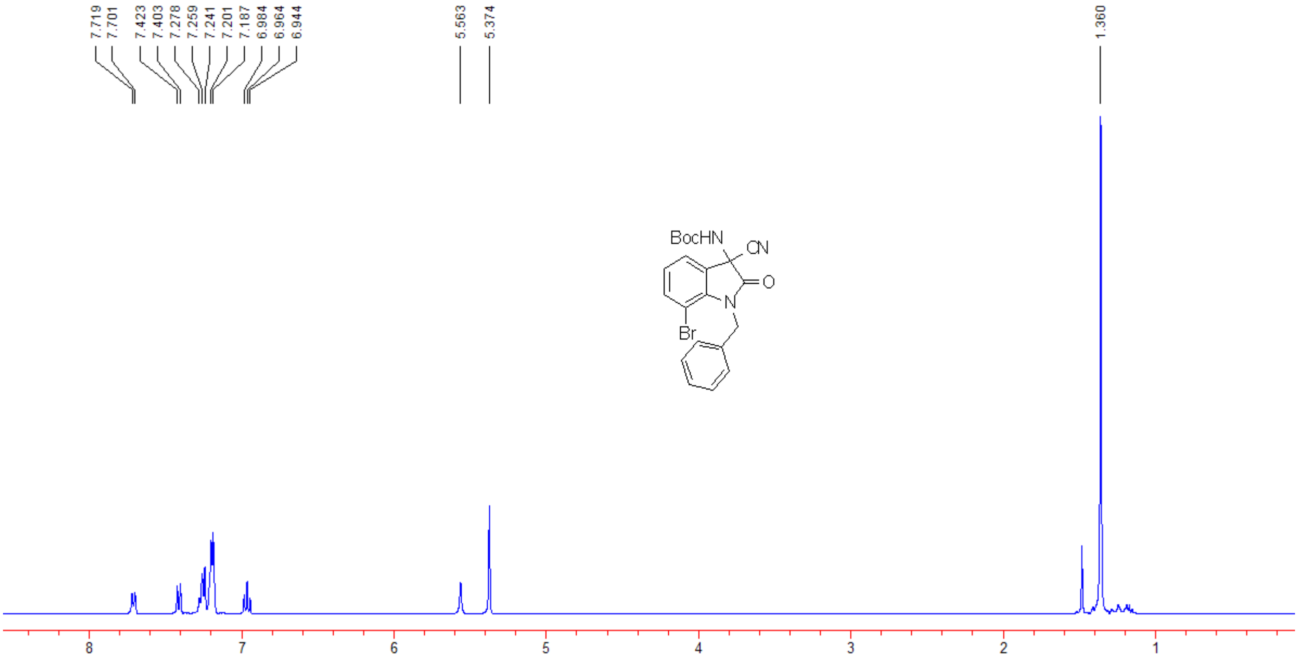

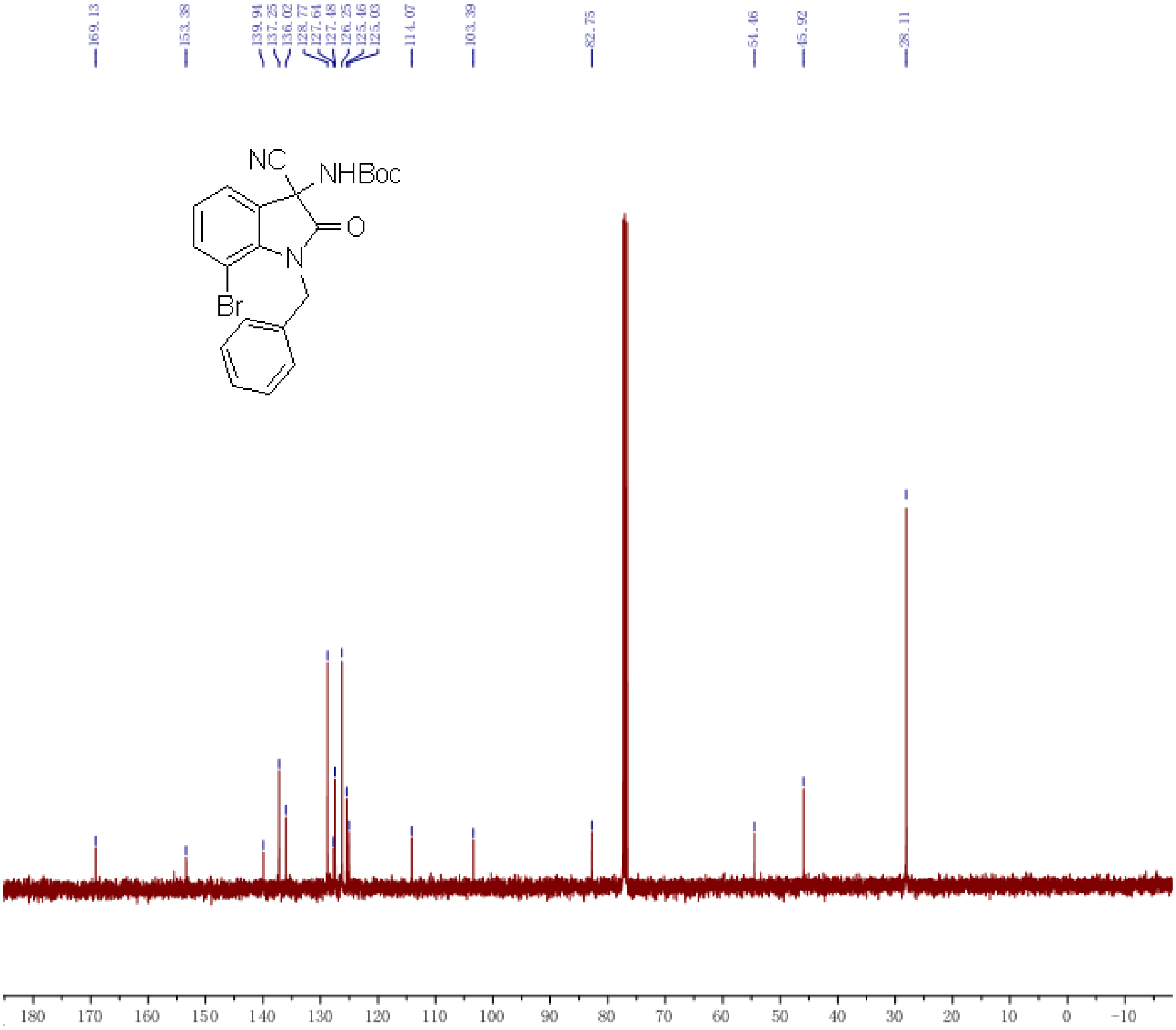

Supplementary Figure 25. ^{1}H and ^{13}C-NMR spectra of product **5k**.

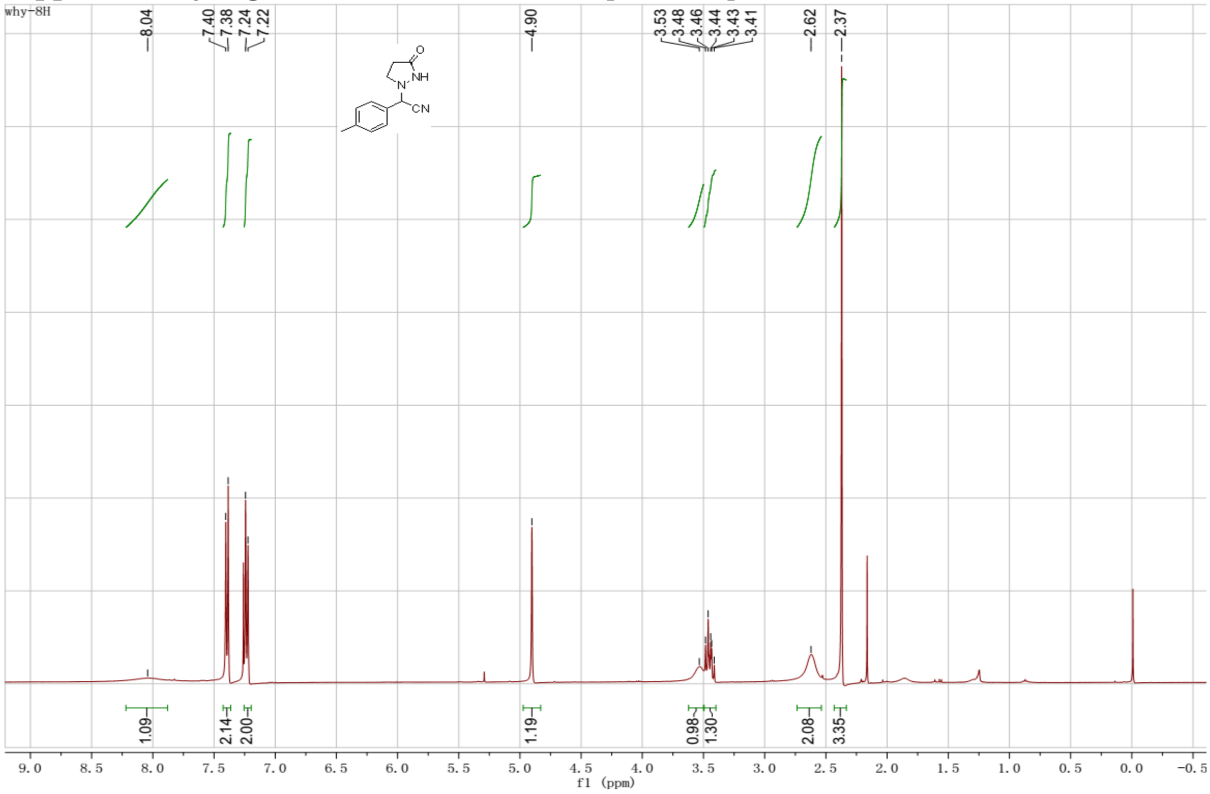

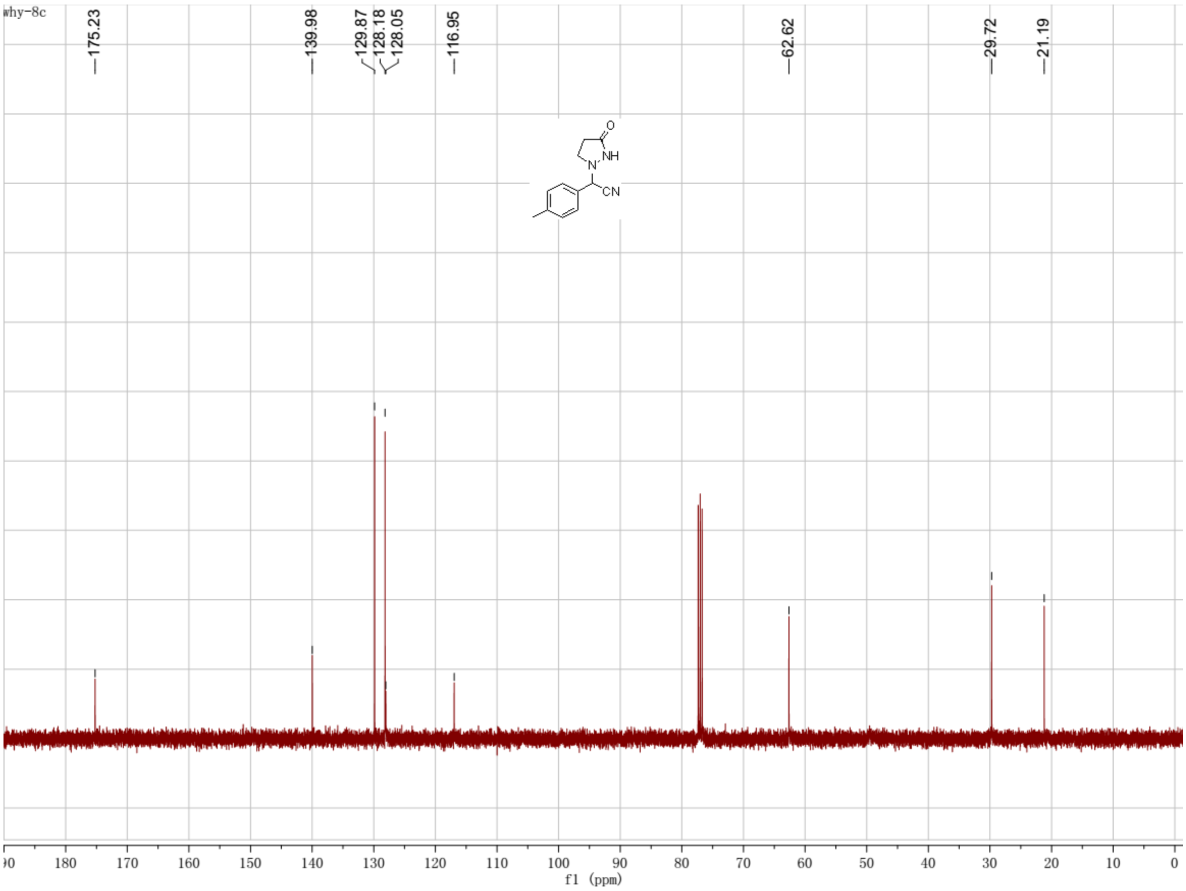

Supplementary Figure 26. ^{1}H and ^{13}C-NMR spectra of product **5l**.

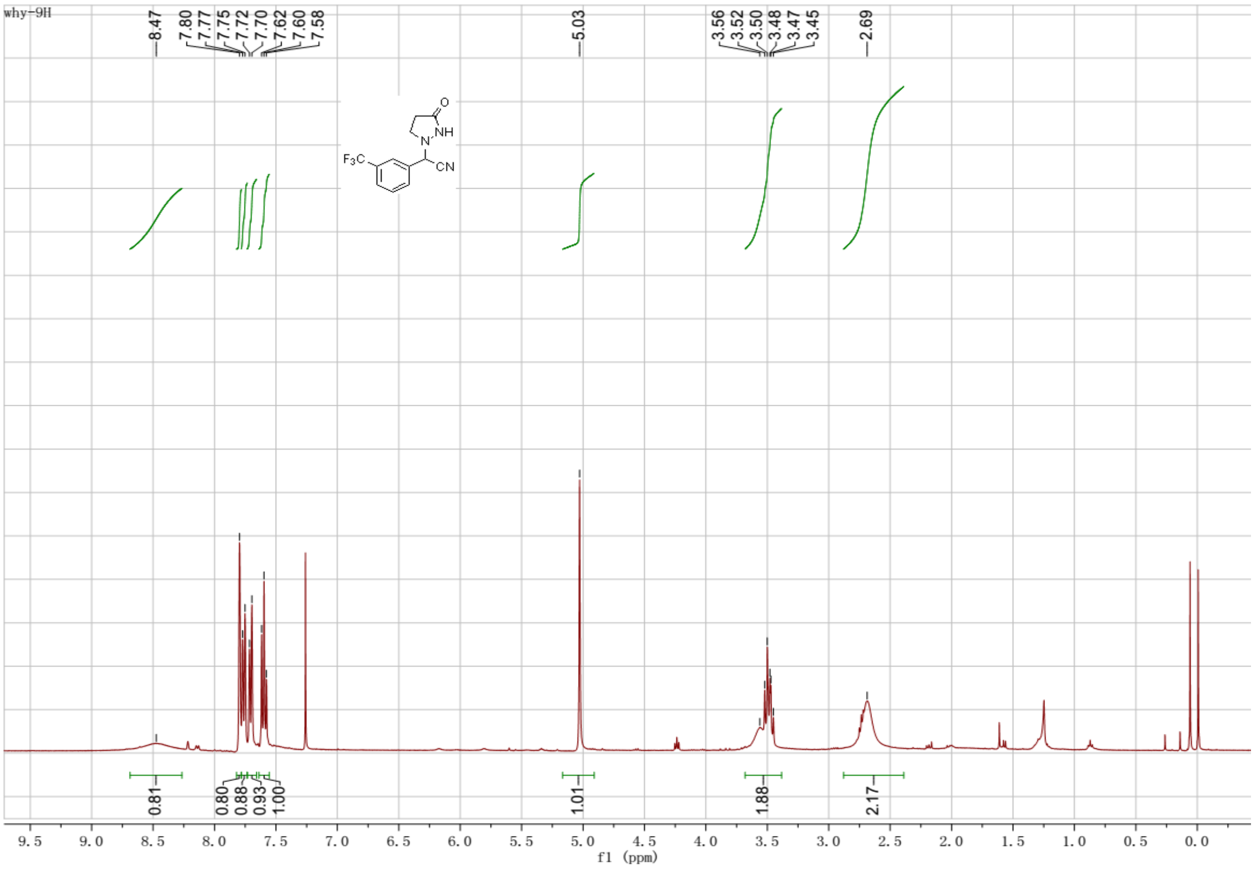

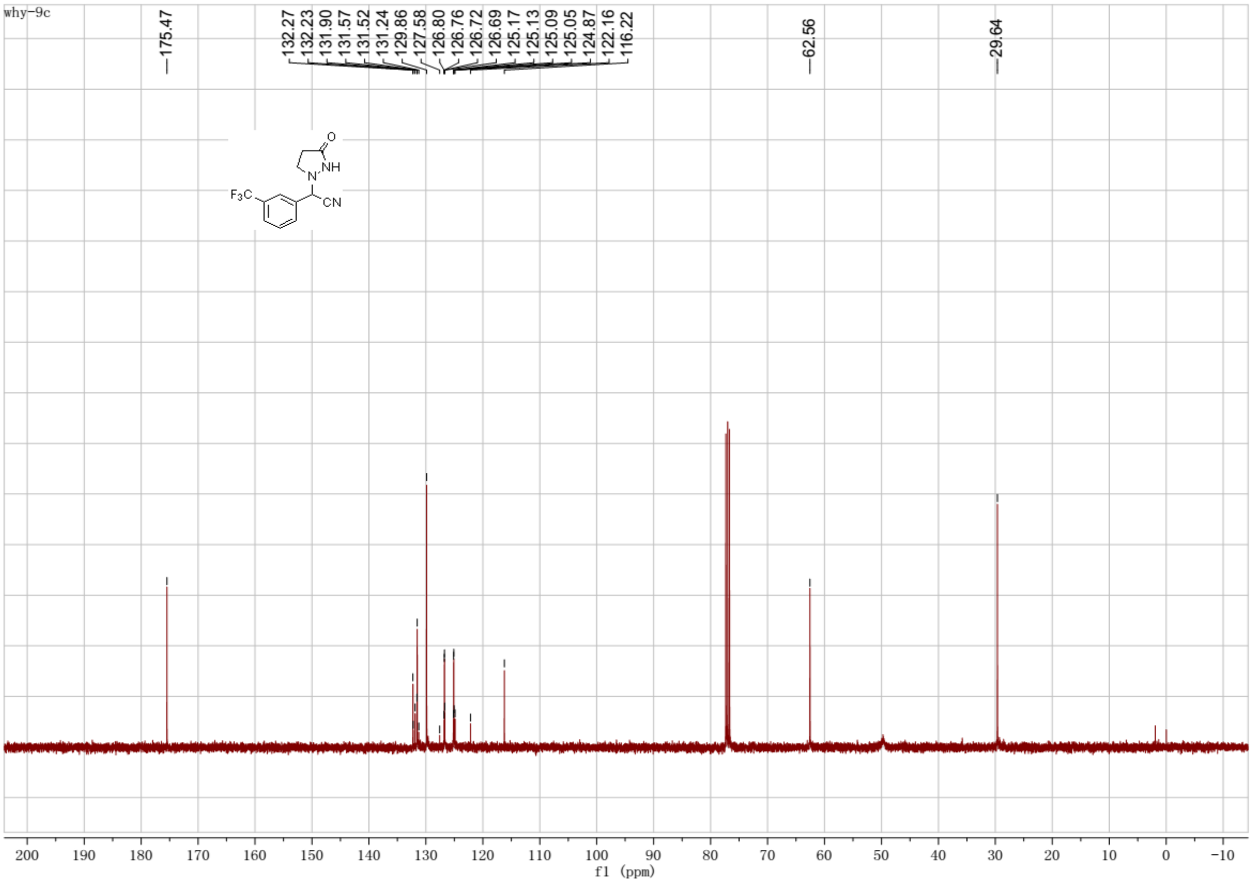

Supplementary Figure 27. ^{1}H and ^{13}C-NMR spectra of product **5m**.

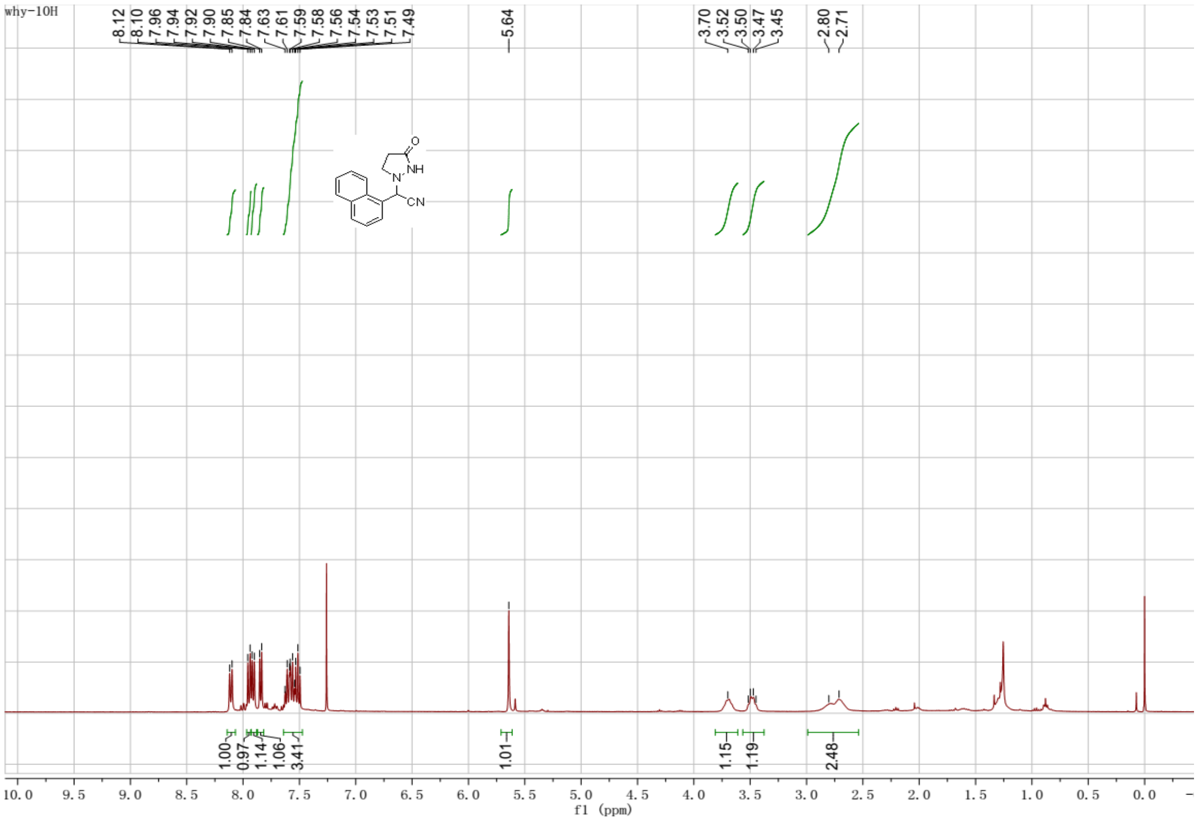

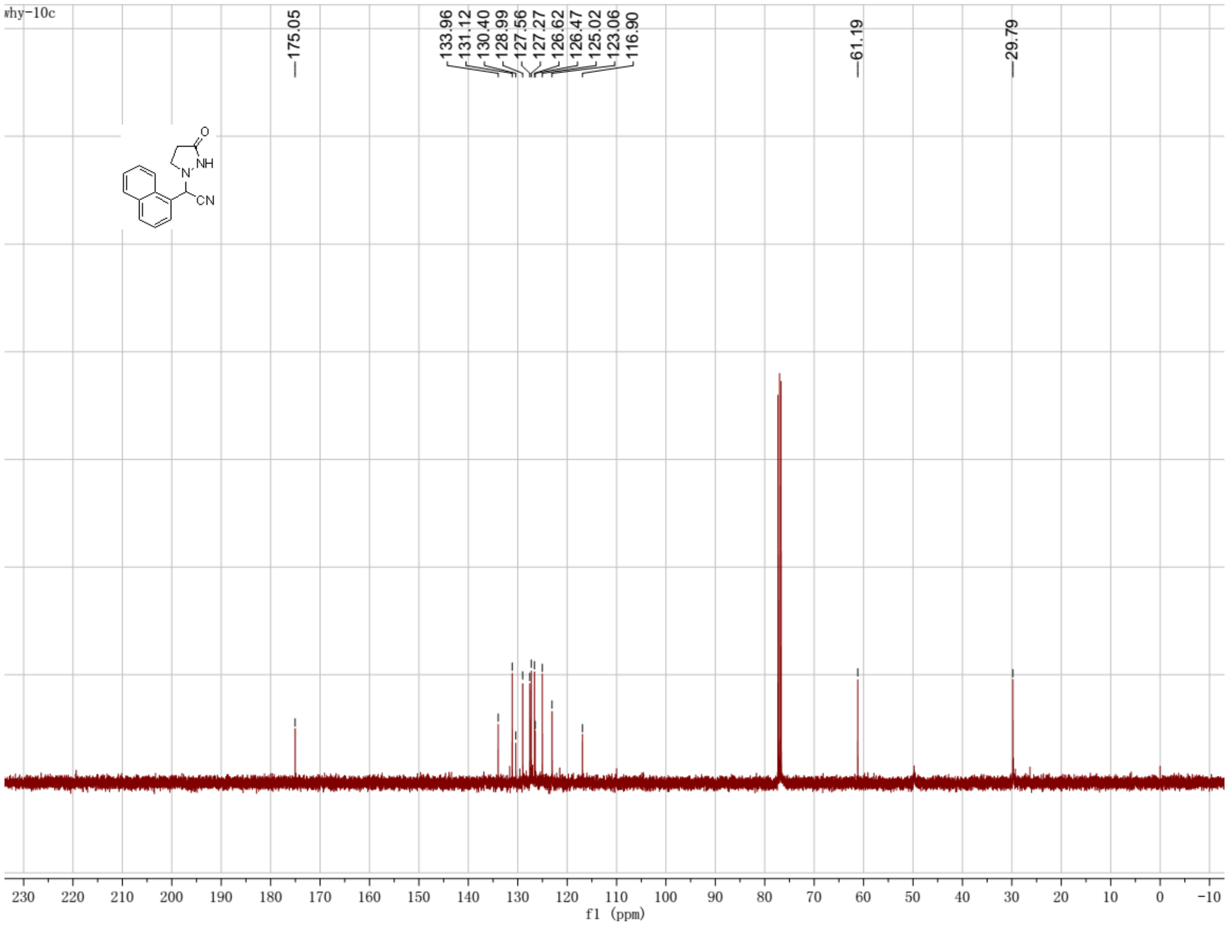

Supplementary Figure 28. ^{1}H and ^{13}C-NMR spectra of product **6b**.

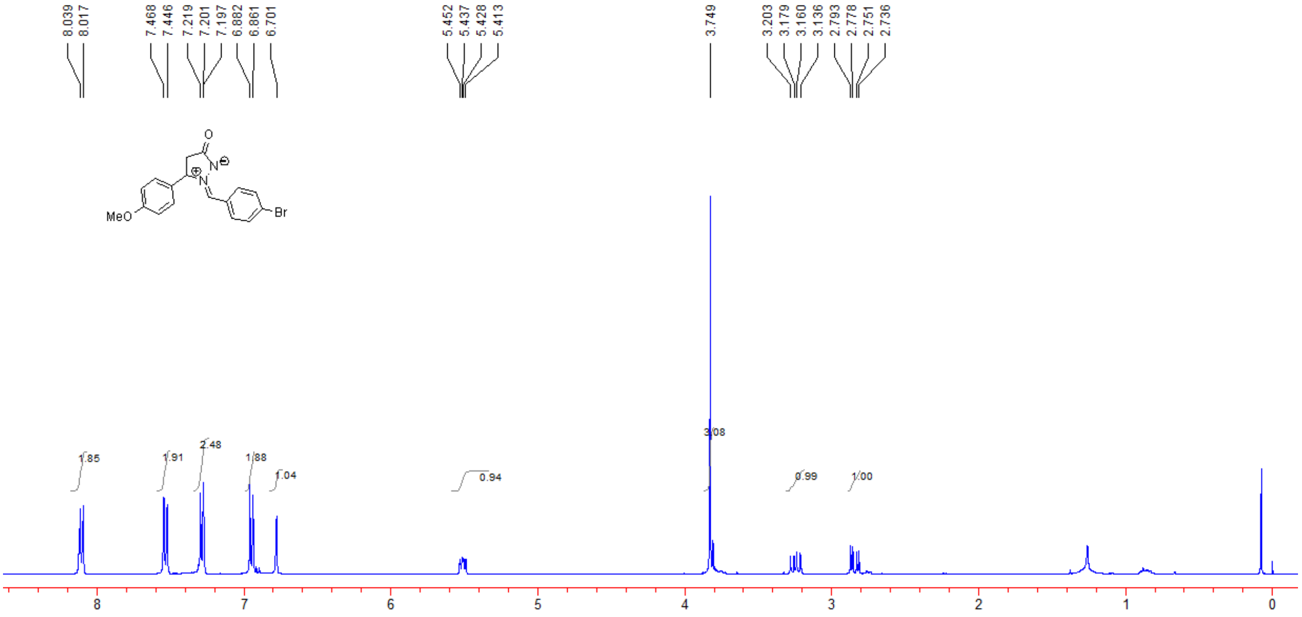

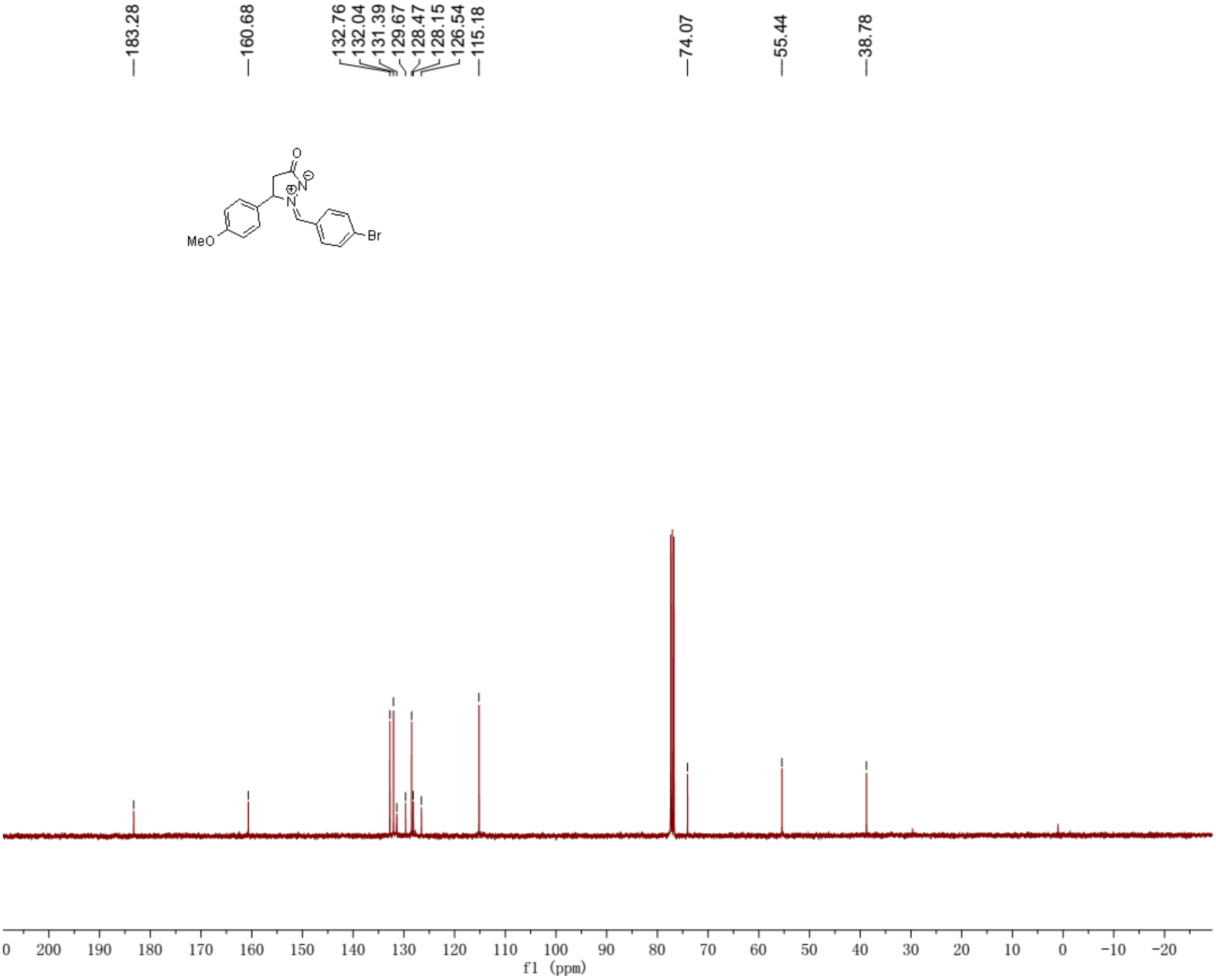

Supplementary Figure 29. ^{1}H and ^{13}C-NMR spectra of product **6c**.

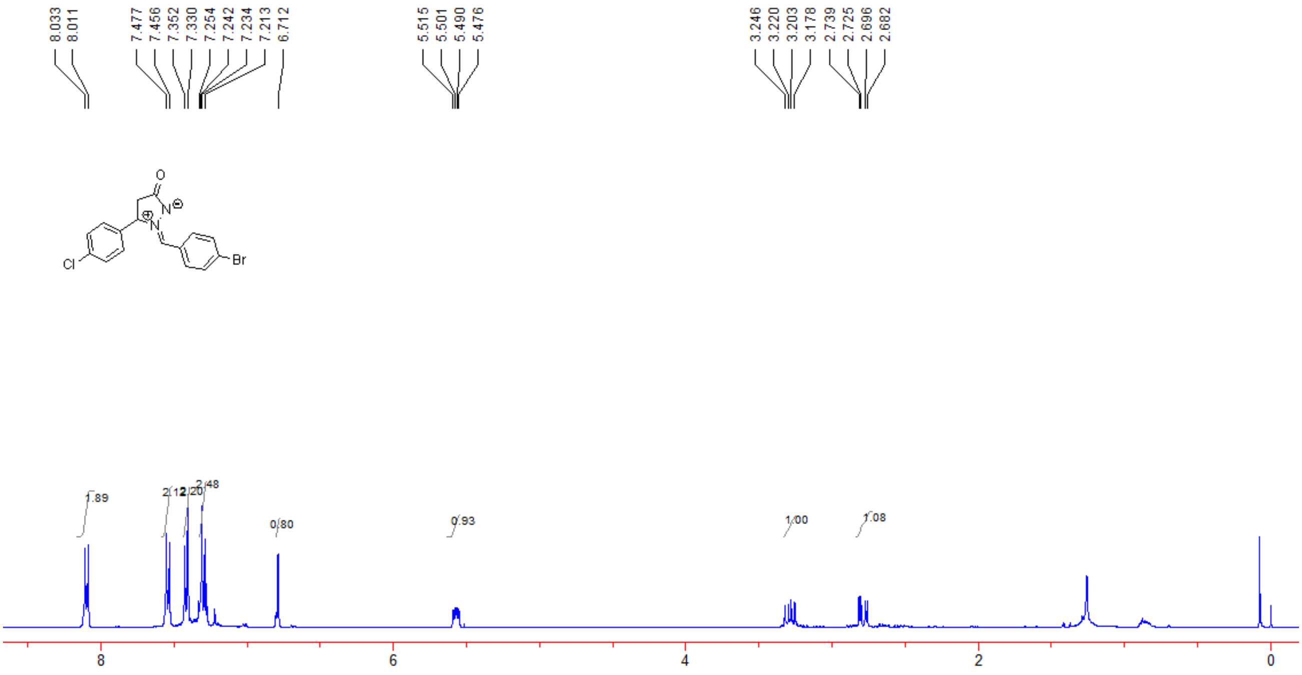

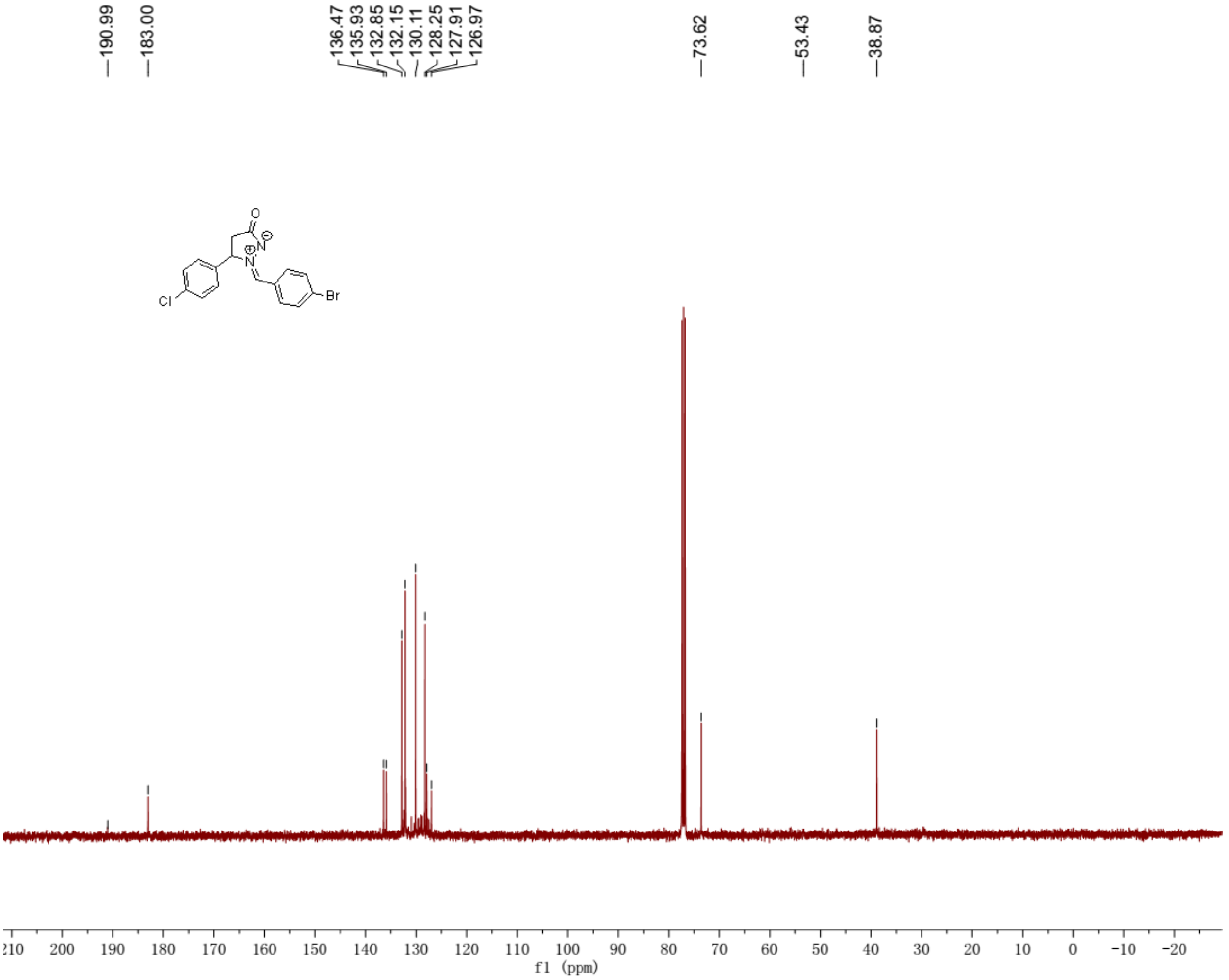

Supplementary Figure 30. ^{1}H and ^{13}C-NMR spectra of product **6d**.

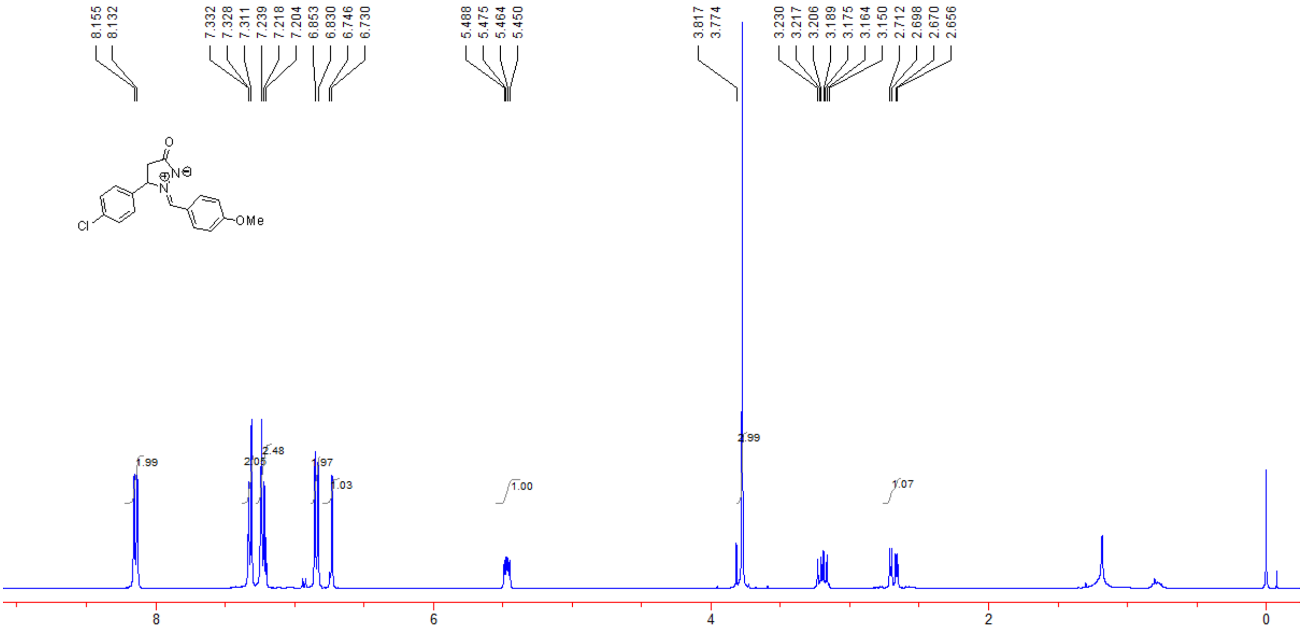

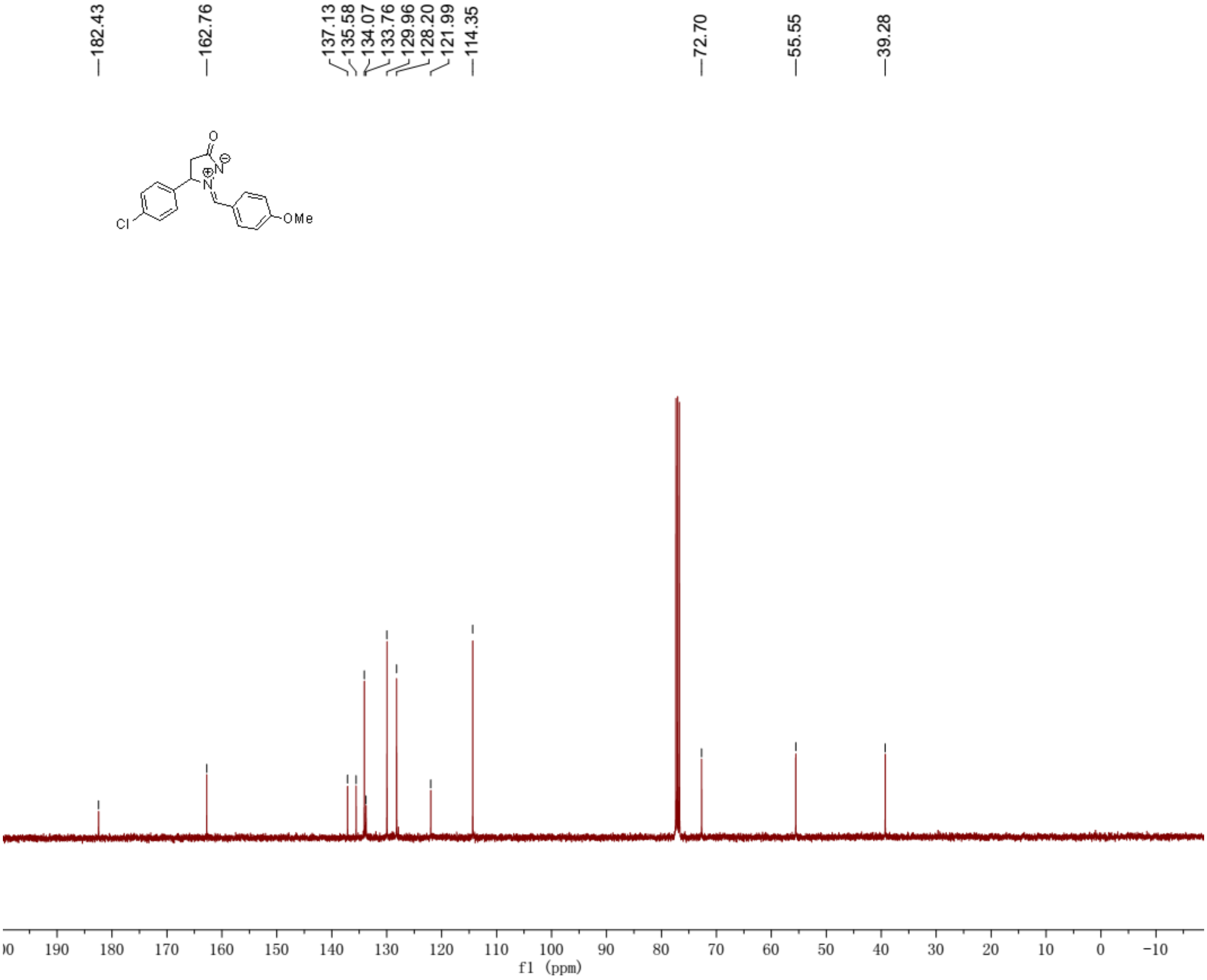

Supplementary Figure 31. ^{1}H and ^{13}C-NMR spectra of product **6e**.

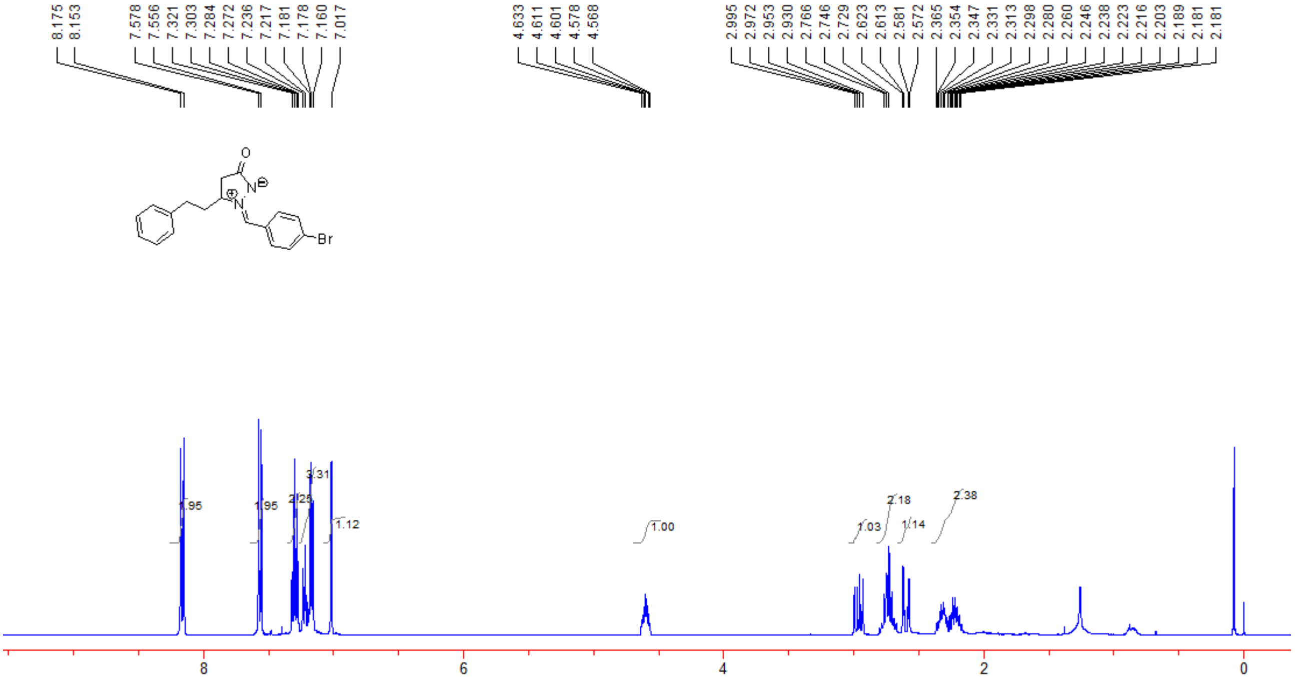

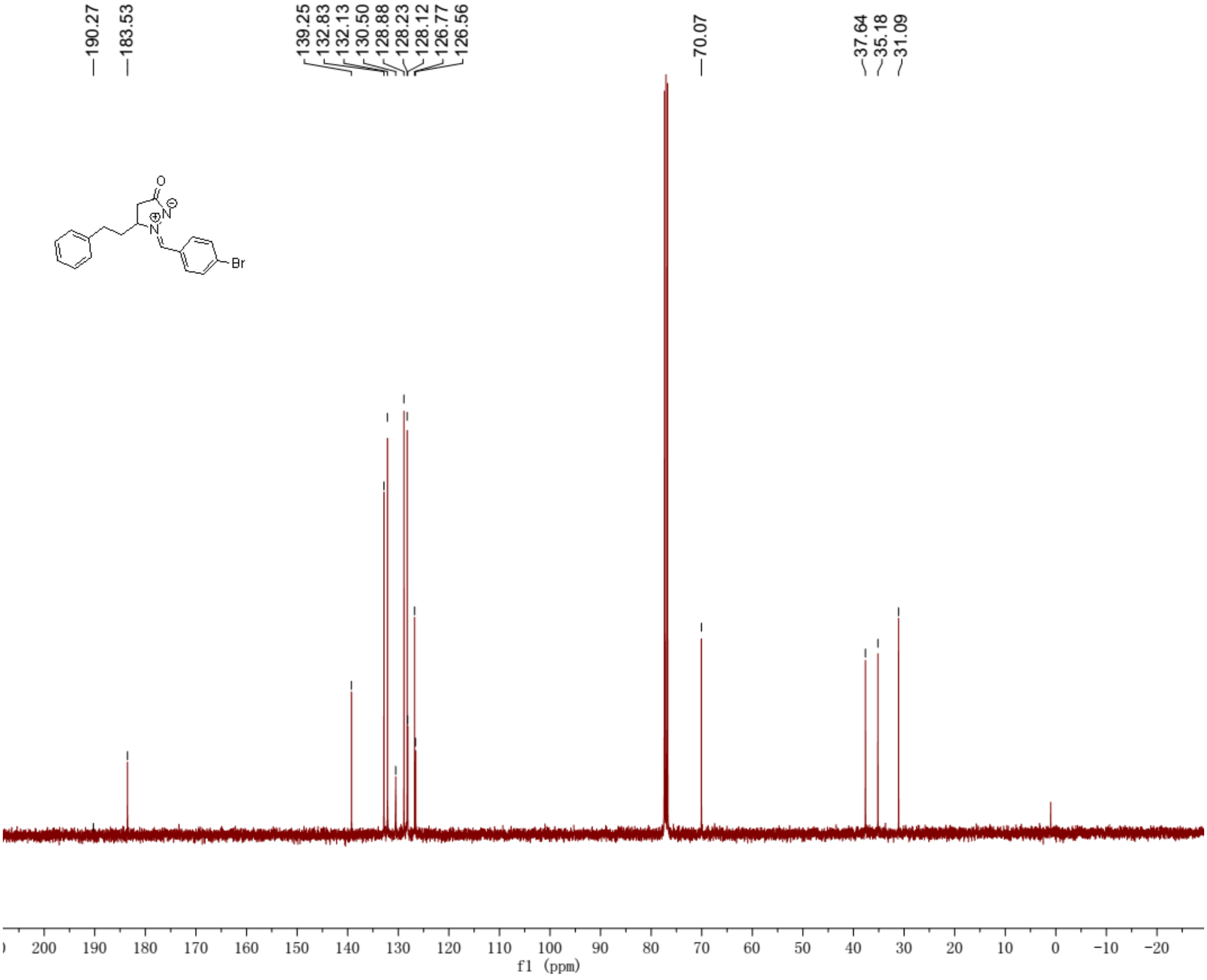

Supplementary Figure 32. ^{1}H and ^{13}C-NMR spectra of product **7a**.

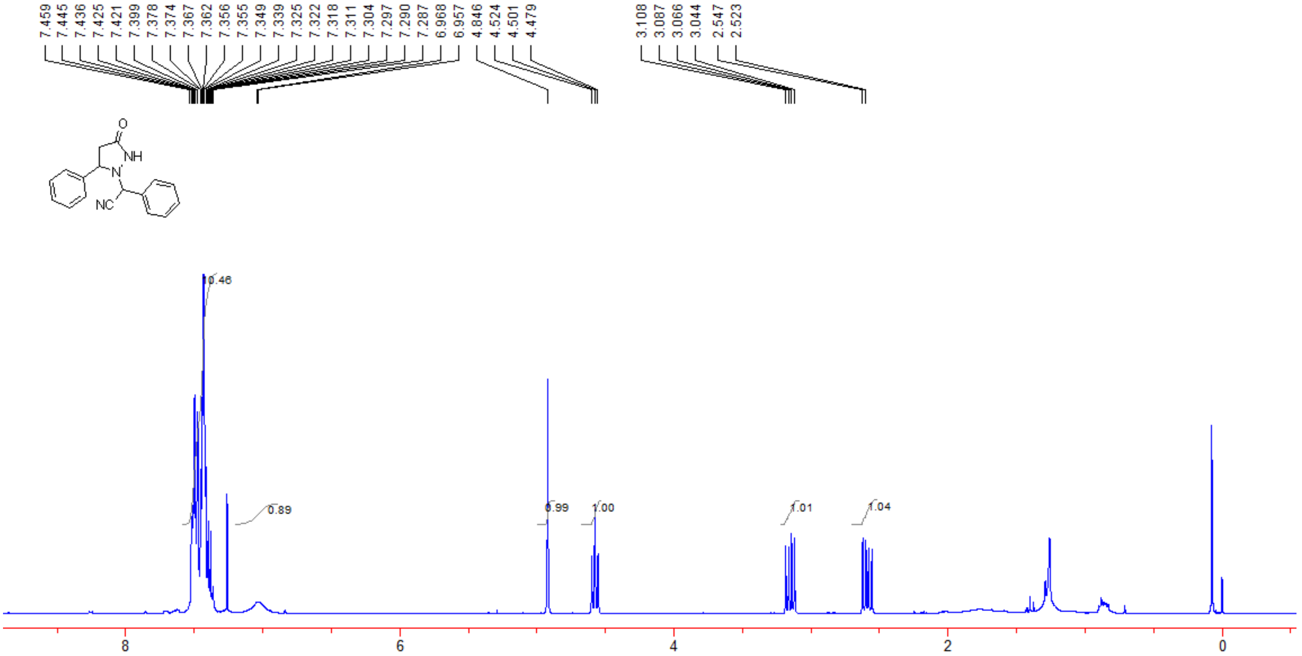

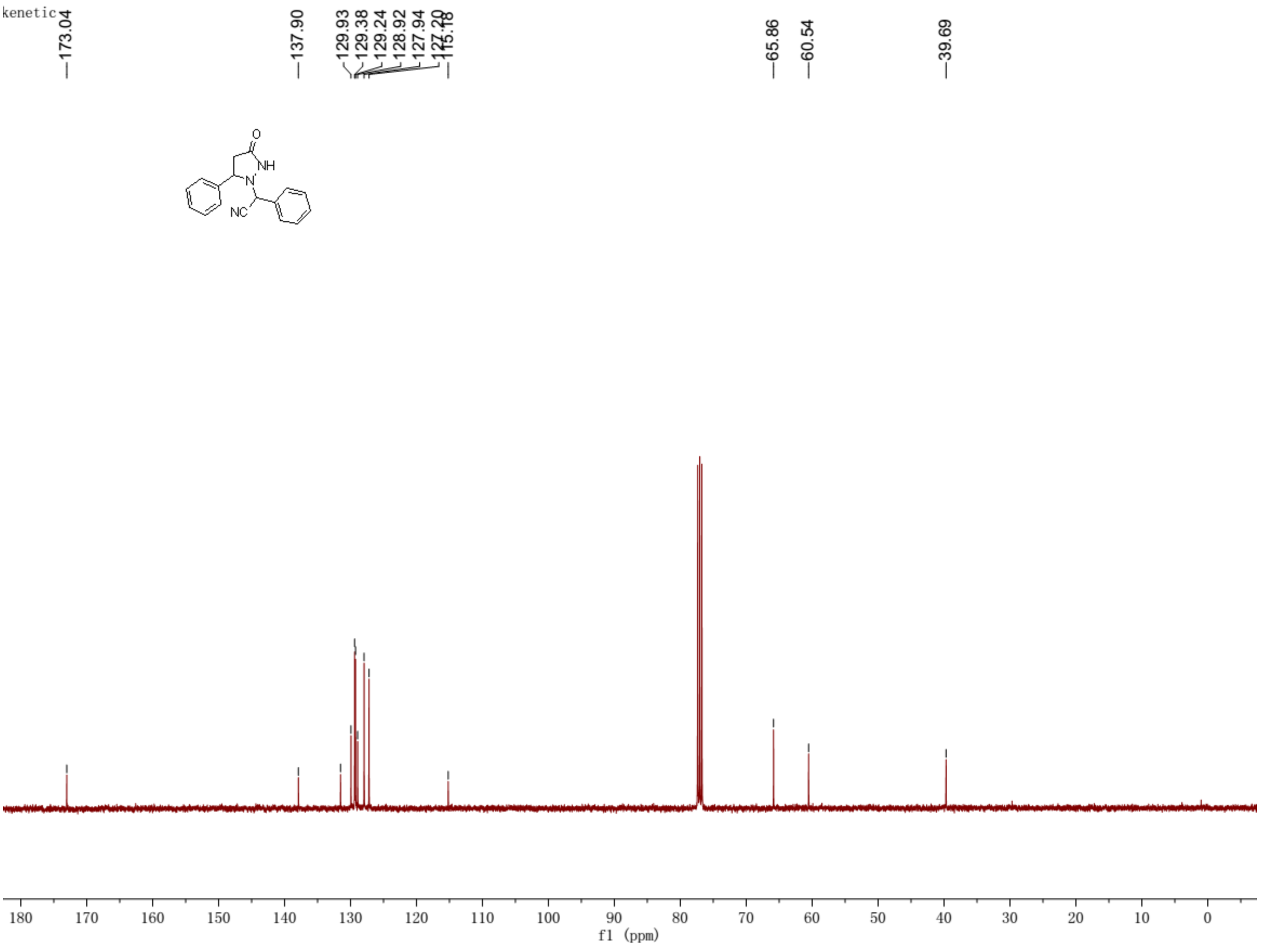

Supplementary Figure 33. ^{1}H and ^{13}C-NMR spectra of product **7b**.

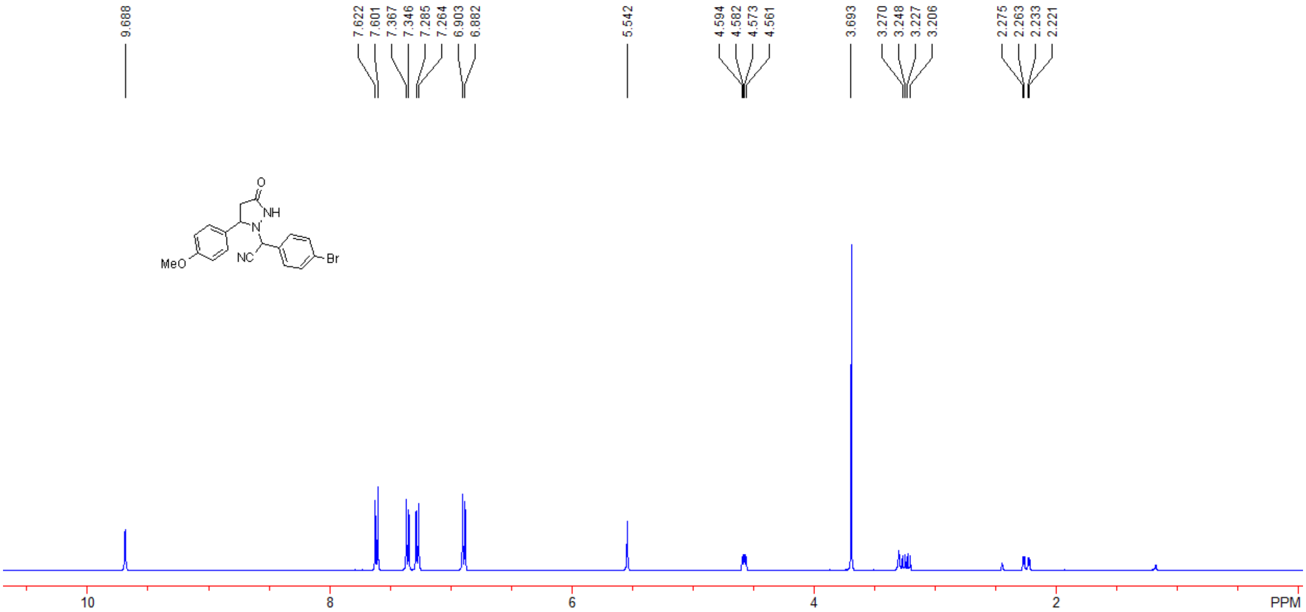

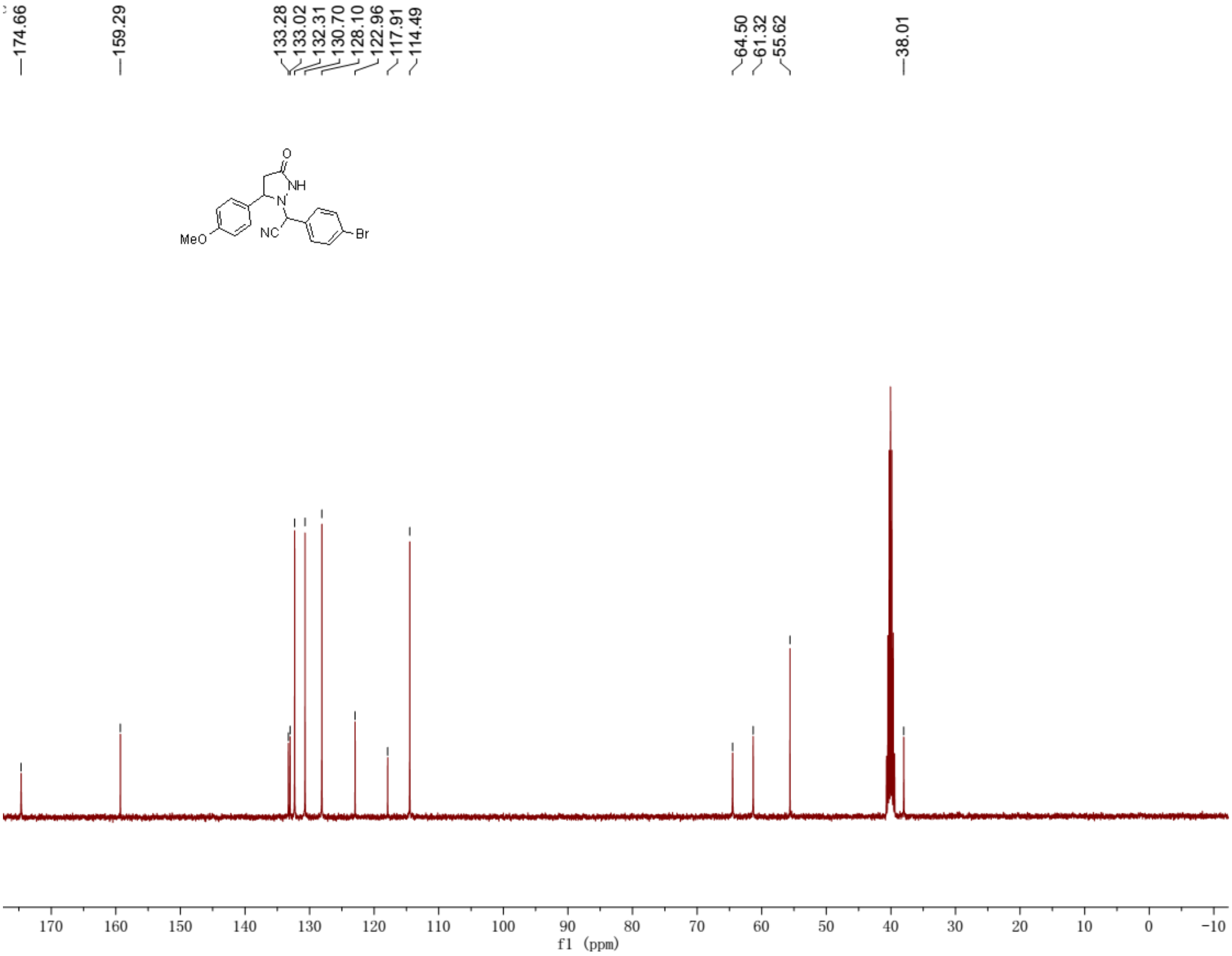

Supplementary Figure 34. ^{1}H and ^{13}C-NMR spectra of product **7c**.

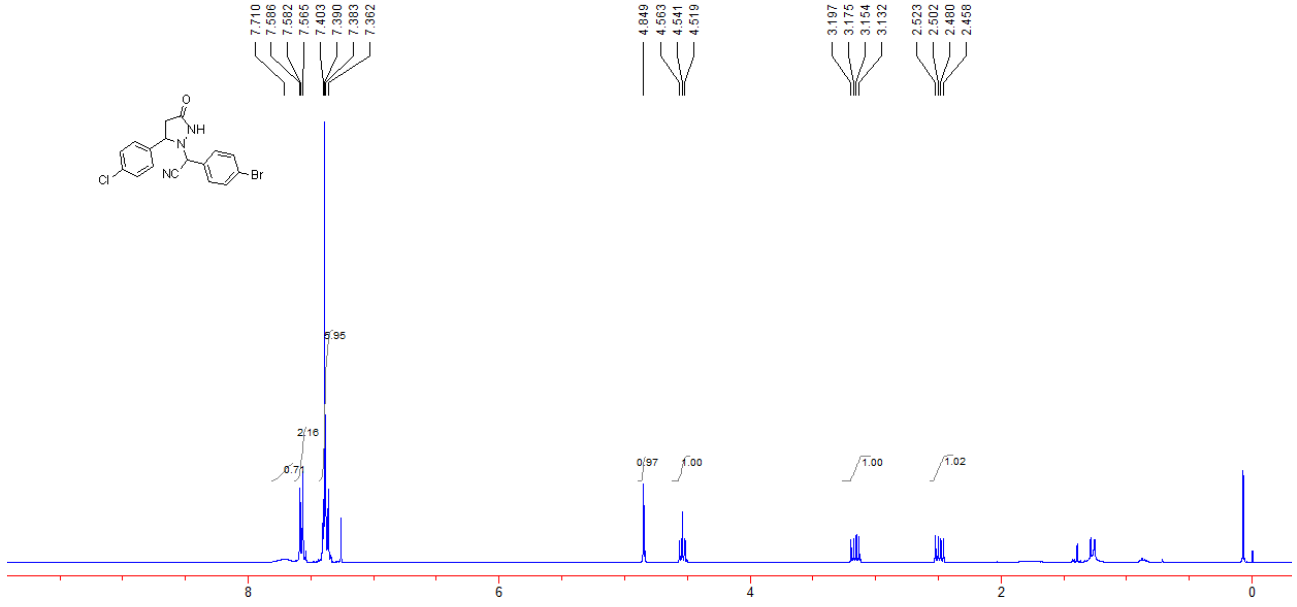

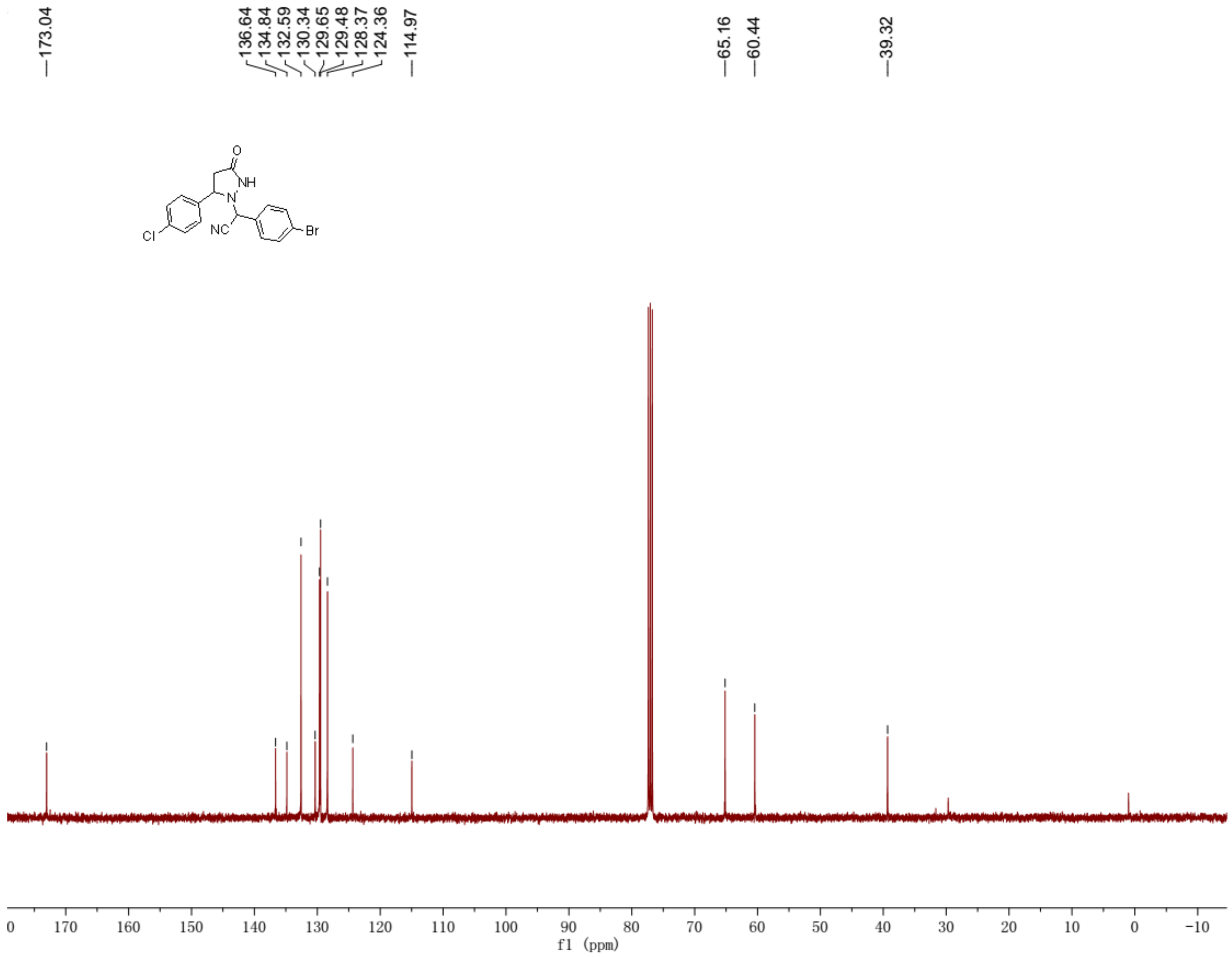

Supplementary Figure 35. ^{1}H and ^{13}C-NMR spectra of product **7d**.

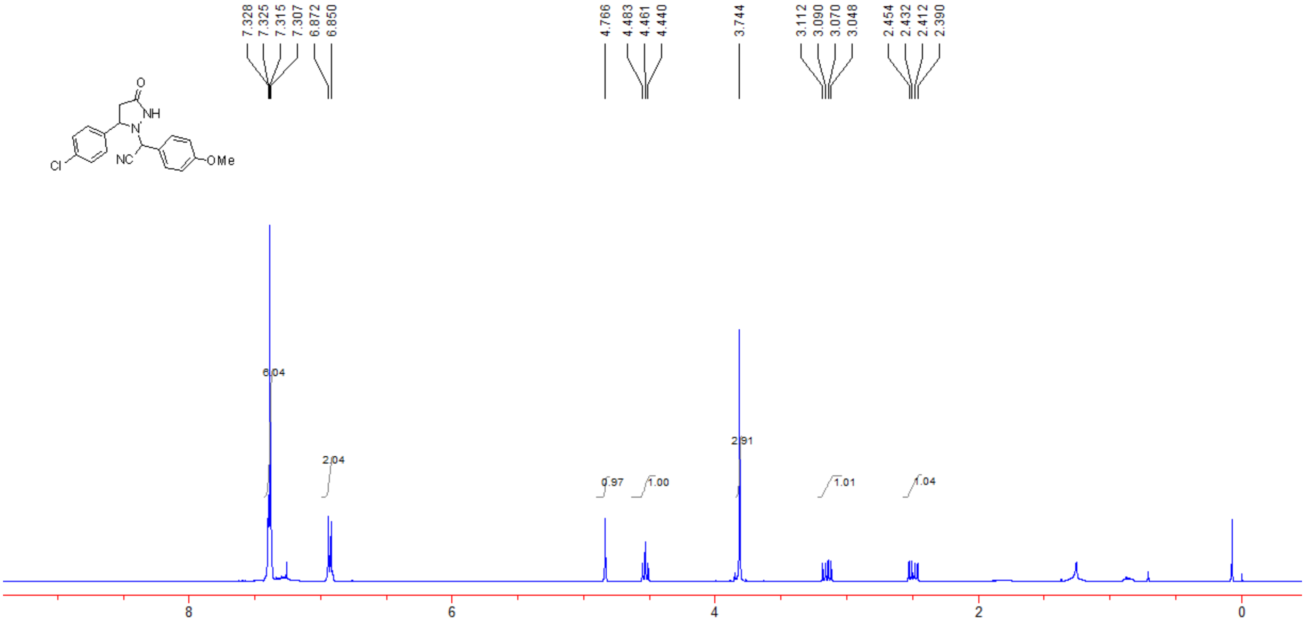

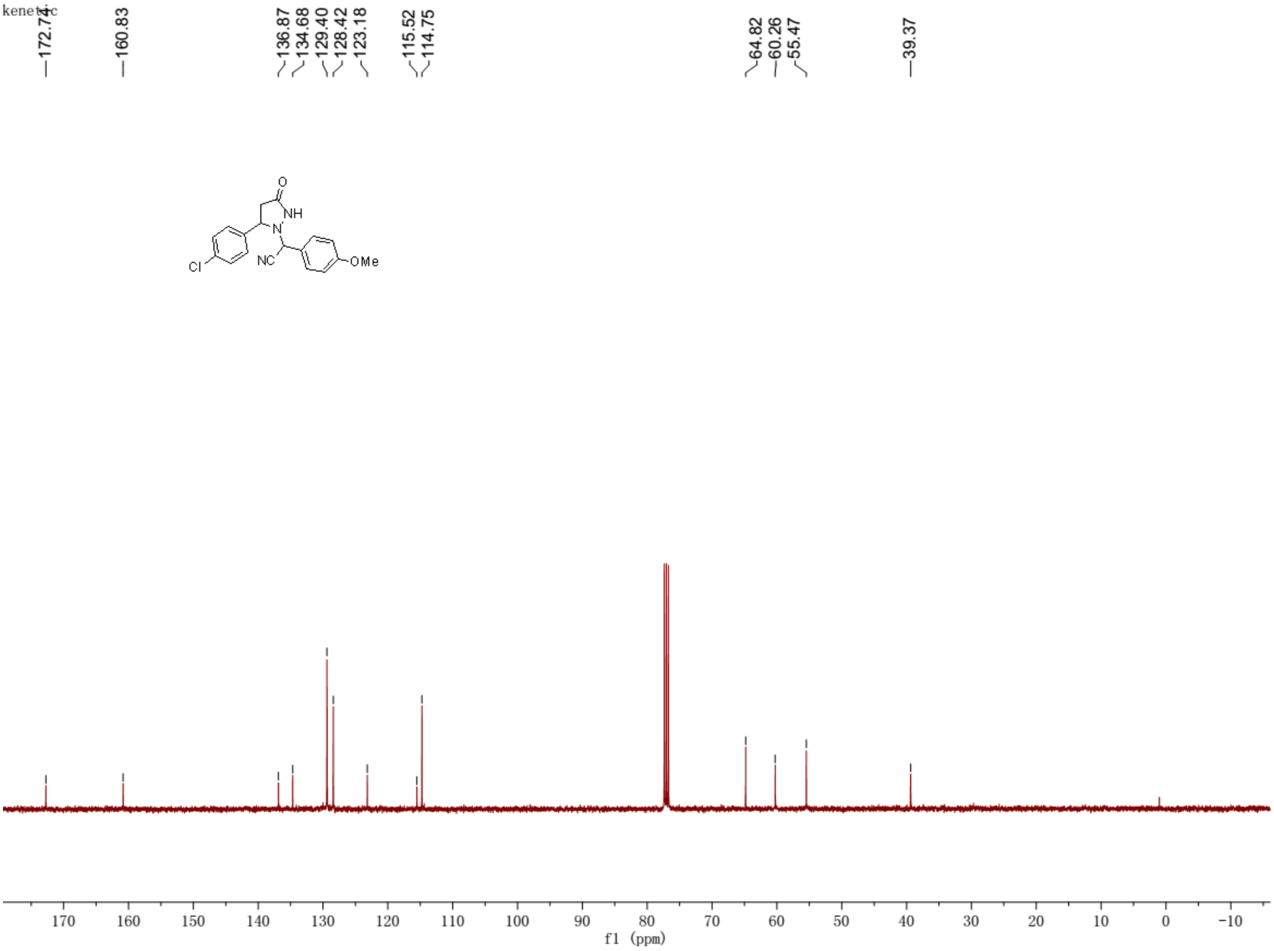

Supplementary Figure 36. ^{1}H and ^{13}C-NMR spectra of product **7e**.

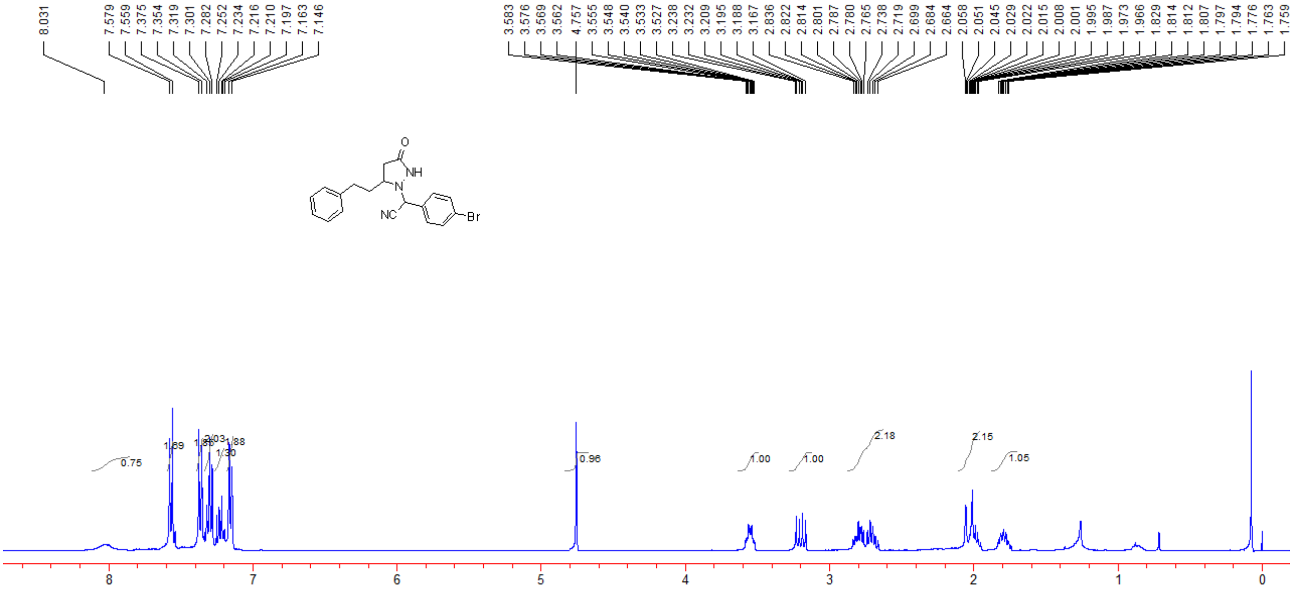

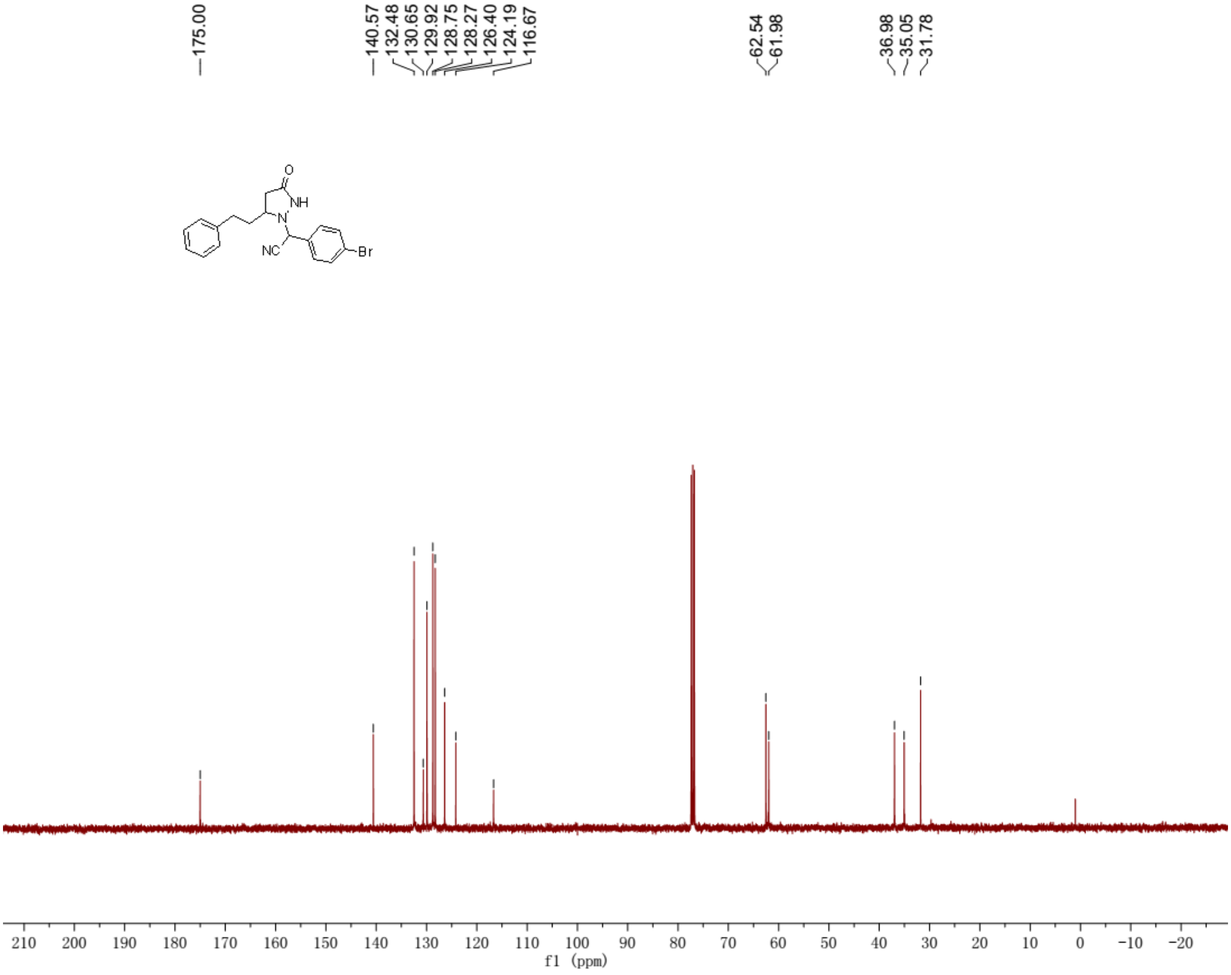

Supplementary Figure 37. HPLC spectra of products **2a**.

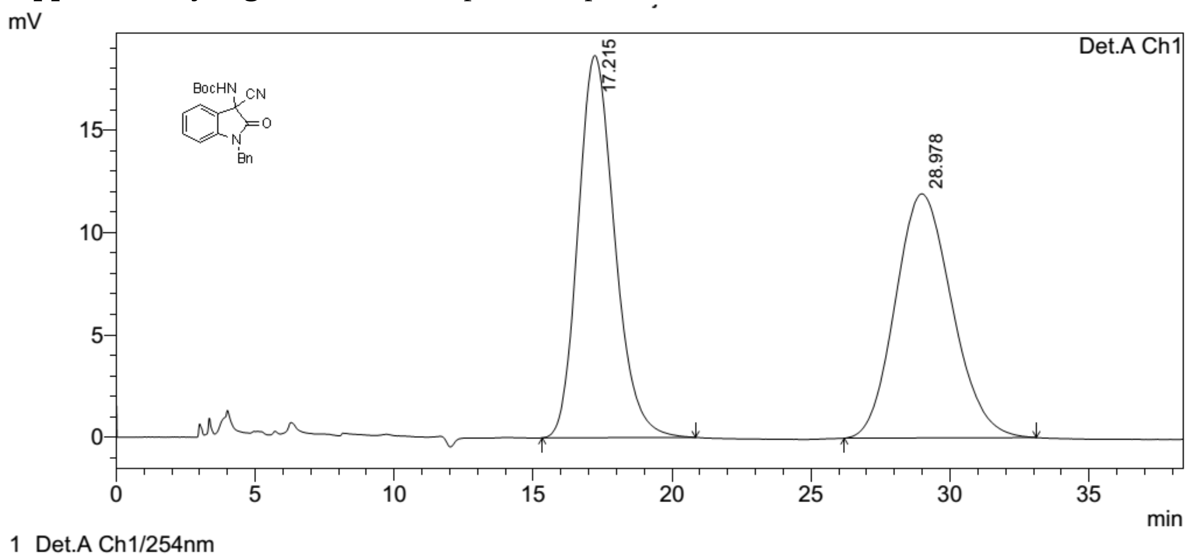

PeakTable

Detector A Ch1 254nm

Peak#	Ret. Time	Area	Height	Area %	Height %
1	17.215	1708194	18669	50.271	61.026
2	28.978	1689802	11923	49.729	38.974
Total		3397996	30592	100.000	100.000

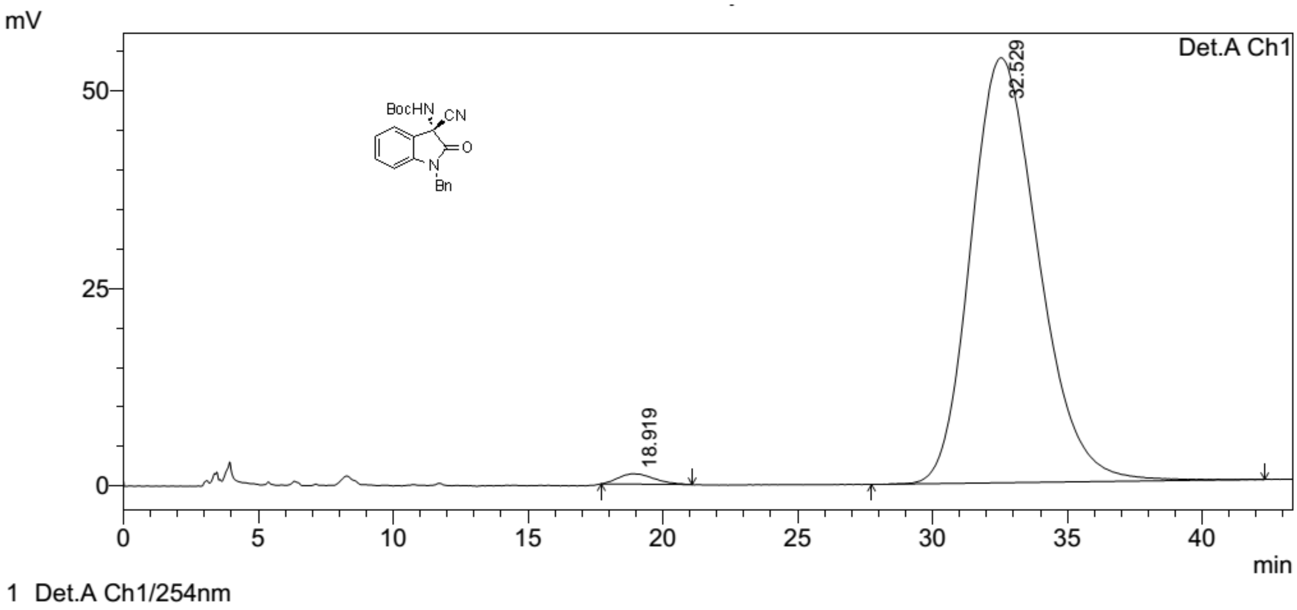

PeakTable

Detector A Ch1 254nm

Peak#	Ret. Time	Area	Height	Area %	Height %
1	18.919	120367	1324	1.262	2.401
2	32.529	9417609	53836	98.738	97.599
Total		9537975	55160	100.000	100.000

Supplementary Figure 38. HPLC spectra of products **2b**.

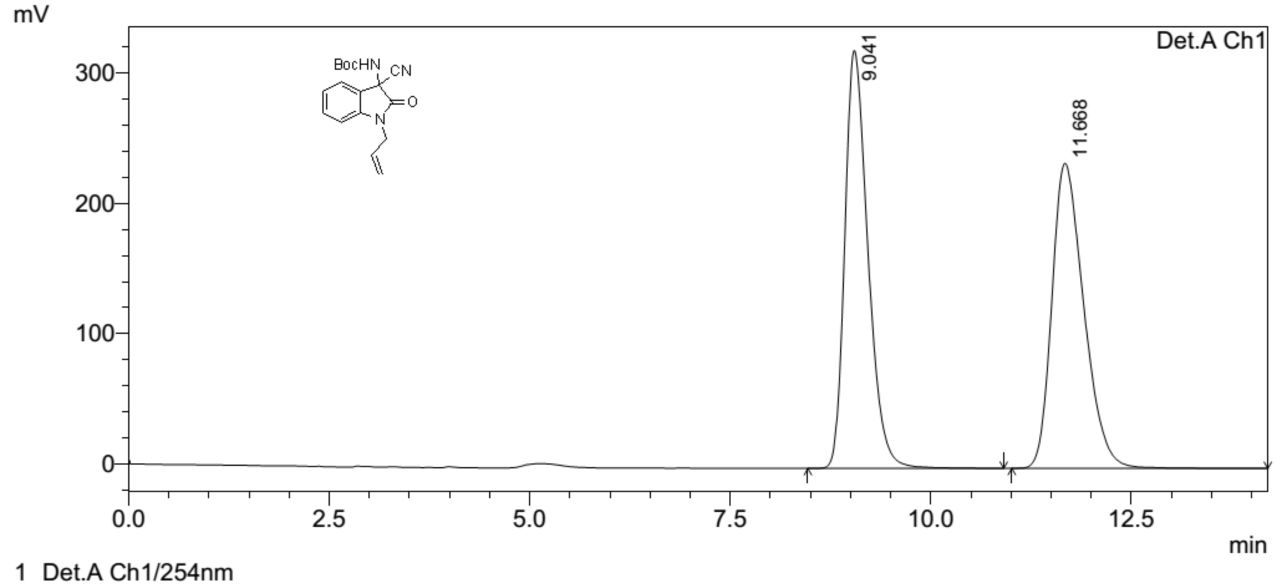

PeakTable

Detector A Ch1 254nm

Peak#	Ret. Time	Area	Height	Area %	Height %
1	9.041	6427904	320921	49.784	57.796
2	11.668	6483670	234347	50.216	42.204
Total		12911574	555268	100.000	100.000

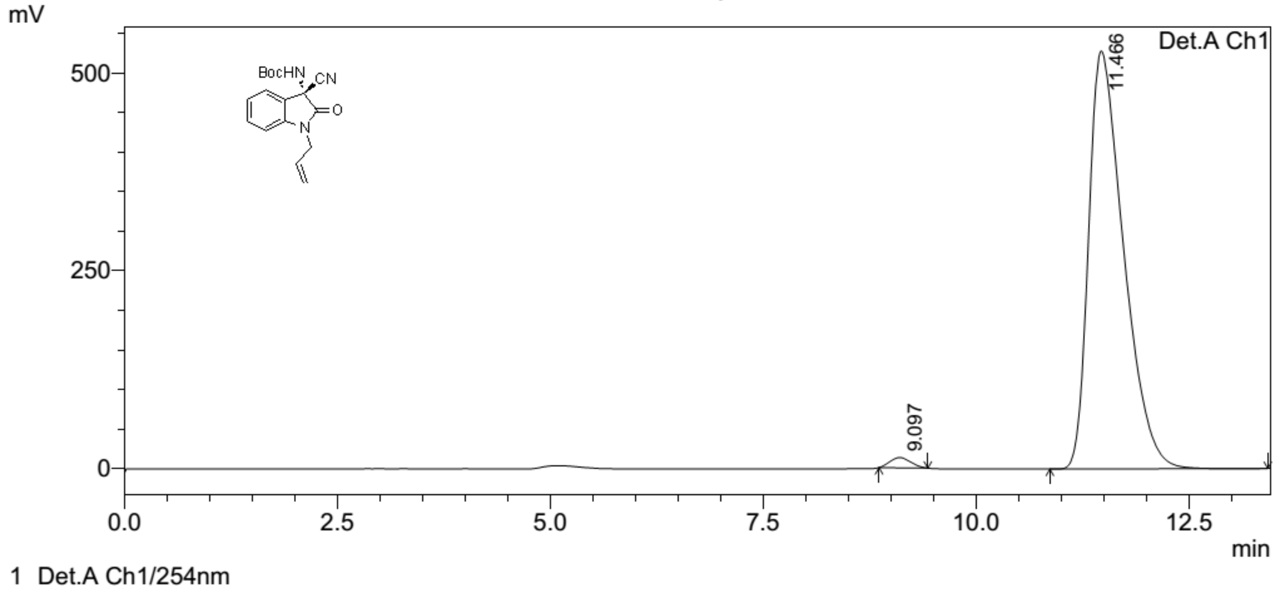

PeakTable

Detector A Ch1 254nm

Peak#	Ret. Time	Area	Height	Area %	Height %
1	9.097	220639	13047	1.445	2.413
2	11.466	15046393	527613	98.555	97.587
Total		15267032	540660	100.000	100.000

Supplementary Figure 39. HPLC spectra of products **2c**.

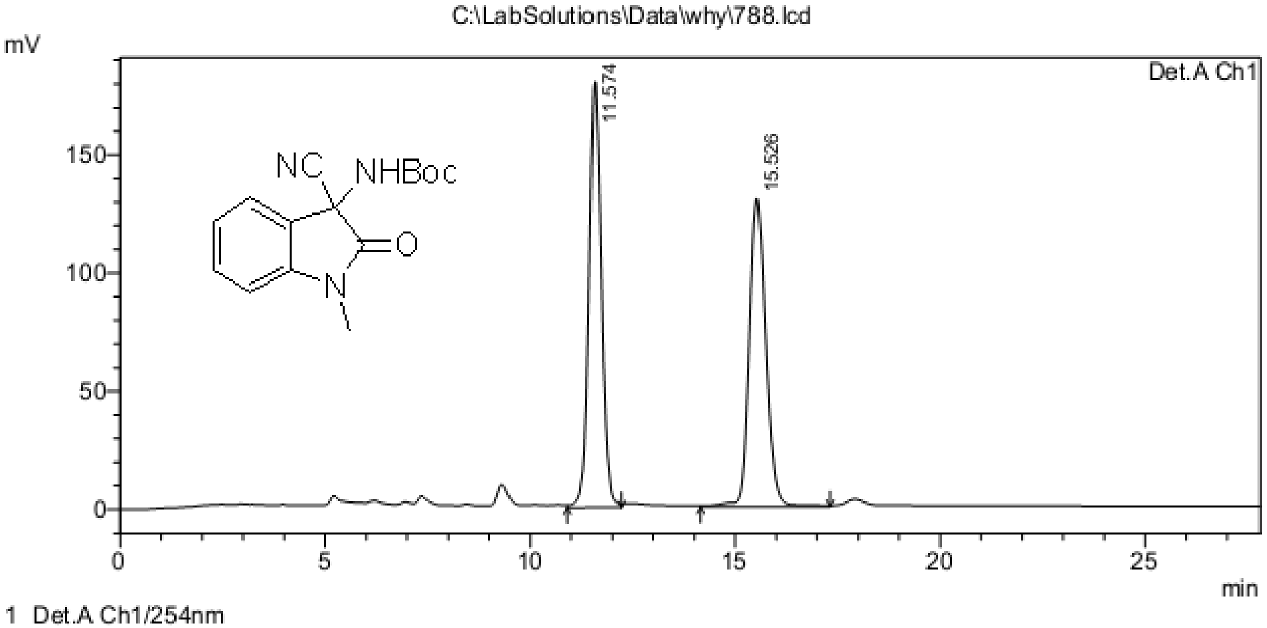

PeakTable

Detector A Ch1 254nm

Peak#	Ret. Time	Area	Height	Area %	Height %
1	11.574	3688235	180209	50.483	58.016
2	15.526	3617729	130409	49.517	41.984
Total		7305963	310618	100.000	100.000

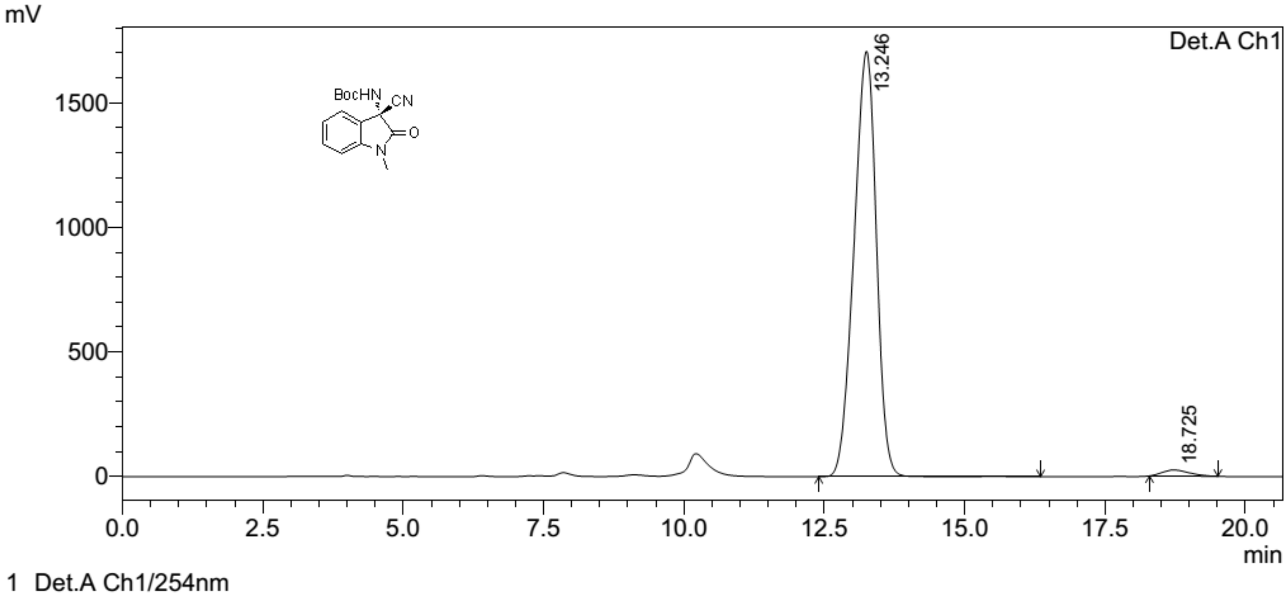

PeakTable

Detector A Ch1 254nm

Peak#	Ret. Time	Area	Height	Area %	Height %
1	13.246	47242983	1706740	98.301	98.595
2	18.725	816524	24330	1.699	1.405
Total		48059507	1731070	100.000	100.000

Supplementary Figure 40. HPLC spectra of products **2d**.

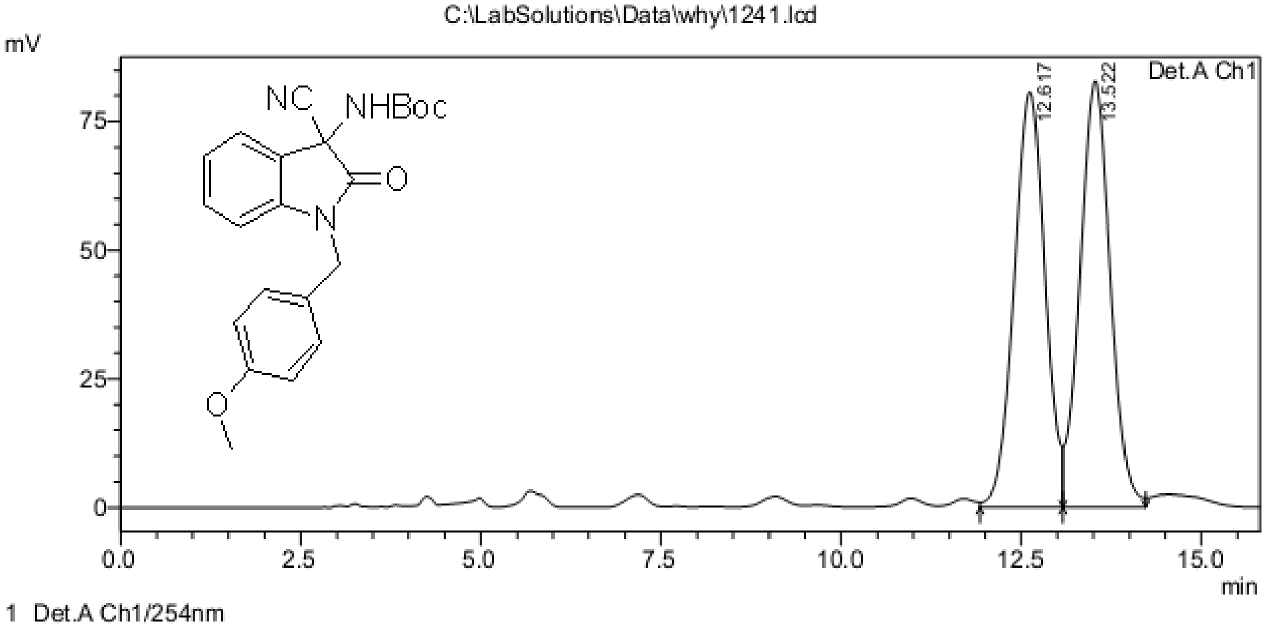

PeakTable

Detector A Ch1 254nm

Peak#	Ret. Time	Area	Height	Area %	Height %
1	12.617	2394798	80742	49.549	49.338
2	13.522	2438422	82908	50.451	50.662
Total		4833220	163650	100.000	100.000

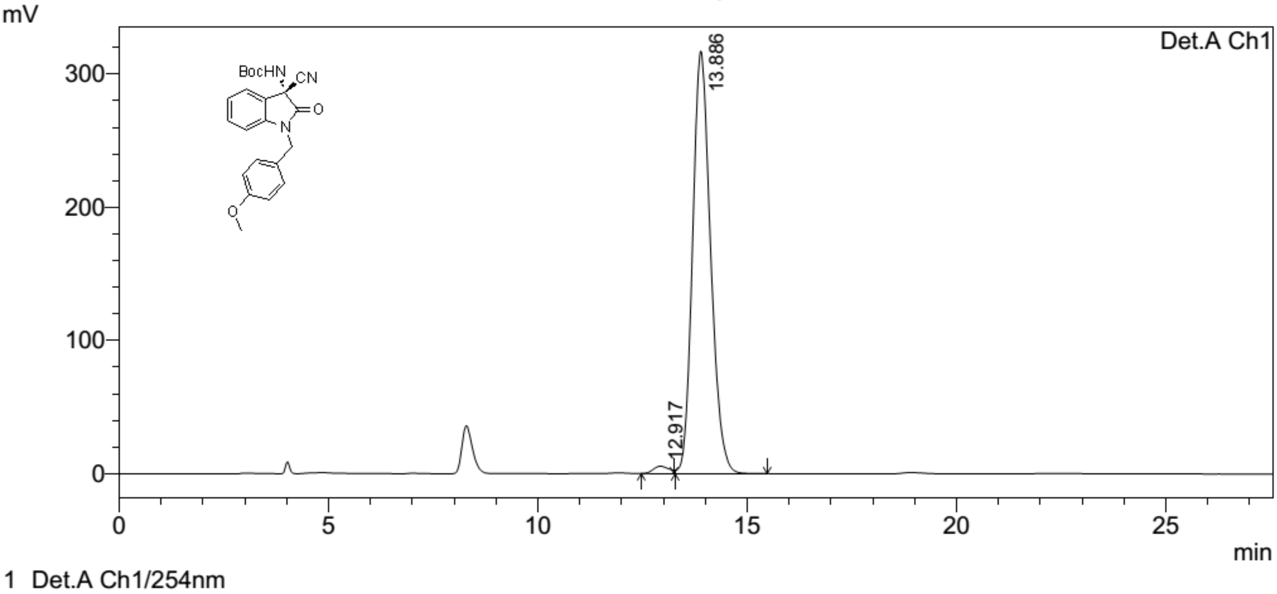

PeakTable

Detector A Ch1 254nm

Peak#	Ret. Time	Area	Height	Area %	Height %
1	12.917	149833	5692	1.556	1.762
2	13.886	9480546	317356	98.444	98.238
Total		9630379	323048	100.000	100.000

Supplementary Figure 41. HPLC spectra of products **2e**.

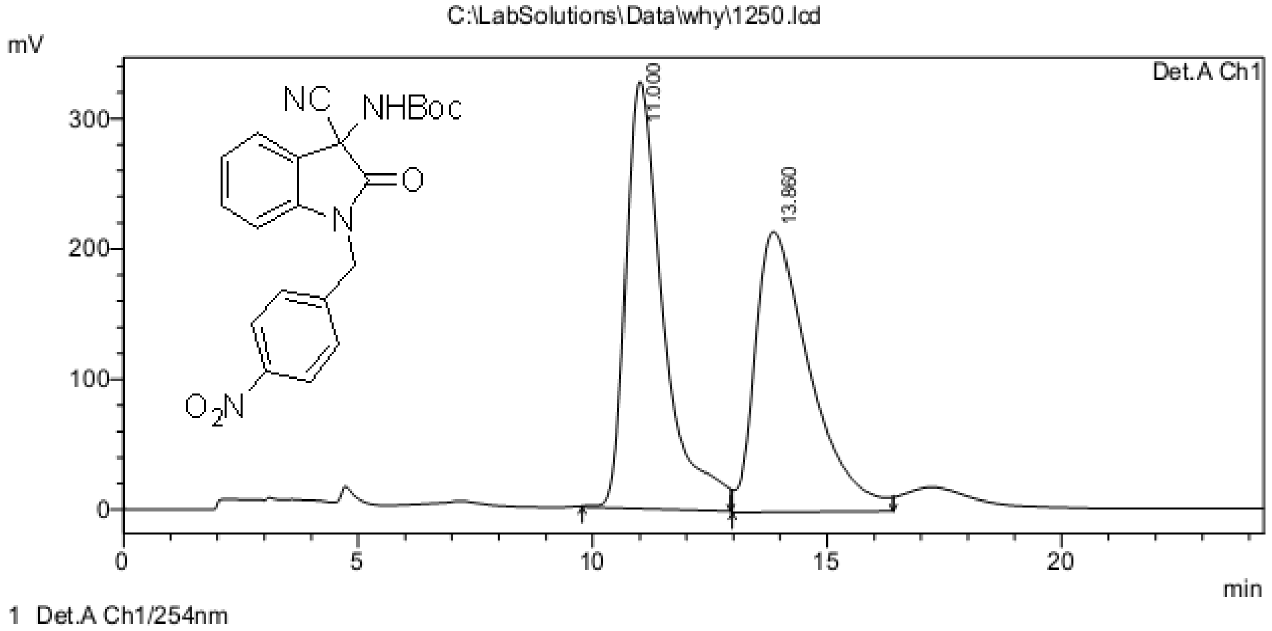

PeakTable

Detector A Ch1 254nm

Peak#	Ret. Time	Area	Height	Area %	Height %
1	11.000	18174208	327588	50.891	60.352
2	13.860	17537516	215209	49.109	39.648
Total		35711724	542798	100.000	100.000

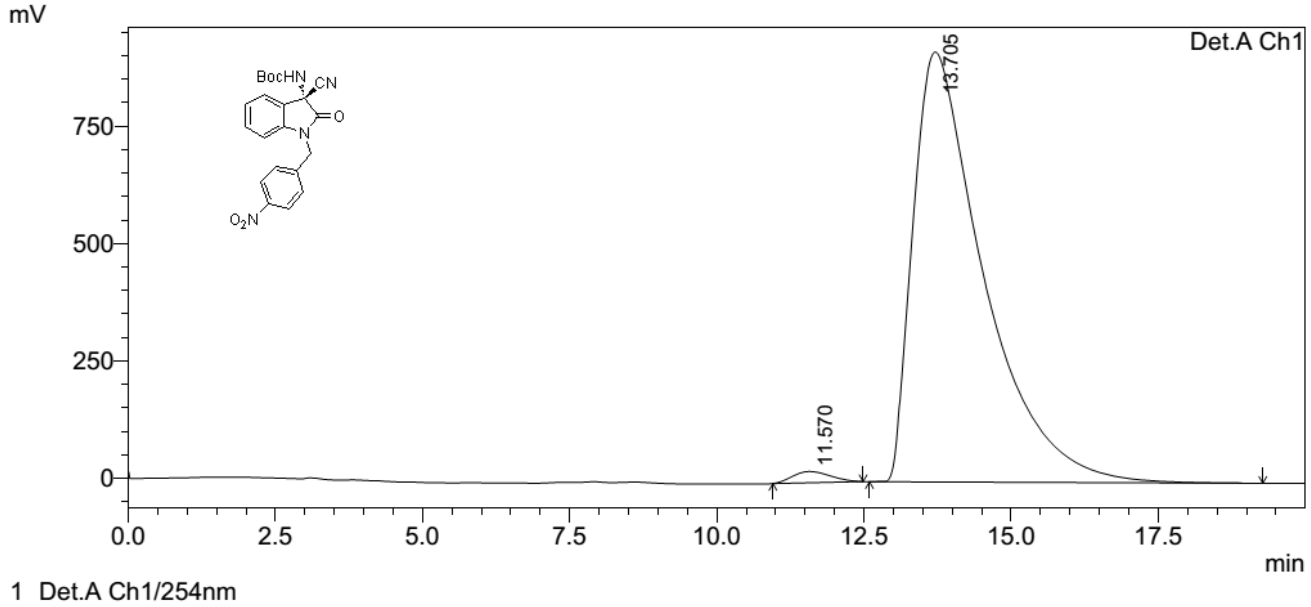

PeakTable

Detector A Ch1 254nm

Peak#	Ret. Time	Area	Height	Area %	Height %
1	11.570	1076315	24090	1.392	2.563
2	13.705	76250580	915915	98.608	97.437
Total		77326894	940005	100.000	100.000

Supplementary Figure 42. HPLC spectra of products **2f**.

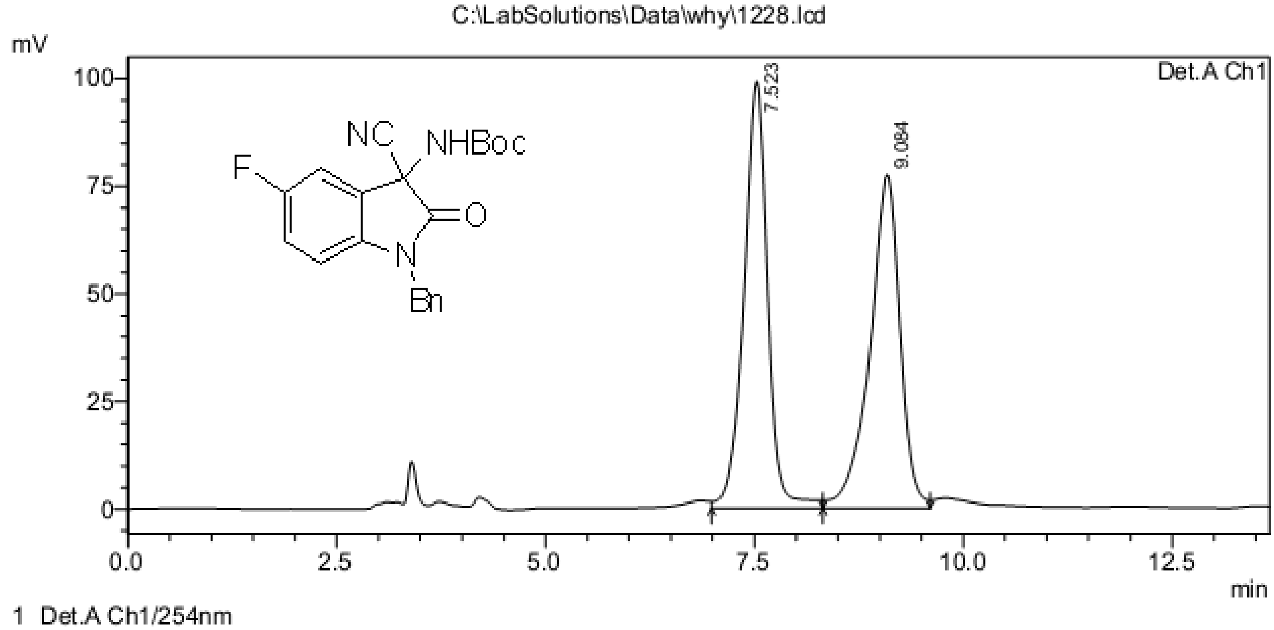

PeakTable

Detector A Ch1 254nm

Peak#	Ret. Time	Area	Height	Area %	Height %
1	7.523	1975649	99211	50.389	56.177
2	9.084	1945177	77395	49.611	43.823
Total		3920826	176606	100.000	100.000

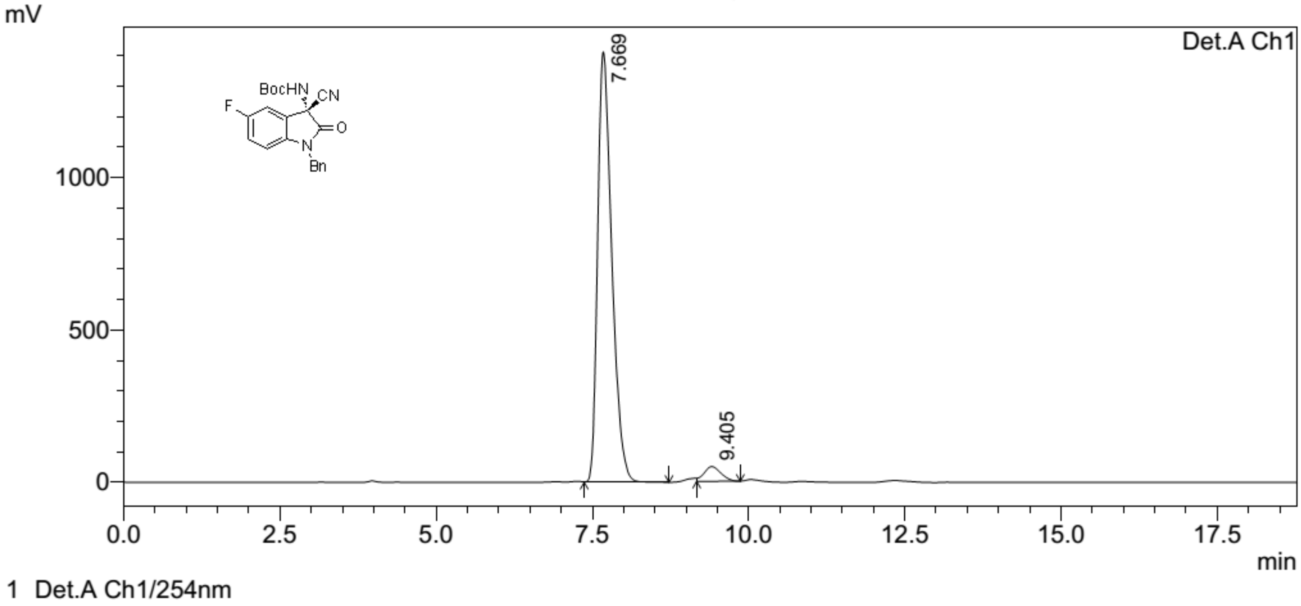

PeakTable

Detector A Ch1 254nm

Peak#	Ret. Time	Area	Height	Area %	Height %
1	7.669	22328935	1413866	96.077	96.653
2	9.405	911800	48957	3.923	3.347
Total		23240735	1462823	100.000	100.000

Supplementary Figure 43. HPLC spectra of products **2g**.

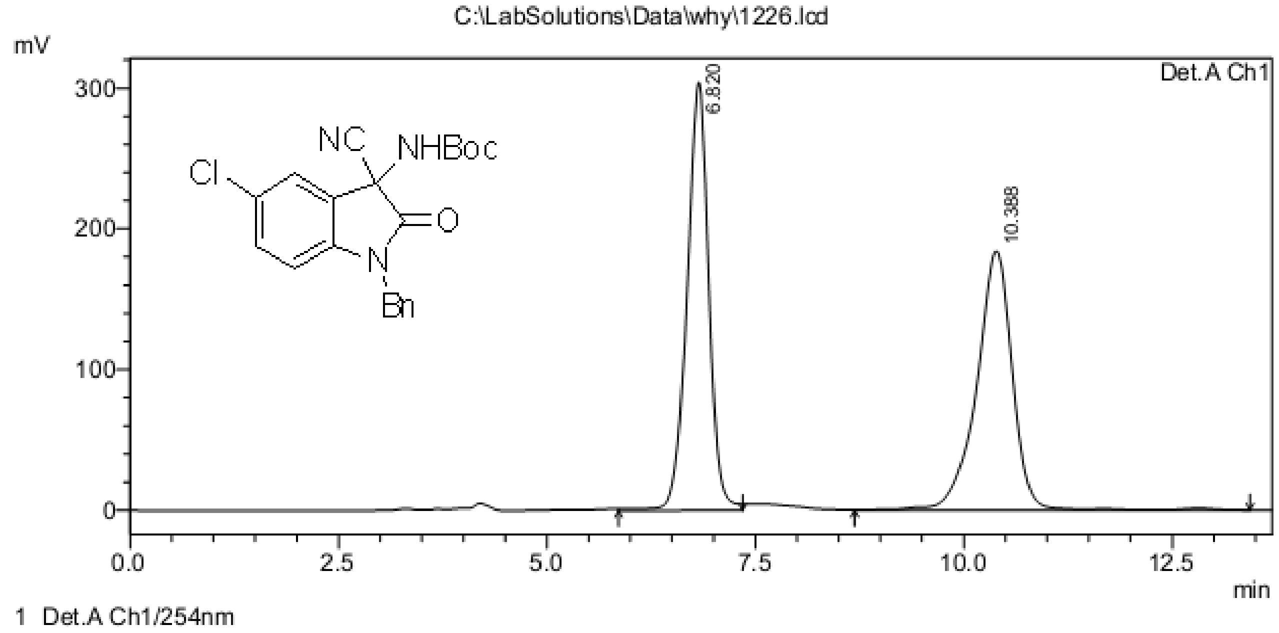

PeakTable

Detector A Ch1 254nm

Peak#	Ret. Time	Area	Height	Area %	Height %
1	6.820	5554831	304249	50.305	62.299
2	10.388	5487433	184121	49.695	37.701
Total		11042265	488370	100.000	100.000

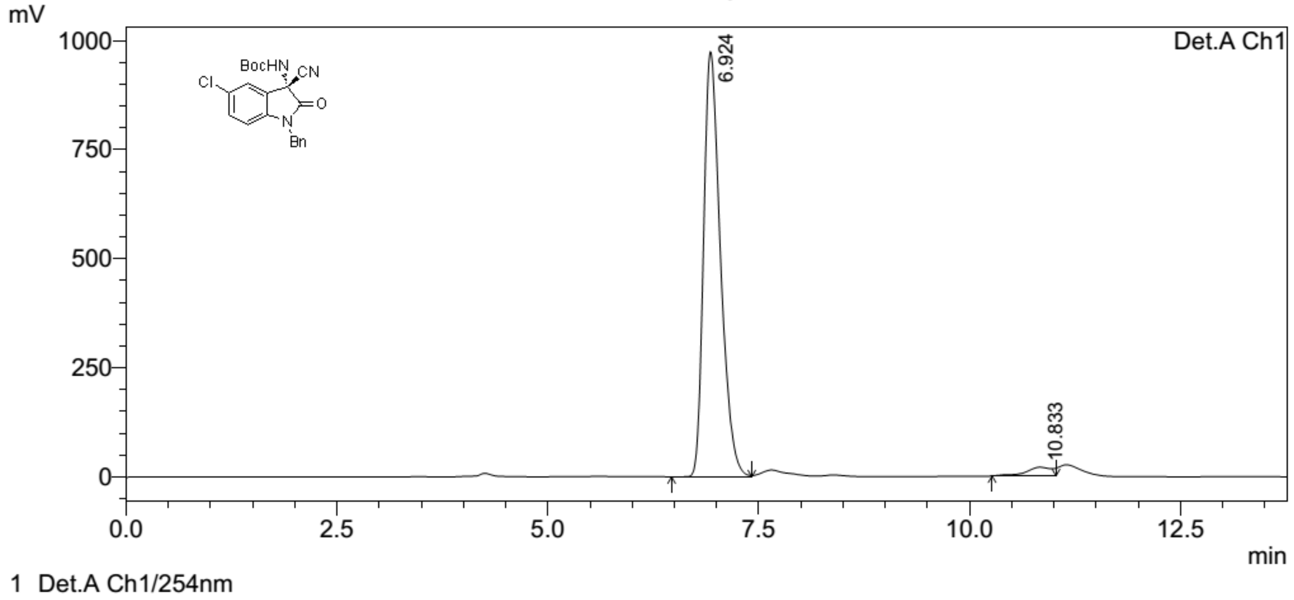

PeakTable

Detector A Ch1 254nm

Peak#	Ret. Time	Area	Height	Area %	Height %
1	6.924	13543916	975281	96.856	97.992
2	10.833	439665	19982	3.144	2.008
Total		13983582	995263	100.000	100.000

Supplementary Figure 44. HPLC spectra of products **2h**.

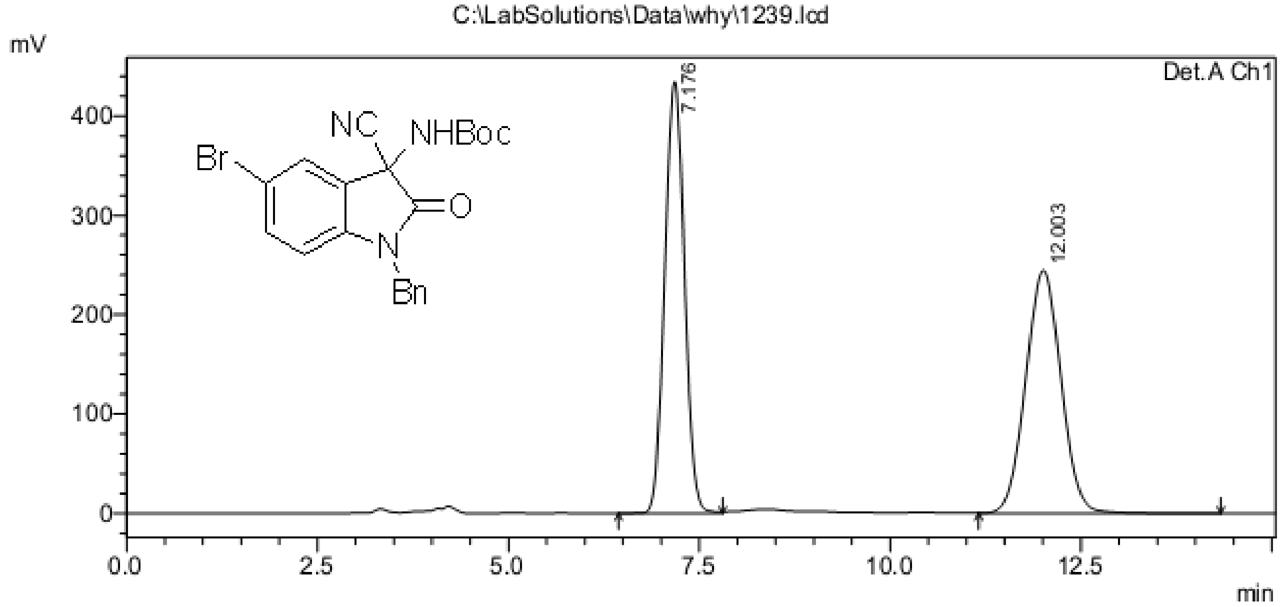

1 Det.A Ch1/254nm

PeakTable

Detector A Ch1 254nm

Peak#	Ret. Time	Area	Height	Area %	Height %
1	7.176	7803798	434459	49.760	63.975
2	12.003	7879157	244646	50.240	36.025
Total		15682955	679106	100.000	100.000

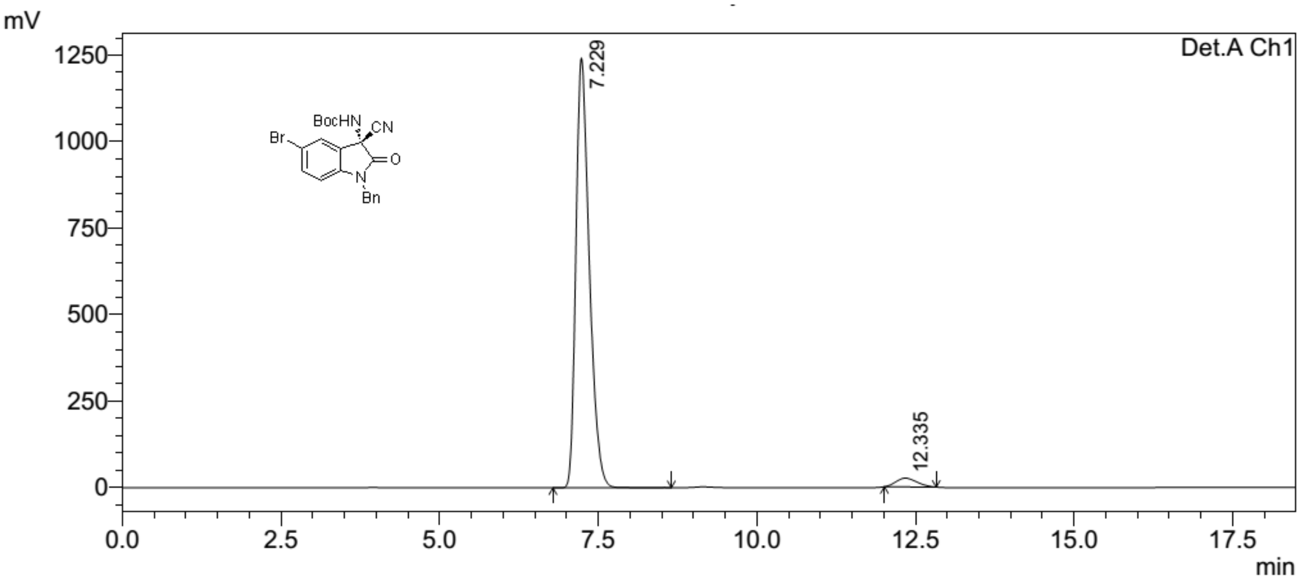

1 Det.A Ch1/254nm

PeakTable

Detector A Ch1 254nm

Peak#	Ret. Time	Area	Height	Area %	Height %
1	7.229	18276830	1242517	96.886	97.995
2	12.335	587519	25425	3.114	2.005
Total		18864348	1267942	100.000	100.000

Supplementary Figure 45. HPLC spectra of products **2i**.

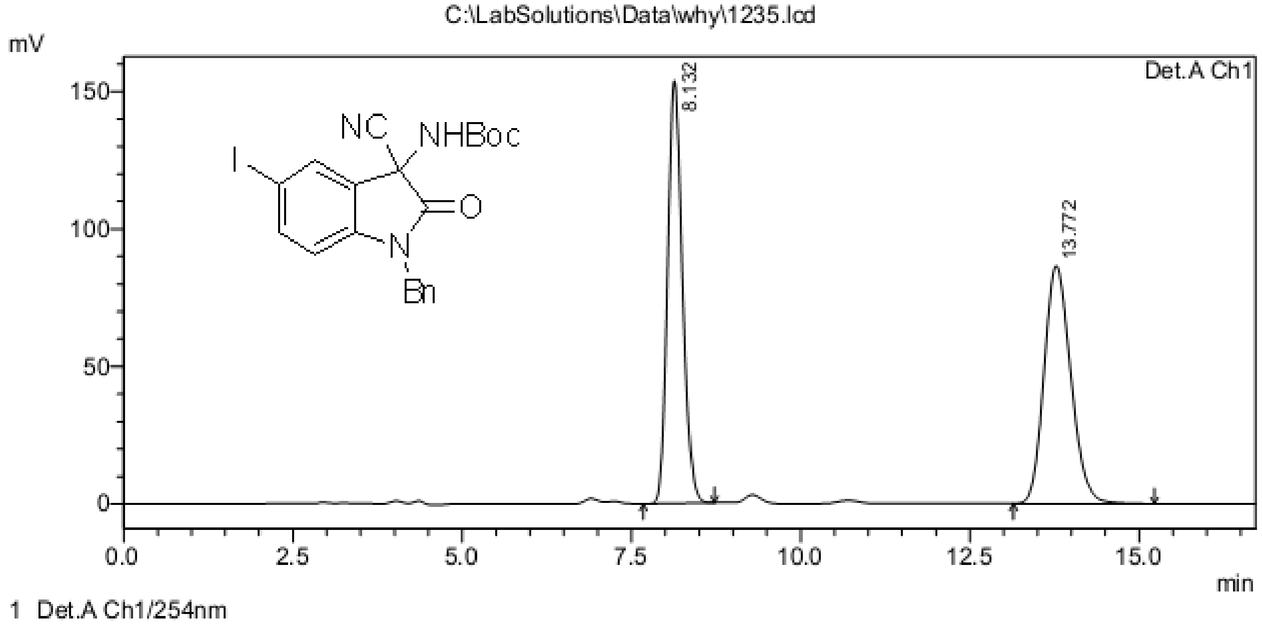

PeakTable

Detector A Ch1 254nm

Peak#	Ret. Time	Area	Height	Area %	Height %
1	8.132	2353758	153812	49.547	63.970
2	13.772	2396831	86632	50.453	36.030
Total		4750590	240444	100.000	100.000

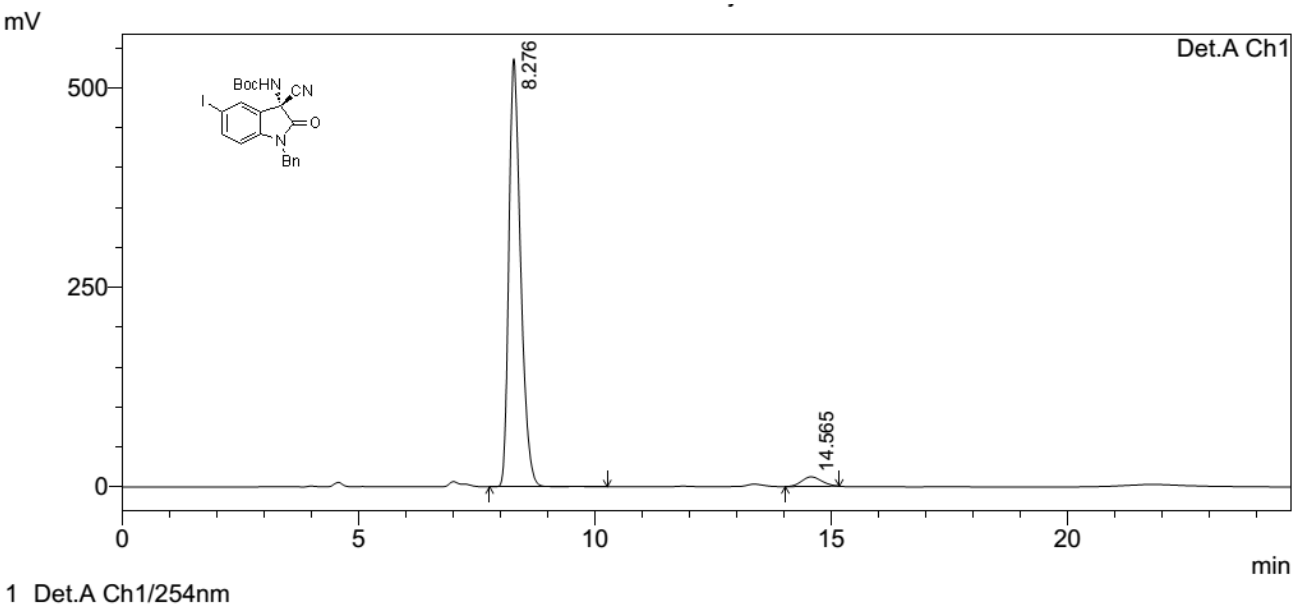

PeakTable

Detector A Ch1 254nm

Peak#	Ret. Time	Area	Height	Area %	Height %
1	8.276	9278323	536662	96.333	97.808
2	14.565	353231	12026	3.667	2.192
Total		9631554	548687	100.000	100.000

Supplementary Figure 46. HPLC spectra of products **2j**.

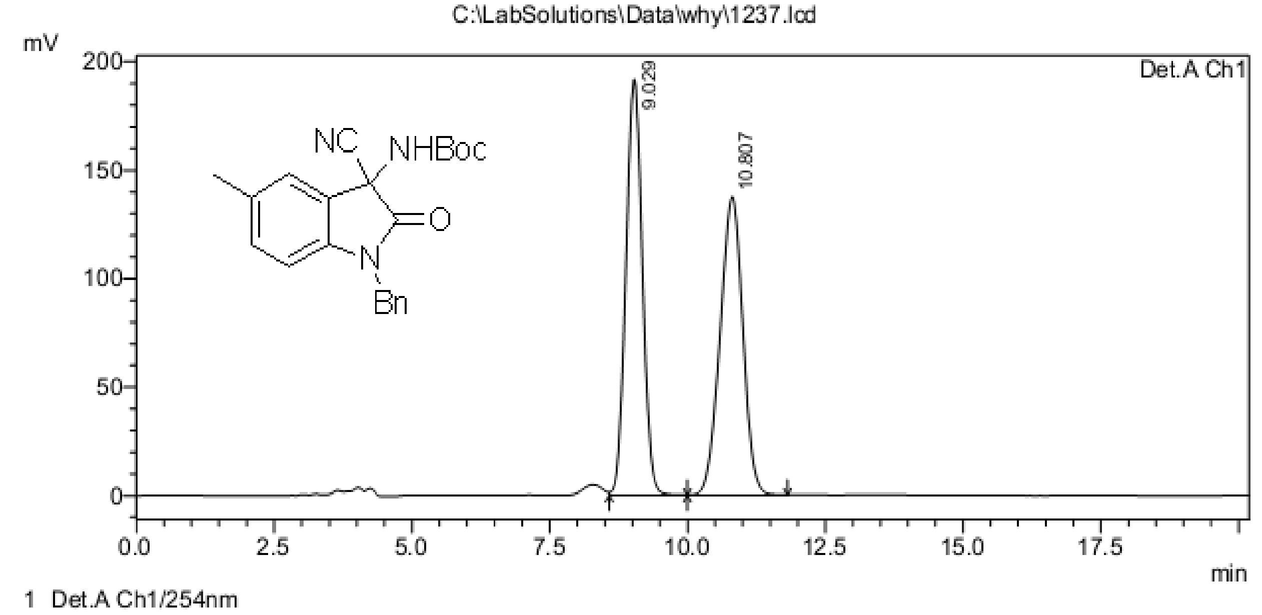

PeakTable

Detector A Ch1 254nm

Peak#	Ret. Time	Area	Height	Area %	Height %
1	9.029	3939093	191959	49.790	58.209
2	10.807	3972301	137819	50.210	41.791
Total		7911394	329777	100.000	100.000

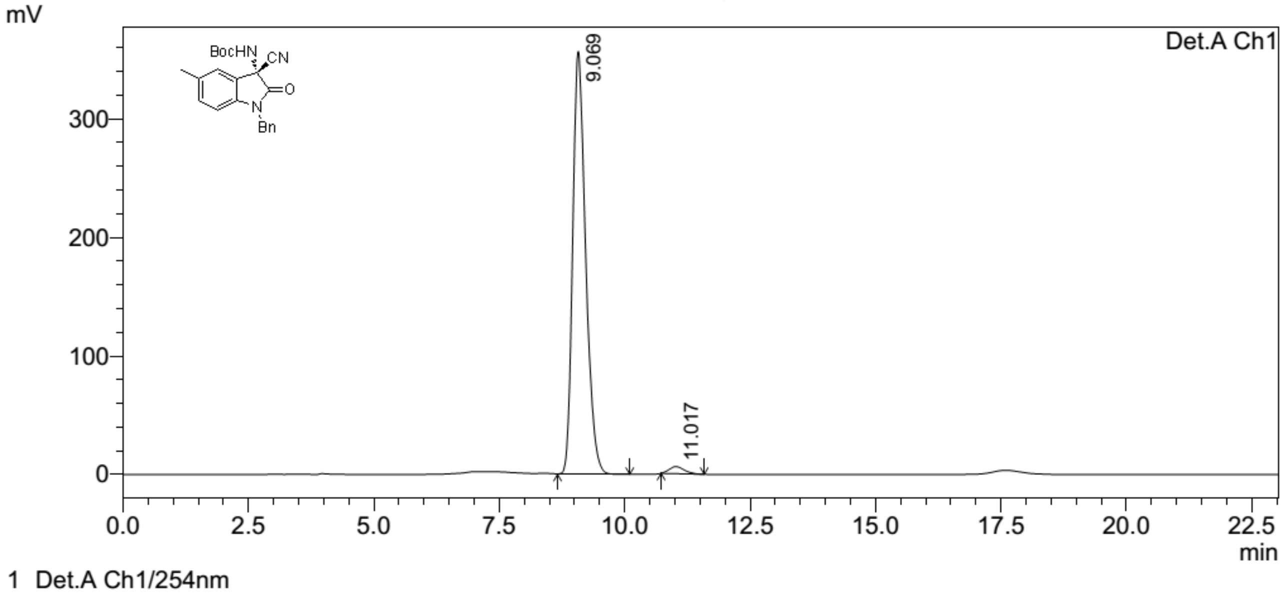

PeakTable

Detector A Ch1 254nm

Peak#	Ret. Time	Area	Height	Area %	Height %
1	9.069	6412198	357068	98.063	98.321
2	11.017	126688	6096	1.937	1.679
Total		6538887	363164	100.000	100.000

Supplementary Figure 47. HPLC spectra of products **2k**.

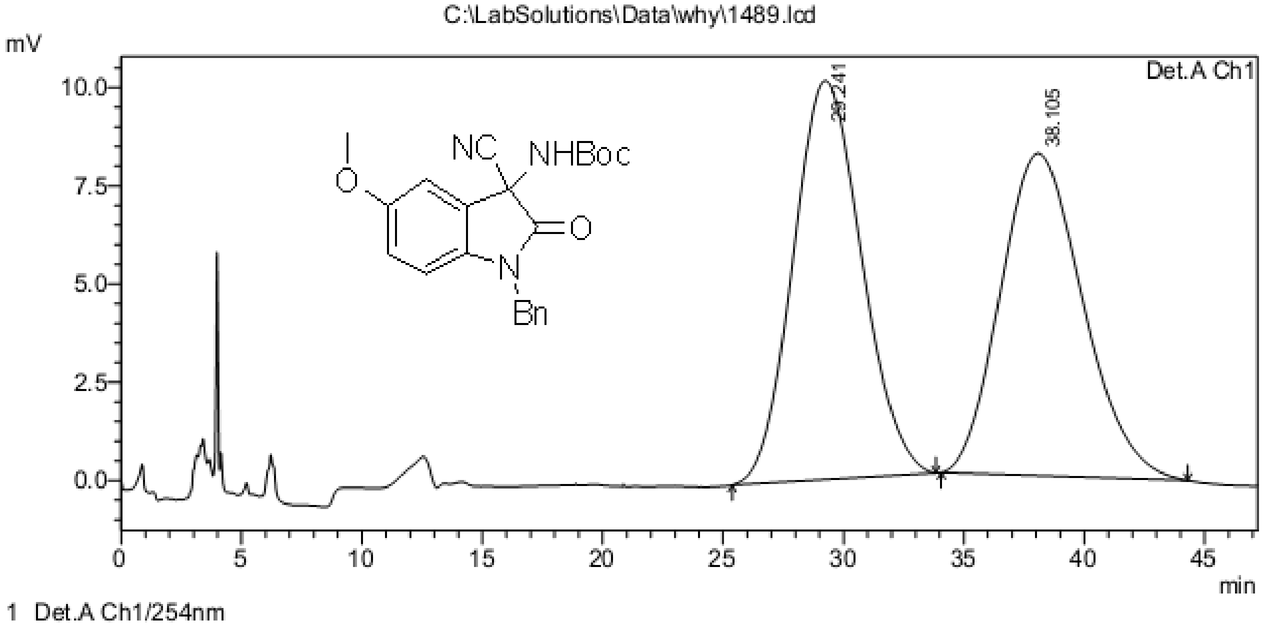

PeakTable

Detector A Ch1 254nm

Peak#	Ret. Time	Area	Height	Area %	Height %
1	29.241	2007555	10154	50.714	55.322
2	38.105	1951050	8200	49.286	44.678
Total		3958605	18354	100.000	100.000

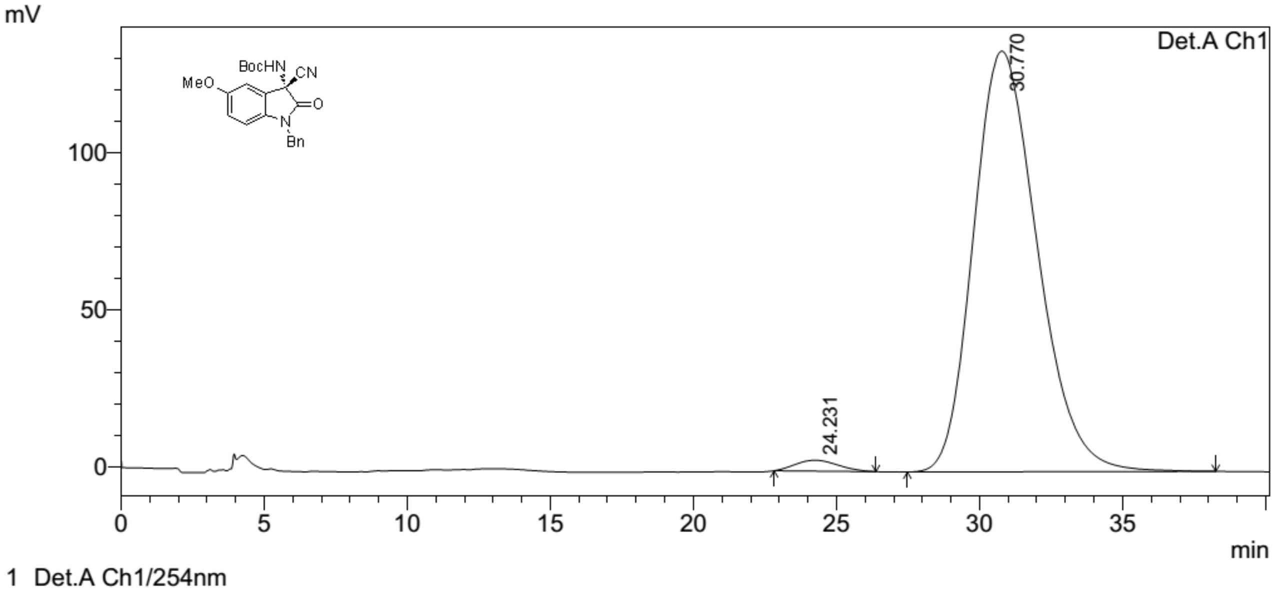

PeakTable

Detector A Ch1 254nm

Peak#	Ret. Time	Area	Height	Area %	Height %
1	24.231	356716	3454	1.686	2.516
2	30.770	20805538	133829	98.314	97.484
Total		21162254	137283	100.000	100.000

Supplementary Figure 48. HPLC spectra of products **2l**.

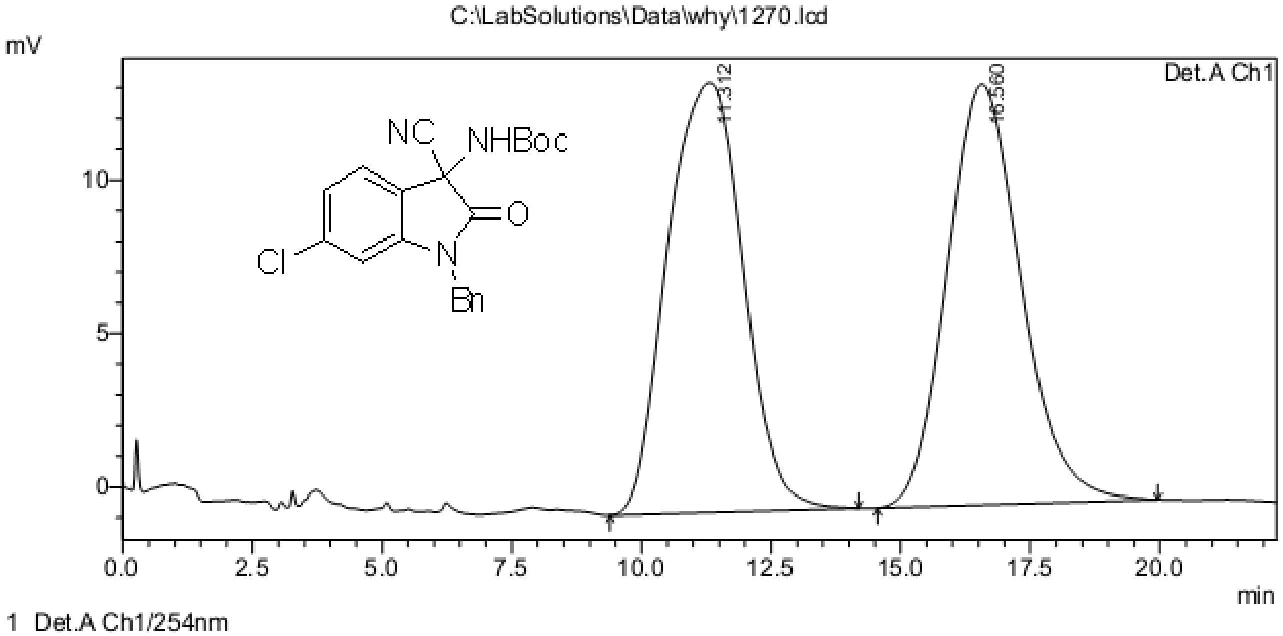

PeakTable

Detector A Ch1 254nm

Peak#	Ret. Time	Area	Height	Area %	Height %
1	11.312	1423212	13998	51.055	50.533
2	16.560	1364413	13703	48.945	49.467
Total		2787625	27701	100.000	100.000

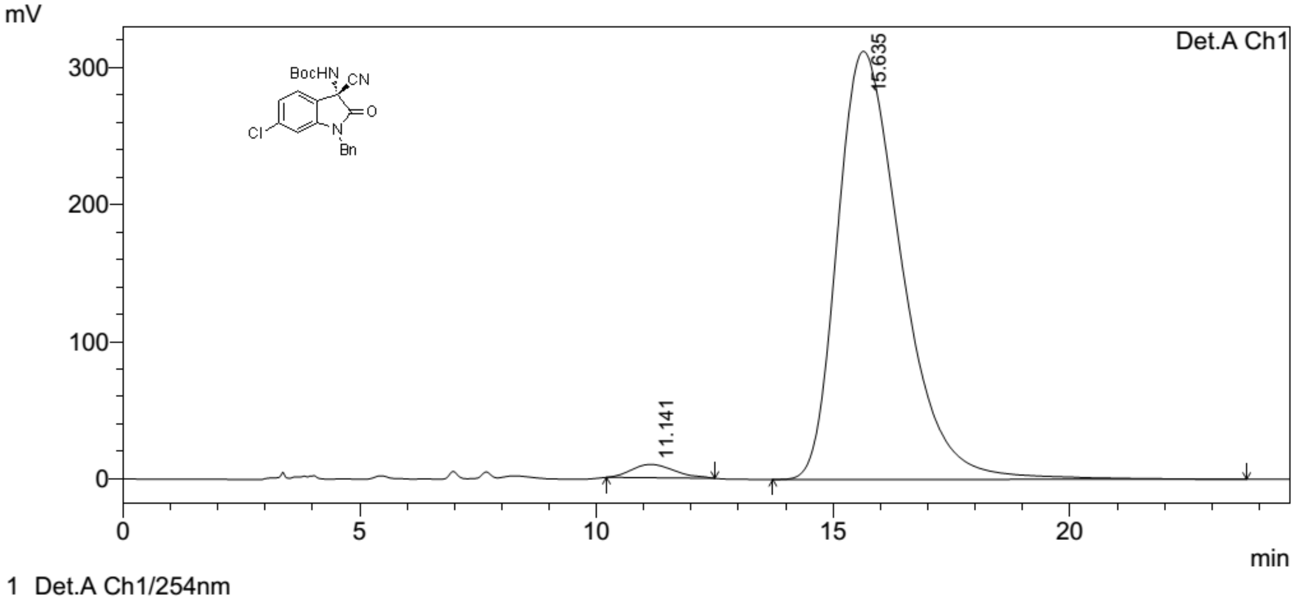

PeakTable

Detector A Ch1 254nm

Peak#	Ret. Time	Area	Height	Area %	Height %
1	11.141	614056	9767	2.045	3.031
2	15.635	29414932	312454	97.955	96.969
Total		30028988	322221	100.000	100.000

Supplementary Figure 49. HPLC spectra of products **2m**.

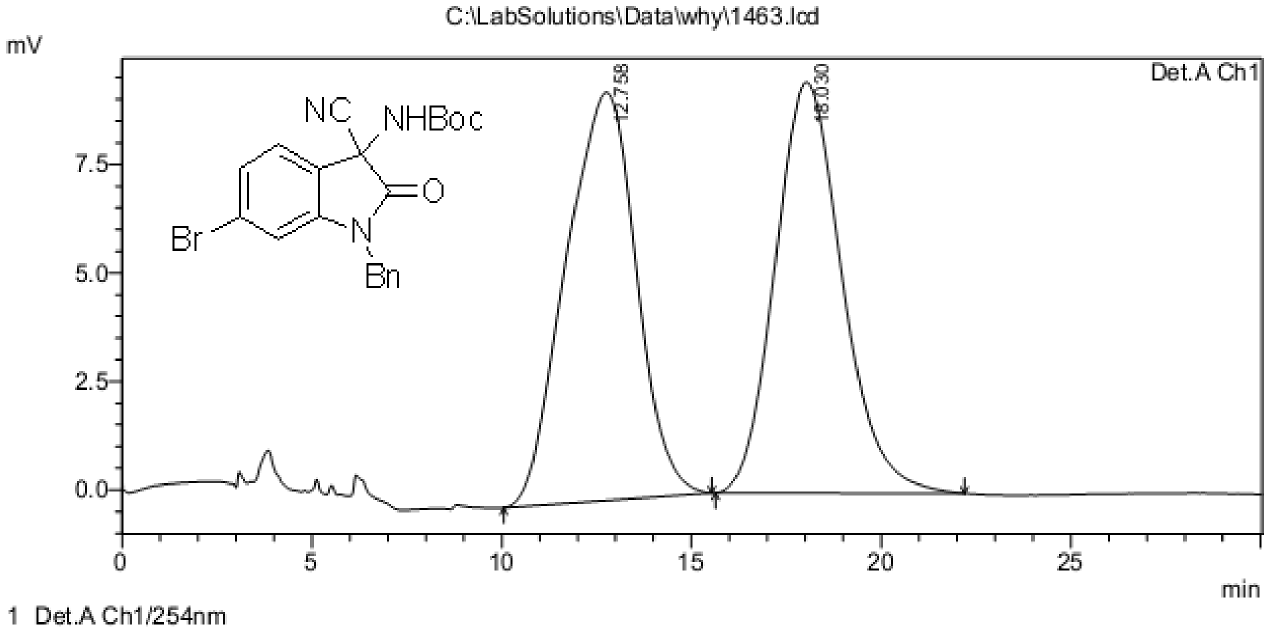

PeakTable

Detector A Ch1 254nm

Peak#	Ret. Time	Area	Height	Area %	Height %
1	12.758	1214112	9401	51.102	49.829
2	18.030	1161730	9465	48.898	50.171
Total		2375842	18866	100.000	100.000

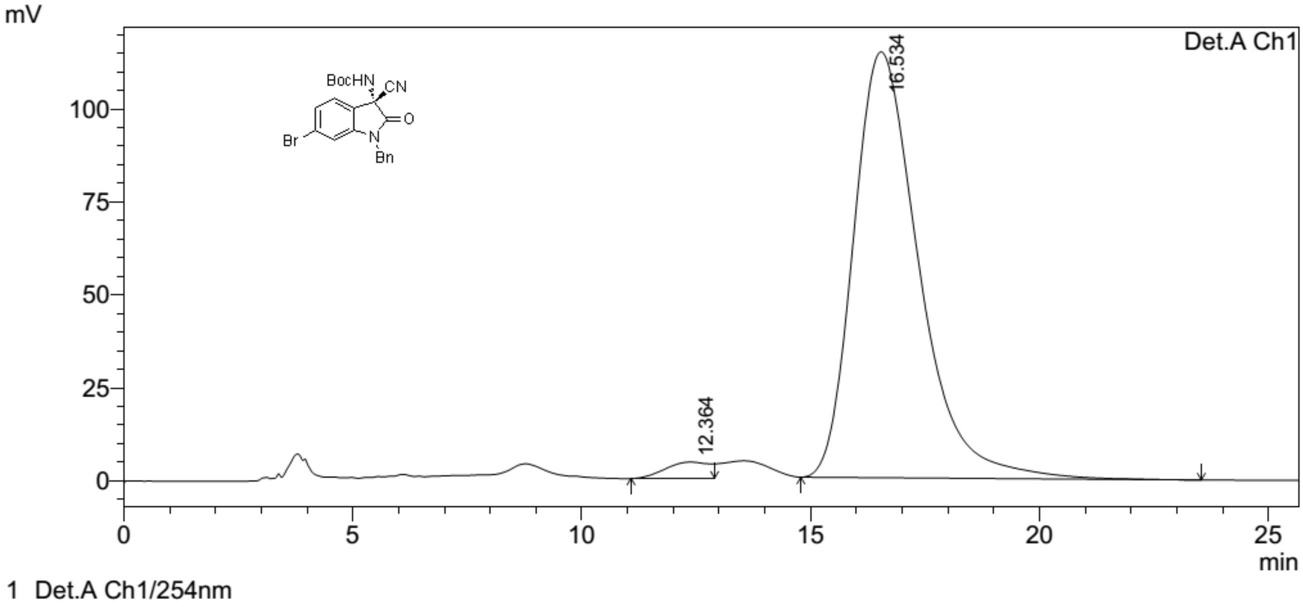

PeakTable

Detector A Ch1 254nm

Peak#	Ret. Time	Area	Height	Area %	Height %
1	12.364	288787	4334	2.480	3.648
2	16.534	11354338	114479	97.520	96.352
Total		11643125	118813	100.000	100.000

Supplementary Figure 50. HPLC spectra of products **2n**.

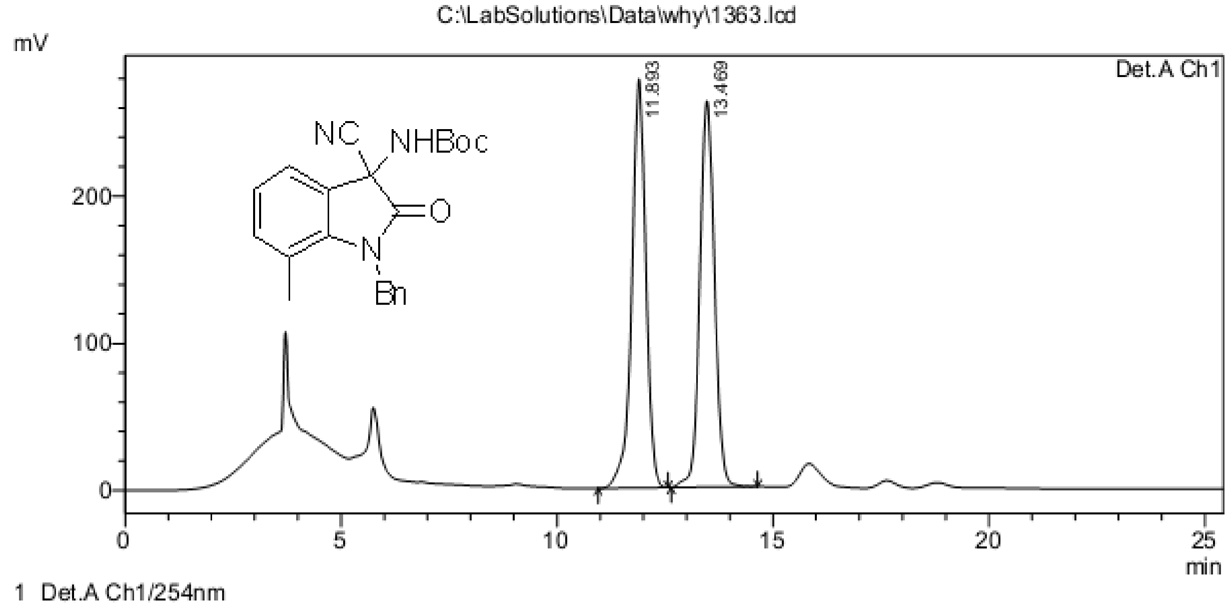

PeakTable

Detector A Ch1 254nm

Peak#	Ret. Time	Area	Height	Area %	Height %
1	11.893	6368773	278105	50.795	51.458
2	13.469	6169377	262349	49.205	48.542
Total		12538150	540455	100.000	100.000

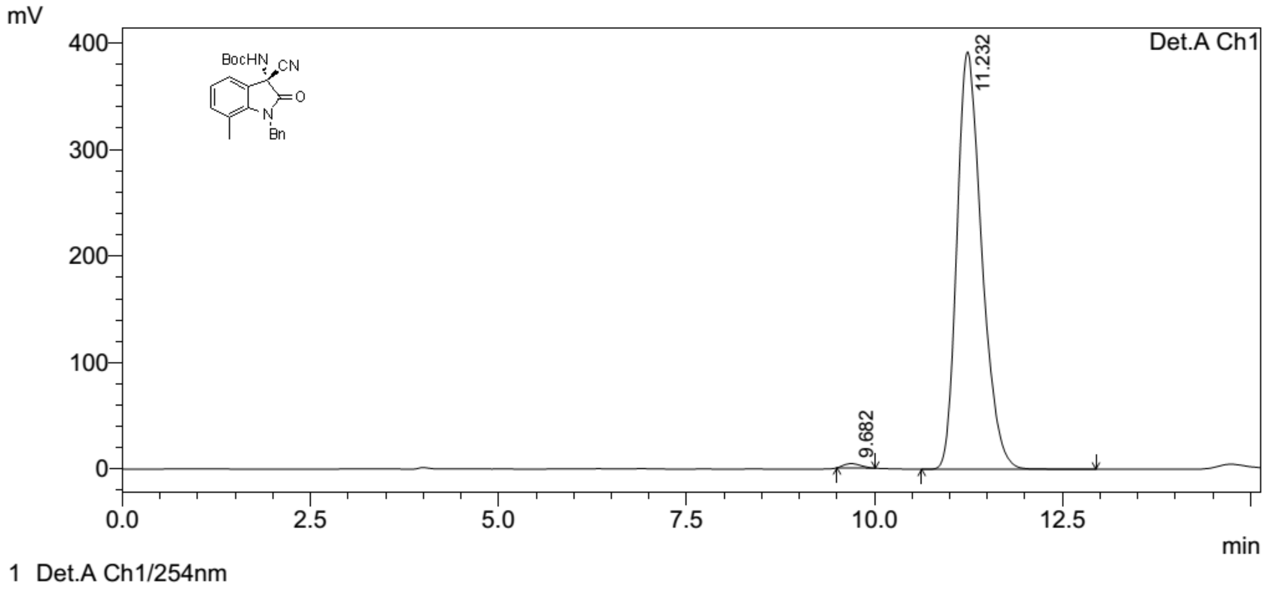

PeakTable

Detector A Ch1 254nm

Peak#	Ret. Time	Area	Height	Area %	Height %
1	9.682	64350	4112	0.711	1.039
2	11.232	8986014	391754	99.289	98.961
Total		9050365	395866	100.000	100.000

Supplementary Figure 51. HPLC spectra of products **2o**.

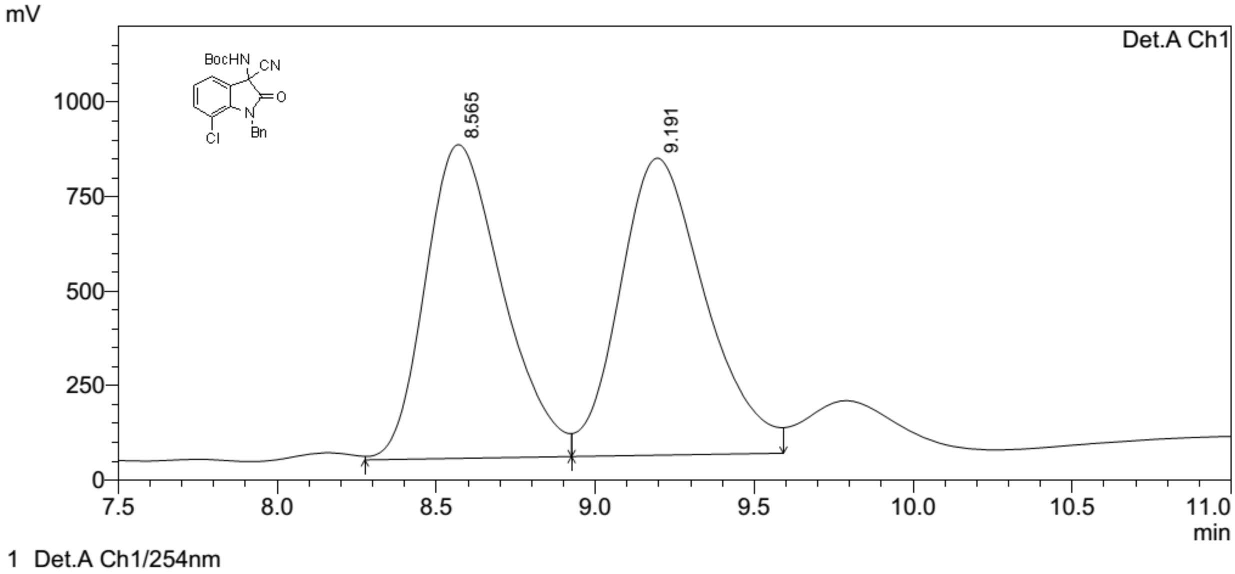

PeakTable

Detector A Ch1 254nm

Peak#	Ret. Time	Area	Height	Area %	Height %
1	8.565	14078422	830147	48.768	51.369
2	9.191	14789940	785908	51.232	48.631
Total		28868362	1616055	100.000	100.000

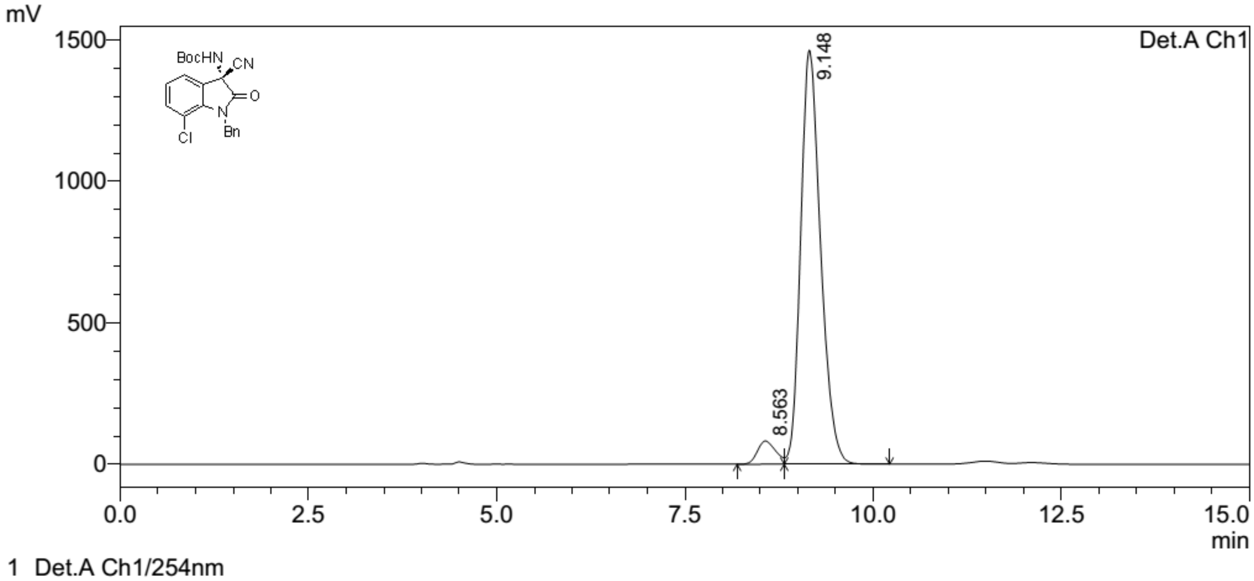

PeakTable

Detector A Ch1 254nm

Peak#	Ret. Time	Area	Height	Area %	Height %
1	8.563	1387523	82222	4.902	5.319
2	9.148	26918528	1463637	95.098	94.681
Total		28306051	1545858	100.000	100.000

Supplementary Figure 52. HPLC spectra of products **2p**.

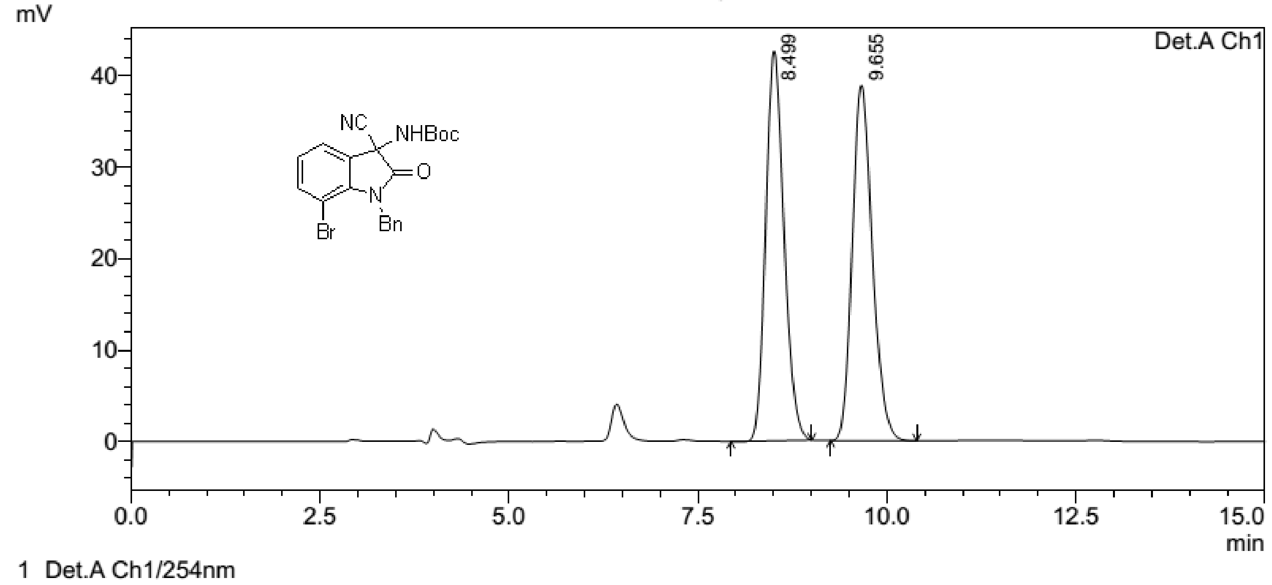

PeakTable

Detector A Ch1 254nm

Peak#	Ret. Time	Area	Height	Area %	Height %
1	8.499	717688	42592	49.981	52.285
2	9.655	718224	38868	50.019	47.715
Total		1435912	81460	100.000	100.000

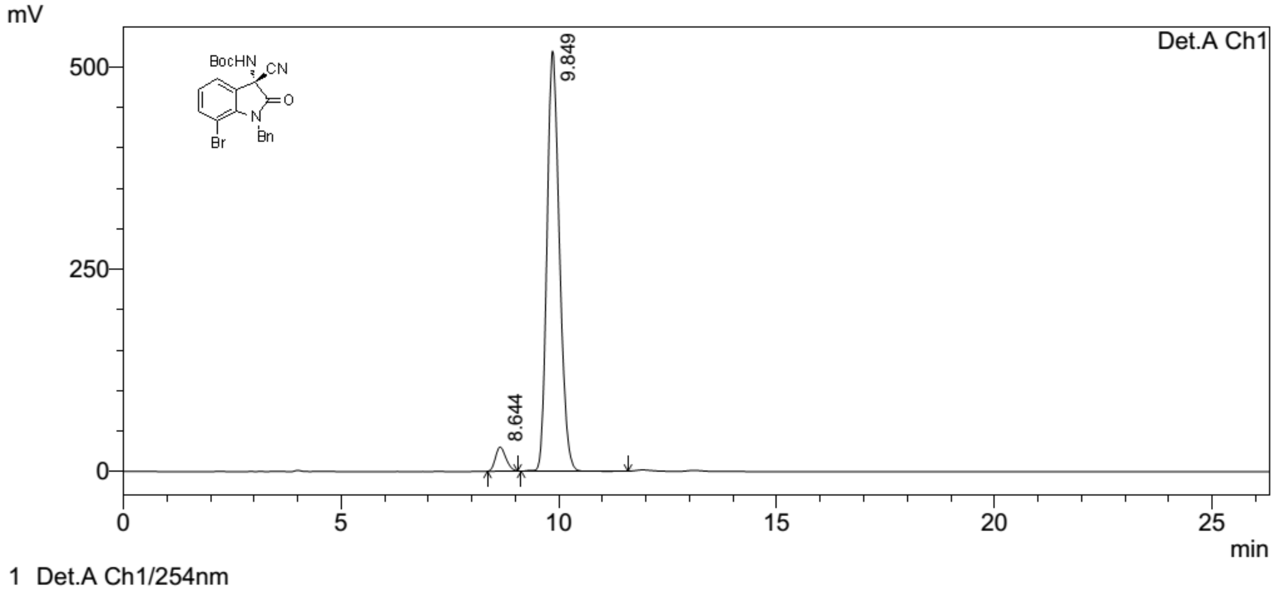

PeakTable

Detector A Ch1 254nm

Peak#	Ret. Time	Area	Height	Area %	Height %
1	8.644	496774	29848	4.601	5.433
2	9.849	10301170	519520	95.399	94.567
Total		10797944	549368	100.000	100.000

Supplementary Figure 53. HPLC spectra of products **5a**.

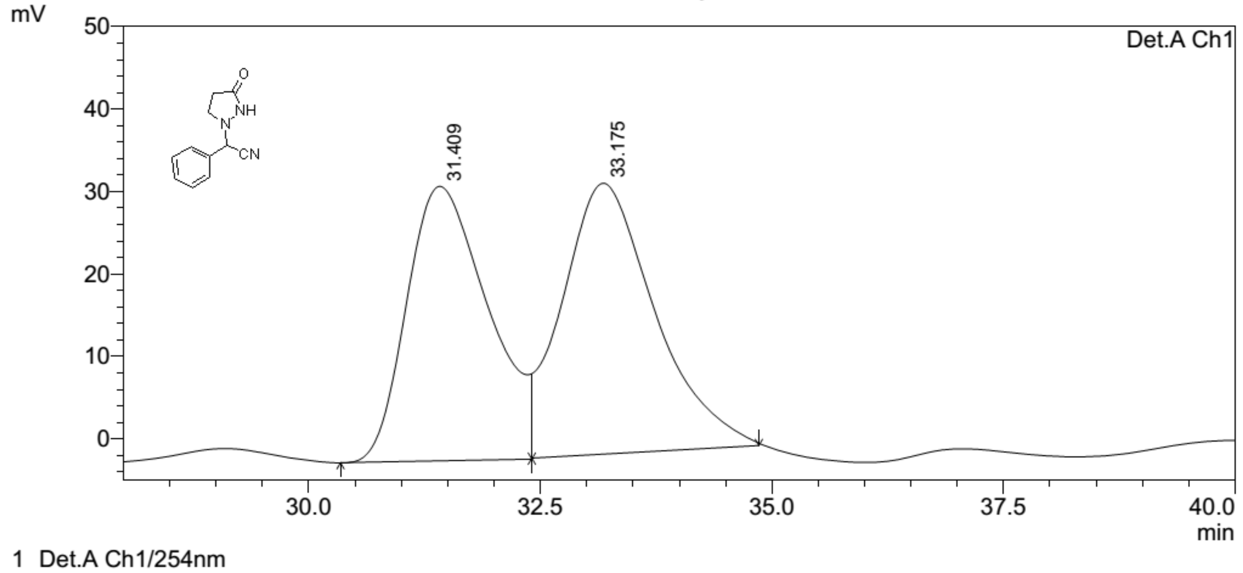

PeakTable

Detector A Ch1 254nm

Peak#	Ret. Time	Area	Height	Area %	Height %
1	31.409	2038225	33292	47.191	50.327
2	33.175	2280885	32859	52.809	49.673
Total		4319110	66151	100.000	100.000

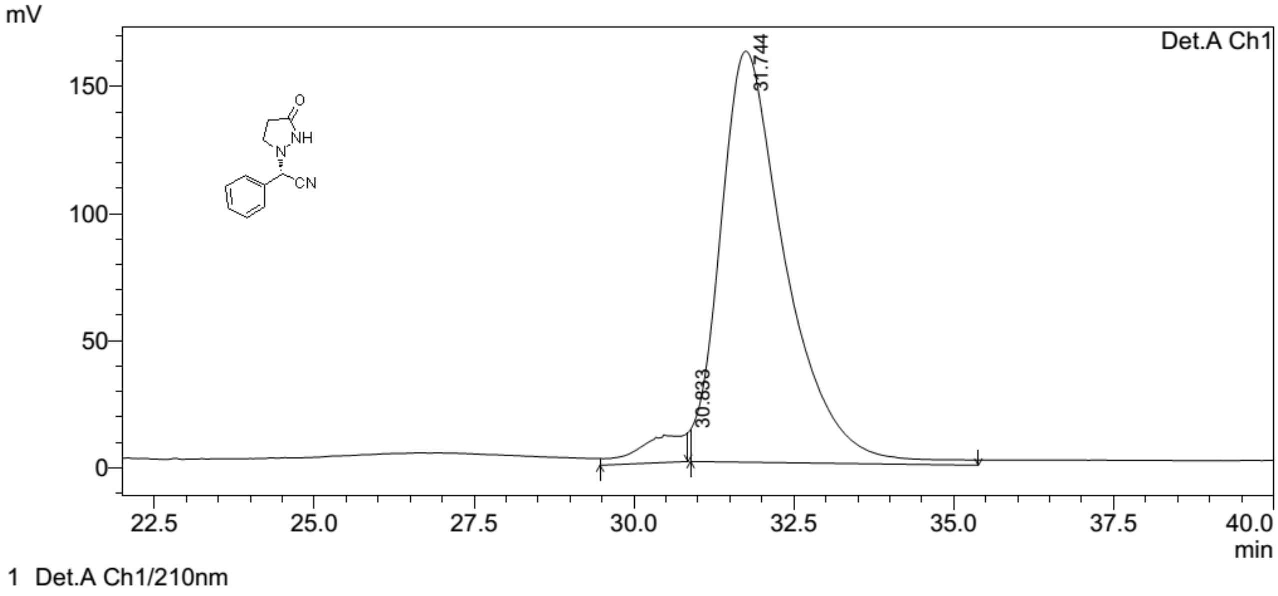

PeakTable

Detector A Ch1 210nm

Peak#	Ret. Time	Area	Height	Area %	Height %
1	30.833	559155	11050	4.623	6.395
2	31.744	11534983	161737	95.377	93.605
Total		12094138	172787	100.000	100.000

Supplementary Figure 54. HPLC spectra of products **5b**.

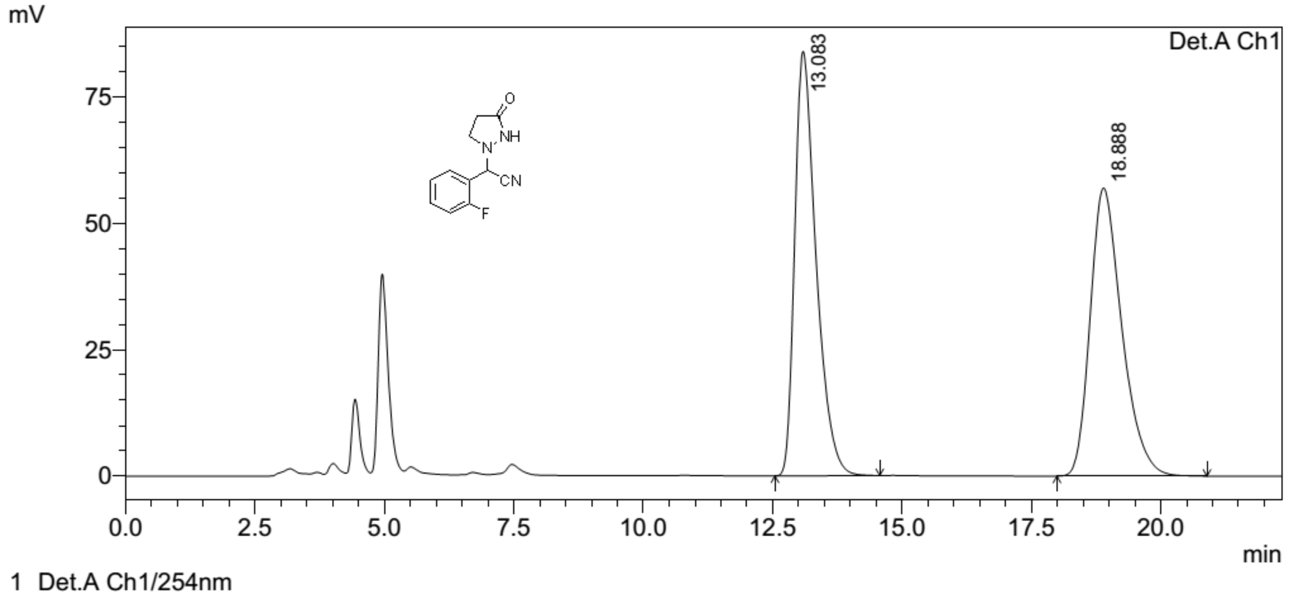

PeakTable

Detector A Ch1 254nm

Peak#	Ret. Time	Area	Height	Area %	Height %
1	13.083	2408687	83833	49.890	59.551
2	18.888	2419269	56942	50.110	40.449
Total		4827956	140775	100.000	100.000

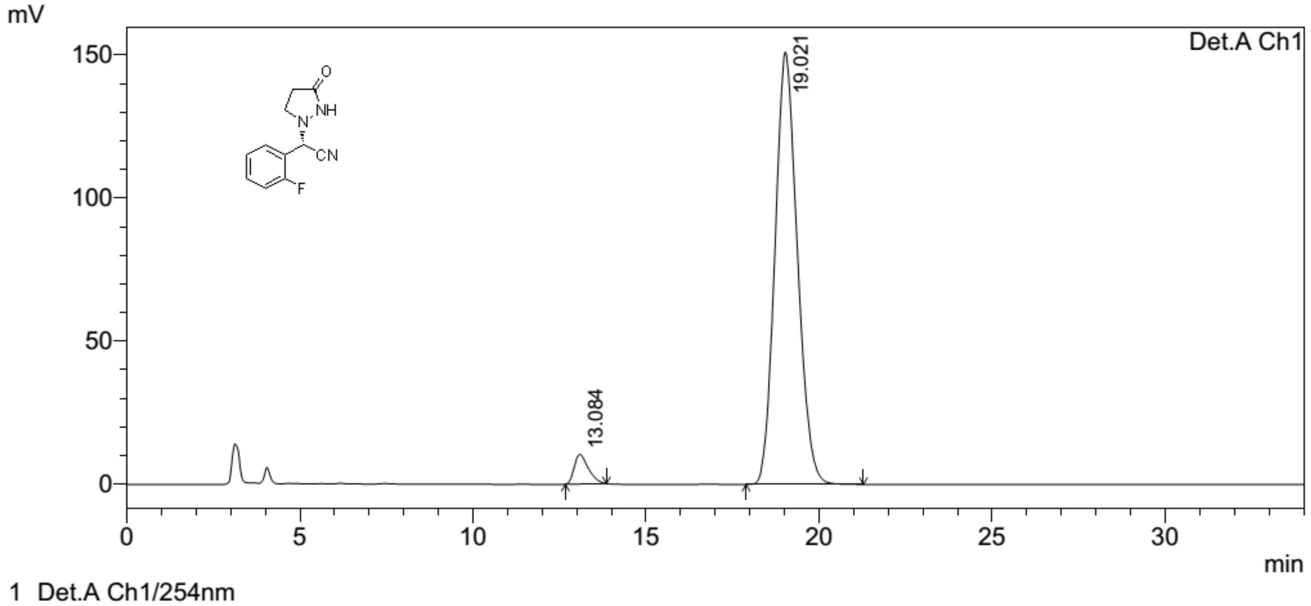

PeakTable

Detector A Ch1 254nm

Peak#	Ret. Time	Area	Height	Area %	Height %
1	13.084	294190	10369	4.158	6.423
2	19.021	6781784	151068	95.842	93.577
Total		7075974	161437	100.000	100.000

Supplementary Figure 55. HPLC spectra of products **5c**.

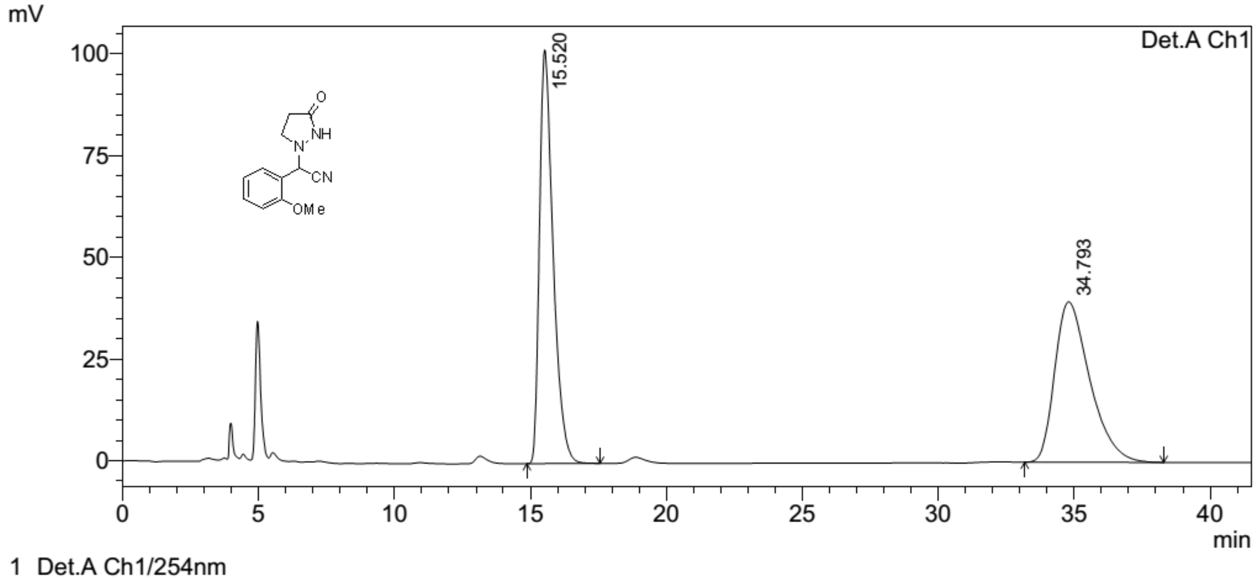

PeakTable

Detector A Ch1 254nm

Peak#	Ret. Time	Area	Height	Area %	Height %
1	15.520	3540771	101665	50.054	72.057
2	34.793	3533103	39426	49.946	27.943
Total		7073875	141091	100.000	100.000

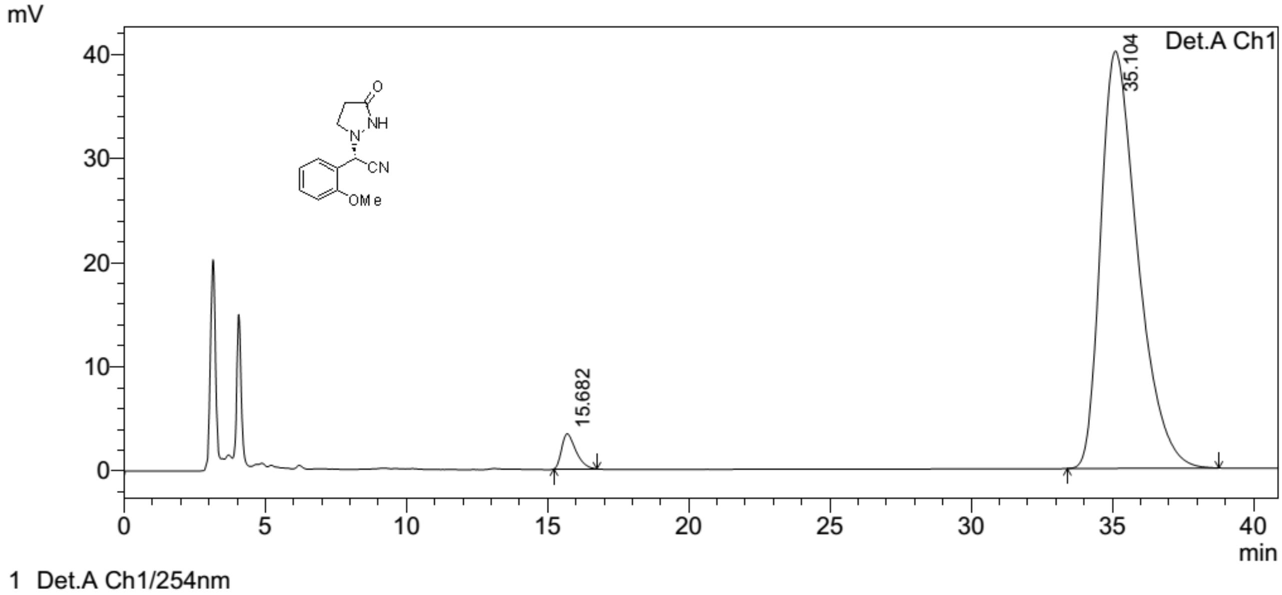

PeakTable

Detector A Ch1 254nm

Peak#	Ret. Time	Area	Height	Area %	Height %
1	15.682	117804	3373	3.129	7.771
2	35.104	3646703	40032	96.871	92.229
Total		3764507	43405	100.000	100.000

Supplementary Figure 56. HPLC spectra of products **5d**.

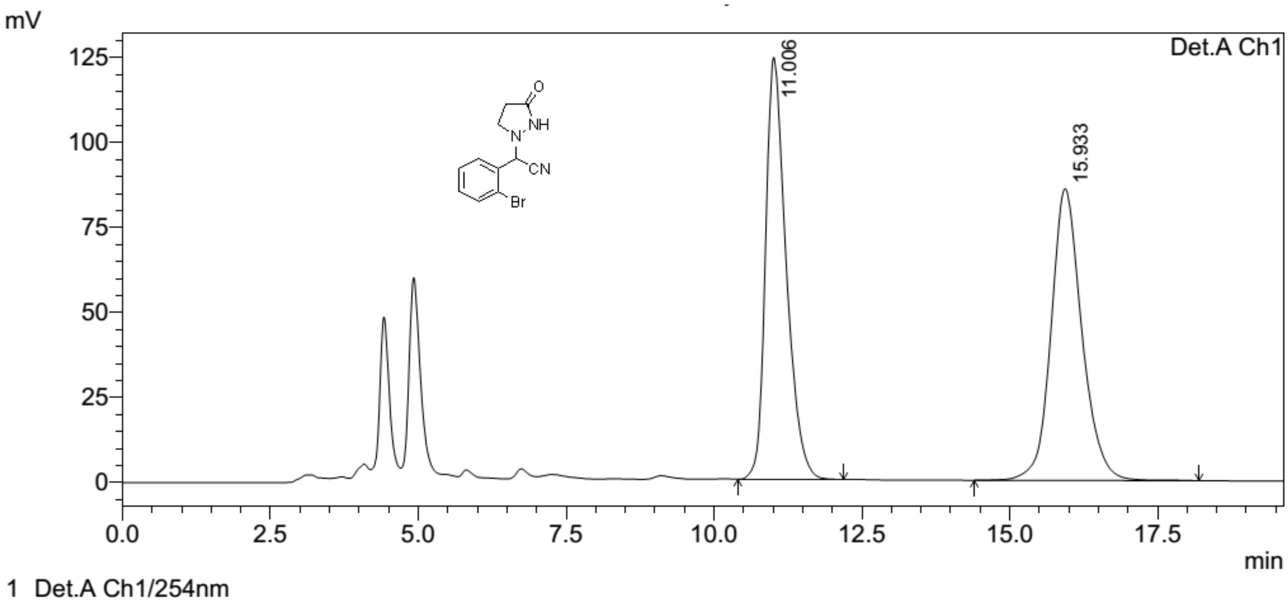

PeakTable

Detector A Ch1 254nm

Peak#	Ret. Time	Area	Height	Area %	Height %
1	11.006	3002035	124197	49.098	59.129
2	15.933	3112297	85847	50.902	40.871
Total		6114332	210044	100.000	100.000

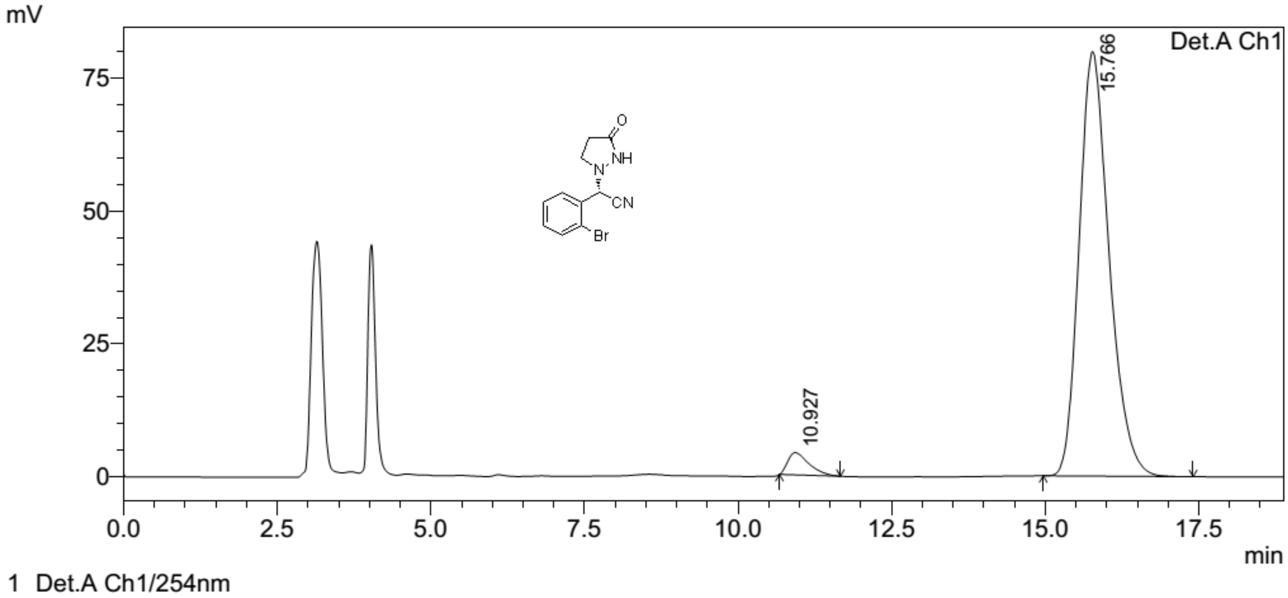

PeakTable

Detector A Ch1 254nm

Peak#	Ret. Time	Area	Height	Area %	Height %
1	10.927	100686	4209	3.586	5.004
2	15.766	2707410	79919	96.414	94.996
Total		2808097	84129	100.000	100.000

Supplementary Figure 57. HPLC spectra of products **5e**.

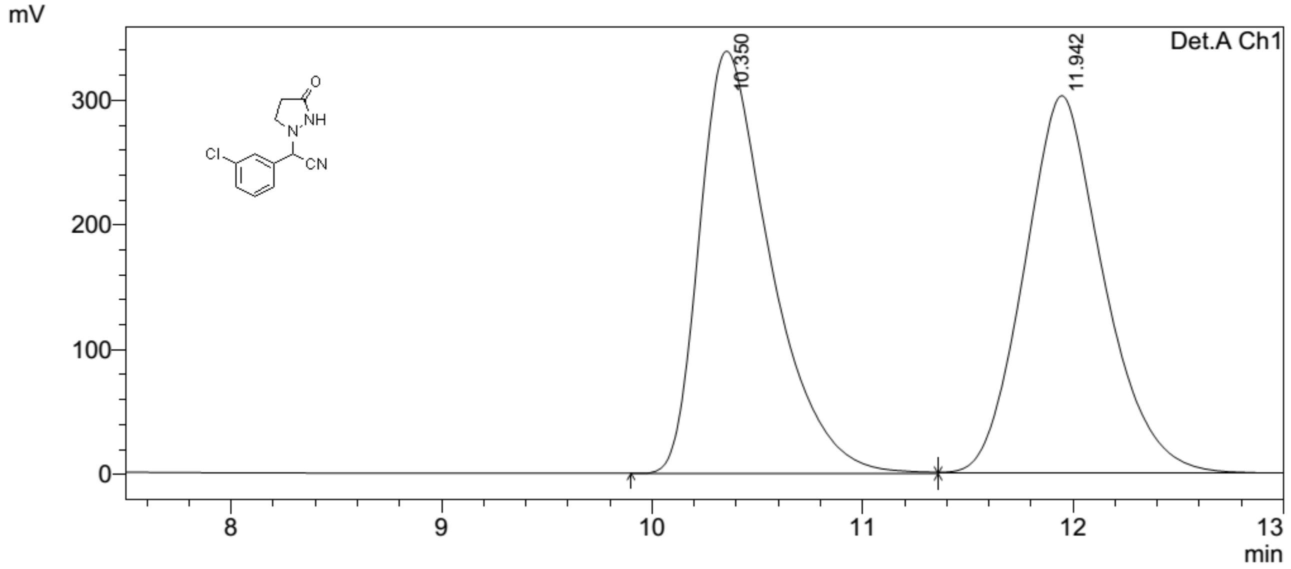

1 Det.A Ch1/254nm

PeakTable

Detector A Ch1 254nm

Peak#	Ret. Time	Area	Height	Area %	Height %
1	10.350	7938499	338398	49.917	52.778
2	11.942	7964977	302775	50.083	47.222
Total		15903477	641173	100.000	100.000

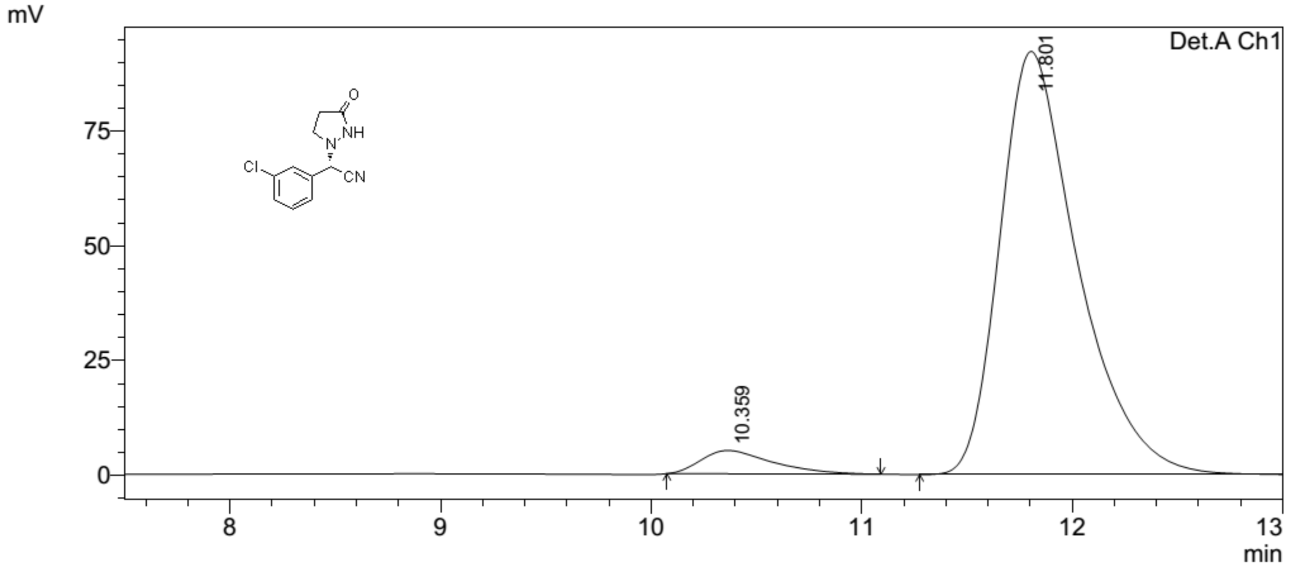

1 Det.A Ch1/254nm

PeakTable

Detector A Ch1 254nm

Peak#	Ret. Time	Area	Height	Area %	Height %
1	10.359	118905	5096	4.772	5.236
2	11.801	2373022	92226	95.228	94.764
Total		2491926	97321	100.000	100.000

Supplementary Figure 58. HPLC spectra of products **5f**.

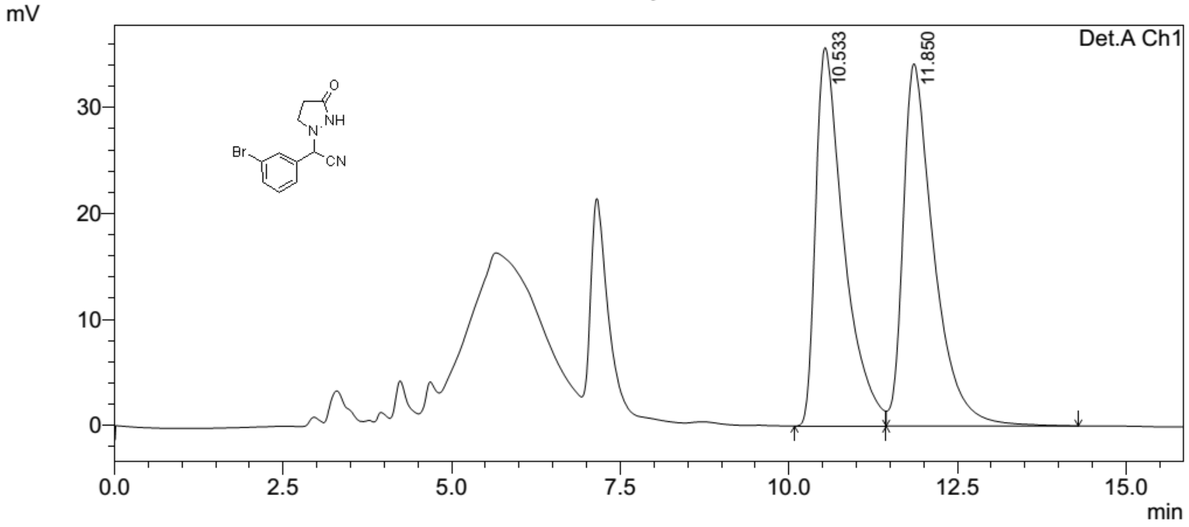

PeakTable

Detector A Ch1 254nm

Peak#	Ret. Time	Area	Height	Area %	Height %
1	10.533	1040915	35720	48.916	51.092
2	11.850	1087066	34193	51.084	48.908
Total		2127980	69913	100.000	100.000

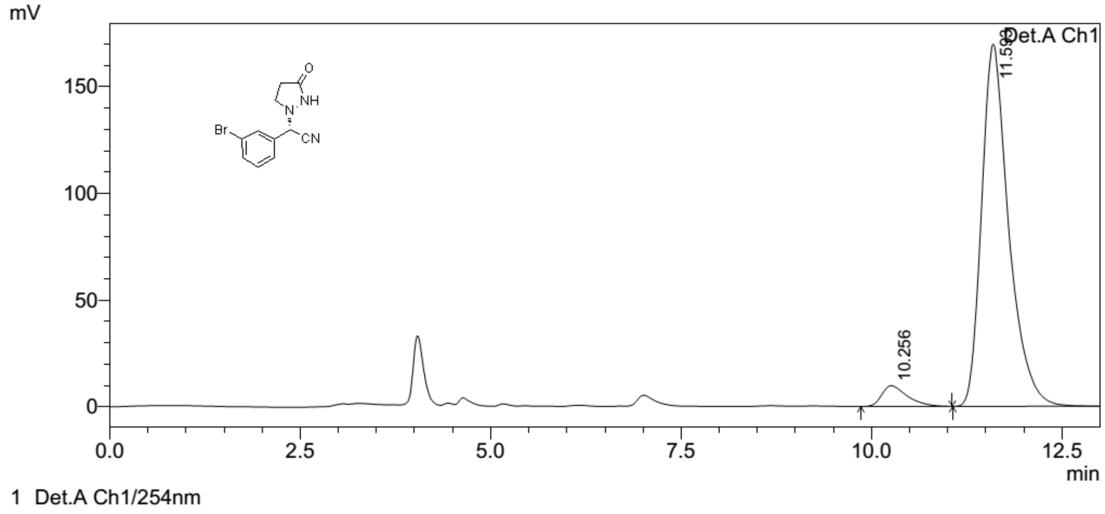

PeakTable

Detector A Ch1 254nm

Peak#	Ret. Time	Area	Height	Area %	Height %
1	10.256	225694	9783	5.093	5.448
2	11.593	4205371	169804	94.907	94.552
Total		4431066	179587	100.000	100.000

Supplementary Figure 59. HPLC spectra of products **5g**.

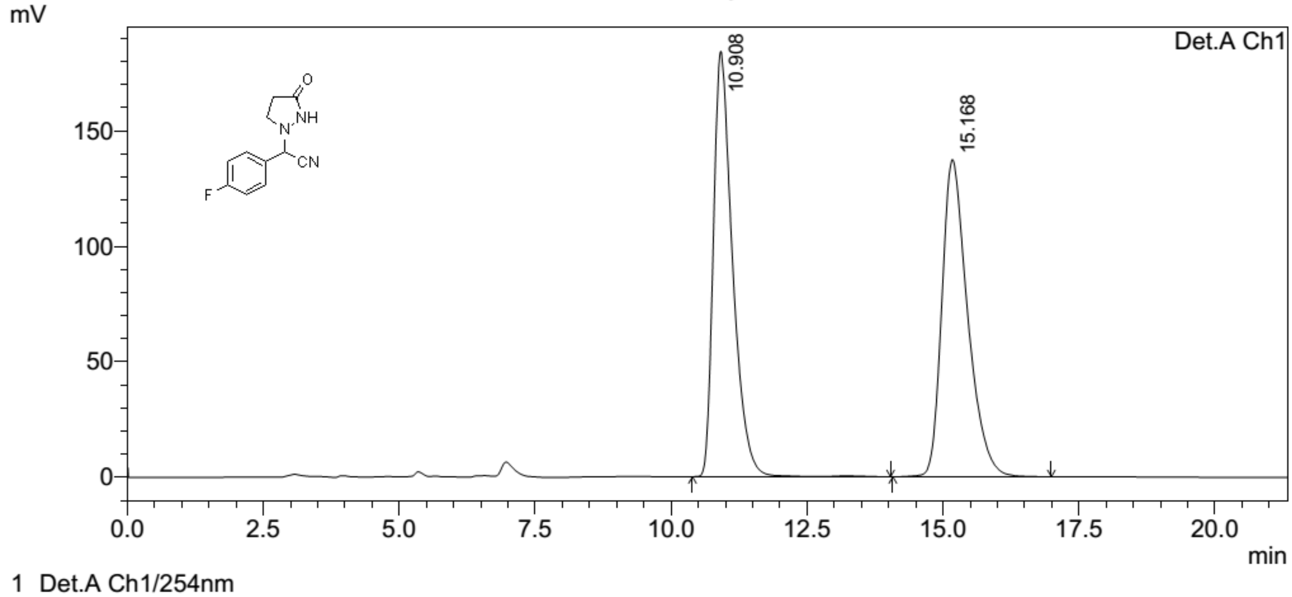

PeakTable

Detector A Ch1 254nm

Peak#	Ret. Time	Area	Height	Area %	Height %
1	10.908	4504341	184089	49.868	57.278
2	15.168	4528204	137307	50.132	42.722
Total		9032544	321396	100.000	100.000

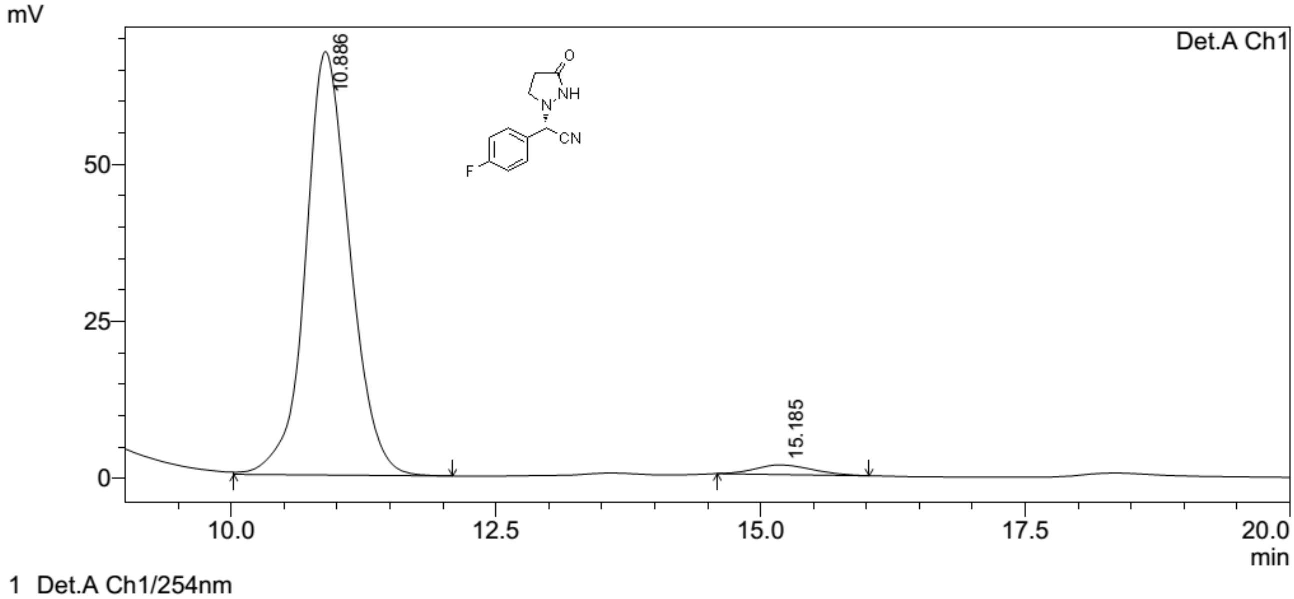

PeakTable

Detector A Ch1 254nm

Peak#	Ret. Time	Area	Height	Area %	Height %
1	10.886	1967335	67384	97.177	97.777
2	15.185	57144	1532	2.823	2.223
Total		2024478	68916	100.000	100.000

Supplementary Figure 60. HPLC spectra of products **5h**.

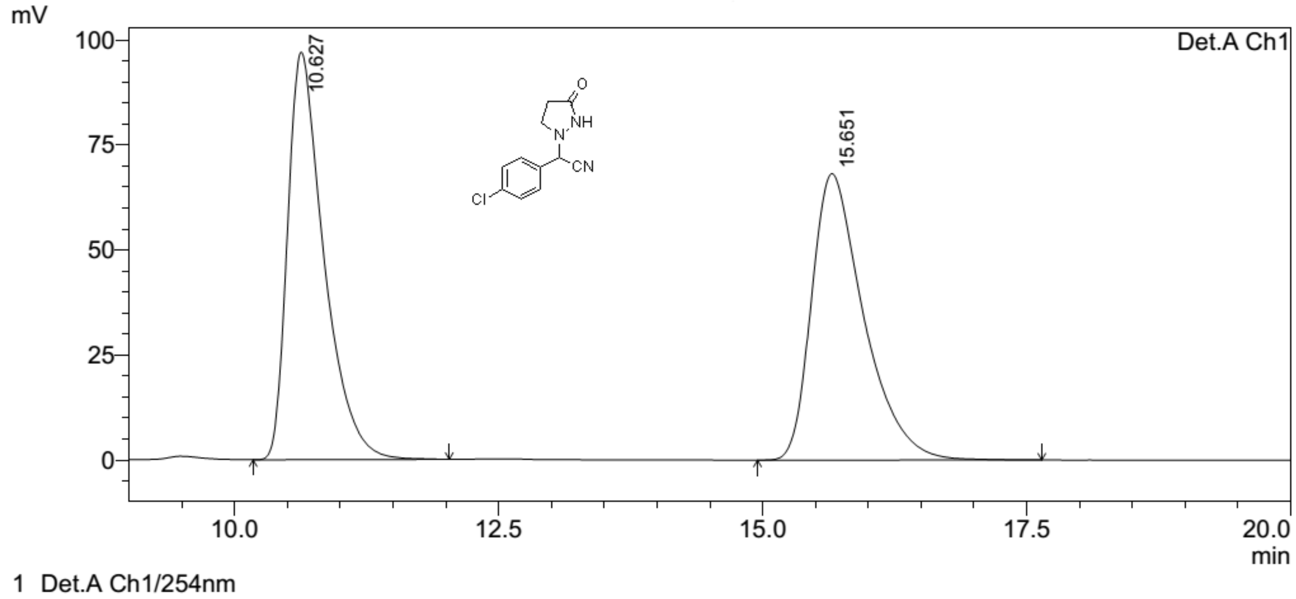

PeakTable

Detector A Ch1 254nm

Peak#	Ret. Time	Area	Height	Area %	Height %
1	10.627	2318401	96998	49.787	58.731
2	15.651	2338243	68158	50.213	41.269
Total		4656644	165156	100.000	100.000

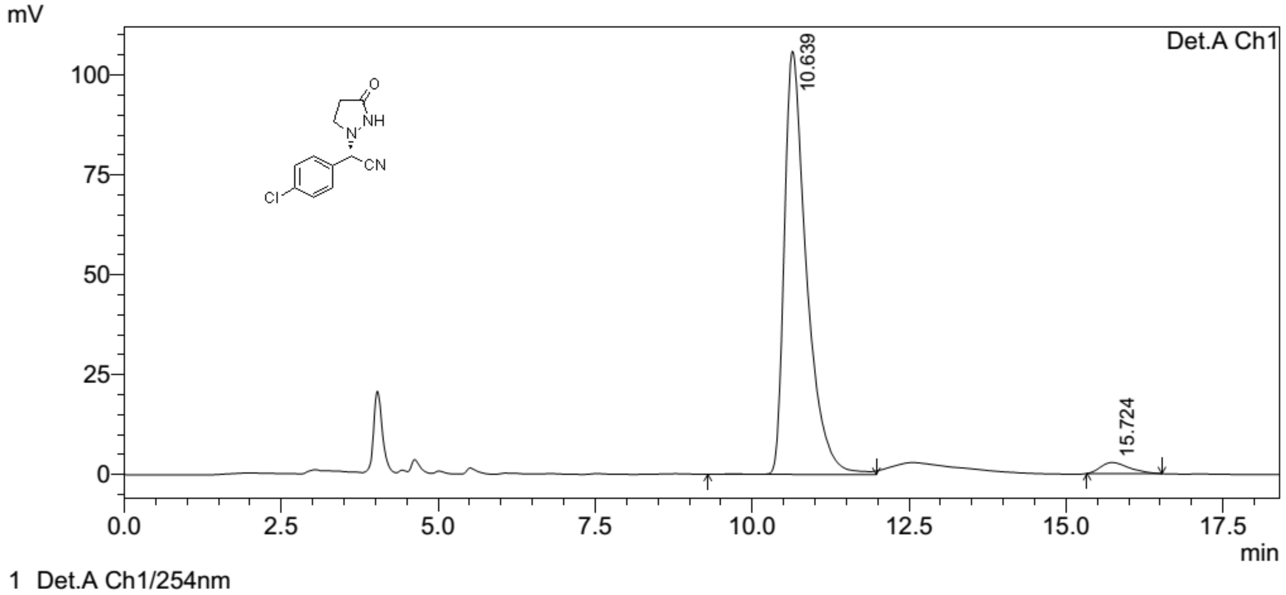

PeakTable

Detector A Ch1 254nm

Peak#	Ret. Time	Area	Height	Area %	Height %
1	10.639	2594901	105911	96.705	97.425
2	15.724	88414	2799	3.295	2.575
Total		2683316	108710	100.000	100.000

Supplementary Figure 61. HPLC spectra of products **5i**.

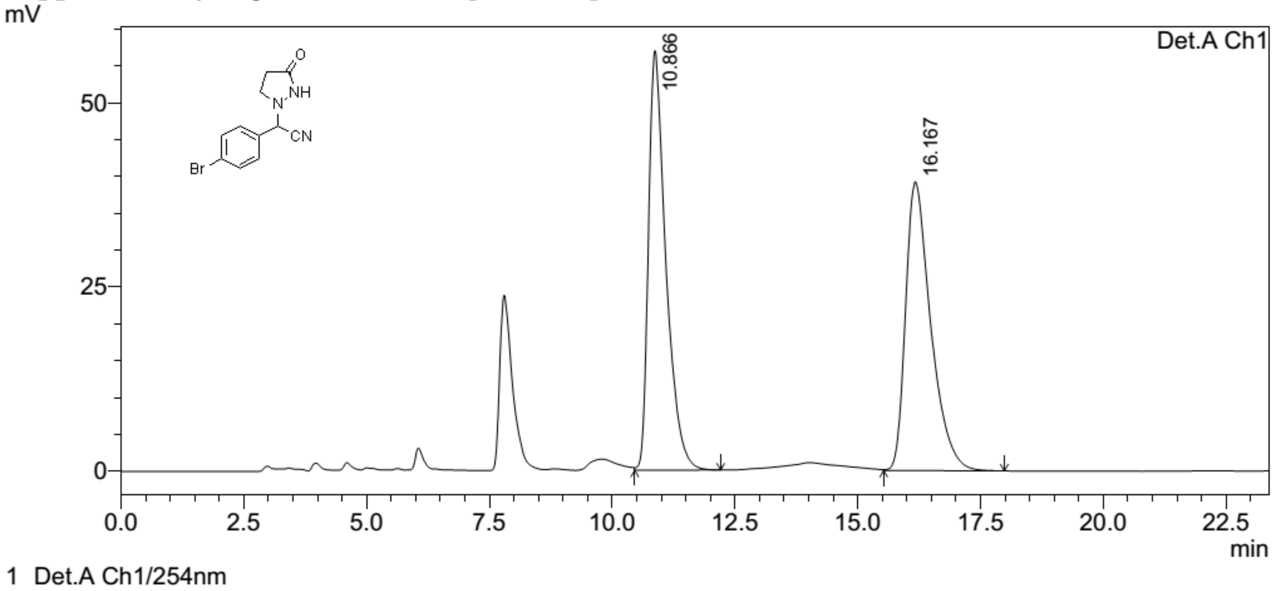

PeakTable

Detector A Ch1 254nm

Peak#	Ret. Time	Area	Height	Area %	Height %
1	10.866	1404180	56936	50.140	59.217
2	16.167	1396345	39212	49.860	40.783
Total		2800525	96148	100.000	100.000

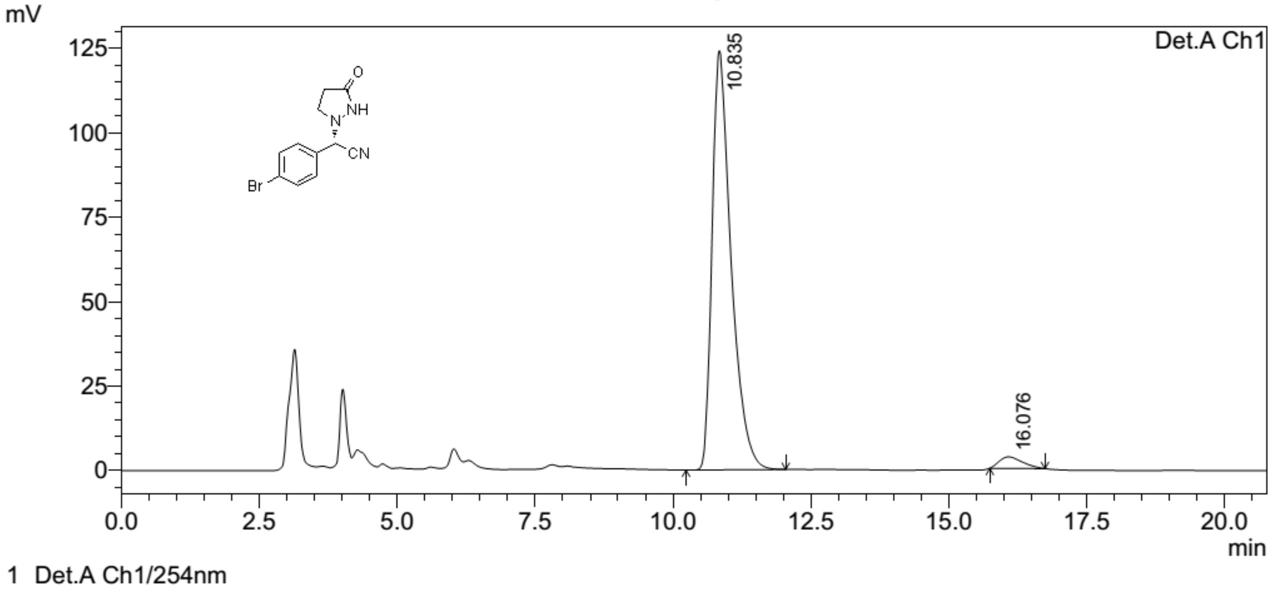

PeakTable

Detector A Ch1 254nm

Peak#	Ret. Time	Area	Height	Area %	Height %
1	10.835	2977195	124162	96.740	97.316
2	16.076	100322	3424	3.260	2.684
Total		3077517	127587	100.000	100.000

Supplementary Figure 62. HPLC spectra of products **5j**.

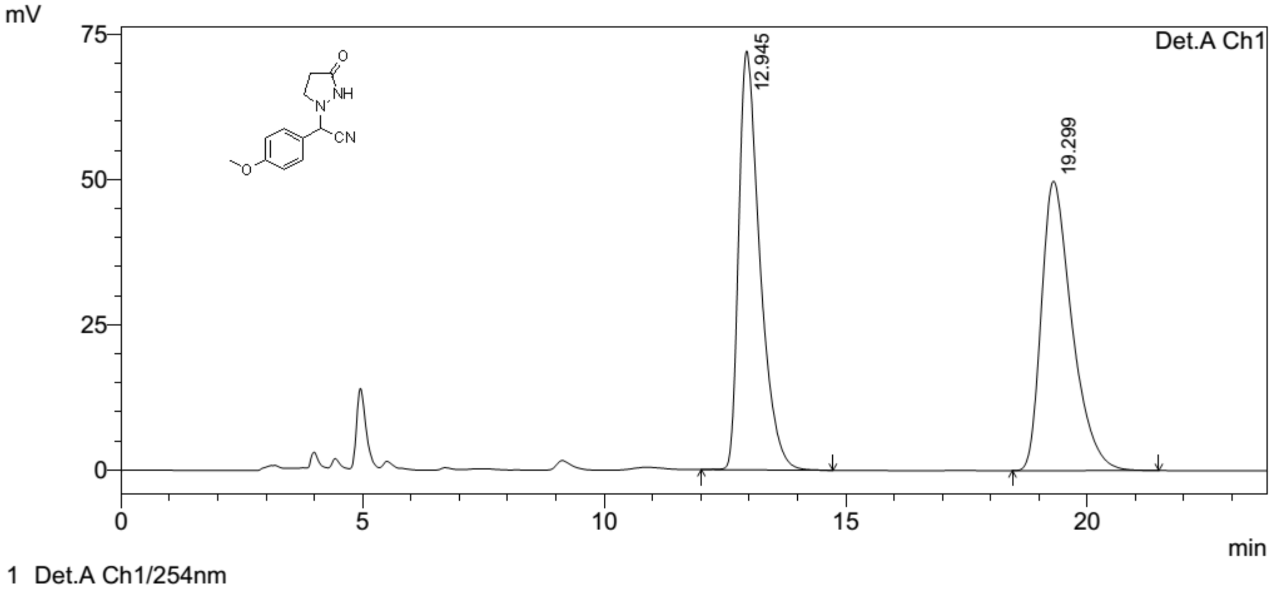

PeakTable

Detector A Ch1 254nm

Peak#	Ret. Time	Area	Height	Area %	Height %
1	12.945	2194786	72125	50.026	59.136
2	19.299	2192494	49840	49.974	40.864
Total		4387280	121965	100.000	100.000

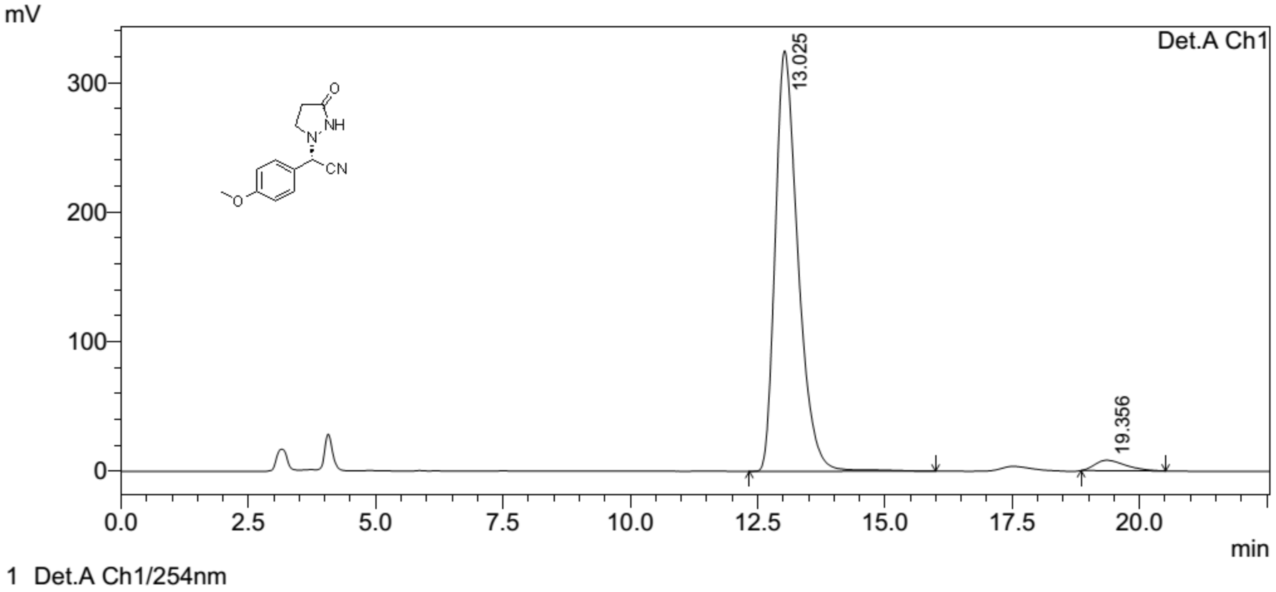

PeakTable

Detector A Ch1 254nm

Peak#	Ret. Time	Area	Height	Area %	Height %
1	13.025	10057265	324589	96.723	97.551
2	19.356	340713	8148	3.277	2.449
Total		10397979	332737	100.000	100.000

Supplementary Figure 63. HPLC spectra of products **5k**.

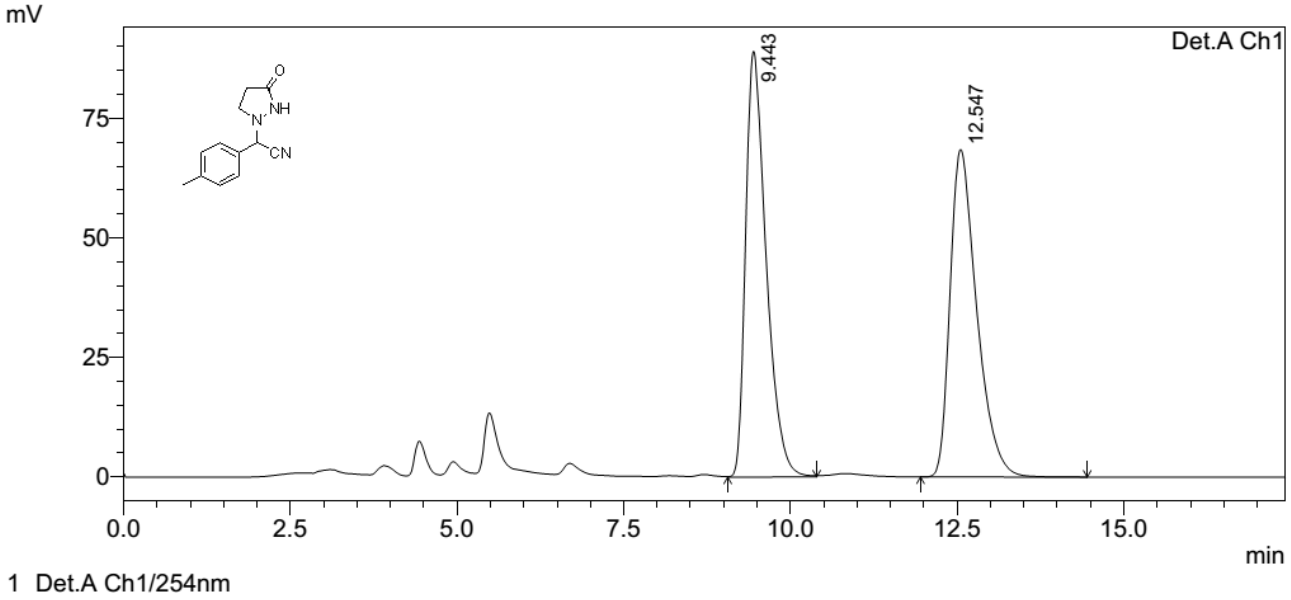

PeakTable

Detector A Ch1 254nm

Peak#	Ret. Time	Area	Height	Area %	Height %
1	9.443	1908399	89028	50.080	56.519
2	12.547	1902277	68491	49.920	43.481
Total		3810676	157519	100.000	100.000

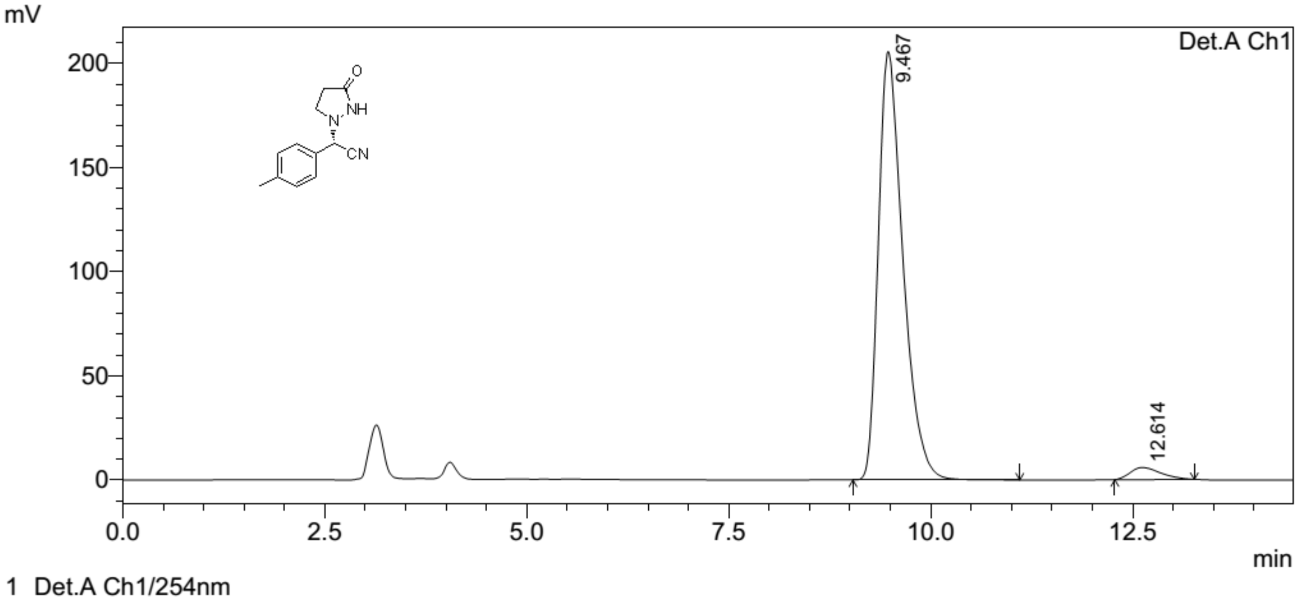

PeakTable

Detector A Ch1 254nm

Peak#	Ret. Time	Area	Height	Area %	Height %
1	9.467	4363971	205536	96.628	97.240
2	12.614	152273	5833	3.372	2.760
Total		4516244	211369	100.000	100.000

Supplementary Figure 64. HPLC spectra of products **5l**.

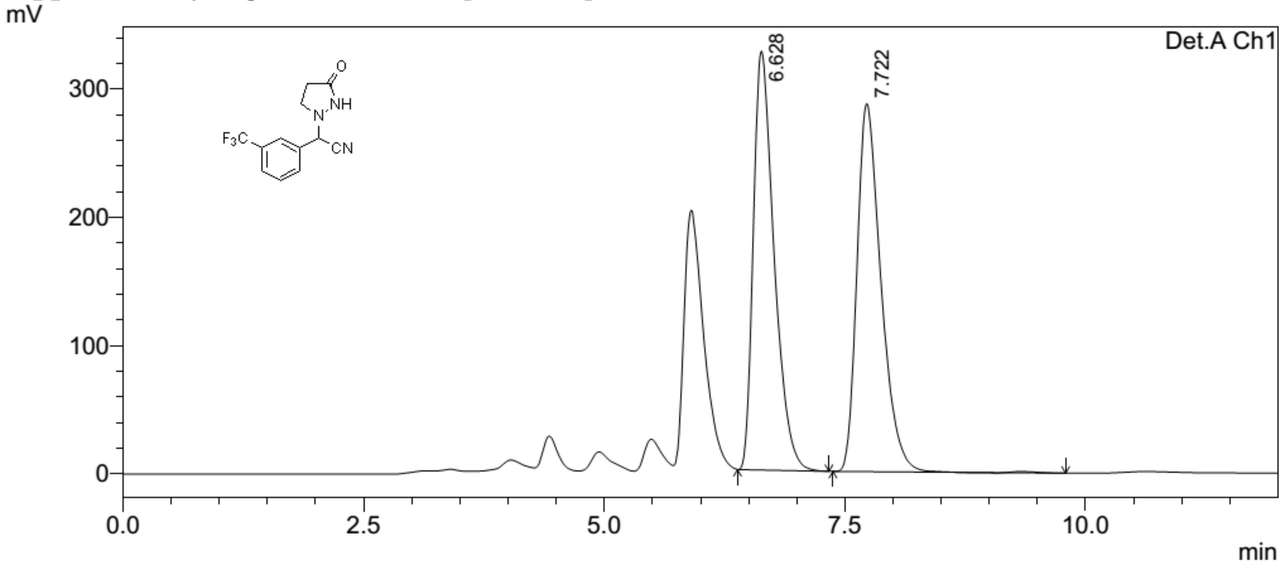

1 Det.A Ch1/254nm

PeakTable

Detector A Ch1 254nm

Peak#	Ret. Time	Area	Height	Area %	Height %
1	6.628	4901271	326819	49.740	53.239
2	7.722	4952558	287052	50.260	46.761
Total		9853829	613871	100.000	100.000

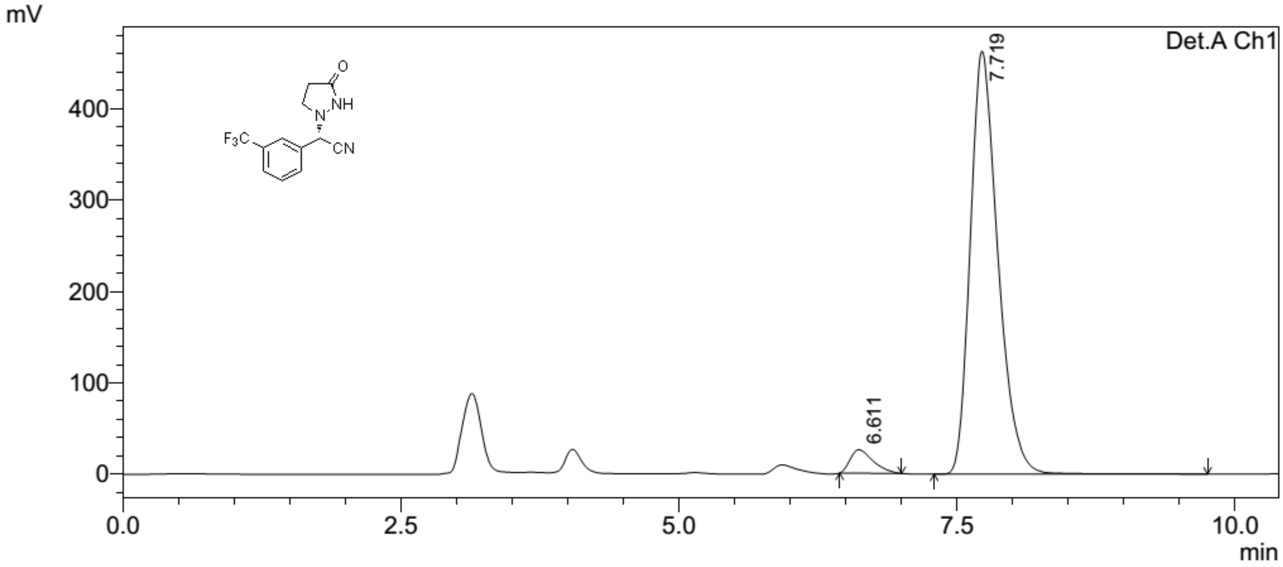

1 Det.A Ch1/254nm

PeakTable

Detector A Ch1 254nm

Peak#	Ret. Time	Area	Height	Area %	Height %
1	6.611	366151	25738	4.392	5.272
2	7.719	7970988	462445	95.608	94.728
Total		8337139	488183	100.000	100.000

Supplementary Figure 65. HPLC spectra of products **5m**.

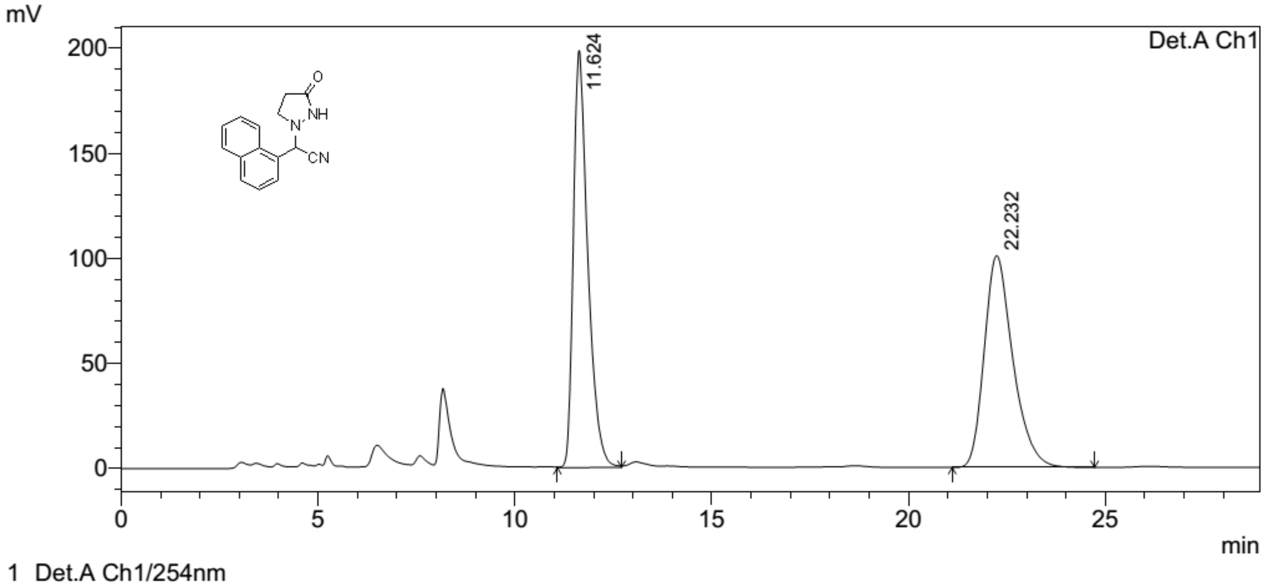

PeakTable

Detector A Ch1 254nm

Peak#	Ret. Time	Area	Height	Area %	Height %
1	11.624	5064877	198419	49.863	66.317
2	22.232	5092659	100778	50.137	33.683
Total		10157537	299197	100.000	100.000

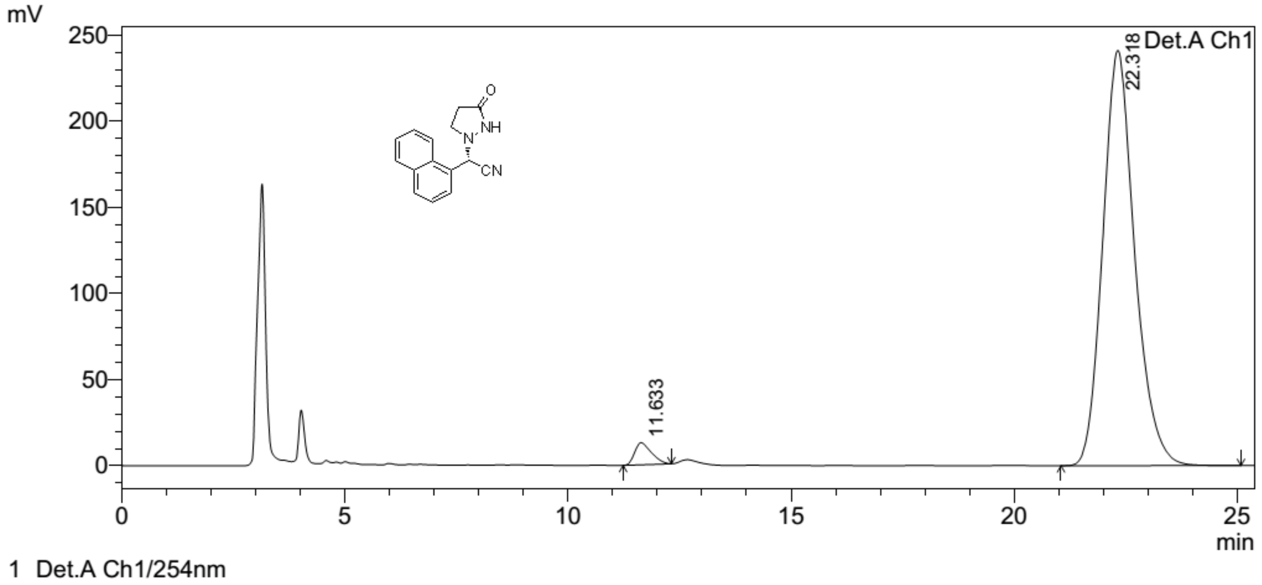

PeakTable

Detector A Ch1 254nm

Peak#	Ret. Time	Area	Height	Area %	Height %
1	11.633	335899	12893	2.726	5.076
2	22.318	11986090	241098	97.274	94.924
Total		12321989	253991	100.000	100.000

Supplementary Figure 66. HPLC spectra of products**5n**.

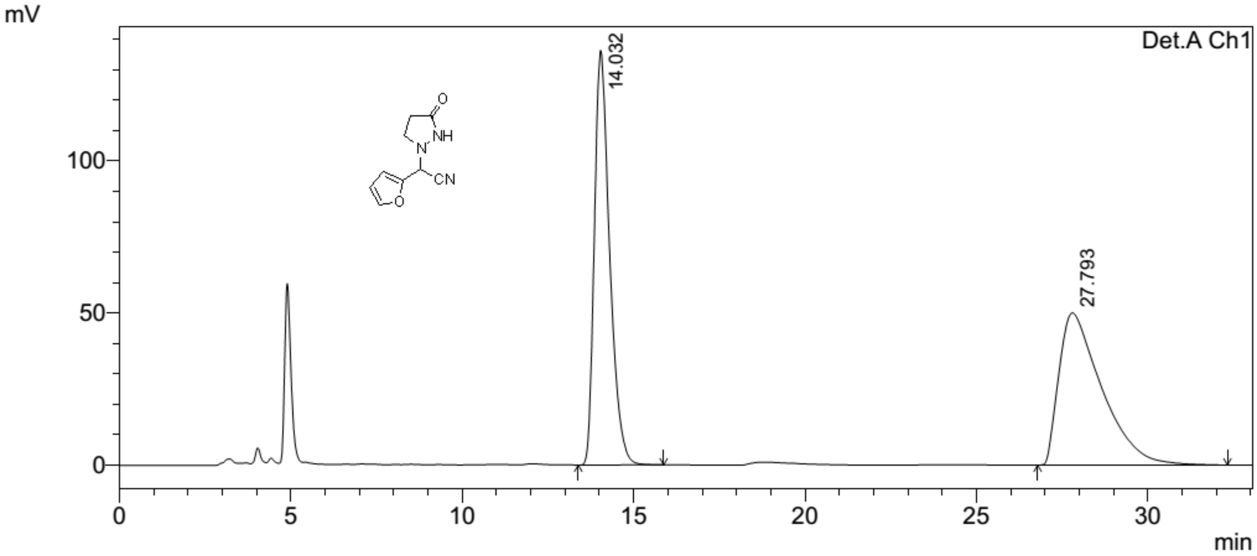

1 Det.A Ch1/254nm

PeakTable

Detector A Ch1 254nm

Peak#	Ret. Time	Area	Height	Area %	Height %
1	14.032	4218214	136237	49.315	73.155
2	27.793	4335447	49995	50.685	26.845
Total		8553661	186232	100.000	100.000

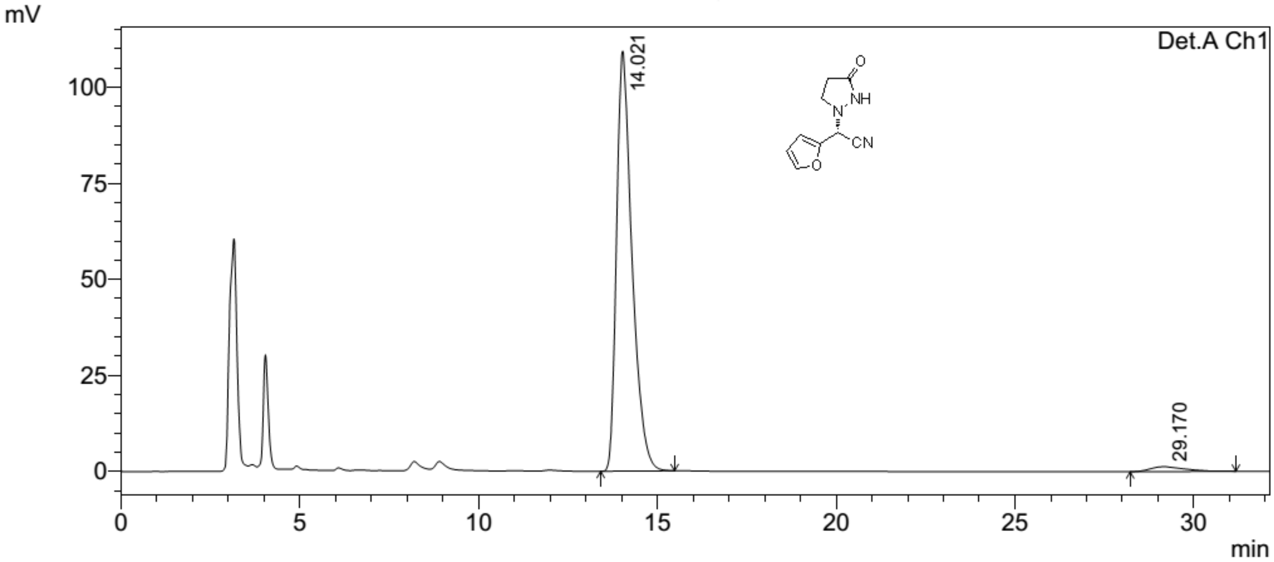

1 Det.A Ch1/254nm

PeakTable

Detector A Ch1 254nm

Peak#	Ret. Time	Area	Height	Area %	Height %
1	14.021	3290907	109396	97.484	98.856
2	29.170	84922	1265	2.516	1.144
Total		3375829	110661	100.000	100.000

Supplementary Figure 67. HPLC spectra of products **5o**.

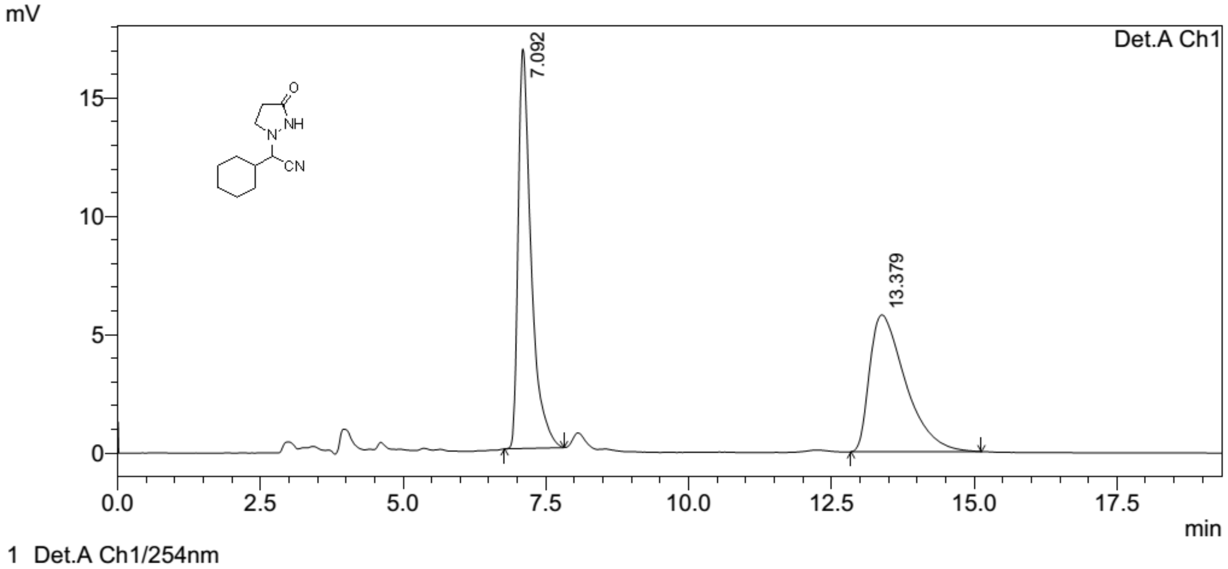

PeakTable

Detector A Ch1 254nm

Peak#	Ret. Time	Area	Height	Area %	Height %
1	7.092	265940	16878	51.237	74.472
2	13.379	253104	5786	48.763	25.528
Total		519044	22664	100.000	100.000

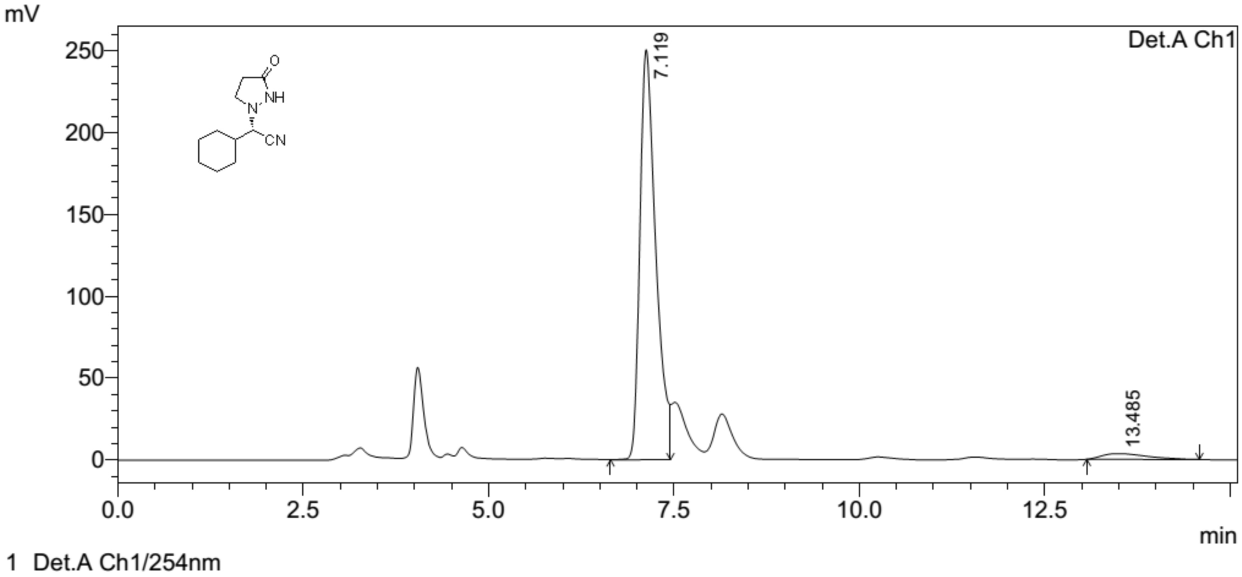

PeakTable

Detector A Ch1 254nm

Peak#	Ret. Time	Area	Height	Area %	Height %
1	7.119	3747046	250493	96.180	98.589
2	13.485	148825	3584	3.820	1.411
Total		3895871	254077	100.000	100.000

Supplementary Figure 68. HPLC spectra of products **6a**.

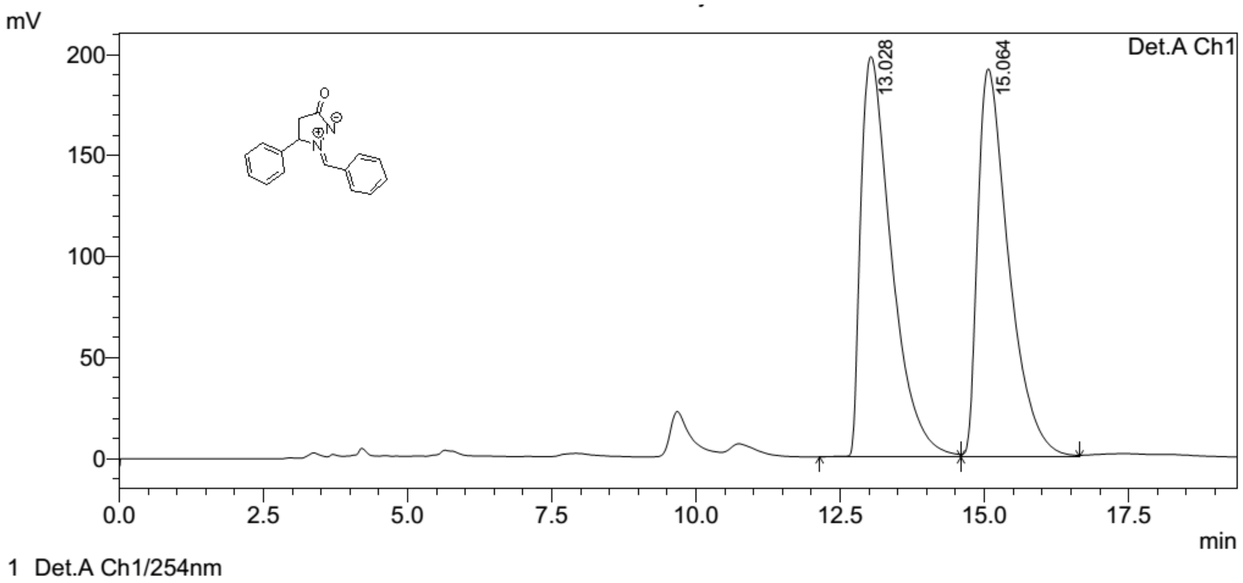

PeakTable

Detector A Ch1 254nm

Peak#	Ret. Time	Area	Height	Area %	Height %
1	13.028	7353272	198233	50.572	50.773
2	15.064	7186895	192195	49.428	49.227
Total		14540167	390429	100.000	100.000

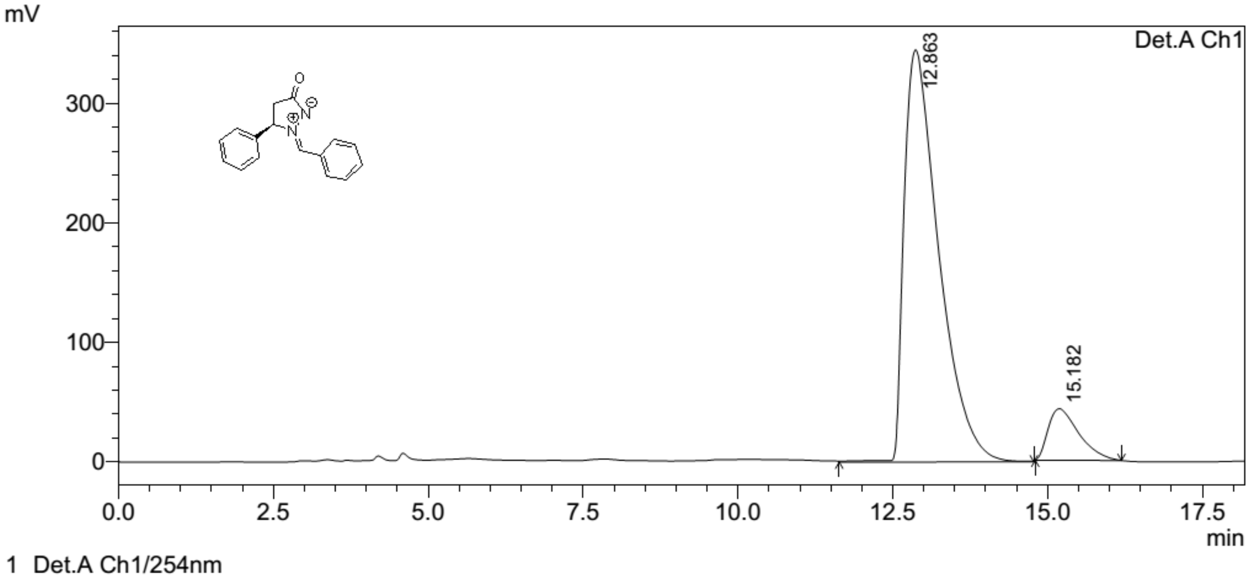

PeakTable

Detector A Ch1 254nm

Peak#	Ret. Time	Area	Height	Area %	Height %
1	12.863	13428185	344900	90.015	88.863
2	15.182	1489519	43226	9.985	11.137
Total		14917704	388127	100.000	100.000

Supplementary Figure 69. HPLC spectra of products **7a**.

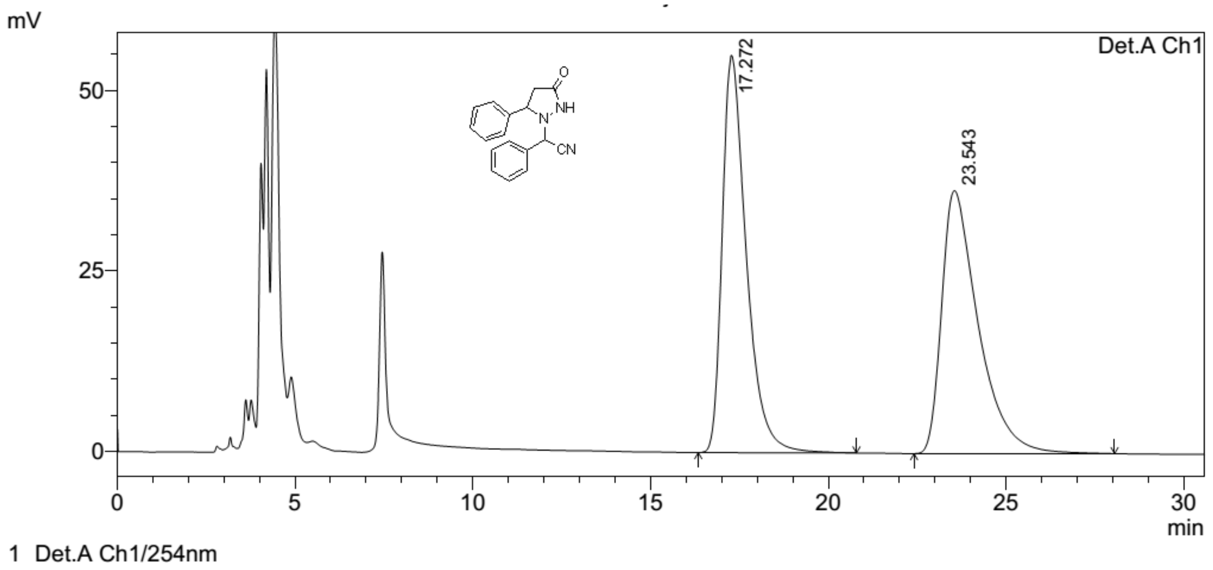

PeakTable

Detector A Ch1 254nm

Peak#	Ret. Time	Area	Height	Area %	Height %
1	17.272	2585308	54996	49.844	60.162
2	23.543	2601505	36418	50.156	39.838
Total		5186813	91414	100.000	100.000

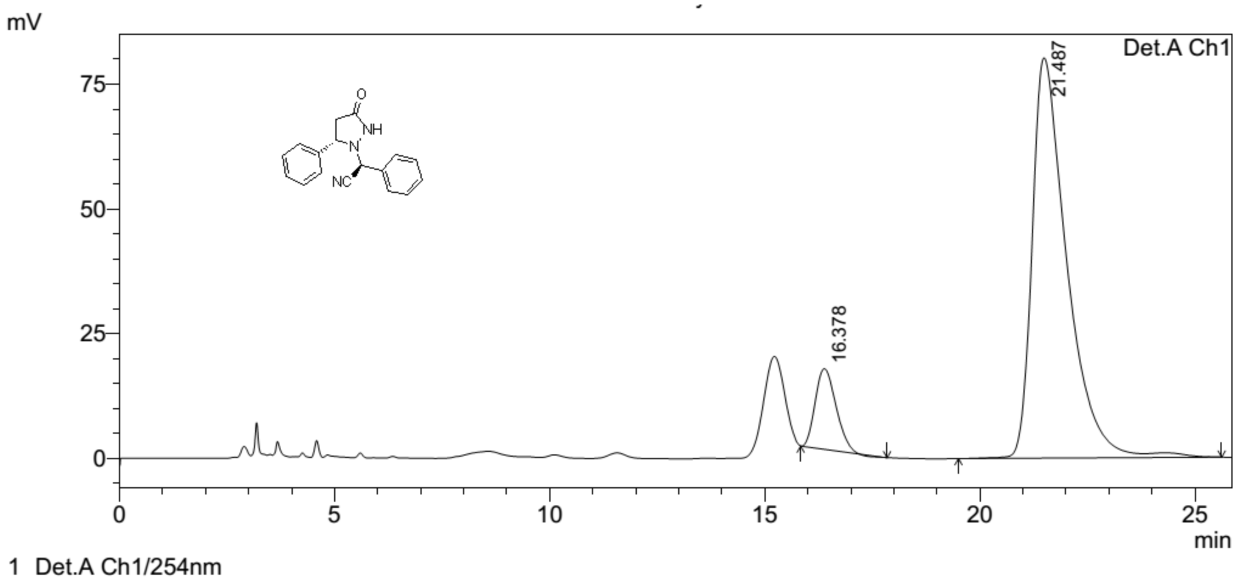

PeakTable

Detector A Ch1 254nm

Peak#	Ret. Time	Area	Height	Area %	Height %
1	16.378	538692	16178	10.720	16.769
2	21.487	4486574	80299	89.280	83.231
Total		5025267	96477	100.000	100.000

Supplementary Figure 70. HPLC spectra of products **6b**.

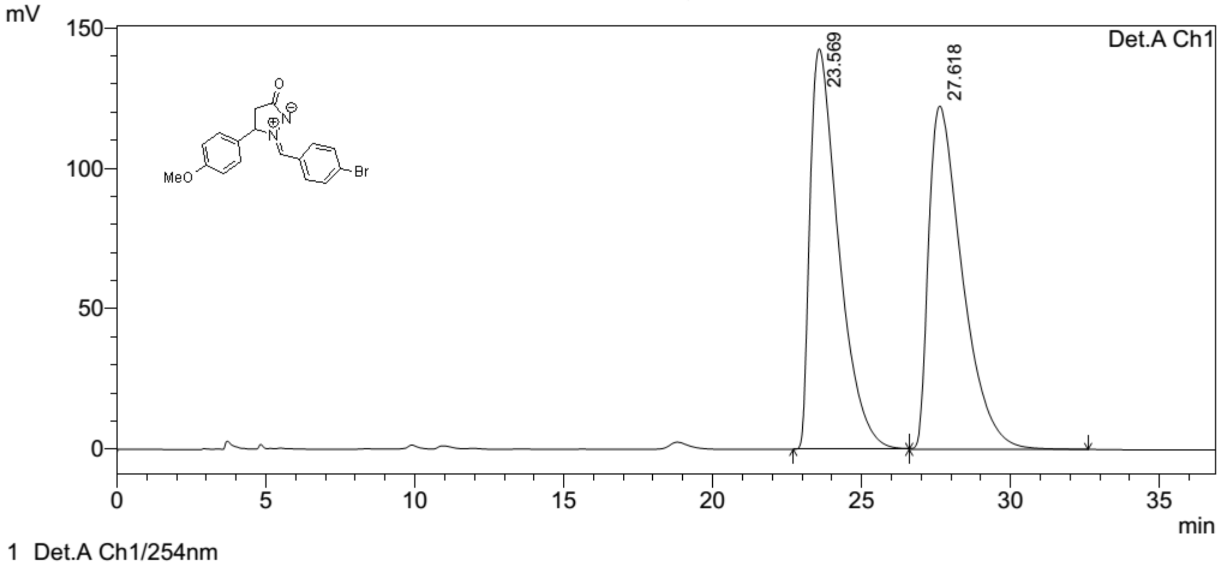

PeakTable

Detector A Ch1 254nm

Peak#	Ret. Time	Area	Height	Area %	Height %
1	23.569	9524222	142552	49.762	53.837
2	27.618	9615285	122230	50.238	46.163
Total		19139507	264783	100.000	100.000

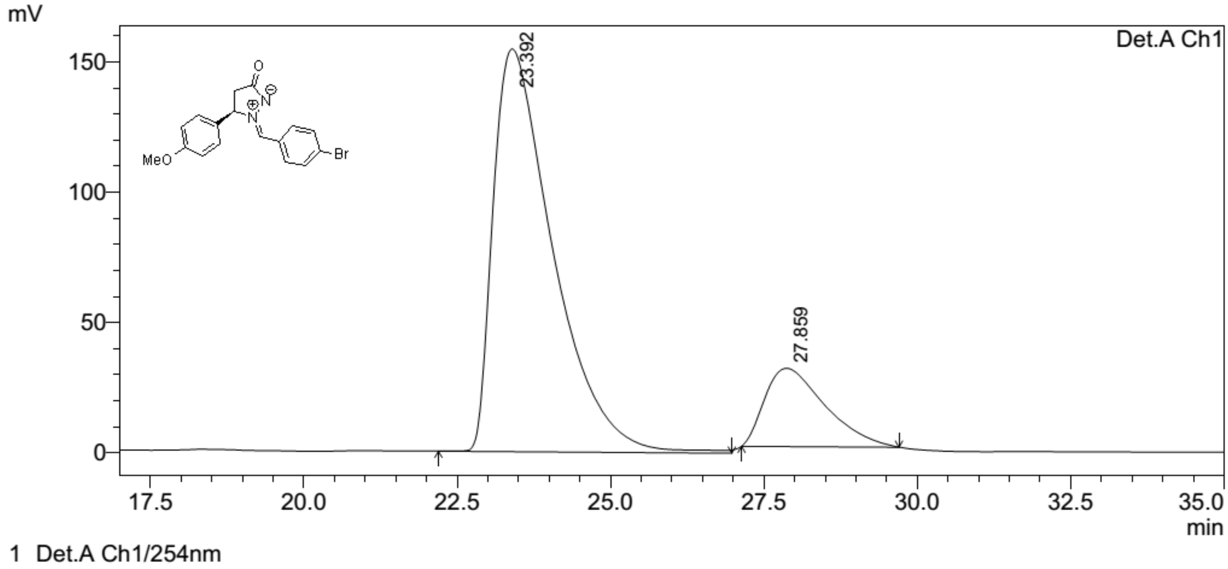

PeakTable

Detector A Ch1 254nm

Peak#	Ret. Time	Area	Height	Area %	Height %
1	23.392	10462895	154618	83.596	83.716
2	27.859	2053107	30076	16.404	16.284
Total		12516002	184693	100.000	100.000

Supplementary Figure 71. HPLC spectra of products **7b**.

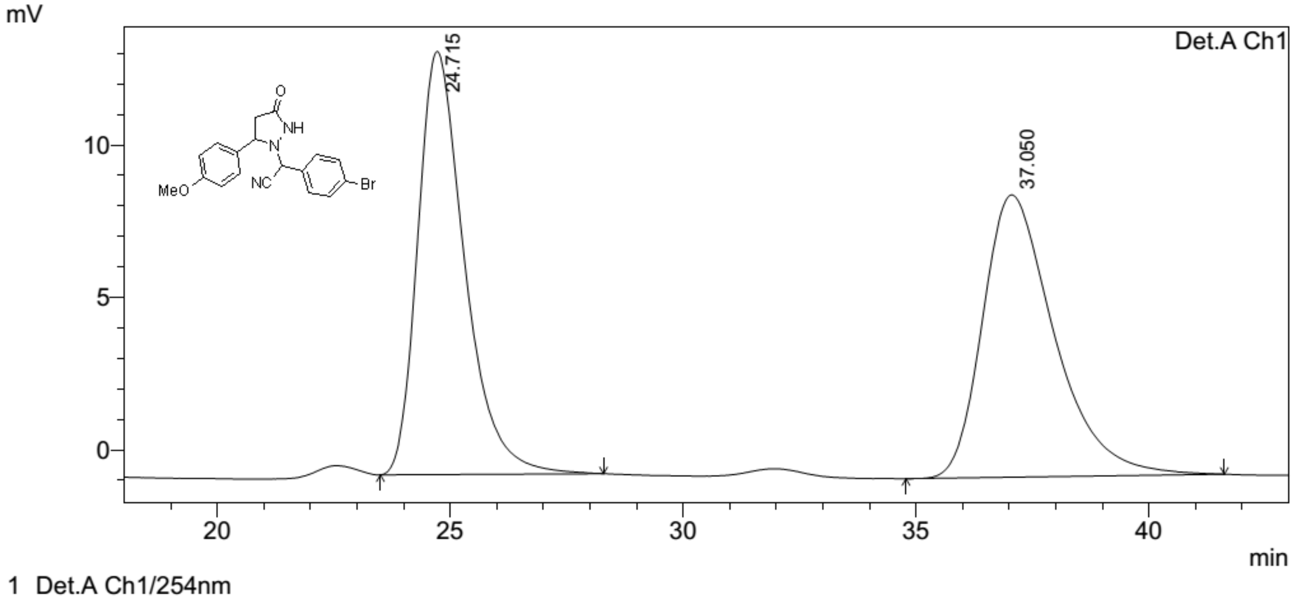

PeakTable

Detector A Ch1 254nm

Peak#	Ret. Time	Area	Height	Area %	Height %
1	24.715	971800	13884	49.484	59.991
2	37.050	992075	9260	50.516	40.009
Total		1963875	23144	100.000	100.000

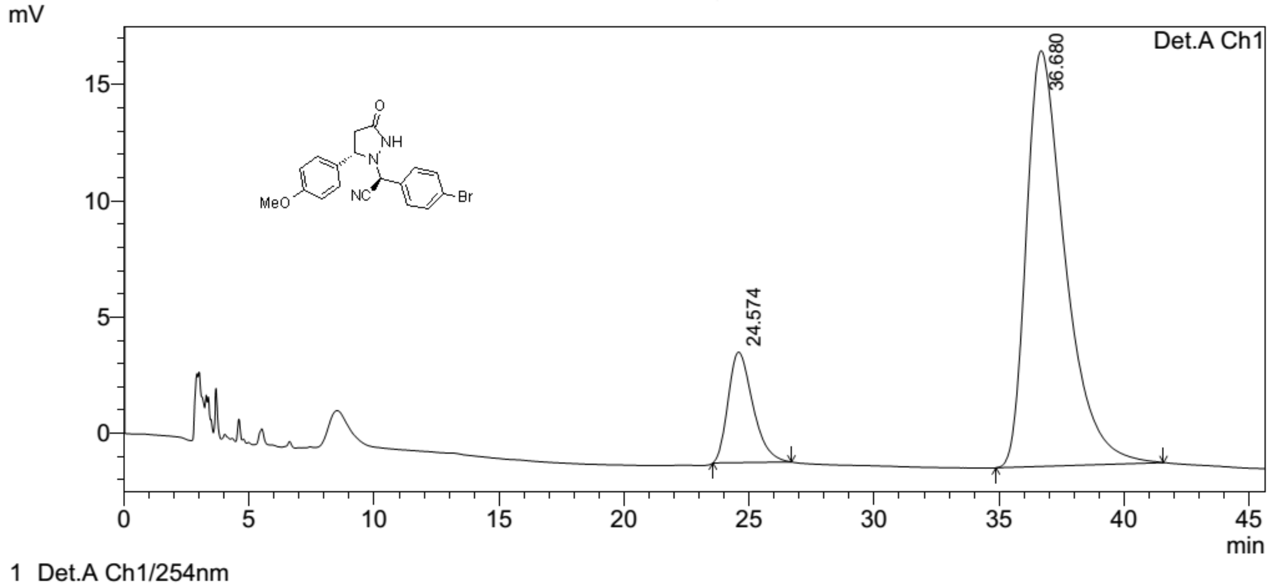

PeakTable

Detector A Ch1 254nm

Peak#	Ret. Time	Area	Height	Area %	Height %
1	24.574	326609	4759	14.414	21.009
2	36.680	1939298	17894	85.586	78.991
Total		2265907	22653	100.000	100.000

Supplementary Figure 72. HPLC spectra of products **6c**.

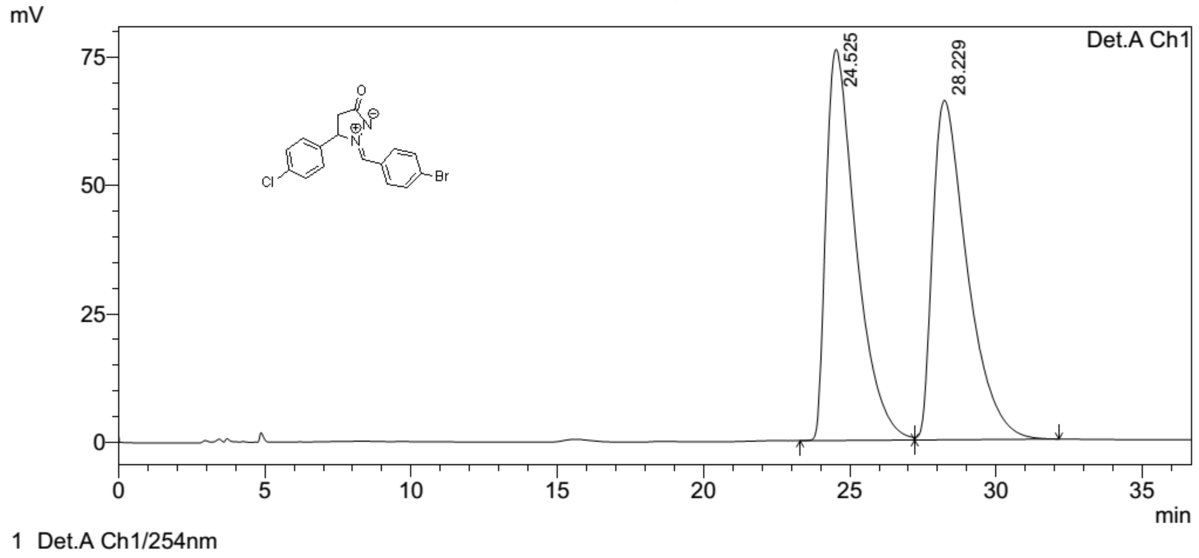

PeakTable

Detector A Ch1 254nm

Peak#	Ret. Time	Area	Height	Area %	Height %
1	24.525	5443147	76177	50.011	53.534
2	28.229	5440831	66120	49.989	46.466
Total		10883978	142297	100.000	100.000

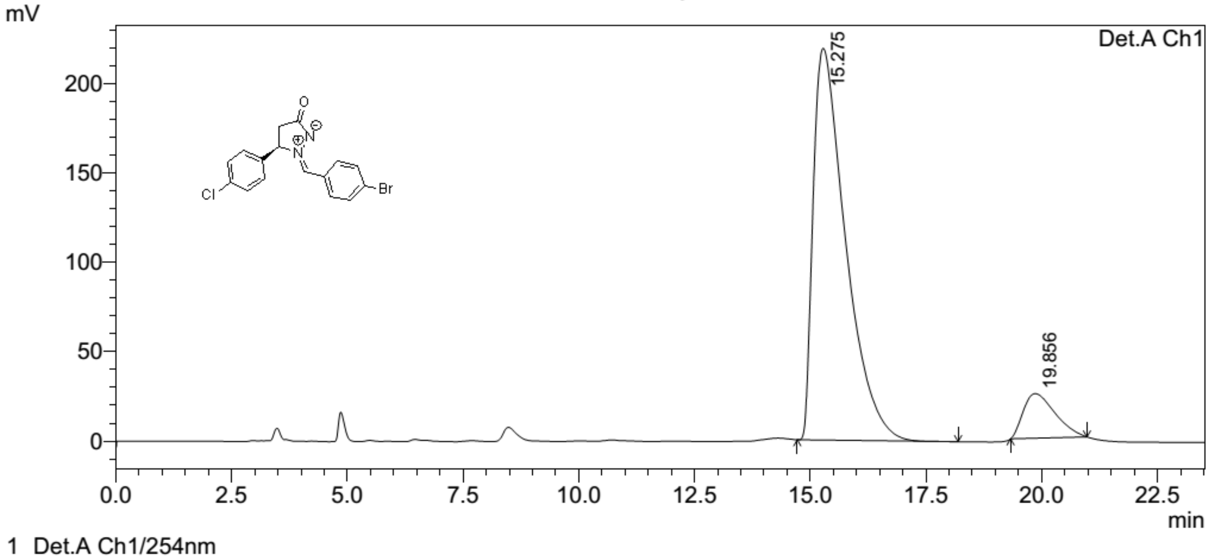

PeakTable

Detector A Ch1 254nm

Peak#	Ret. Time	Area	Height	Area %	Height %
1	15.275	10648865	219093	89.913	89.778
2	19.856	1194711	24946	10.087	10.222
Total		11843576	244038	100.000	100.000

Supplementary Figure 73. HPLC spectra of products **7c**.

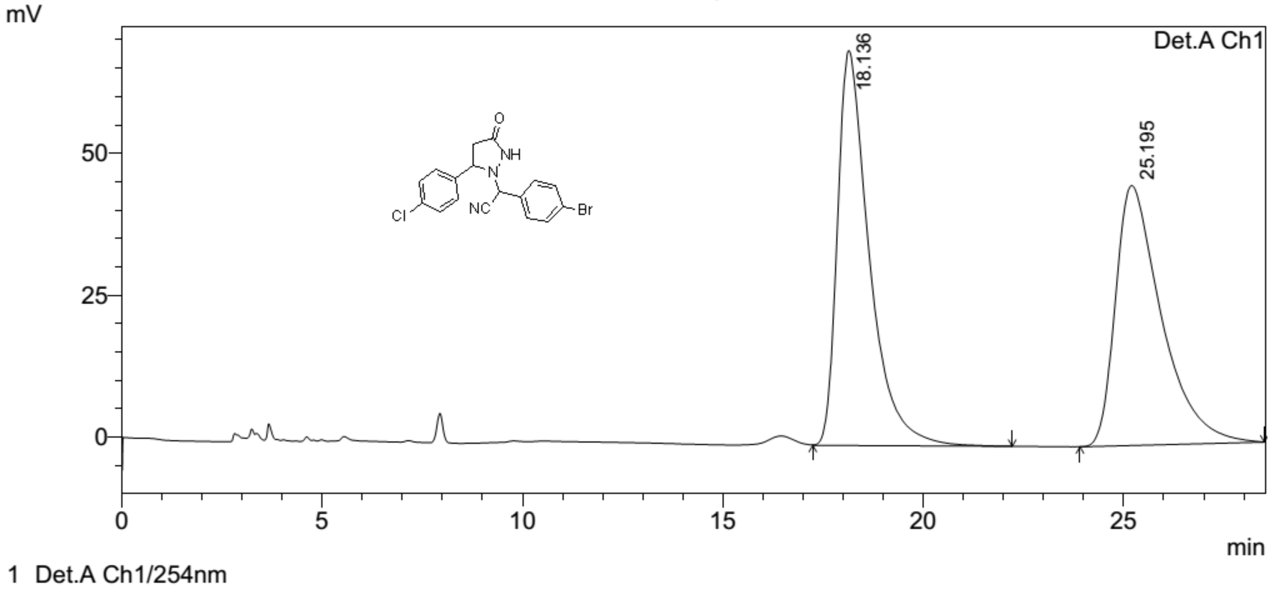

PeakTable

Detector A Ch1 254nm

Peak#	Ret. Time	Area	Height	Area %	Height %
1	18.136	3843579	69559	51.324	60.299
2	25.195	3645272	45799	48.676	39.701
Total		7488851	115358	100.000	100.000

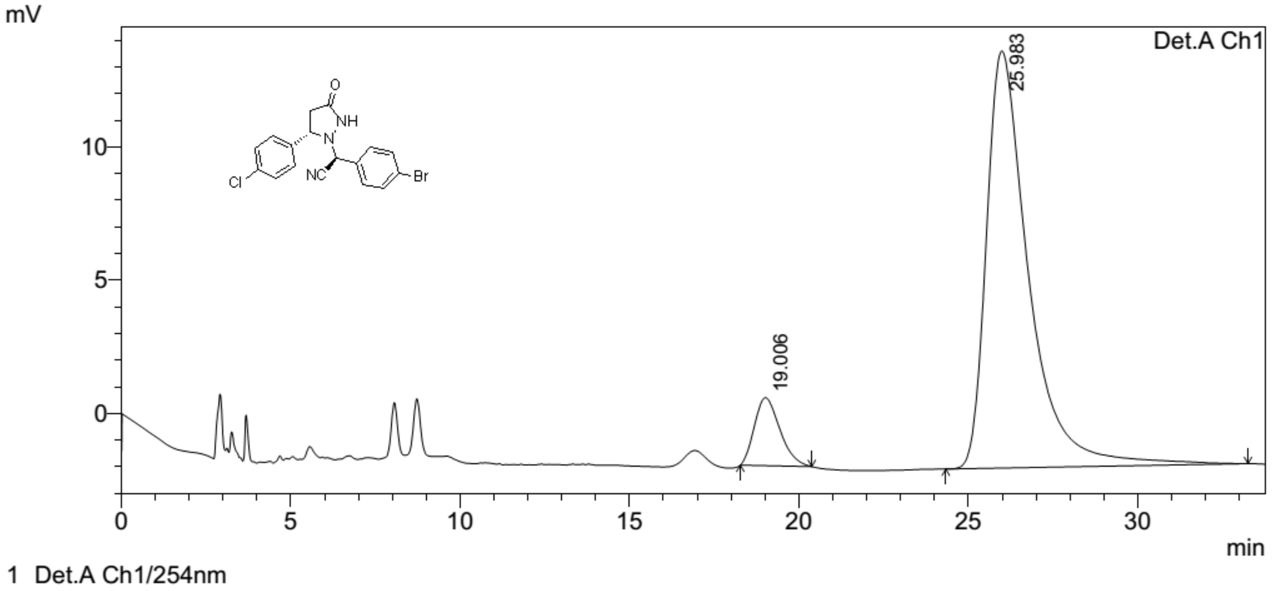

PeakTable

Detector A Ch1 254nm

Peak#	Ret. Time	Area	Height	Area %	Height %
1	19.006	133460	2548	8.947	13.997
2	25.983	1358206	15654	91.053	86.003
Total		1491666	18202	100.000	100.000

Supplementary Figure 74. HPLC spectra of products **6d**.

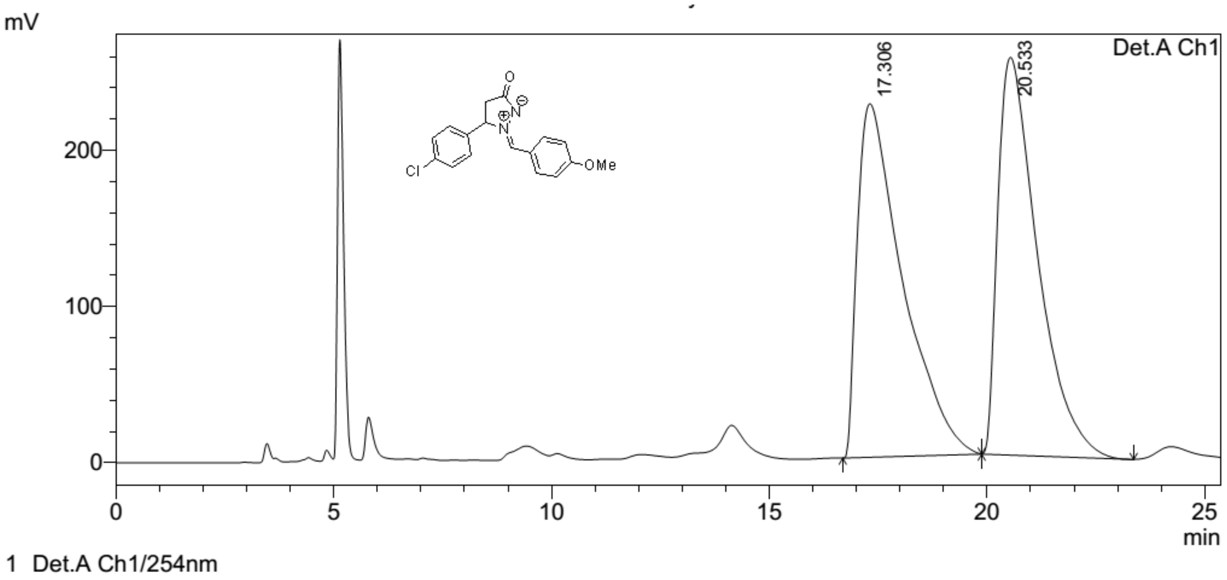

PeakTable

Detector A Ch1 254nm

Peak#	Ret. Time	Area	Height	Area %	Height %
1	17.306	16280565	226397	50.228	47.070
2	20.533	16132700	254583	49.772	52.930
Total		32413266	480979	100.000	100.000

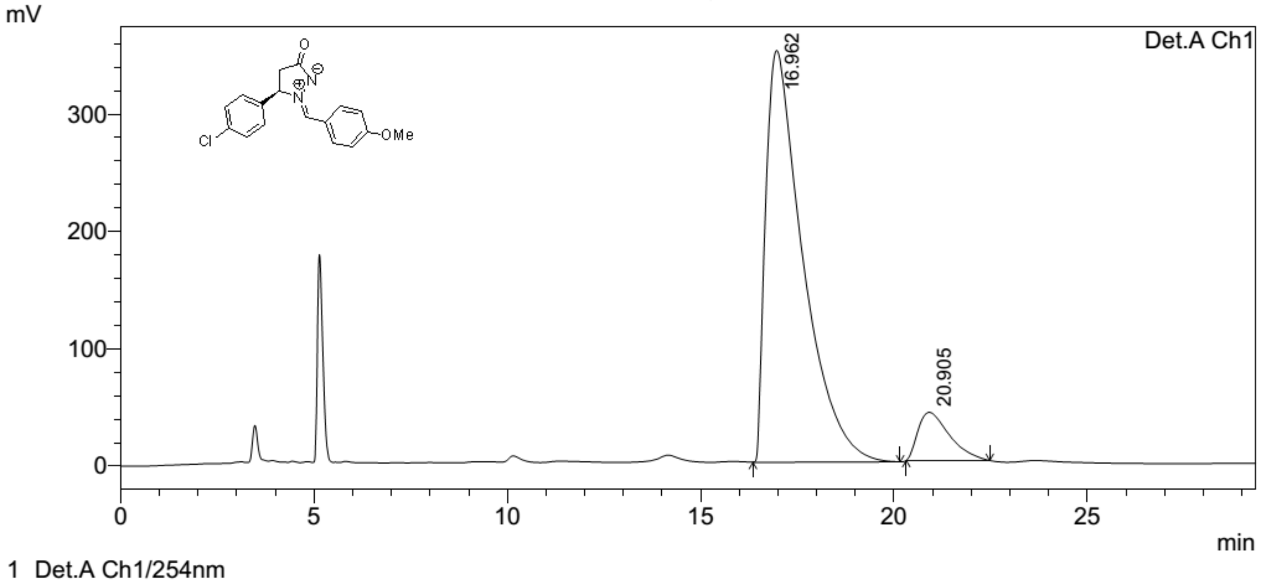

PeakTable

Detector A Ch1 254nm

Peak#	Ret. Time	Area	Height	Area %	Height %
1	16.962	23417357	350981	90.943	89.528
2	20.905	2332067	41054	9.057	10.472
Total		25749423	392035	100.000	100.000

Supplementary Figure 75. HPLC spectra of products **7d**.

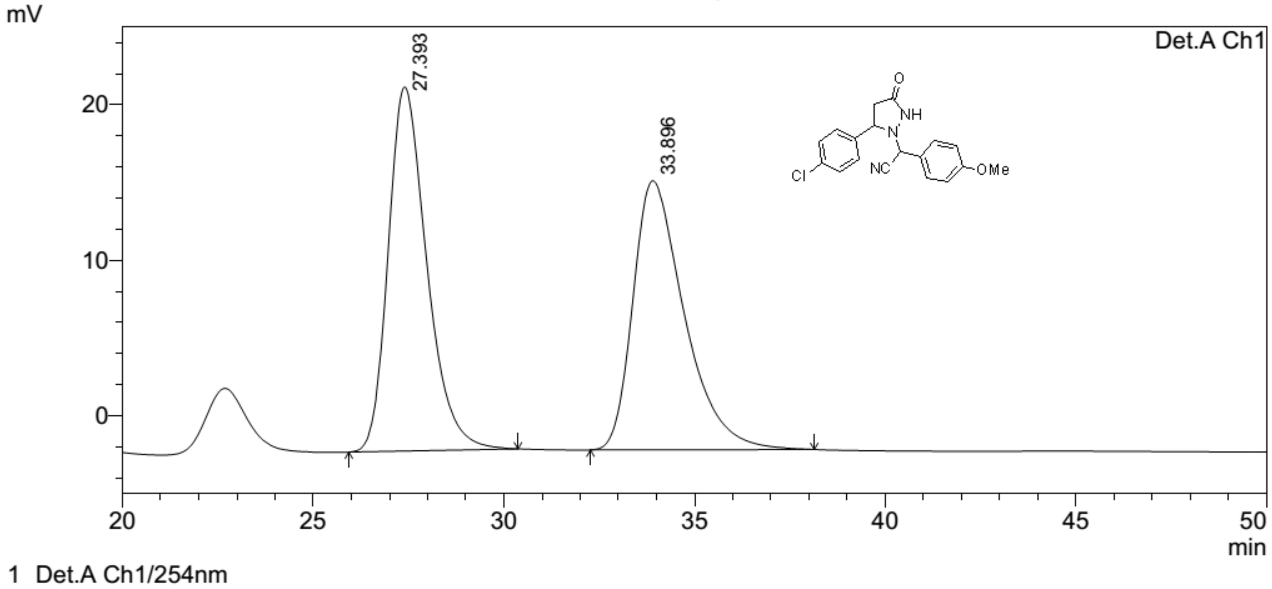

PeakTable

Detector A Ch1 254nm

Peak#	Ret. Time	Area	Height	Area %	Height %
1	27.393	1642989	23430	50.313	57.503
2	33.896	1622532	17316	49.687	42.497
Total		3265520	40745	100.000	100.000

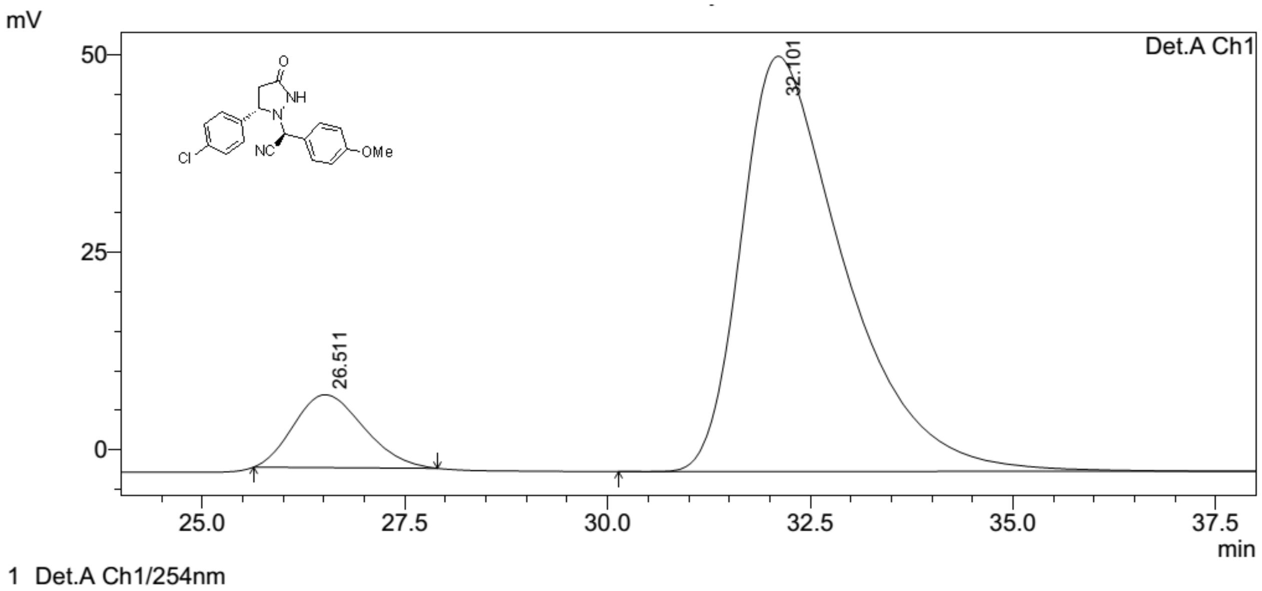

PeakTable

Detector A Ch1 254nm

Peak#	Ret. Time	Area	Height	Area %	Height %
1	26.511	555858	9247	10.673	14.956
2	32.101	4651988	52582	89.327	85.044
Total		5207846	61830	100.000	100.000

Supplementary Figure 76. HPLC spectra of products **6e**.

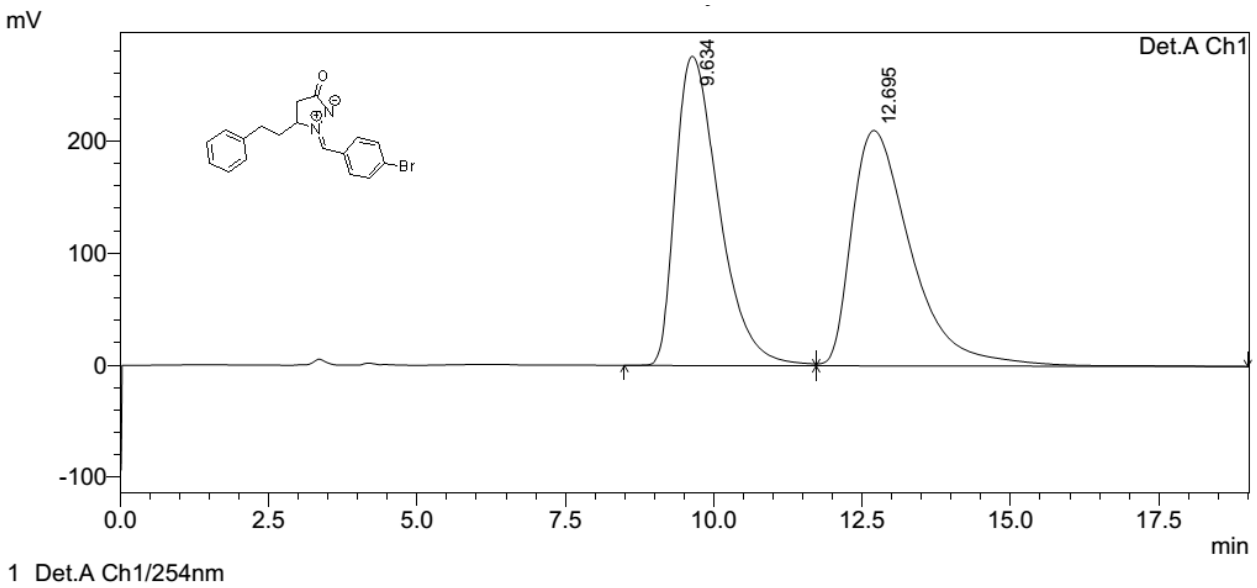

PeakTable

Detector A Ch1 254nm

Peak#	Ret. Time	Area	Height	Area %	Height %
1	9.634	14121236	276305	49.140	56.786
2	12.695	14615745	210267	50.860	43.214
Total		28736980	486572	100.000	100.000

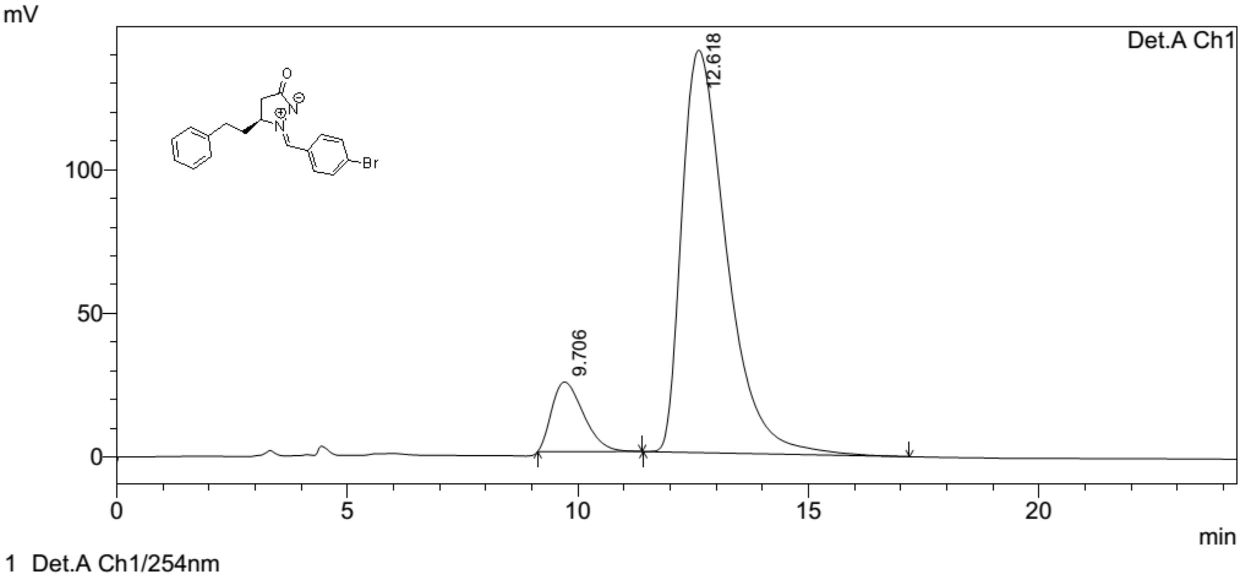

PeakTable

Detector A Ch1 254nm

Peak#	Ret. Time	Area	Height	Area %	Height %
1	9.706	1162809	24267	10.936	14.759
2	12.618	9470075	140153	89.064	85.241
Total		10632883	164420	100.000	100.000

Supplementary Figure 77. HPLC spectra of products **7e**.

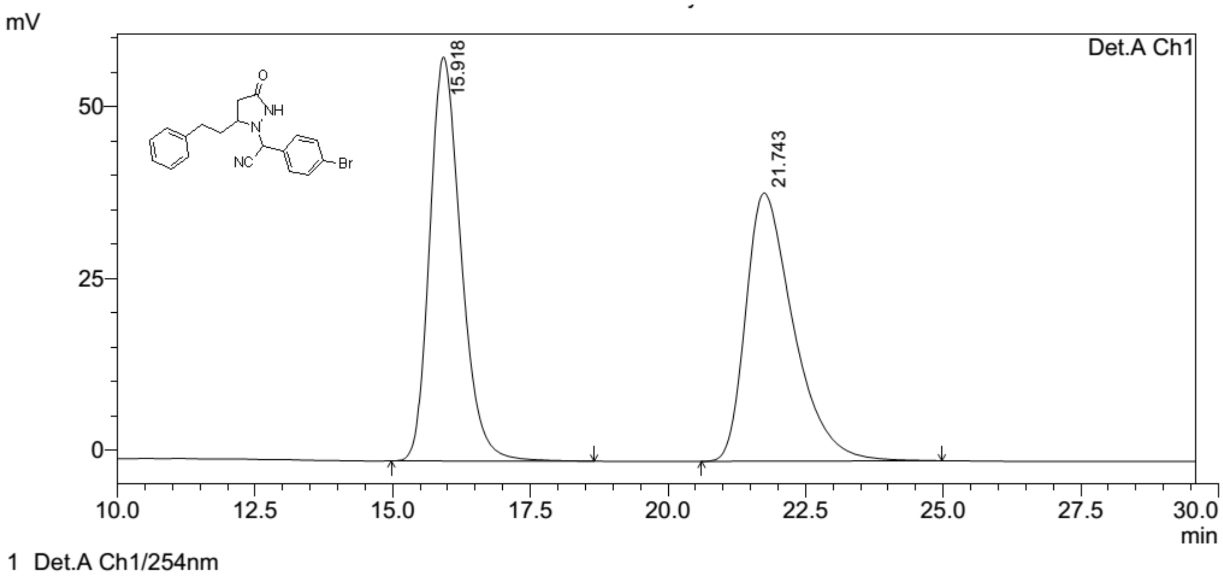

PeakTable

Detector A Ch1 254nm

Peak#	Ret. Time	Area	Height	Area %	Height %
1	15.918	2326230	58733	49.784	60.088
2	21.743	2346434	39012	50.216	39.912
Total		4672664	97744	100.000	100.000

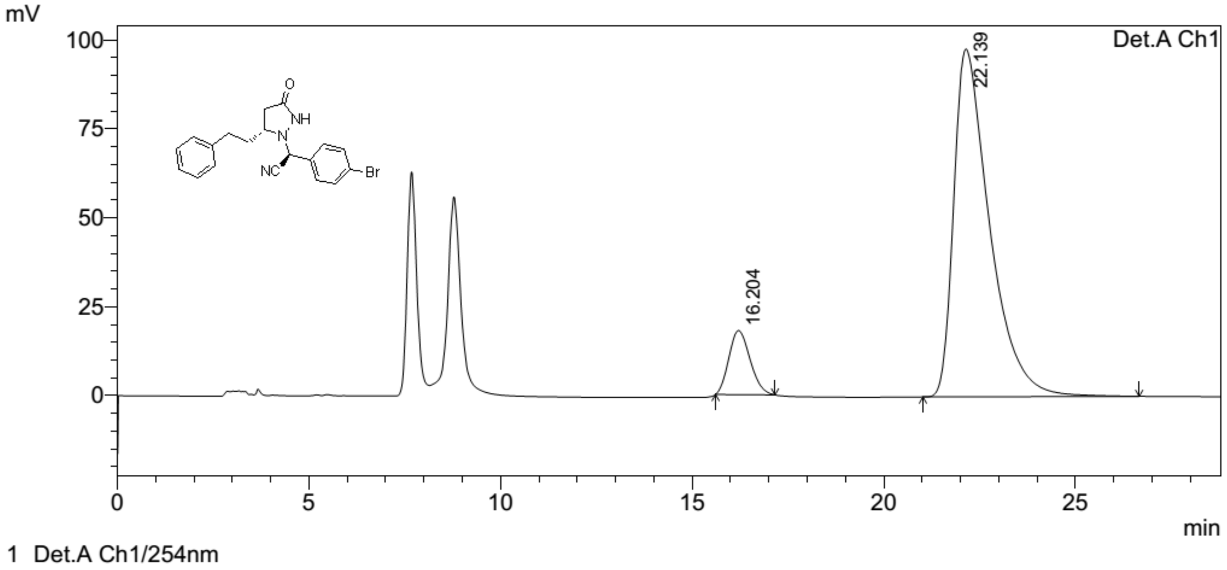

PeakTable

Detector A Ch1 254nm

Peak#	Ret. Time	Area	Height	Area %	Height %
1	16.204	694678	18030	9.842	15.557
2	22.139	6363808	97862	90.158	84.443
Total		7058486	115891	100.000	100.000

Supplementary Table 1. The optimization of the reaction conditions.[a]

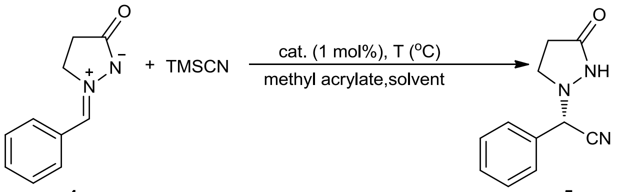

4a **5a**

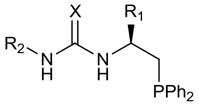

3a': R_1 = Bn, X = S, R_2= 3,5-$CF_3C_6H_4$
3b': R_1 = 2-(S)-sBu, X = S, R_2 = 3,5-$CF_3C_6H_4$
3c': R_1 = 2-(S)-sBu, X = O, R_2 = 3,5-$CF_3C_6H_4$
3d': R_1 = tBu, X = S, R_2 = 3,5-$CF_3C_6H_4$
3e': R_1 = Bn, X = S, R_2 = 4-$NO_2C_6H_4$
3f': R_1 = 2-(S)-sBu, X = S, R_2 = 4-$MeOC_6H_4$

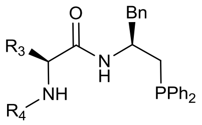

3g': R_3 = Ph, R_4 = 4-nitrobenzoyl
3h': R_3 = Ph, R_4 = 4-methoxybenzoyl
3i': R_3 = H, R_4 = 4-nitrobenzoyl
3j': R_3 = tBu, R_4 = 4-nitrobenzoyl

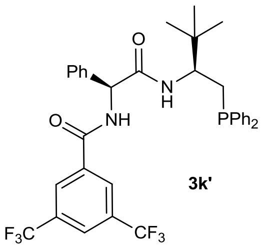

3k'

Entry	catalyst	solvent	T (°C)	Yield (%)[b]	Ee (%)[c]
1	**3a'**	toluene	25	95	10
2	**3b'**	toluene	-	90	15
3	**3c'**	toluene	-	92	10
4	**3d'**	toluene	-	93	40
5	**3e'**	toluene	-	92	20
6	**3f'**	toluene	-	91	16
7	**3g'**	toluene	-	93	60
8	**3h'**	toluene	-	92	56
9	**3i'**	toluene	-	90	-5
10	**3j'**	toluene	-	92	58
11	**3k'**	toluene	-	97	93
12	**3k'**	CH_2Cl_2	-	96	75
13	**3k'**	$CHCl_3$	-	95	67
14	**3k'**	CH_3CN	-	96	20
15	**3k'**	toluene	0	96	93
16	**3k'**	toluene	-30	98	95

[a]Unless otherwise noted, the reactions were performed with **4a** (0.1mmol), TMSCN (0.2mmol) and acrylates (1mol%) in the presence of chiral phosphine(1mol%) in solvent (1 mL) at 25 °C for 5 min.Yields of **5a** were isolated yield. The ees were determined by chiral HPLC analysis.

Supplementary Table 2. Crystal data and structure refinement for CCDC 1407409.

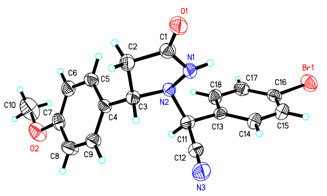

Identification code	cd15022
Empirical formula	C18 H16 Br N3 O2
Formula weight	386.25
Temperature	293(2) K
Wavelength	0.71073 Å
Crystal system	Monoclinic
Space group	P 21/c

Unit cell dimensions	a = 10.000(7) Å	α= 90 °.
	b = 14.735(10) Å	β= 95.070(18) °.
	c = 11.517(8) Å	γ = 90 °.
Volume	1690(2) Å^3	
Z	4	
Density (calculated)	1.518 Mg/m^3	
Absorption coefficient	2.447 mm^{-1}	
F(000)	784	
Crystal size	0.180 x 0.140 x 0.080 mm^3	
Theta range for data collection	2.044 to 26.000 °.	
Index ranges	-6<=h<=12, -18<=k<=18, -14<=l<=14	
Reflections collected	10116	
Independent reflections	3319 [R(int) = 0.0972]	
Completeness to theta = 25.242 °	100.0 %	
Absorption correction	Semi-empirical from equivalents	
Max. and min. transmission	0.7456 and 0.5247	
Refinement method	Full-matrix least-squares on F^2	
Data / restraints / parameters	3319 / 1 / 222	
Goodness-of-fit on F^2	0.927	
Final R indices [I>2sigma(I)]	R1 = 0.0543, wR2 = 0.1058	
R indices (all data)	R1 = 0.1214, wR2 = 0.1302	
Extinction coefficient	n/a	
Largest diff. peak and hole	0.437 and -0.402 e.Å^{-3}	

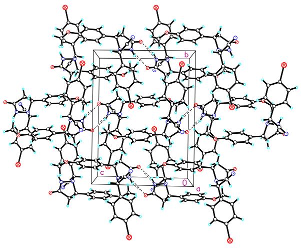

The single crystal x-ray diffraction data for 7b (CCDC 1407409)

CCDC 1407409 (compound **7b**) contains the supplementary crystallographic data for this paper. These data can be obtained free of charge from The Cambridge Crystallographic Data Centre via www.ccdc.cam.ac.uk/data_request/cif.

According to the absolute configuration of **5a** and **6a**, which were determined by comparison of the optical rotation with literature data, the configuration of the compound **7a** could be estimated as *trans* or *syns* (*S*,*S* or *S*,*R*), and on the basis of the relative configuration of the compound **7b**, so the absolute configuration of **7a** is *S*,*S*.

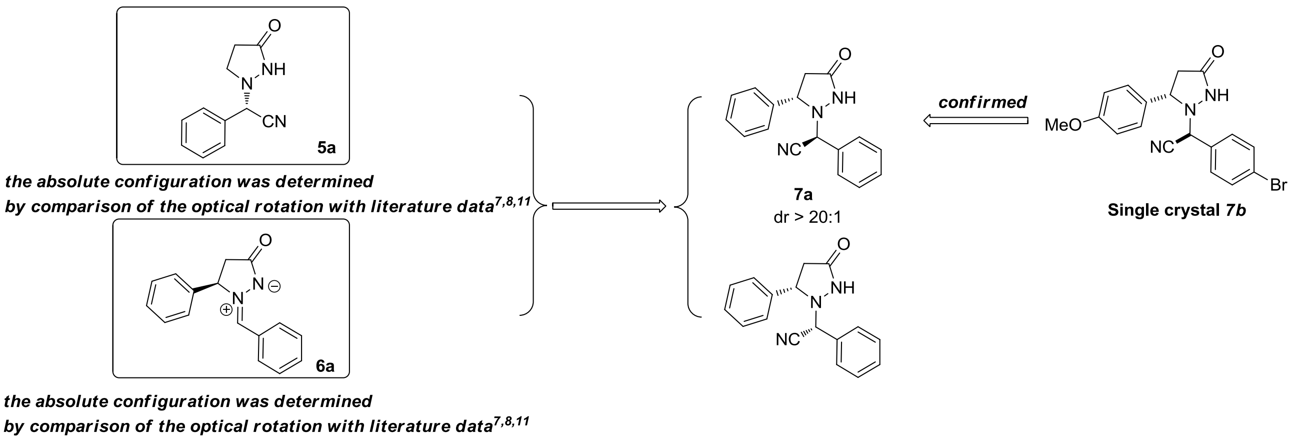

Supplementary Methods:

General

The ^{1}H NMR spectra were recorded on a Bruker (400 MHz). All chemical shifts (δ) were given in ppm. Data were reported as follows: chemical shift, intergration, multiplicity (s = single, d = doublet, t = triplet, q = quartet, br = broad, m = multiplet) and coupling constants (Hz). ^{13}C NMR spectra were recorded on a DPX-400 (400 MHz). ^{19}F NMR were recorded on a Agilent 400 (376 MHz). ^{31}P NMR spectra were recorded on a Agilent 400 (163 MHz). Flash column chromatography was performed using H silica gel. For thin-layer chromatography (TLC), silica gel plates (HSGF 254) were used and compounds were visualized by irradiation with UV light. Analytical high performance liquid chromatography (HPLC) was carried out on SHIMADZU equipment using chiral columns. Melting points were determined on a SGW X-4 melting point and were uncorrected. Optical rotations were measured on a JASCO P-1010 Polarimeter at λ =589 nm. IR spectra were recorded on a Perkin-Elmer 983G instrument. Mass spectra analysis was performed on API 200 LC/MS system (Applied Biosystems Co. Ltd.).Commercially available materials purchased from Adamas-beta®, Alfa-Acear® or Sigma-Aldrich®were used as received. The synthesis of imines and the catalysts were prepared according to the reported methods[1-2].

The Dipeptide-based Organophosphines[1-3]

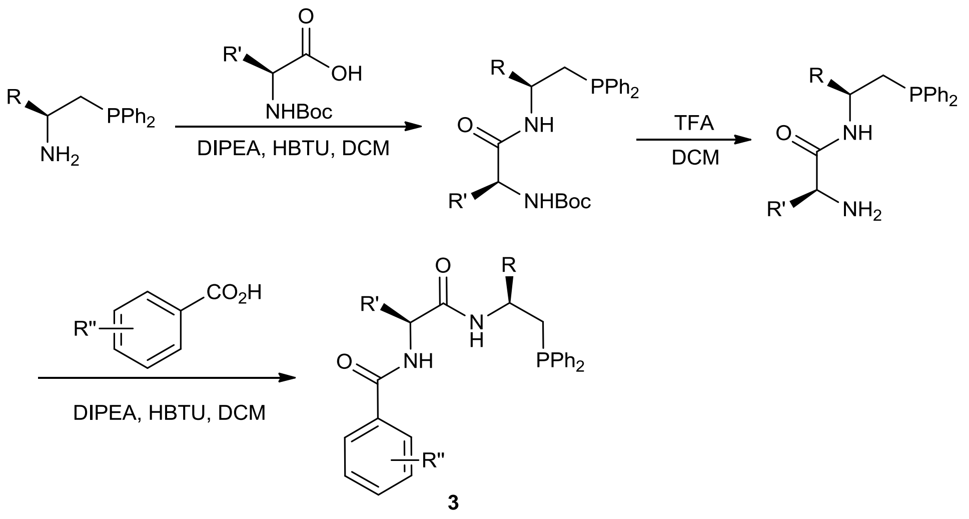

The Dipeptide-based Organophosphines were prepared according to the reported methods.[1-3]

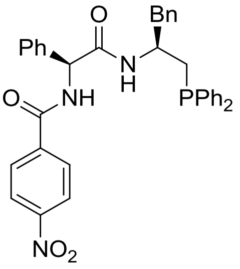

Catalyst **3j**.Yellow solid; m.p. 110-112 °C;$[\alpha]_D^{24.9}$= 65.6 (*c* = 1.00, $CHCl_3$);IR (neat) *v* 3264, 3061, 1677, 1599, 1528, 1483, 1344, 1108cm^{-1};^{1}H NMR (400 MHz, $CDCl_3$) δ 8.19-8.17 (d, *J* = 8 Hz, 2H), 7.92-7.90 (d, *J* = 8 Hz, 2H), 7.42-7.41 (br, 2H), 7.32-7.12 (m, 16H), 7.05-7.03 (d, *J* = 8 Hz, 2H), 6.35-6.30 (m, 1H), 5.63-5.59 (m, 1H), 4.14-4.07 (m, 1H), 2.94-2.92 (d, *J* = 8 Hz, 2H), 2.21-2.19 (d, *J* = 8 Hz, 2H); ^{13}C NMR (100 MHz, $CDCl_3$) δ 169.10, 164.62, 149.66, 139.28, 137.82-137.70 (d, *J* = 12 Hz), 137.52-137.40 (d, *J* = 12 Hz), 137.24, 137.22, 132.87-132.67 (d, *J* = 20 Hz), 132.65-132.46 (d, *J* = 20 Hz), 129.34, 129.09, 128.89, 128.80, 128.61, 128.59-128.57 (d, *J* = 2 Hz), 128.52-128.50 (d, *J* = 2 Hz), 128.47-128.45 (d, *J* = 2 Hz), 127.51, 126.64, 123.67, 123.48, 57.64, 49.92-49.76 (d, *J* = 16 Hz), 41.25-41.17 (d, *J* = 8 Hz), 33.25-33.10 (d, *J* = 15 Hz);^{31}P NMR (400 MHz, $CDCl_3$) δ -24.41; HRMS (ESI): calcd. for $[M+H]^+$ ($C_{36}H_{33}N_3O_4P$) requires 602.2130, found 602.2190.

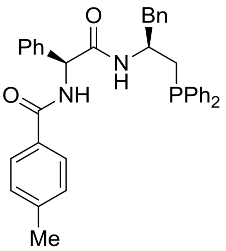

Catalyst**3k**.White solid; m.p. 168-171 °C; $[\alpha]_D^{26.0}$= 61.2 (*c* = 1.00, $CHCl_3$);IR (neat) *v* 3289, 3059,

2923, 1658, 1630, 1563, 1494, 1433, 738 cm^{-1};^{1}H NMR (400 MHz, $CDCl_3$) δ 7.70-7.68 (d, *J* = 8 Hz, 2H), 7.52-7.50 (d, *J* = 8 Hz, 1H), 7.42-7.40 (m, 2H), 7.30-7.13 (m, 18H), 7.03-7.01 (d, *J* = 8 Hz, 2H), 6.48-6.46 (d, *J* = 8 Hz, 1H), 5.64-5.62 (d, *J* = 8 Hz, 1H), 4.15-4.07 (m, 1H), 2.91-2.90 (d, *J* = 4 Hz, 2H), 2.38 (s, 3H), 2.22-2.19 (m, 2H); ^{13}C NMR (100 MHz, $CDCl_3$) δ 169.48, 166.59, 142.19, 138.10-137.98 (d, *J* = 10 Hz), 138.04, 137.77-137.64 (d, *J* = 10 Hz), 137.51, 132.94-132.75 (d, *J* = 20 Hz), 132.68-132.49 (d, *J* = 20 Hz), 131.00, 129.35, 129.16, 128.86, 128.78, 128.63, 128.51-128.50 (d, *J* = 2 Hz), 128.45-128.43 (d, *J* = 2 Hz), 128.38, 128.16, 127.42, 127.24, 126.48, 57.36, 49.67-49.52 (d, *J* = 15 Hz), 41.30-41.22 (d, *J* = 8 Hz), 33.33-33.17 (d, *J* = 15 Hz), 21.48;
^{31}P NMR (400 MHz, $CDCl_3$) δ -24.01; HRMS (ESI): calcd. for $[M+H]^+$ ($C_{37}H_{36}N_2O_2P$) requires 571.2436, found 571.2498.

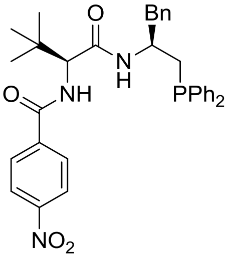

Catalyst**3l**.Yellow solid; m.p. 85-88 ^{o}C;$[\alpha]_D^{25.8}$= 26.0 (*c* = 1.00, $CHCl_3$);IR (neat) *v* 3307, 3070, 2963, 1795, 1640, 1601, 1528, 1452, 1346, 1227, 1107 cm^{-1}; ^{1}H NMR (400 MHz, $CDCl_3$) δ 8.49-8.47 (d, *J* = 8 Hz, 1H), 8.24-8.22 (d, *J* = 8 Hz, 2H), 7.90-7.88 (d, *J* = 8 Hz, 2H), 7.37-7.29 (m, 9H), 7.16-7.12 (t, *J* = 8 Hz, 2H), 7.08-7.00 (m, 4H), 6.23-6.21 (d, *J* = 8 Hz, 1H), 4.45-4.42 (d, *J* = 8 Hz, 1H), 4.25-4.18 (m, 1H), 2.92-2.81 (m, 2H), 2.34-2.29 (m, 1H), 2.19-2.13 (m, 1H), 1.06 (s, 9H); ^{13}C NMR (100 MHz, $CDCl_3$) δ 169.84, 165.59, 149.52, 139.91, 138.43-138.31 (d, *J* = 12 Hz), 137.62-137.48 (d, *J* = 14 Hz), 137.44, 133.20-133.01 (d, *J* = 20 Hz), 132.41-132.22 (d, *J* = 20 Hz), 132.02, 129.43, 129.07, 128.71, 128.66-128.62 (d, *J* = 4 Hz), 128.58-128.55 (d, *J* = 4 Hz), 128.36, 128.29, 126.47, 124.28, 123.67, 61.40, 48.84-48.68 (d, *J* = 16 Hz), 42.12-42.05 (d, *J* = 7 Hz), 35.58, 33.30-33.15 (d, *J* = 15 Hz), 27.07-27.05 (d, *J* = 2 Hz);^{31}P NMR (400 MHz, $CDCl_3$) δ -24.72; HRMS (ESI): calcd. for $[M+H]^+$ ($C_{34}H_{37}N_3O_4P$) requires 582.2443, found 582.2502.

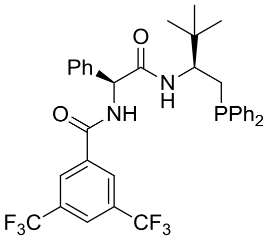

Catalyst **3m**.White solid; m.p. 135-139°C; $[\alpha]_D^{25.3}$= -95.2 (*c* = 1.00, $CHCl_3$);IR (neat) *v* 3296, 3070,

2965, 1659, 1644, 1547, 1307, 1277, 1139, 1109 cm^{-1};^{1}H NMR (400 MHz, $CDCl_3$) δ 8.26 (s, 2H), 7.99 (s, 1H), 7.83-7.81 (d, *J* = 8 Hz, 1H), 7.60-7.58 (d, *J* = 8 Hz, 2H), 7.51-7.48 (m, 1H), 7.42-7.34 (m, 9H), 7.17-7.13 (m, 2H), 5.60-5.58 (d, *J* = 8 Hz, 1H), 5.45-5.43 (d, *J* = 8 Hz, 1H), 3.86-3.78 (m, 1H), 2.42-2.40 (d, *J* = 8 Hz, 1H), 1.63-1.59 (m, 2H), 0.83 (s, 9H); ^{13}C NMR (100 MHz, CD_3SOCD_3) δ 165.54, 165.12, 164.76, 164.55, 157.81, 157.02, 150.49, 150.27, 148.19, 144.40, 144.40, 144.00, 143.94, 143.48, 140.39, 134.02, 133.63, 132.68, 132.03, 131.67, 130.82, 130.56, 130.10, 129.81, 128.52, 128.38, 127.06, 124.14, 119.25, 116.55, 115.72, 79.66, 56.51, 49.07, 19.01;^{31}P NMR (400 MHz, $CDCl_3$) δ -22.38; HRMS (ESI): calcd. for $[M+Na]^+$ ($C_{35}H_{34}F_6N_2O_2P$) requires 659.2184, found 659.2237.

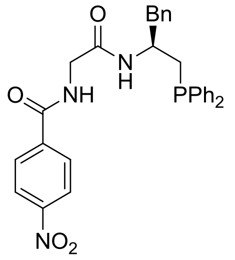

Catalyst**3n**.Yellow solid; m.p. 100-103°C; $[\alpha]_D^{25.1}$= -5.77 (*c* = 1.00, $CHCl_3$);IR (neat) *v* 3289, 3070, 2927, 1643, 1600, 1433, 1345, 1298, 1242, 1108 cm^{-1};^{1}H NMR (400 MHz, $CDCl_3$) δ 8.15-8.10 (br, 2H), 7.90-7.88 (d, *J* = 8 Hz, 2H), 7.35-7.08 (m, 15H), 4.29-4.26 (br, 1H), 3.95-3.81 (qd, *J* = 16 Hz, *J* = 4 Hz, 2H), 2.95-2.92 (t, *J* = 4 Hz, 2H), 2.37-2.32 (dd, *J* = 12 Hz, *J* = 4 Hz, 1H), 2.23-2.17 (dd, *J* = 12 Hz, *J* = 4 Hz, 1H); ^{13}C NMR (100 MHz, $CDCl_3$) δ 168.28, 165.52, 149.58, 138.89, 138.08-137.96 (d, *J* = 12 Hz), 137.91-137.78 (d, *J* = 12 Hz), 137.53, 132.82-132.71 (d, *J* = 11 Hz), 132.63-132.52 (d, *J* = 11 Hz), 129.37, 128.84-128.76 (d, *J* = 8 Hz), 128.57-128.55 (d, *J* = 2 Hz), 128.50, 128.48, 128.39, 126.56, 123.57, 49.17-49.01 (d, *J* = 16 Hz), 43.85, 41.78-41.70 (d, *J* = 8 Hz), 38.62, 33.55-33.41 (d, *J* = 21 Hz); ^{31}P NMR (400 MHz, $CDCl_3$) δ -23.66; HRMS (ESI): calcd. for $[M+H]^+$ ($C_{30}H_{29}N_3O_4P$) requires 526.1817, found 526.1892.

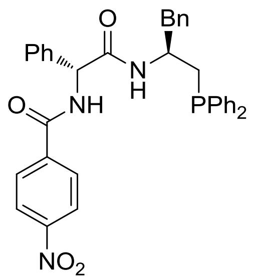

Catalyst**3o**.Yellow solid; m.p. 94-96°C; $[\alpha]_D^{25.6}$= -6.65 (*c* = 1.00, $CHCl_3$);IR (neat) *v* 3279, 3062, 2921, 1677, 1632, 1599, 1433, 1344, 867cm^{-1};^{1}H NMR (400 MHz, $CDCl_3$) δ 8.23-8.21 (d, *J* = 8 Hz,

2H), 7.93-7.91 (d, *J* = 8 Hz, 2H), 7.39-7.29 (m, 13H), 7.22-7.20 (m, 2H), 7.15-7.08 (m, 3H), 6.75-6.73 (d, *J* = 8 Hz, 2H), 5.59-5.54 (br, 1H), 5.21-5.20 (br, 1H), 4.40-4.33 (m, 1H), 2.84-2.73 (qd, *J* = 12 Hz, *J* = 4 Hz, 2H), 2.28-2.13 (qd, *J* = 16 Hz, *J* = 8 Hz, 2H); ^{13}C NMR (100 MHz, $CDCl_3$) δ 168.86, 164.63, 149.58, 139.15, 137.95-137.83 (d, *J* = 12 Hz), 137.62, 137.50-137.38 (d, *J* = 12 Hz), 136.58, 132.77-132.58 (d, *J* = 20 Hz), 129.56, 129.09, 129.01, 128.91, 128.67, 128.60, 128.53, 128.49, 128.42-128.38 (d, *J* = 4 Hz), 127.40, 126.53, 123.66, 57.35, 48.65-48.49 (d, *J* = 16 Hz), 40.91-40.83 (d, *J* = 8 Hz), 33.12-32.97 (d, *J* = 15 Hz); ^{31}P NMR (400 MHz, $CDCl_3$) δ -23.92; HRMS (ESI): calcd. for $[M+H]^+$ ($C_{36}H_{33}N_3O_4P$) requires 602.2130, found 602.2192.

General procedures for asymmetric cyanation of ketoimines derived from isatins (GP1).

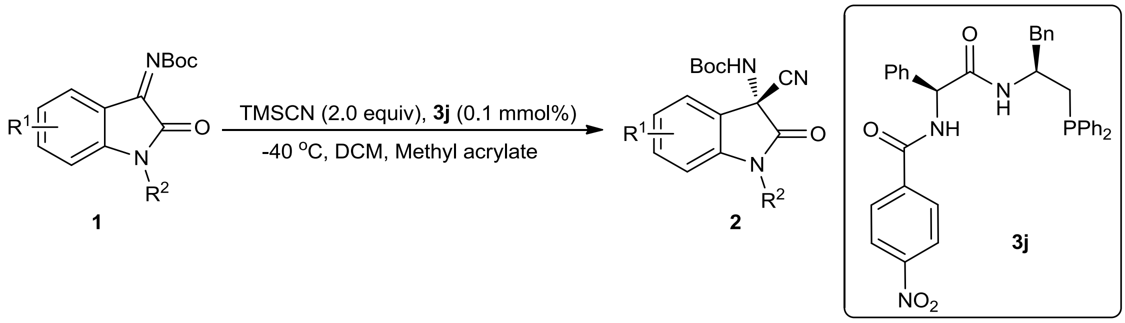

To a vial containing catalyst **3j** (0.1 mol%) , methyl acrylate (0.1 mol%) in CH_2Cl_2 (1 mL) was added TMSCN (0.2 mmol) at -40 °C, and then the ketoimine **1** was added into the mixture. The mixture was stirred until the reaction was completed (minitored by TLC), and then purified by column chromatography on silica gel to afford the product **2**.

(*R*)-*tert*-butyl (1-benzyl-3-cyano-2-oxoindolin-3-yl)carbamate (2a)[4-6]

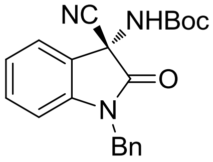

The compound **2a** was prepared according to **GP1** and was purified by flash chromatography (petroleum ether and ethyl acetate). White solid; 98% yield; $[\alpha]_D^{24.0}$ = 80.7 (*c* 0.75, $CHCl_3$); ^{1}H NMR (400 MHz, $CDCl_3$) δ 7.88-7.86 (d, *J* = 8 Hz, 1H), 7.38-7.29 (m, 6H), 7.19-7.15 (t, *J* = 8Hz, 1H), 6.81-6.79 (d, *J* = 8 Hz, 1H), 5.64 (s, 1H), 4.99 (s, 2H), 1.47 (s, 9H).

Enantiometric excess: 97%, determined by HPLC (Chiralpak AS-H column, hexane/*i*-PrOH 85:15, λ=254 nm, flow rate 1.0 mL/min; t_{major} = 32.5 min; t_{minor}= 18.9 min).

(*R*)-tert-butyl (1-allyl-3-cyano-2-oxoindolin-3-yl)carbamate (2b)[4-6]

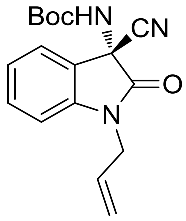

The compound **2b** was prepared according to **GP1** and was purified by flash chromatography (petroleum ether and ethyl acetate). White solid, 97% yield; $[\alpha]_D^{24.8}$ = 59.5 (*c* 1.50, $CHCl_3$);^{1}H NMR (400 MHz, $CDCl_3$) δ 7.85-7.83 (s, *J* = 8 Hz, 1H), 7.40-7.37 (t, *J* = 8 Hz, 1H), 7.18-7.14 (t, *J* = 8 Hz, 1H), 6.90-6.88 (d, *J* = 8 Hz, 1H), 5.86-5.79 (m, 1H), 5.64 (s, 1H), 5.33-5.27 (br, 2H), 4.43-4.32 (br, 2H), 1.42 (s, 9H)

Enantiometric excess: 97%, determined by HPLC (ChiralpakPC-II column, hexane/*i*-PrOH 85:15, λ=254 nm, flow rate 1.0 mL/min; t_{major} = 11.4 min; t_{minor}=9.0 min).

(*R*)-*tert*-butyl (3-cyano-1-methyl-2-oxoindolin-3-yl)carbamate (2c)[4-6]

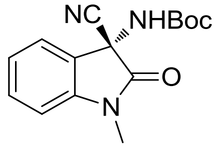

The compound **2c** was prepared according to **GP1** and was purified by flash chromatography (petroleum ether and ethyl acetate). White solid, 99% yield; $[\alpha]_D^{25.2}$ = 112.76 (*c* 1.00, $CHCl_3$); ^{1}H NMR (400 MHz, $CDCl_3$) δ 7.85-7.83 (d, *J* = 8 Hz, 1H), 7.45-7.41 (t, *J* = 8 Hz, 1H), 7.20-7.15 (t, *J* = 8 Hz, 1H), 6.91-6.89 (d, *J* = 8 Hz, 1H), 5.52 (s, 1H), 3.28 (s, 3H), 1.42 (s, 9H).

Enantiometric excess: 97%, determined by HPLC (Chiralpak AD-H column, hexane/*i*-PrOH 85:15, λ=254 nm, flow rate 1.0 mL/min; t_{major} = 13.2 min; t_{minor}= 18.7 min).

(*R*)-*tert*-butyl (3-cyano-1-(4-methoxybenzyl)-2-oxoindolin-3-yl)carbamate (2d)

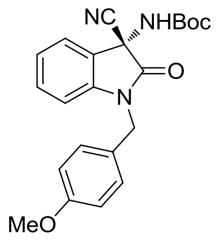

The compound **2d** was prepared according to **GP1** and was purified by flash chromatography (petroleum ether and ethyl acetate). White solid; 99% yield; mp: 145-148 ^{o}C; $[\alpha]_D^{26.3}$ = 79.0 (*c* 1.00, $CHCl_3$); IR (neat): *v* 3320, 2978, 2933, 1868, 1717, 1611, 1514, 1487, 1369, 1177, 1159, 1033, 942 cm^{-1}; ^{1}H NMR (400 MHz, $CDCl_3$) δ 7.63-7.61 (d, *J* = 8 Hz, 1H), 7.11-7.04 (m, 4H), 6.94-6.90 (t, *J* = 8 Hz, 1H), 6.66-6.64 (d, *J* = 8 Hz, 2H), 6.59-6.57 (d, *J* = 8 Hz, 1H), 5.35 (s, 1H), 4.68 (s, 2H), 3.57 (s,

3H), 1.22 (s, 9H); ^{13}C NMR (100 MHz, $CDCl_3$) δ 168.25, 159.47, 153.69, 142.25, 131.20, 128.73, 126.32, 124.86, 124.23, 114.67, 114.46, 110.36, 82.36, 55.30, 54.85, 44.44, 28.13.

HRMS (ESI): calcd. for $[M+Na]^+$ ($C_{22}H_{23}N_3O_4Na$) requires 416.1586, found 416.1590.

Enantiometric excess: 97%, determined by HPLC (Chiralpak AD-H column, hexane/*i*-PrOH 85:15, λ=254 nm, flow rate 1.0 mL/min; t_{major} = 13.8 min; t_{minor}= 12.9 min).

(*R*)-*tert*-butyl (3-cyano-1-(4-nitrobenzyl)-2-oxoindolin-3-yl)carbamate (2e)

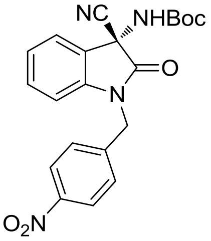

The compound **2e** was prepared according to **GP1** and was purified by flash chromatography (petroleum ether and ethyl acetate). White solid; 99% yield; mp: 94-98 °C; $[\alpha]_D^{24.7}$ = -4.44 (*c* 1.50, $CHCl_3$); IR (neat): *v* 3326, 2978, 2929, 1791, 1717, 1643, 1610, 1558, 1522, 1368, 1344, 1259, 1159, 1016, 938 cm^{-1}; ^{1}H NMR (400 MHz, $CDCl_3$) δ 8.22-8.20 (d, *J* = 8 Hz, 2H), 7.73-7.71 (d, *J* = 8 Hz, 1H), 7.56-7.54 (d, *J* = 8 Hz, 2H), 7.34-7.30 (t, *J* = 8 Hz, 1H), 7.20-7.16 (t, *J* = 8 Hz, 1H), 6.67-6.65 (d, *J* = 8 Hz, 1H), 5.72 (s, 1H), 5.22-5.18 (d, *J* = 16 Hz, 1H), 4.96-4.92 (d, *J* = 16 Hz, 1H), 1.43 (s, 9H); ^{13}C NMR (100 MHz, $CDCl_3$) δ 168.44, 153.35, 147.79, 141.75, 141.68, 131.35, 128.11, 125.62, 124.66, 124.48, 124.22, 114.38, 109.89, 82.88, 44.23, 28.13.

HRMS (ESI): calcd. for $[M+Na]^+$ ($C_{21}H_{20}N_4O_5Na$) requires 431.1331, found 431.1331.

Enantiometric excess: 97%, determined by HPLC (Chiralpak OD-H column, hexane/*i*-PrOH 85:15, λ=254 nm, flow rate 1.0 mL/min; t_{major} = 13.7 min; t_{minor}= 11.5 min).

(*R*)-*tert*-butyl (1-benzyl-3-cyano-5-fluoro-2-oxoindolin-3-yl)carbamate (2f)

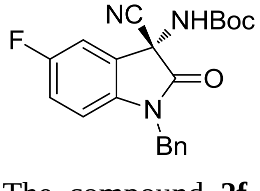

The compound **2f** was prepared according to **GP1** and was purified by flash chromatography (petroleum ether and ethyl acetate). White solid; 98% yield; mp: 66-69 °C; $[\alpha]_D^{26.3}$ = 117.8 (*c* 1.50, $CHCl_3$); IR (neat): *v* 3311, 2979, 2929, 1717, 1618, 1493, 1454, 1393, 1369, 1344, 1269, 1160, 1081, 1018, 948 cm^{-1}; ^{1}H NMR (400 MHz, $CDCl_3$) δ 7.65-7.63 (d, *J* = 8 Hz, 1H), 7.37-7.29 (m, 5H), 7.02-6.98 (t, *J* = 8 Hz, 1H), 6.70-6.67 (m, 1H), 5.61 (s, 1H), 4.95 (s, 2H), 1.46 (s, 9H); ^{13}C NMR (100 MHz, $CDCl_3$) δ 168.04, 161.03, 158.50, 153.67, 138.18, 138.18, 134.03, 129.13, 128.27, 127.19, 117.91 (d, *J* = 23 Hz), 114.85 (d, *J* = 23 Hz), 114.19, 111.19 (d, *J* = 7 Hz), 82.82, 54.89,

45.17, 28.39.

HRMS (ESI): calcd. for $[M+Na]^+$ ($C_{21}H_{20}FN_3O_3Na$) requires 404.1386, found 404.1390.

Enantiometric excess: 92%, determined by HPLC (Chiralpak AD-H column, hexane/*i*-PrOH 85:15, λ=254 nm, flow rate 1.0 mL/min; t_{major} = 7.6 min; t_{minor}= 9.4 min).

(*R*)-*tert*-butyl (1-benzyl-5-chloro-3-cyano-2-oxoindolin-3-yl)carbamate (2g)

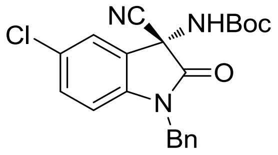

The compound **2g** was prepared according to **GP1** and was purified by flash chromatography (petroleum ether and ethyl acetate). White solid; 99% yield; mp: 86-89 °C; $[\alpha]_D^{25.1}$ = 65.97 (*c* 0.75, $CHCl_3$); IR (neat): *v* 3320, 2979, 2930, 1717, 1646, 1609, 1506, 1485, 1369, 1340, 1257, 1159, 1121, 1018, 946cm^{-1}; ^{1}H NMR (400 MHz, $CDCl_3$) δ 7.83 (s, 1H), 7.36-7.29 (m, 7H), 6.71-6.69 (d, *J* = 8 Hz, 1H), 5.92-5.88 (br, 1H), 4.98 (s, 2H), 1.48 (s, 9H); ^{13}C NMR (100 MHz, $CDCl_3$) δ 167.89, 153.54, 140.75, 133.83, 131.24, 129.81 129.13, 128.35, 127.18, 126.68, 126.06, 114.06, 111.36, 82.81, 54.69, 45.09, 28.14.

HRMS (ESI): calcd. for $[M+Na]^+$ ($C_{21}H_{20}ClN_3O_3Na$) requires 420.1091, found 420.1093.

Enantiometric excess: 93%, determined by HPLC (Chiralpak AD-H column, hexane/*i*-PrOH 85:15, λ=254 nm, flow rate 1.0 mL/min; t_{major} = 6.9 min; t_{minor}= 10.8 min).

(*R*)-*tert*-butyl (1-benzyl-5-bromo-3-cyano-2-oxoindolin-3-yl)carbamate (2h)

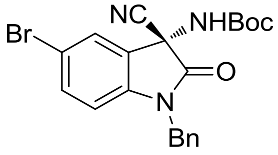

The compound **2h** was prepared according to **GP1** and was purified by flash chromatography (petroleum ether and ethyl acetate). White solid; 98% yield; mp: 109-111 °C; $[\alpha]_D^{24.3}$ = 53.0 (*c* 1.50, $CHCl_3$); IR (neat): *v* 3320, 2977, 2930, 1717, 1635, 1607, 1497, 1482, 1456, 1369, 1340, 1259, 1158, 1081, 1018, 945 cm^{-1}; ^{1}H NMR (400 MHz, $CDCl_3$) δ 7.95 (s, 1H), 7.42-7.40 (d, *J* = 8 Hz, 1H), 7.36-7.28 (m, 5H), 6.63-6.61 (d, *J* = 8 Hz, 1H), 5.59 (s, 1H), 4.94 (s, 2H), 1.45 (s, 9H); ^{13}C NMR (100 MHz, $CDCl_3$) δ 167.81, 153.54, 141.25, 134.16, 133.80, 129.31, 129.13, 128.29, 127.19, 126.35, 116.93, 114.08, 111.83, 82.83, 54.62, 45.06, 28.15.

HRMS (ESI): calcd. for $[M+Na]^+$ ($C_{21}H_{20}BrN_3O_3Na$) requires 464.0586, found 464.0587.

Enantiometric excess: 90%, determined by HPLC (Chiralpak AD-H column, hexane/*i*-PrOH 85:15, λ=254 nm, flow rate 1.0 mL/min; t_{major} = 7.2 min; t_{minor}= 12.3 min).

(*R*)-*tert*-butyl (1-benzyl-3-cyano-5-iodo-2-oxoindolin-3-yl)carbamate (2i)

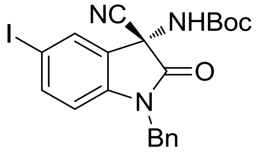

The compound **2i** was prepared according to **GP1** and was purified by flash chromatography (petroleum ether and ethyl acetate). White solid; 97% yield; mp: 74-76 °C; $[\alpha]_D^{24.8}$ = 46.7 (*c* 1.50, $CHCl_3$); IR (neat): *ν* 3319, 2978, 2928, 1717, 1603, 1480, 1455, 1420, 1369, 1341, 1258, 1158, 1045, 1018, 944cm^{-1}; ^{1}H NMR (400 MHz, $CDCl_3$) δ 8.13 (s, 1H), 7.65-7.63 (d, *J* = 8 Hz, 1H), 7.40-7.32 (m, 5H), 6.56-6.54 (d, *J* = 4 Hz, 1H), 5.65 (s, 1H), 4.97 (s, 2H), 1.49 (s, 9H); ^{13}C NMR (100 MHz, $CDCl_3$) δ 167.66, 153.52, 141.94, 140.08, 134.67, 133.82, 129.13, 128.28, 127.20, 126.56, 114.06, 112.32, 86.64, 82.78, 54.44, 45.44, 45.01, 28.16.

HRMS (ESI): calcd. for $[M+Na]^+$ ($C_{21}H_{20}IN_3O_3Na$) requires 512.0447, found 512.0440.

Enantiometric excess: 93%, determined by HPLC (Chiralpak AD-H column, hexane/*i*-PrOH 85:15, λ=254 nm, flow rate 1.0 mL/min; t_{major} = 8.2 min; t_{minor}= 14.5 min).

(*R*)-*tert*-butyl (1-benzyl-3-cyano-5-methyl-2-oxoindolin-3-yl)carbamate (2j)

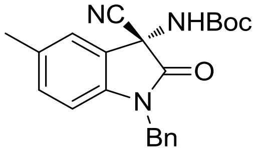

The compound **2j** was prepared according to **GP1** and was purified by flash chromatography (petroleum ether and ethyl acetate). White solid; 98% yield; mp: 75-77 °C; $[\alpha]_D^{24.0}$ = 103.4 (*c* 1.00, $CHCl_3$); IR (neat): *ν* 3319, 2966, 2927, 1717, 1618, 1604, 1497, 1455, 1368, 1345, 1260, 1160, 1082, 1018, 946cm^{-1}; ^{1}H NMR (400 MHz, $CDCl_3$) δ 7.65 (s, 1H), 7.35-7.27 (m, 5H), 7.09-7.07 (d, *J* = 8 Hz, 1H), 6.64-6.62 (d, *J* = 8 Hz, 1H), 5.54 (s, 1H), 4.93 (s, 2H), 2.32 (s, 3H), 1.44 (s, 9H); ^{13}C NMR (100 MHz, $CDCl_3$) δ 168.22, 153.70, 139.74, 134.38, 134.22, 131.53, 129.01, 128.07, 127.22, 126.91, 124.73, 114.75, 110.09, 82.36, 54.93, 44.91, 28.17, 21.07.

HRMS (ESI): calcd. for $[M+Na]^+$ ($C_{22}H_{23}N_3O_3Na$) requires 400.1637, found 400.1638.

Enantiometric excess: 96%, determined by HPLC (Chiralpak AD-H column, hexane/*i*-PrOH 85:15, λ=254 nm, flow rate 1.0 mL/min; t_{major} = 9.0 min; t_{minor}= 11.0 min).

(*R*)-*tert*-butyl (1-benzyl-3-cyano-5-methoxy-2-oxoindolin-3-yl)carbamate (2k)

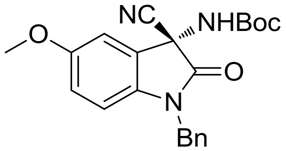

The compound **2k** was prepared according to **GP1** and was purified by flash chromatography (petroleum ether and ethyl acetate). White solid; 99% yield; mp: 62-65 °C; $[\alpha]_D^{26.4}$ = 78.6 (*c* 1.50, $CHCl_3$); IR (neat): *v* 3310, 2977, 2931, 1716, 1635, 1539, 1497, 1456, 1368, 1275, 1160, 1041, 1017, 948 cm^{-1}; ^{1}H NMR (400 MHz, $CDCl_3$) δ 7.52 (s, 1H), 7.37-7.32 (m, 5H), 6.86 (q, *J* = 8 Hz, *J* = 4 Hz, 1H), 6.69-6.67 (d, *J* = 8 Hz, 1H), 5.61 (s, 1H), 4.96 (s, 2H), 3.81 (s, 3H), 1.49 (s, 9H); ^{13}C NMR (100 MHz, $CDCl_3$) δ 167.98, 157.01, 153.75, 135.33, 134.34, 129.03, 128.10, 127.22, 125.85, 116.37, 114.65, 112.97, 110.97, 82.56, 55.92, 55.10, 44.99, 28.15.

HRMS (ESI): calcd. for $[M+Na]^+$ ($C_{22}H_{23}N_3O_4Na$) requires 416.1586, found 416.1582.

Enantiometric excess: 97%, determined by HPLC (Chiralpak AS-H column, hexane/*i*-PrOH 85:15, λ=254 nm, flow rate 1.0 mL/min; t_{major} = 30.7 min; t_{minor}= 24.2 min).

(*R*)-*tert*-butyl (1-benzyl-6-chloro-3-cyano-2-oxoindolin-3-yl)carbamate (2l)

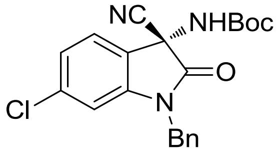

The compound **2l** was prepared according to **GP1** and was purified by flash chromatography (petroleum ether and ethyl acetate). White solid; 98% yield; mp: 112-114 °C; $[\alpha]_D^{26.3}$ = 52.7 (*c* 1.50, $CHCl_3$); IR (neat): *v* 3309, 2978, 2928, 1718, 1608, 1487, 1455, 1441, 1368, 1279, 1255, 1159, 1075, 1045, 1018, 882 cm^{-1}; ^{1}H NMR (400 MHz, $CDCl_3$) δ 7.76-7.74 (d, *J* = 8 Hz, 1H), 7.39-7.31 (m, 5H), 7.13-7.11 (d, *J* = 8 Hz, 1H), 6.76 (s, 1H), 5.58 (s, 1H), 4.94 (s, 1H), 1.44 (s, 9H); ^{13}C NMR (100 MHz, $CDCl_3$) δ 168.28, 153.57, 143.44, 137.29, 133.75, 129.18, 128.35, 127.21, 124.29, 123.00, 82.69, 54.42, 45.08, 28.13.

HRMS (ESI): calcd. for $[M+Na]^+$ ($C_{21}H_{20}ClN_3O_3Na$) requires 420.1091, found 420.1090.

Enantiometric excess: 95%, determined by HPLC (Chiralpak AS-H column, hexane/*i*-PrOH 85:15, λ=254 nm, flow rate 1.0 mL/min; t_{major} = 15.6 min; t_{minor}= 11.1 min).

(*R*)-*tert*-butyl (1-benzyl-6-bromo-3-cyano-2-oxoindolin-3-yl)carbamate (2m)

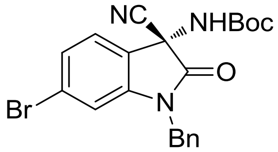

The compound **2m** was prepared according to **GP1** and was purified by flash chromatography (petroleum ether and ethyl acetate). White solid; 97% yield; mp: 135-137 °C; $[\alpha]_D^{24.1}$ = 65.1 (*c* 1.50, $CHCl_3$); IR (neat): *v* 3312, 2979, 2929, 1716, 1604, 1486, 1455, 1369, 1279, 1158, 1061, 1018, 981 cm^{-1}; ^{1}H NMR (400 MHz, $CDCl_3$) δ 7.69-7.67 (d, *J* = 8 Hz, 1H), 7.38-7.28 (m, 6H), 6.90 (s, 1H), 5.58 (s, 1H), 4.92 (s, 2H), 1.43 (s, 9H); ^{13}C NMR (100 MHz, $CDCl_3$) δ 168.15, 153.50, 143.47, 133.73, 129.18, 128.35, 127.48, 127.27, 125.21, 123.54, 114.05, 113.73, 82.70, 54.47, 45.07, 28.12.
HRMS (ESI): calcd. for $[M+Na]^+$ ($C_{21}H_{20}BrN_3O_3Na$) requires 464.0586, found 464.0582.
Enantiometric excess: 95%, determined by HPLC (Chiralpak AS-H column, hexane/*i*-PrOH 85:15, λ=254 nm, flow rate 1.0 mL/min; t_{major} = 16.5 min; t_{minor}= 12.3 min).

(*R*)-*tert*-butyl (1-benzyl-3-cyano-7-methyl-2-oxoindolin-3-yl)carbamate (2n)

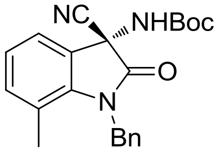

The compound **2n** was prepared according to **GP1** and was purified by flash chromatography (petroleum ether and ethyl acetate). White solid; 98% yield; mp: 77-79 °C; $[\alpha]_D^{24.9}$ = 68.9 (*c* 1.00, $CHCl_3$); IR (neat): *v* 3318, 2978, 2930, 1717, 1601, 1497, 1453, 1419, 1392, 1368, 1253, 1159, 1081, 1017, 950cm^{-1}; ^{1}H NMR (400 MHz, $CDCl_3$) δ 7.52 (br, 1H), 7.21-7.17 (t, *J* = 8 Hz, 2H), 7.13-7.10 (br, 2H), 7.07-7.05 (br, 1H), 6.91-6.90 (br, 2H), 5.47 (s, 1H), 5.07 (s, 2H), 2.10 (s, 3H), 1.29 (s, 9H); ^{13}C NMR (100 MHz, $CDCl_3$) δ 169.36, 153.51, 140.30, 136.21, 135.23, 129.10, 127.62, 125.61, 124.34, 123.81, 121.09, 114.71, 82.37, 54.55, 46.26, 28.15, 18.66.
HRMS (ESI): calcd. for $[M+Na]^+$ ($C_{22}H_{23}N_3O_3Na$) requires 400.1637, found 400.1638.
Enantiometric excess: 99%, determined by HPLC (Chiralpak AD-H column, hexane/*i*-PrOH 85:15, λ=254 nm, flow rate 1.0 mL/min; t_{major} = 11.2 min; t_{minor}=9.6 min).

(*R*)-*tert*-butyl (1-benzyl-7-chloro-3-cyano-2-oxoindolin-3-yl)carbamate (2o)[4-6]

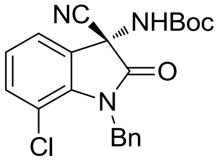

The compound **2o** was prepared according to **GP1** and was purified by flash chromatography (petroleum ether and ethyl acetate). White solid; 99% yield; $[\alpha]_D^{24.6}$ = 56.7 (*c* 1.50, $CHCl_3$); ^{1}H NMR (400 MHz, $CDCl_3$) δ 7.78-7.76 (d, *J* = 8 Hz, 1H), 7.38-7.29 (m, 6H), 7.15-7.11 (t, *J* = 8 Hz, 1H), 5.69 (s, 1H), 5.43 (s, 2H), 1.47 (s, 9H).
Enantiometric excess: 90%, determined by HPLC (Chiralpak AD-Hcolumn, hexane/*i*-PrOH 85:15,

λ=254 nm, flow rate 1.0 mL/min; t_{major} = 9.1 min; t_{minor}=8.5 min).

(*R*)-*tert*-butyl (1-benzyl-7-bromo-3-cyano-2-oxoindolin-3-yl)carbamate (2p)

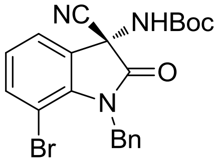

The compound **2p** was prepared according to **GP1** and was purified by flash chromatography (petroleum ether and ethyl acetate). White solid; 98% yield; mp: 128-130 °C; $[\alpha]_D^{26.6}$ = 54.1 (*c* 1.50, $CHCl_3$); IR (neat): *v* 3264, 2975, 2929, 1716, 1605, 1558, 1506, 1497, 1465, 1368, 1282, 1254, 1162, 1017, 972 cm^{-1}; ^{1}H NMR (400 MHz, $CDCl_3$) δ 7.72-7.70 (d, *J* = 8 Hz, 1H), 7.42-7.40 (d, *J* = 8 Hz, 1H), 7.28-7.24 (br, 2H), 7.20-7.19 (br, 4H), 6.98-6.94 (t, *J* = 8 Hz, 1H), 5.56 (s, 1H), 5.37 (s, 2H), 1.36 (s, 9H); ^{13}C NMR (100 MHz, $CDCl_3$) δ 169.13, 153.38, 139.94, 137.25, 136.02, 128.77, 127.64, 127.48, 126.25, 125.46, 125.03, 114.07, 103.39, 82.75, 54.46, 45.92, 28.11.
HRMS (ESI): calcd. for $[M+Na]^+$ ($C_{21}H_{20}BrN_3O_3Na$) requires 464.0586, found 464.0582.
Enantiometric excess: 91%, determined by HPLC (Chiralpak AD-H column, hexane/*i*-PrOH 85:15, λ=254 nm, flow rate 1.0 mL/min; t_{major} = 9.8 min; t_{minor}= 8.6 min).

General procedures for asymmetric cyanation of azomethineimines (GP2).

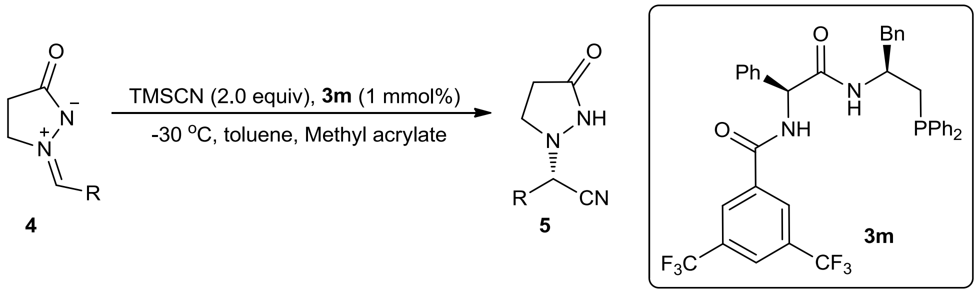

To a vial containing catalyst **3n** (1 mol%) , methyl acrylate (1 mol%) in toluene (1 mL) was added TMSCN (0.2 mmol) at -30 °C, and then the aldimine was added into the mixture. The mixture was stirred until the reaction was completed (minitored by TLC), and then purified by column chromatography on silica gel to afford the product **5**.

(*S*)-2-(3-oxopyrazolidin-1-yl)-2-phenylacetonitrile (5a)[7-11]

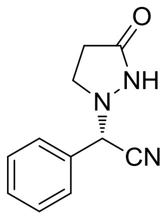

The compound **5a** was prepared according to **GP2** and was purified by flash chromatography (petroleum ether and ethyl acetate). White solid; 98% yield; $[\alpha]_D^{26.3}$ = -110.2 (c 1.00, $CHCl_3$); ^{1}H NMR (400 MHz, $CDCl_3$) δ 7.55-7.52 (m, 2H), 7.46-7.44 (m, 3H), 4.96 (s, 1H), 3.58 (br, 1H), 3.53-3.46 (m, 1H), 2.65 (br, 2H);
Enantiometric excess: 91%, determined by HPLC (ChiralpakPC-4 column, hexane/*i*-PrOH 82:18, λ=254 nm, flow rate 1.0 mL/min; t_{major} = 31.7 min; t_{minor}=30.8 min).

(*S*)-2-(2-fluorophenyl)-2-(3-oxopyrazolidin-1-yl)acetonitrile(5b)[7-11]

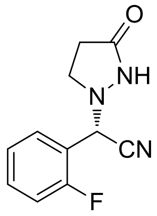

The compound **5b** was prepared according to **GP2** and was purified by flash chromatography (petroleum ether and ethyl acetate). White solid; 97% yield; $[\alpha]_D^{26.3}$ = -90.6 (c 1.00, $CHCl_3$); ^{1}H NMR (400 MHz, $CDCl_3$) δ 8.19 (br, 1H), 7.63-7.59 (dt, *J* = 8 Hz, J = 2 Hz, 1H), 7.47-7.41 (m, 1H), 7.26-7.22 (t, *J* = 8 Hz, 1H), 7.16-7.11 (t, *J* = 8 Hz, 1H), 5.19 (s, 1H), 3.66-3.59 (m, 1H), 3.51-3.44 (m, 1H), 2.67 (br, 1H), 2.55-2.47 (m, 1H)
Enantiometric excess: 91%, determined by HPLC (Chiralpak AD-H column, hexane/*i*-PrOH 85:15, λ=254 nm, flow rate 1.0 mL/min; t_{major} = 19.0 min; t_{minor}=13.0 min).

(*S*)-2-(2-methoxyphenyl)-2-(3-oxopyrazolidin-1-yl)acetonitrile(5c)[7-11]

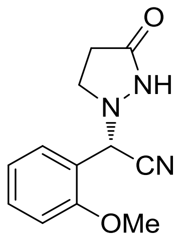

The compound **5c** was prepared according to **GP2** and was purified by flash chromatography (petroleum ether and ethyl acetate). White solid; 95% yield; $[\alpha]_D^{26.2}$ = -83.9 (c 0.50, $CHCl_3$); ^{1}H

NMR (400 MHz, $CDCl_3$) δ 8.15 (br, 1H), 7.52-7.51 (d, *J* = 8 Hz, 1H), 7.40-7.36 (t, *J* = 8 Hz, 1H), 7.02-6.98 (t, *J* = 8 Hz, 1H), 6.93-6.91 (d, *J* = 8 Hz, 1H), 5.29 (s, 1H), 3.86 (s, 3H), 3.63-3.56 (m, 1H), 3.46-3.39 (m, 1H), 2.63 (br, 1H), 2.53-2.45 (m, 1H);

Enantiometric excess: 93%, determined by HPLC (Chiralpak AD-H column, hexane/*i*-PrOH 85:15, λ=254 nm, flow rate 1.0 mL/min; t_{major} = 35.1 min; t_{minor}= 15.6 min).

(*S*)-2-(2-bromophenyl)-2-(3-oxopyrazolidin-1-yl)acetonitrile(5d)[7-11]

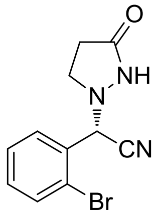

The compound **5d** was prepared according to **GP2** and was purified by flash chromatography (petroleum ether and ethyl acetate). White solid; 98% yield; $[\alpha]_D^{24.5}$ = -109.7 (c 1.00, $CHCl_3$); ^1H NMR (400 MHz, $CDCl_3$) δ 8.06 (br, 1H), 7.70-7.68 (d, *J* = 8 Hz, 1H), 7.65-7.63 (d, *J* = 8 Hz, 1H), 7.44-7.40 (t, *J* = 8 Hz, 1H), 7.32-7.28 (t, *J* = 8 Hz, 1H), 5.30 (s, 1H), 3.70-3.63 (m, 1H), 3.48-3.42 (m, 1H), 2.80 (br, 1H), 2.62-2.55 (m, 1H);

Enantiometric excess: 93%, determined by HPLC (Chiralpak AD-H column, hexane/*i*-PrOH 85:15, λ=254 nm, flow rate 1.0 mL/min; t_{major} = 15.7 min; t_{minor}=10.9 min).

(*S*)-2-(3-chlorophenyl)-2-(3-oxopyrazolidin-1-yl)acetonitrile (5e)[7-11]

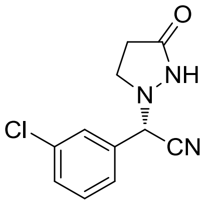

The compound **5e** was prepared according to **GP2** and was purified by flash chromatography (petroleum ether and ethyl acetate). White solid, 91% yield; $[\alpha]_D^{24.8}$ = -111.7 (c 0.75, $CHCl_3$); ^1H NMR (400 MHz, $CDCl_3$) δ 7.52 (s, 1H), 7.43-7.34 (m, 3H), 4.95 (s, 1H), 3.49 (br, 1H), 3.47-3.42 (m, 1H), 2.67 (br, 2H);

Enantiometric excess: 91%, determined by HPLC (Chiralpak AD-H column, hexane/*i*-PrOH 85:15, λ=254 nm, flow rate 1.0 mL/min; t_{major} = 11.8 min; t_{minor}= 10.3 min).

(*S*)-2-(3-bromophenyl)-2-(3-oxopyrazolidin-1-yl)acetonitrile(5f)[7-11]

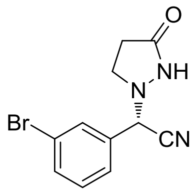

The compound **5f** was prepared according to **GP2** and was purified by flash chromatography (petroleum ether and ethyl acetate). White solid; 97% yield; $[\alpha]_D^{23.9}$ = -94.3 (c 0.75, $CHCl_3$); ^1H NMR (400 MHz, $CDCl_3$) δ 8.19 (br, 1H), 7.69 (s, 1H), 7.56-7.54 (d, *J* = 8 Hz, 1H), 7.48-7.46 (d, *J* = 8 Hz, 1H), 7.34-7.28 (m, 1H), 4.96 (s, 1H), 3.53-3.44 (m, 2H), 2.73-2.57 (m, 2H);

Enantiometric excess: 90%, determined by HPLC (Chiralpak AD-H column, hexane/*i*-PrOH 85:15, λ=254 nm, flow rate 1.0 mL/min; t_{major} = 11.5 min; t_{minor}=10.2 min).

(*S*)-2-(4-fluorophenyl)-2-(3-oxopyrazolidin-1-yl)acetonitrile(5g)[7-11]

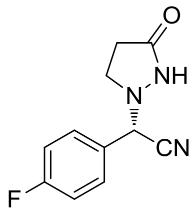

The compound **5g** was prepared according to **GP2** and was purified by flash chromatography (petroleum ether and ethyl acetate). White solid; 94% yield; $[\alpha]_D^{26.2}$ = -99.3 (c 1.00, $CHCl_3$); ^1H NMR (400 MHz, $CDCl_3$) δ 8.42 (br, 1H), 7.52 (d, *J* = 8 Hz, 1H), 7.49 (d, *J* = 8 Hz, 1H), 7.14-7.10 (t, *J* = 8 Hz, 2H), 4.93 (s, 1H), 3.51 (br, 1H), 3.47-3.40 (m, 1H), 2.62 (br, 2H);

Enantiometric excess: 95%, determined by HPLC (Chiralpak AD-H column, hexane/*i*-PrOH 85:15, λ=254 nm, flow rate 1.0 mL/min; t_{major} = 10.8 min; t_{minor}= 15.1 min).

(*S*)-2-(4-chlorophenyl)-2-(3-oxopyrazolidin-1-yl)acetonitrile(5h)[7-11]

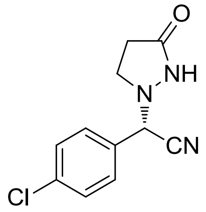

The compound **5h** was prepared according to **GP2** and was purified by flash chromatography (petroleum ether and ethyl acetate). White solid; 97% yield; $[\alpha]_D^{26.3}$ = -102.3 (c 0.75, $CHCl_3$); ^1H NMR (400 MHz, $CDCl_3$) δ 7.49-7.41 (m, 4H), 4.92 (s, 1H), 3.56 (br, 1H), 3.51-3.44 (m, 1H), 2.73-2.70 (m, 1H), 2.66 (br, 1H);

Enantiometric excess: 93%, determined by HPLC (Chiralpak AD-H column, hexane/*i*-PrOH 85:15,

λ=254 nm, flow rate 1.0 mL/min; t_{major} = 10.6 min; t_{minor}= 15.7 min).

(*S*)-2-(4-bromophenyl)-2-(3-oxopyrazolidin-1-yl)acetonitrile(5i)[7-11]

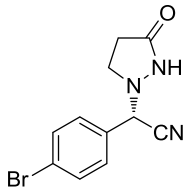

The compound **5i** was prepared according to **GP2** and was purified by flash chromatography (petroleum ether and ethyl acetate). White solid; 96% yield; $[\alpha]_D^{24.7}$ = -81.4 (c 1.00, $CHCl_3$); ^1H NMR (400 MHz, $CDCl_3$) δ 7.58-7.56 (d, *J* = 8 Hz, 2H), 7.41-7.39 (d, *J* = 8 Hz, 2H), 4.88 (s, 1H), 3.58 (br, 1H), 3.52-3.46 (m, 1H), 2.73-2.69 (m, 1H), 2.66 (br, 1H);

Enantiometric excess: 93%, determined by HPLC (Chiralpak AD-H column, hexane/*i*-PrOH 85:15, λ=254 nm, flow rate 1.0 mL/min; t_{major} = 10.8 min; t_{minor}= 16.0 min).

(*S*)-2-(4-methoxyphenyl)-2-(3-oxopyrazolidin-1-yl)acetonitrile(5j)[7-11]

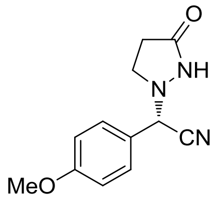

The compound **5j** was prepared according to **GP2** and was purified by flash chromatography (petroleum ether and ethyl acetate). White solid; 95% yield; $[\alpha]_D^{26.2}$ = -80.7 (c 1.25, $CHCl_3$); ^1H NMR (400 MHz, $CDCl_3$) δ 7.44-7.42 (d, *J* = 8 Hz, 2H), 6.95-6.93 (d, *J* = 8 Hz, 2H), 4.86 (s, 1H), 3.83 (s, 3H), 3.57 (br, 1H), 3.51-3.44 (m, 1H), 2.61 (br, 2H);

Enantiometric excess: 93%, determined by HPLC (Chiralpak AD-H column, hexane/*i*-PrOH 85:15, λ=254 nm, flow rate 1.0 mL/min; t_{major} = 12.1 min; t_{minor}= 10.5 min).

(*S*)-2-(3-oxopyrazolidin-1-yl)-2-(p-tolyl)acetonitrile(5k)

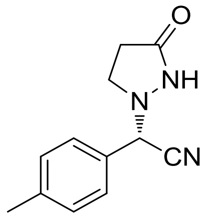

The compound **5k** was prepared according to **GP2** and was purified by flash chromatography (petroleum ether and ethyl acetate). White solid, m.p. 122℃-125℃;$[\alpha]_D^{23.8}$ = -105.9 (c 1.00, $CHCl_3$); ^1H NMR (400 MHz, $CDCl_3$) δ 8.04 (br, 1H), 7.40-7.38 (d, *J* = 8 Hz, 2H), 7.24-7.22 (d, *J* = 8 Hz, 2H),

4.90 (s, 1H), 3.53 (br, 1H), 3.48-3.41 (m, 1H), 2.62 (br, 2H), 2.37 (s, 3H); ^{13}C NMR (100 MHz, $CDCl_3$) δ 175.23, 139.98, 129.87, 128.18, 128.05, 116.95, 62.62, 29.72, 21.19;
HRMS (ESI): calcd. for $[M+H]^+$ ($C_{12}H_{14}N_3O$) requires 216.1059, found 216.1131;
Enantiometric excess: 93%, determined by HPLC (Chiralpak AD-H column, hexane/*i*-PrOH 85:15, λ=254 nm, flow rate 1.0 mL/min; t_{major} = 9.4 min; t_{minor}= 12.6 min).

(*S*)-2-(3-oxopyrazolidin-1-yl)-2-(3-(trifluoromethyl)phenyl)acetonitrile(5l)

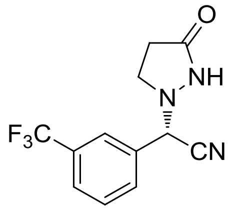

The compound **5l** was prepared according to **GP2** and was purified by flash chromatography (petroleum ether and ethyl acetate). Brown oil; $[\alpha]_D^{24.2}$ = -45.8 (c 0.75, $CHCl_3$); ^{1}H NMR (400 MHz, $CDCl_3$) δ 8.47 (br, 1H), 7.80 (s, 1H), 7.77-7.75 (d, *J* = 8 Hz, 1H), 7.72-7.70 (d, *J* = 8 Hz, 1H), 7.62-7.58 (t, *J* = 8 Hz, 1H), 5.03 (s, 1H), 3.56-3.45 (m, 2H), 2.69 (br, 2H); ^{13}C NMR (100 MHz, $CDCl_3$) δ 175.47, 132.27, 132.23 (q, *J* = 33 Hz), 131.52, 129.86, 129.61 (q, *J* = 271 Hz), 126.80 (q, *J* = 40 Hz), 116.22, 62.56, 29.64;
HRMS (ESI): calcd. for $[M+H]^+$ ($C_{12}H_{10}F_3N_3O$) requires 270.0776, found 270.0847;
Enantiometric excess: 91%, determined by HPLC (Chiralpak AD-H column, hexane/*i*-PrOH 85:15, λ=254 nm, flow rate 1.0 mL/min; t_{major} = 7.7 min; t_{minor}=6.6 min).

(*S*)-2-(naphthalen-1-yl)-2-(3-oxopyrazolidin-1-yl)acetonitrile(5m)

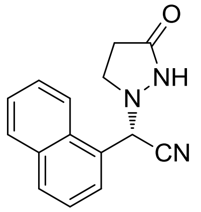

The compound **5m** was prepared according to **GP2** and was purified by flash chromatography (petroleum ether and ethyl acetate). White solid, 95℃-97℃;$[\alpha]_D^{23.8}$ = -109.6 (c 0.75, $CHCl_3$); ^{1}H NMR (400 MHz, $CDCl_3$) δ 8.12-8.10 (d, *J* = 8 Hz, 1H), 7.96-7.94 (d, *J* = 8 Hz, 1H), 7.92-7.90 (d, *J* = 8 Hz, 1H), 7.86-7.84 (d, *J* = 8 Hz, 1H), 7.63-7.49 (m, 3H), 5.64 (s, 1H), 3.70 (br, 1H), 3.52-3.45 (m, 1H), 2.80 (br, 1H), 2.71 (br, 1H); ^{13}C NMR (100 MHz, $CDCl_3$) δ 175.05, 133.96, 131.12, 130.40,

128.99, 127.58, 127.27, 126.62, 126.47, 125.02, 123.06, 116.90, 61.19, 29.79;
HRMS (ESI): calcd. for $[M+H]^+$ ($C_{15}H_{14}N_3O$) requires 252.1059, found 252.1130;
Enantiometric excess: 95%, determined by HPLC (Chiralpak AD-H column, hexane/*i*-PrOH 85:15, λ=254 nm, flow rate 1.0 mL/min; t_{major} = 22.3 min; t_{minor}=11.6 min).

(*S*)-2-(furan-2-yl)-2-(3-oxopyrazolidin-1-yl)acetonitrile(5n)[7-11]

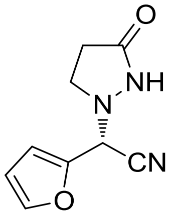

The compound **5n** was prepared according to **GP2** and was purified by flash chromatography (petroleum ether and ethyl acetate). White solid; 92% yield; $[\alpha]_D^{26.3}$ = -128.4 (c 1.50, $CHCl_3$); ^1H NMR (400 MHz, $CDCl_3$) δ 8.22 (br, 1H), 7.49-7.48 (m, 1H), 6.62-6.61 (d, *J* = 4 Hz, 1H), 6.44-6.43 (dd, *J* = 4 Hz, 1H), 4.97 (s, 1H), 3.65-3.62 (br, 1H), 3.56-3.48 (m, 1H), 2.52 (br, 2H);
Enantiometric excess: 95%, determined by HPLC (Chiralpak AD-H column, hexane/*i*-PrOH 85:15, λ=254 nm, flow rate 1.0 mL/min; t_{major} = 14.0 min; t_{minor}=29.1 min).

(*S*)-2-cyclohexyl-2-(3-oxopyrazolidin-1-yl)acetonitrile(5o)[7-11]

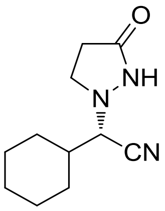

The compound **5o** was prepared according to **GP2** and was purified by flash chromatography (petroleum ether and ethyl acetate). White solid; 99% yield; $[\alpha]_D^{26.3}$ = -23.5 (c 1.00, $CHCl_3$); ^1H NMR (400 MHz, $CDCl_3$) δ 9.22 (s, 1H), 3.61-3.53 (dd, *J* = 8 Hz, 1H), 3.47-3.40 (dd, *J* = 8 Hz, 1H), 3.38-3.36 (d, *J* = 8 Hz, 1H), 2.78 (br, 1H), 2.64 (br, 1H), 2.02-1.99 (d, *J* = 12 Hz, 2H), 1.79-1.62 (m, 4H), 1.35-0.93 (m, 5H);
Enantiometric excess: 93%, determined by HPLC (Chiralpak AD-H column, hexane/*i*-PrOH 85:15, λ=254 nm, flow rate 1.0 mL/min; t_{major} = 7.11 min; t_{minor}=13.4 min).

General procedures for asymmetric kenetic resolution of azomethineimines (GP3).

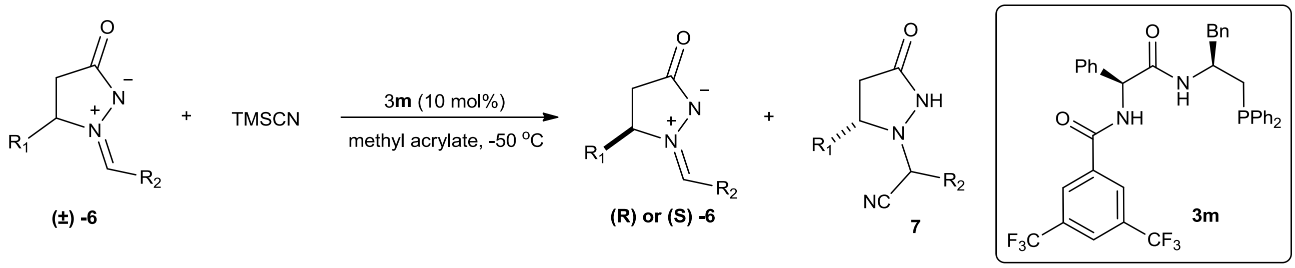

To a vial containing catalyst **3m** (1 mol%) , methyl acrylate (1 mol%) in toluene (1 mL) was added TMSCN (0.12mmol) at -30 °C, and then the aldimine**6** (0.2 mmol) was added into the mixture. The mixture was stirred until the reaction was completed (minitored by TLC), and then purified by column chromatography on silica gel to afford the product **6** and **7.**

(*R*, *Z*)-2-benzylidene-5-oxo-3-phenylpyrazolidin-2-ium-1-ide (6a)[7-11]

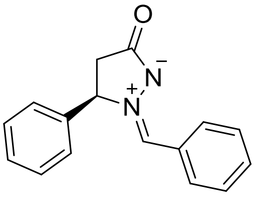

The compound **6a** was prepared according to **GP3** and was purified by flash chromatography (petroleum ether and ethyl acetate). White solid; 48% yield; $[\alpha]_D^{27.3}$ = 14.4 (c 1.00, $CHCl_3$); ^{1}H NMR (400 MHz, $CDCl_3$) δ 8.27-8.24 (br, 2H), 7.46-7.41 (m, 6H), 7.37-7.35 (m, 2H), 6.84 (s, 1H), 5.57-5.53 (q, 1H), 3.33-3.26 (dd, *J* = 16 Hz, *J* = 8 Hz, 1H), 2.89-2.83 (dd, *J* = 16 Hz, *J* = 8 Hz, 1H); Enantiometric excess: 80%, determined by HPLC (Chiralpak AD-H column, hexane/*i*-PrOH 80:20, λ=254 nm, flow rate 1.0 mL/min; t_{major} = 12.8 min; t_{minor}= 15.1 min).

(*S*)-2-((*S*)-3-oxo-5-phenylpyrazolidin-1-yl)-2-phenylacetonitrile (7a)

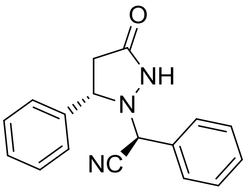

The compound **7a** was prepared according to **GP3** and was purified by flash chromatography (CH_2Cl_2 and Methanol). White solid; mp. 48-50 °C; 49% yield; $[\alpha]_D^{27.3}$ = -149.9 (c 1.00, $CHCl_3$); IR (neat): *v*3174, 3063, 2924, 2853, 1698, 1494, 1453, 1266 cm^{-1};^{1}H NMR (400 MHz, $CDCl_3$) δ 7.46-7.28 (m, 10H), 6.97 (s, 1H), 4.85 (s, 1H), 4.52-4.48 (t, *J* = 8 Hz, 1H), 3.11-3.04 (dd, *J* = 16 Hz, *J* = 8 Hz, 1H), 2.55-2.52 (dd, *J* = 16 Hz, *J* = 8 Hz, 1H); ^{13}C NMR (100 MHz, $CDCl_3$) δ 173..04, 137.90, 131.52, 129.93, 129.38, 129.24, 128.92, 127.94, 127.20, 115.18, 65.86, 60.54, 39.69;

HRMS (ESI): calcd. for $[M+H]^+$ ($C_{17}H_{16}N_3O$) requires 278.1215, found 278.1285 ;
Enantiometric excess: 79%, determined by HPLC (ChiralpakPC-II column, hexane/*i*-PrOH 80:20, λ=254 nm, flow rate 1.0 mL/min; t_{major} = 21.4 min; t_{minor}= 16.3 min).

(*R*, *Z*)-2-(4-bromobenzylidene)-3-(4-methoxyphenyl)-5-oxopyrazolidin-2-ium-1-ide (6b)

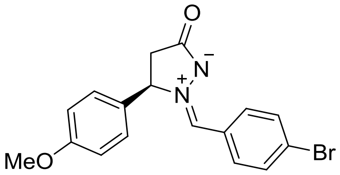

The compound **6b** was prepared according to **GP3** and was purified by flash chromatography (petroleum ether and ethyl acetate). White solid; mp. 64-67 °C; 49% yield; $[\alpha]_D^{27.5}$ = -16.3 (c 1.50, $CHCl_3$); IR (neat): *v*3052, 2928, 2837, 1677, 1586, 1554, 1422, 1302, 1252 cm^{-1}; ^1H NMR (400 MHz, $CDCl_3$) δ 8.04-8.02 (d, *J* = 8 Hz, 2H), 7.47-7.45 (d, *J* = 8 Hz, 2H), 7.22-7.20 (d, *J* = 8 Hz, 2H), 6.88-6.86 (d, *J* = 8 Hz, 2H), 6.70 (s, 1H), 5.45-5.41 (m, 1H), 3.75 (s, 3H), 3.20-3.14 (dd, *J* = 16 Hz, *J* = 8 Hz, 1H), 2.79-2.74 (dd, *J* = 16 Hz, *J* = 8 Hz, 1H); ^{13}C NMR (100 MHz, $CDCl_3$) δ 183.28, 160.68, 132.76, 132.04, 131.39, 129.67, 128.47, 128.15, 126.54, 115.18, 74.07, 55.44, 38.78;
HRMS (ESI): calcd. for $[M+H]^+$ ($C_{17}H_{16}BrN_2O_2$) requires 359.0317, found 359.0383;
Enantiometric excess: 67%, determined by HPLC (Chiralpak AD-H column, hexane/*i*-PrOH 80:20, λ=254 nm, flow rate 1.0 mL/min; t_{major} = 23.3 min; t_{minor}=27.8 min).

(*S*)-2-(4-bromophenyl)-2-((*S*)-5-(4-methoxyphenyl)-3-oxopyrazolidin-1-yl)acetonitrile (7b)

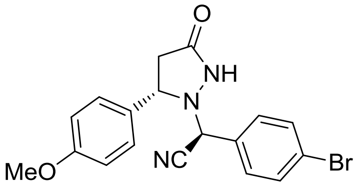

The compound **7b** was prepared according to **GP3** and was purified by flash chromatography (CH_2Cl_2 and Methanol). White solid; mp. 61-63 °C; 48% yield; $[\alpha]_D^{28.0}$ = -84.4 (c 1.50, $CHCl_3$); IR (neat): *v*3052, 2957, 2930, 2838, 1692, 1609, 1513, 1431, 1251, 1040 cm^{-1};^1H NMR (400 MHz, CD_3SOCD_3) δ 9.69 (s, 1H), 7.62-7.60 (d, *J* = 8 Hz, 1H), 7.37-7.35 (d, *J* = 8 Hz, 1H), 7.28-2.26 (d, *J* = 8 Hz, 1H), 6.90-6.88 (d, *J* = 8 Hz, 1H), 5.54 (s, 1H), 4.59-4.56 (dd, *J* = 8 Hz, *J* = 4 Hz, 1H), 3.68 (s, 3H), 3.27-3.21 (dd, *J* = 16 Hz, *J* = 8 Hz, 1H), 2.27-2.22 (dd, *J* = 16 Hz, *J* = 8 Hz, 1H); ^{13}C NMR (100 MHz, CD_3SOCD_3) δ
HRMS (ESI): calcd. for $[M+H]^+$ ($C_{18}H_{17}BrN_3O_2$) requires 386.0426, found 386.0493;
Enantiometric excess: 71%, determined by HPLC (ChiralpakPC-II column, hexane/*i*-PrOH 80:20, λ=254 nm, flow rate 1.0 mL/min; t_{major} = 36.6 min; t_{minor}=24.5 min).

(*R*, *Z*)-2-(4-bromobenzylidene)-3-(4-chlorophenyl)-5-oxopyrazolidin-2-ium-1-ide (6c)

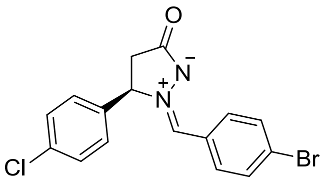

The compound **6c** was prepared according to **GP3** and was purified by flash chromatography (petroleum ether and ethyl acetate). White solid; mp. 69-72 ^{o}C;42% yield; $[\alpha]_D^{27.8}$ = 20.2 (c 1.50, $CHCl_3$); IR (neat): *v* 3052, 2925, 2853, 1678, 1586, 1490, 1303, 1088 cm^{-1};^{1}H NMR (400 MHz, $CDCl_3$) δ 8.03-8.01 (d, *J* = 8 Hz, 2H), 7.48-7.46 (d, *J* = 8 Hz, 2H), 7.35-7.33 (d, *J* = 8 Hz, 2H), 7.25-7.23 (d, *J* = 8 Hz, 2H), 6.71 (s, 1H), 5.51-5.48 (m, 1H), 3.25-3.18 (dd, *J* = 16 Hz, *J* = 8 Hz, 1H), 2.74-2.68 (dd, *J* = 16 Hz, *J* = 8 Hz, 1H); ^{13}C NMR (100 MHz, $CDCl_3$) δ 190.99, 183.00, 136.47, 135.93, 132.85, 132.15, 130.11, 128.25, 127.91, 126.97, 73.62, 53.43, 38.87;
HRMS (ESI): calcd. for $[M+H]^+$ ($C_{16}H_{13}BrClN_2O$) requires 362.9822, found 362.9889;
Enantiometric excess: 80%, determined by HPLC (Chiralpak AD-H column, hexane/*i*-PrOH 80:20, λ=254 nm, flow rate 1.0 mL/min; t_{major} = 15.2 min; t_{minor}= 19.8 min).

(*S*)-2-(4-bromophenyl)-2-((*S*)-5-(4-chlorophenyl)-3-oxopyrazolidin-1-yl)acetonitrile (7c)

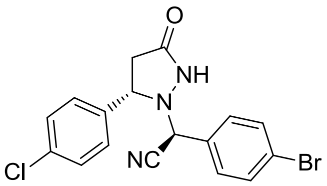

The compound **7c** was prepared according to **GP3** and was purified by flash chromatography (CH_2Cl_2 and Methanol). White solid; mp. 66-68 ^{o}C; 45% yield; $[\alpha]_D^{27.7}$ = -98.4 (c 1.50, $CHCl_3$); IR (neat): *v*3174, 3066, 2925, 2854, 1703, 1592, 1488, 1402, 1264 cm^{-1};^{1}H NMR (400 MHz, $CDCl_3$) δ 7.71 (s, 1H), 7.59-7.57 (d, *J* = 8 Hz, 2H), 7.40-7.36 (m, 6H), 4.85 (s, 1H), 4.56-4.52 (t, *J* = 8 Hz, 1H), 3.19-3.13 (dd, *J* = 16 Hz, *J* = 8 Hz, 1H), 2.52-2.46 (dd, *J* = 16 Hz, *J* = 8 Hz, 1H); ^{13}C NMR (100 MHz, $CDCl_3$) δ 173. 04, 136.64, 134.84, 132.59, 130.34, 129.65, 129.48, 128.37, 124.36, 114.97, 65.16, 60.44, 39.32;
HRMS (ESI): calcd. for $[M+H]^+$ ($C_{17}H_{14}BrClN_3O$) requires 389.9931, found 389.9997;
Enantiometric excess: 82%, determined by HPLC (ChiralpakPC-II column, hexane/*i*-PrOH 80:20, λ=254 nm, flow rate 1.0 mL/min; t_{major} = 25.9 min; t_{minor}=19.0 min).

(*R*, *Z*)-3-(4-chlorophenyl)-2-(4-methoxybenzylidene)-5-oxopyrazolidin-2-ium-1-ide (6d)

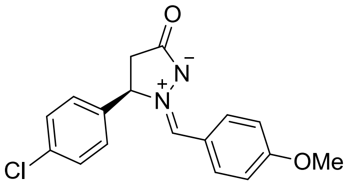

The compound **6d** was prepared according to **GP3** and was purified by flash chromatography (petroleum ether and ethyl acetate). White solid; 56-59 °C; 47% yield; $[\alpha]_D^{27.9}$ = 8.3 (c 1.50, $CHCl_3$); IR (neat): *v*3051, 2929, 2840, 1658, 1600, 1565, 1509, 1461, 1305, 1261 cm^{-1};^{1}H NMR (400 MHz, $CDCl_3$) δ 8.15-8.13 (d, *J* = 8 Hz, 2H), 7.33-7.31 (d, *J* = 8 Hz, 2H), 7.24-7.22 (d, *J* = 8 Hz, 2H), 6.85-6.83 (d, *J* = 8 Hz, 2H), 6.75 (s, 1H), 5.49-5.45 (m, 1H), 3.77 (s, 3H), 3.23-3.15 (dd, *J* = 16 Hz, *J* = 8 Hz, 1H), 2.71-2.66 (dd, *J* = 16 Hz, *J* = 8 Hz, 1H); ^{13}C NMR (100 MHz, $CDCl_3$) δ 182.43, 162.76, 137.13, 135.58, 134.07, 133.76, 129.96, 128.20, 121.99, 114.35, 72.70, 55.55, 39.28;
HRMS (ESI): calcd. for $[M+H]^+$ ($C_{17}H_{16}ClN_2O_2$) requires 315.0822 found 315.0890;
Enantiometric excess: 82%, determined by HPLC (Chiralpak AD-H column, hexane/*i*-PrOH 80:20, λ=254 nm, flow rate 1.0 mL/min; t_{major} = 16.9 min; t_{minor}=20.9 min).

(*S*)-2-((*S*)-5-(4-chlorophenyl)-3-oxopyrazolidin-1-yl)-2-(4-methoxyphenyl)acetonitrile (7d)

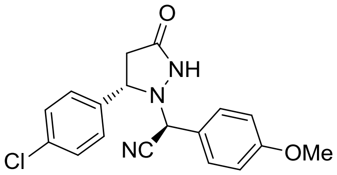

The compound **7d** was prepared according to **GP3** and was purified by flash chromatography (CH_2Cl_2 and Methanol). White solid; 61-63 °C; 45% yield; $[\alpha]_D^{28.2}$ = -96.3 (c 1.50, $CHCl_3$);IR (neat): *v*3065, 2959, 2930, 2839, 1611, 1512, 1463, 1422, 1252, 1178, 1090 cm^{-1};^{1}H NMR (400 MHz, $CDCl_3$) δ 7.33-7.31 (m, 6H), 6.87-6.85 (d, *J* = 8 Hz, 2H), 4.77 (s, 1H), 4.48-4.44 (t, *J* = 8 Hz, 1H), 3.74 (s, 3H), 3.11-3.05 (dd, *J* = 16 Hz, 1H), 2.45-2.39 (dd, *J* = 16 Hz, *J* = 8 Hz, 1H); ^{13}C NMR (100 MHz, $CDCl_3$) δ 172.74, 160.83, 136.87, 134.68, 129.40, 128.42, 123.18, 115.52, 114.75, 64.82, 60.26, 55.47, 39.37;
HRMS (ESI): calcd. for $[M+H]^+$ ($C_{18}H_{17}ClN_3O_2$) requires 342.0931, found 342.1000;
Enantiometric excess: 80%, determined by HPLC (ChiralpakPC-II column, hexane/*i*-PrOH 80:20, λ=254 nm, flow rate 1.0 mL/min; t_{major} = 32.1 min; t_{minor}=26.5 min).

(*S*, *Z*)-2-(4-bromobenzylidene)-5-oxo-3-phenethylpyrazolidin-2-ium-1-ide (6e)

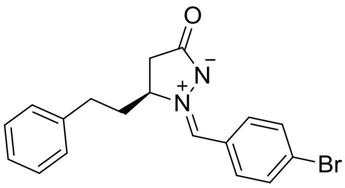

The compound **6e** was prepared according to **GP3** and was purified by flash chromatography (petroleum ether and ethyl acetate). White solid; 98-100 °C; 49% yield; $[\alpha]_D^{27.4}$ = 79.5 (c 1.50, $CHCl_3$); IR (neat): *v*3019, 2921, 2859, 1650, 1593, 1448, 1337, 1304, 1091 cm^{-1};^{1}H NMR (400 MHz, $CDCl_3$) δ 8.17-8.15 (d, *J* = 8 Hz, 2H), 7.57-7.55 (d, *J* = 8 Hz, 2H), 7.32-7.27 (m, 2H), 7.23-7.16 (m, 3H), 7.01 (s, 1H), 4.63-4.57 (m, 1H), 2.99-2.93 (dd, *J* = 16 Hz, *J* = 8 Hz, 1H), 2.77-2.73 (m, 2H), 2.62-2.57 (dd, *J* = 16 Hz, *J* = 4 Hz, 1H), 2.36-2.18 (m, 2H); ^{13}C NMR (100 MHz, $CDCl_3$) δ 190.27, 183.53, 139.25, 132.83, 132.13, 130.50, 128.88, 128.23, 128.12, 126.77, 126.56, 70.07, 37.64, 35.18, 31.09;

HRMS (ESI): calcd. for $[M+H]^+$ ($C_{18}H_{18}BrN_2O$) requires 357.0524, found 357.0591;

Enantiometric excess: 78%, determined by HPLC (ChiralpakOD-H column, hexane/*i*-PrOH60:40, λ=254 nm, flow rate 1.0 mL/min; t_{major} = 12.6 min; t_{minor}=9.7 min).

(*S*)-2-(4-bromophenyl)-2-((*R*)-3-oxo-5-phenethylpyrazolidin-1-yl)acetonitrile (7e)

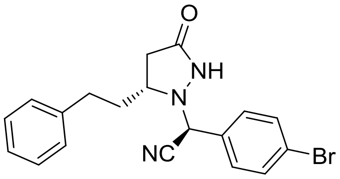

The compound **7e** was prepared according to **GP3** and was purified by flash chromatography (CH_2Cl_2 and Methanol). White solid; 52-55 °C; 48% yield; $[\alpha]_D^{28.1}$ = -46.6 (c 1.50, $CHCl_3$); IR (neat): *v*3061, 3026, 2926, 2855, 1693, 1592, 1488, 1453, 1423, 1263, 1072 cm^{-1};^{1}H NMR (400 MHz, $CDCl_3$) δ 8.03 (s, 1H), 7.57-7.55 (d, *J* = 8 Hz, 2H), 7.37-7.35 (d, *J* = 8 Hz, 2H), 7.31-7.28 (t, *J* = 8 Hz, 2H), 7.25-7.20 (m, 1H), 7.16-7.14 (d, *J* = 8 Hz, 2H), 4.75 (s, 1H), 3.58-3.53 (br, 1H), 3.24-3.17 (dd, *J* = 16 Hz, *J* = 8 Hz, 1H), 2.84-2.66 (m, 2H), 2.06-1.97 (m, 2H), 1.83-1.76 (m, 1H); ^{13}C NMR (100 MHz, $CDCl_3$) δ 175.00, 140.57, 132.48, 130.65, 129.92, 128.75, 128.27, 126.40, 124.19, 116.67, 62.54, 61.98, 36.98, 35.05, 31.78;

HRMS (ESI): calcd. for $[M+H]^+$ ($C_{19}H_{19}BrN_3O$) requires 384.0633, found 384.0700;

Enantiometric excess: 81%, determined by HPLC (ChiralpakPC-II column, hexane/*i*-PrOH 80:20, λ=254 nm, flow rate 1.0 mL/min; t_{major} = 22.1 min; t_{minor}=16.2 min).

Supplementary References

1.Xiao, H. *et al*. Asymmetric [3+2]cycloadditions of allenoates and dual activated olefins catalyzed by simple bifunctional N-acyl aminophosphines.*Angew.Chem. Int. Ed*. **49**, 4467-4470 (2010).

2. Han, X., Wang, Y., Zhong, F. & Lu, Y. Enantioselective [3+2]cycloaddotion of allenes to acrylates catalyzed by dipeptide-derived novel phosphines: Facile creation of functionalized cyclopentenes containing quaternary stereogenic centers.*J. Am. Chem. Soc*. **133**, 1726-1729 (2011).

3. Yao, W., Dou, X. & Lu, Y. Highly enantioselective synthesis of 3,4-dihydropyrans through a phosphine-catalyzed [4+2]annulation of allenoates and β,γ-unsaturated α-keto esters. *J. Am. Chem. Soc*. **137**, 54-57 (2015).

4.Yan, W. *et al*. Synthesis of N-alkoxycarbonylketoimines derived from isatins and their application in enantioselective synthesis of 3-aminooxindoles. *Org. Lett*. **14**, 2512-2515 (2012).

5. Liu, Y.-L. & Zhou, J. Organocatalytic asymmetric cyanation of isatin derived N-Bocketoimines. *Chem. Commun*. **49**, 4421-4423 (2013).

6. Wang, D. *et al*. The quinine thiourea-catalyzed asymmetric Strecker reaction: An approach for the synthesis of 3-aminooxindoles.*Adv. Synth. Catal*.**355**,548-558 (2013).

7. Shintani, R. &Fu,G. C. A new Copper-catalyzed [3+2]cycloaddition: Enantioselective coupling of terminal alkynes with azomethine imines to generate five-membered nitrogen heterocycles.*J. Am. Chem. Soc*. **125**, 10778-10779 (2003).

8. Suárez, A., Downey, C. W. & Fu, G. C. Kinetic resolutions of azomethine imines via Copper-catalyzed [3+2]cycloadditions. *J. Am. Chem. Soc*.**127**, 11244-11245 (2005).

9. Na, R. *et al*. Phosphine-catalyzed annulations of azomethine imines: Allene-dependent [3+2], [3+3], [4+3], and [3+2+3] pathways.*J. Am. Chem. Soc*. **133**, 13337-13348 (2011).

10. Li, N.-K. et al. EnantioselectiveStrecker-type reaction between azomethine imines and trimethylsilyl cyanide catalyzed by a cinchona alkaloid-derived thiourea bearing multiple hydrogen-bonding donors.*RSC Adv*.**3**, 9154-9157 (2013).

11. Wang, M., Huang, Z., Xu, J. & Chi, Y. R. N-heterocyclic carbine-catalyzed [3+4]cycloadditionand kinetic resolution of azomethine imines. *J. Am. Chem. Soc*. **136**, 1214-1217 (2014).
